# Sequence-based identification of inositol monophosphatase-like histidinol-phosphate phosphatases (HisN) in *Corynebacterium glutamicum*, *Actinobacteria*, and beyond

**DOI:** 10.1186/s12866-017-1069-4

**Published:** 2017-07-18

**Authors:** Robert Kasimir Kulis-Horn, Christian Rückert, Jörn Kalinowski, Marcus Persicke

**Affiliations:** Microbial Genomics and Biotechnology, Center for Biotechnology, Bielefeld University, Universitätsstraße 27, 33615 Bielefeld, Germany

**Keywords:** HisN, Cg0911, Histidinol-phosphate phosphatase (HolPase), Inositol monophosphatase (IMPase)-like, *Corynebacterium glutamicum*, Kinetic data, Sequence motifs, Phylogenetic analysis

## Abstract

**Background:**

The eighth step of l-histidine biosynthesis is carried out by an enzyme called histidinol-phosphate phosphatase (HolPase). Three unrelated HolPase families are known so far. Two of them are well studied: HAD-type HolPases known from *Gammaproteobacteria* like *Escherichia coli* or *Salmonella enterica* and PHP-type HolPases known from yeast and *Firmicutes* like *Bacillus subtilis*. However, the third family of HolPases, the inositol monophosphatase (IMPase)-like HolPases, present in *Actinobacteria* like *Corynebacterium glutamicum* (HisN) and plants, are poorly characterized. Moreover, there exist several IMPase-like proteins in bacteria (e.g. CysQ, ImpA, and SuhB) which are very similar to HisN but most likely do not participate in l-histidine biosynthesis.

**Results:**

Deletion of *hisN*, the gene encoding the IMPase-like HolPase in *C. glutamicum*, does not result in complete l-histidine auxotrophy. Out of four *hisN* homologs present in the genome of *C. glutamicum* (*impA*, *suhB*, *cysQ*, and *cg0911*), only *cg0911* encodes an enzyme with HolPase activity. The enzymatic properties of HisN and Cg0911 were determined, delivering the first available kinetic data for IMPase-like HolPases.

Additionally, we analyzed the amino acid sequences of potential HisN, ImpA, SuhB, CysQ and Cg0911 orthologs from bacteria and identified six conserved sequence motifs for each group of orthologs. Mutational studies confirmed the importance of a highly conserved aspartate residue accompanied by several aromatic amino acid residues present in motif 5 for HolPase activity. Several bacterial proteins containing all identified HolPase motifs, but showing only moderate sequence similarity to HisN from *C. glutamicum,* were experimentally confirmed as IMPase-like HolPases, demonstrating the value of the identified motifs. Based on the confirmed IMPase-like HolPases two profile Hidden Markov Models (HMMs) were build using an iterative approach. These HMMs allow the fast, reliable detection and differentiation of the two paralog groups from each other and other IMPases.

**Conclusion:**

The kinetic data obtained for HisN from *C. glutamicum,* as an example for an IMPase-like HolPases, shows remarkable differences in enzyme properties as compared to HAD- or PHP-type HolPases. The six sequence motifs and the HMMs presented in this study can be used to reliably differentiate between IMPase-like HolPases and IMPase-like proteins with no such activity, with the potential to enhance current and future genome annotations. A phylogenetic analysis reveals that IMPase-like HolPases are not only present in *Actinobacteria* and plant but can be found in further bacterial phyla, including, among others, *Proteobacteria*, *Chlorobi* and *Planctomycetes.*

**Electronic supplementary material:**

The online version of this article (doi:10.1186/s12866-017-1069-4) contains supplementary material, which is available to authorized users.

## Background

The gram-positive soil-bacterium *Corynebacterium glutamicum*, a member of the order *Corynebacteriales* within the taxonomical class *Actinobacteria* [1], plays an important role in industrial amino acid fermentation, with annual production scales of more than 2.5 and 1.5 million tons l-glutamate and l-lysine, respectively [2]. Strains for the production of further amino acids including l-alanine, l-isoleucine, l-phenylalanine, l-serine, l-tryptophan, and l-valine are available [3]. It is obvious that the in-depth understanding of the amino acid biosynthesis pathways and their regulation in this organism is necessary not only for further improvement of existing production strains, but also facilitates the development of new production strains, like for the production of l-histidine [4].

The entire l-histidine biosynthesis pathway is present in *C. glutamicum* and has been reviewed recently [5]. So far, all organisms known to synthesize l-histidine, including archaea, bacteria, yeast, and plants, use the same pathway for the biosynthesis. Although there are differences in gene organization, including several gene fusion events, most of the enzymes seem to have a common ancestor [5, 6]. One interesting exception is the histidinol-phosphate phosphatase (HolPase) [EC 3.1.3.15] catalyzing the eighth step of l-histidine biosynthesis, the dephosphorylation of l-histidinol-phosphate (HolP) to l-histidinol. Three unrelated HolPase families are known so far. *C. glutamicum* possesses a HolPase belonging to the family of inositol monophosphatase (IMPase)-like proteins, a subgroup of the FIG (FBPase/IMPase/GlpX-like domain) superfamily encoded by *hisN* [7, 8]. IMPase-like HolPases are a characteristic of the *Actinobacteria* and genera possessing a HisN homolog can be found in almost all taxonomical orders of this bacterial class [5]. Additionally, IMPase-like HolPases have been discovered in plants [9]. Functional characterizations of IMPase-like HolPases have been conducted in *C. glutamicum* [7], *Streptomyces coelicolor* [10], and *Arabidopsis thaliana* [9]. The HolPase activity of the HisN homolog in *Mycobacterium tuberculosis* (gene *Rv3137*) is supported at least indirectly, since it is not possible to delete this gene if a l-histidine free medium is used during the required selection steps [11].

Outside the *Actinobacteria*, there exist at least two further major classes of HolPases. The first class belongs to the HAD (Haloacid dehalogenase-like hydrolase) superfamily of proteins. The HAD-type HolPase activity is in general present on a bifunctional His(NB) enzyme that catalyzes the eighth and additionally the sixth step of l-histidine biosynthesis, the dehydration of imidazole glycerol-phosphate (IGP) [12]. The two activities are independent of each other with the HolPase and IGP dehydratase activities being found in the N-terminal and C-terminal domain of the bifunctional protein, respectively [13]. Bifunctional HAD-type HolPases are in general only found in *Gammaproteobacteria* [12], and have been extensively studied in *Salmonella enterica* serovar Typhimurium [14, 15] and *Escherichia coli* [13]. A monofunctional HAD-type HolPase has been discovered in the archaeon *Thermococcus onnurieneus* few years ago and homologs can be found in further archaeal genomes [16]. The second class of HolPases belongs to the PHP (polymerase and HolPase) subfamily of the metallo-dependent hydrolase (MDH) superfamily of proteins. The PHP-type HolPases are monofunctional and can be found in yeasts and in different bacterial lineages [12]. Examples for organisms with a well-studied PHP-type HolPase are *Saccharomyces cerevisiae* [17, 18], *Bacillus subtilis* [19], and *Lactococcus lactis* [20].

Our special interest in the corynebacterial HolPase arises from the observation that deletion of *hisN* in *C. glutamicum* results in pronounced l-histidine bradytrophy instead of complete auxotrophy [5]. A similar observation has been previously made with HolPase mutants of *S. cerevisiae,* resulting in the discovery of a second phosphatase with HolPase side activity [17]. Four HisN paralogs are encoded in the genome of *C. glutamicum* (Cg0911, SuhB, ImpA, and CysQ) [7, 21] and are therefore interesting candidates for alternative HolPases. The present study pursued three different aims: 1) The identification of an alternative HolPase in *C. glutamicum*; 2) The determination of the kinetic parameters of HisN in *C. glutamicum*, since up to our knowledge no such data has been reported for any IMPase-like HolPase so far; 3) The identification of one or more sequence motifs to reliably discriminate between IMPase-like HolPases and other IMPase-like proteins with no such activity.

## Results

### Genetic study on *hisN* and its four paralogs

During our previous investigation of different l-histidine gene deletion mutants of *C. glutamicum* [5], we observed that deletion of *hisN*, encoding the IMPase-like HolPase, does not result in l-histidine auxotrophy, but only in a pronounced bradytrophy of the mutant. Therefore, we started a closer investigation of the 8^th^ step of l-histidine biosynthesis in *C. glutamicum* in general and the Δ*hisN* mutant in particular.

**Fig. 1:**
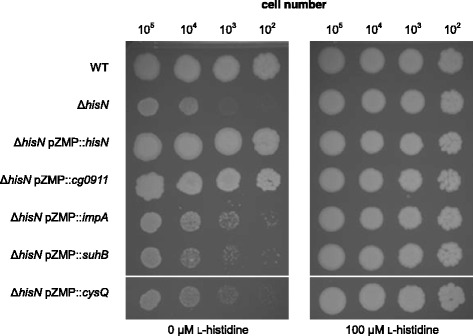
Comparative growth test of *C. glutamicum* Δ*hisN* mutants with plasmid-based expression of *hisN* or one of its four paralogs. Equal amounts of cells were placed on MM1 minimal medium plates with or without l-histidine supplementation and incubated for 6 days at 30 °C

Growth of the Δ*hisN* mutant was visible after several days of incubation on minimal medium plates without l-histidine. Addition of l-histidine abolished the observed growth defect completely (Fig. 1). The residual growth of the Δ*hisN* mutant was not specific to one single mutant, but was observed with every confirmed *hisN* deletion mutant constructed during this study and was also confirmed for an independently constructed Δ*hisN* strain [7] that slightly differed in the extend of the *hisN* deletion (data not shown). The genome of *C. glutamicum* contains four genes encoding putative HisN paralogs that have been already recognized in the original publication describing the HolPase activity of the *hisN* gene product [7]. All of them are grouped into the FIG superfamily of proteins according to their conserved domains and most of them are annotated as putative IMPases or fructose-1,6-bisphosphatases (Table 1). The degree of sequence similarity between HisN and one of its four putative paralogs is comparable in every case (24-26% identity, 37-41% similarity) with CysQ being least similar. In addition, all putative paralogs share the same degree of similarity if compared one to another. Since the four paralogs have so far not been analyzed for their function in *C. glutamicum*, we hypothesized that one of them might be responsible for the residual growth of the Δ*hisN* mutant.

**Table 1:** HisN paralogs in *C. glutamicum*

HisN paralog (gene)	protein length [aa]	sequence similarity on protein level (identity, similarity, BlastP score) [%], [%] [bits]					annotation	HolPase activity
		HisN	Cg0911	ImpA	SuhB	CysQ		
HisN (*cg0910*)	260	100, 100 515	26, 41 78	25, 41 86	27, 42 82	24, 37 52	HolPase ^a^	yes ^a, b^
Cg0911 (*cg0911*)	288 *	26, 41 77	100, 100 585	22, 35 58	27, 41 89	21, 30 84	putative IMPase ^c^ or archaeal FBPase ^d^	yes (very low) ^b^
ImpA (*cg2298*)	275	25, 41 81	22, 35 74	100, 100 561	22, 32 80	15, 26 35	putative IMPase ^c^ or archaeal FBPase ^d^	not detected
SuhB (*cg2090*)	280	27, 42 82	27, 41 89	22, 32 80	100, 100 567	23, 34 48	putative IMPase ^c^ or archaeal FBPase ^d^	not detected
CysQ (*cg0967*)	252	24, 37 52	21, 30 59	15, 26 35	23, 34 48	100, 100 521	putative PAPSPase ^c, d^	not detected

^*^ Based on RNAseq results, transcription of *cg0911* starts 9 nt downstream of the currently annotated *cg0911* CDS [5], resulting in a shorter Cg0911 protein (288 aa instead of 291 aa)

^a^ [7]

^b^ this study

^c^ GenBank: BX927147.1 [21]

^d^ GenBank: BA000036.3 [22]

PAPSPase = 3'-phosphoadenosine 5'-phosphosulfate (PAPS) 3'-phosphatase

Moreover, two of the putative *hisN* paralogs, namely *cg0911* and *impA*, form operons with other l-histidine biosynthesis genes. The *cg0911* gene is transcribed together with *hisN* and *impA* is part of the larger *hisHA-impA-hisFI-cg2294* transcription unit [5].

In order to test if any of the four putative *hisN* paralogs encodes a gene with HolPase activity, the genes were cloned into the constitutive shuttle expression vector pZMP (approximately 15 copies per cell, *tac* promoter). Sequencing of the inserts revealed that the *cg0911* gene sequence from the *C. glutamicum* wild type strain used in this study is identical to that presented in the *C. glutamicum* ATCC 13032 reference sequence BA000036.3 [22] and has two single nucleotide polyphormisms as compared to reference sequence BX927147.1 [21] (one silent mutation and one resulting in a G50R mutation). The resulting plasmids were isolated from *E. coli* and subsequently transferred into the *C. glutamicum* Δ*hisN* strain. Since it was not possible to obtain an error free *impA* insert in *E. coli* (i.e. frame shift or promoter mutations; data not shown) the pZMP::*impA* assembly mix was directly used for transformation of *C. glutamicum* Δ*hisN* resulting in the correct Δ*hisN* pZMP::*impA* mutant (checked by sequencing of the *impA* insert and the promoter region). A comparative growth test was conducted on minimal medium plates to check if one of the genes is able to complement the Δ*hisN* growth defect *in trans* (Fig. 1).

Expression of *impA*, *suhB* or *cysQ* did not improve the growth of the Δ*hisN* strain on minimal medium. Beside the complementation by *hisN* itself, a complementation of the Δ*hisN* growth defect was only observed with *cg0911*. However, growth of the Δ*hisN* pZMP::*cg0911* strain was slower compared to the WT. Single colonies of this strain appeared 24 h later on the plates and remained smaller in size, even if the incubation was prolonged (data not shown). Supplementation with l-histidine resulted in the same growth phenotype of all tested mutants and did not differ from the WT. These results suggest that *cg0911* is encoding an enzyme with weak HolPase activity.

**Fig. 2:**
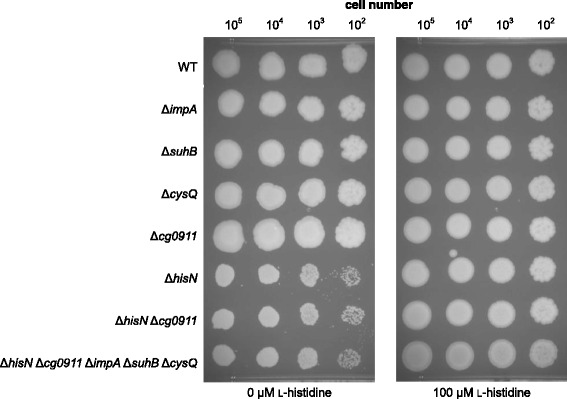
Comparative growth test of mutants with deletion of *hisN* or paralogous genes. Equal amounts of cells were placed on MM1 minimal medium plates with or without l-histidine supplementation and incubated for 6 days at 30 °C

To obtain further insight into the function of the different *hisN* paralogs, deletion mutants were constructed. Each paralog was separately deleted in the WT. In addition, a Δ*hisN* Δ*cg0911* double mutant and a quintuple mutant, lacking *hisN* and all its paralogs, were generated. Growth of the different mutants was again monitored on minimal medium plates (Fig. 2).

The single deletion of one of the *hisN* paralogs in the WT had no effect on growth of the mutants. Unexpectedly, we did not observe a further reduction of growth of the Δ*hisN* Δ*cg0911* double or the Δ*hisN* Δ*cg0911* Δ*impA* Δ*suhB* Δ*cysQ* quintuple mutant as compared to the Δ*hisN* single mutant. Supplementation with l-histidine resulted in the same growth of all mutants. None of the *hisN* paralogs was needed for normal growth of *C. glutamicum* under the tested conditions. Moreover, although the complementation assay clearly demonstrated HolPase activity of the *cg0911* gene product in vivo if expressed on a multiple copy plasmid, this activity does not account for l-histidine biosynthesis in a measurable degree if present in single copy under control of the native promotor.

### Enzymatic characterization of HisN and Cg0911

To the best of our knowledge, no kinetic data is available on the HolPase activity of HisN from *C. glutamicum* or any other organism possessing an IMPase-like HolPase. HolPase activity of the IMPase-like HolPases from *A. thaliana* and *S. coelicolor* has been deduced from complementation studies, and only the general phosphatase activity using the substrate *para*-nitrophenylphosphate (pNPP) has been demonstrated in vitro for the latter [9, 10]. Therefore, we determined the kinetic parameters of an IMPase-like HolPase with its natural substrate HolP using HisN_*Cg*_ as an example and comparing it to the HolPase activity of Cg0911, the second IMPase-like protein in *C. glutamicum* possessing HolPase activity.

Both proteins were heterologously expressed in *E. coli* and purified tag-free using the commercial IMPACT^™^ system. Purity and molecular weight of the purified proteins were estimated by one-dimensional SDS-PAGE (Additional file 1: Figure S1) and identity was confirmed by MALDI-TOF-MS analysis (data not shown). The activity of HisN and Cg0911 was assayed by the release of inorganic phosphate (P_i_) from HolP as described in Materials and Methods.

**Fig. 3:**
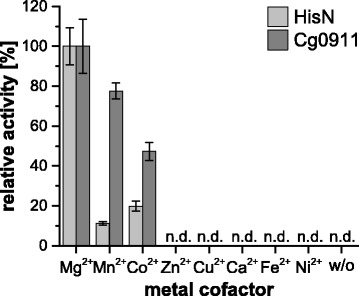
Relative activity of HisN (light grey) and Cg0911 (dark grey) in the presence of different divalent metal ions. Activity with Mg^2+^ was set to 100% for each protein individually. Assay conditions: 30 °C, pH = 7.35, 5 mM divalent metal ions, 500 μM HolP. n.d. = not detectable, w/o = without, n = 4

So far, all studied HolPases of the PHP- or HAD-type were shown to be dependent on divalent metal ions [13, 16, 20, 23]. The same holds true for eukaryotic and bacterial IMPases [11, 24, 25]. Therefore, in a first step, we evaluated the metal ion preference of HisN and Cg0911 as examples of IMPase-like HolPases (Fig. 3).

Both enzymes were inactive if metal ions were omitted from the reaction mixture. Presence of 10 mM EDTA also resulted in no activity (data not shown). In the presence of 5 mM Mg^2+^, Mn^2+^, or Co^2+^, release of P_i_ was detected. HisN showed a clear preference towards Mg^2+^ (100% activity) over Co^2+^ (20% activity) and Mn^2+^ (11% activity). The metal ion preference of Cg0911 was less pronounced. The enzyme still exhibited 78% of its maximal activity in the presence of Mn^2+^ and 47% in the presence of Co^2+^. No release of P_i_ from HolP was detectable in the presence of Zn^2+^, Cu^2+^, Ca^2+^, Fe^2+^, or Ni^2+^ with either enzyme.

**Fig. 4:**
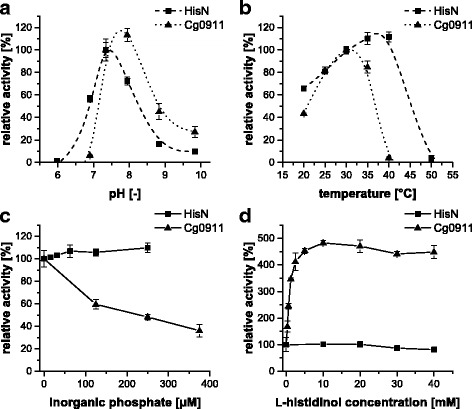
Enzyme characteristics of HisN (■) and Cg0911 (▲). **a** Effect of different pH values. The activity at pH 7.35 was set to 100% for each protein individually. Assay conditions: 30 °C, 5 mM MgSO_4_, 500 μM HolP, n = 4. **b** Effect of incubation temperature. The activity at 30 °C was set to 100% for each protein individually. Assay conditions: pH = 7.35, 5 mM MgSO_4_, 500 μM HolP, n = 4. **c** Influence of inorganic phosphate (P_i_) and **d** influence of l-histidinol. The activity without the addition of P_i_ or l-histidinol was set to 100% for each enzyme individually. Assay conditions: 30 °C, pH = 7.35, 5 mM MgSO_4_, 250 μM (HisN) and 500 μM (Cg0911) HolP. 1 M TEA-puffer instead of 0.1 M was used in the l-histidinol experiments. n = 3

Next, activity of HisN and Cg0911 was assayed in response to the pH of the reaction buffer (Fig. 4a). The buffering substances were adapted to the intended pH values. HisN exhibited maximal activity at pH 7.35. HisN activity decreases almost uniformly beyond the optimal pH, with no activity present at around pH 6 and reduced to 10% at around pH 10. The pH profile of Cg0911 was shifted to the alkaline conditions by 0.5 to 1 pH units. Maximal Cg0911 activity was observed at around pH 8 and was only little reduced at pH 7.35, followed by a sharp loss in activity towards more acidic conditions. The drop in activity towards more alkaline conditions was less pronounced and the enzyme exhibited still 30% of its activity at around pH 10. Since both HisN and Cg0911 were highly active at pH = 7.35, and this pH value corresponded well to the internal pH value of 7.5 ± 0.5 in *C. glutamicum* [26], a pH of 7.35 was kept constant during all further measurements.

The activity of HisN and Cg0911 was determined in a temperature range from 20 to 50 °C (Fig. 4b). Maximal HisN activity was reached at 35–40 °C. No activity was observed at 50 °C, indicating heat denaturation of the protein. The HolPase activity of Cg0911 was even more heat sensitive. Maximal Cg0911 activity was reached at 30 °C and less than 5% of this activity remained at 40 °C. To retain comparability between the two enzymes, all following measurements were conducted at 30 °C, since both enzymes were active at this temperature and it reflects the optimal growth temperature of *C. glutamicum*.

The turnover number (k_cat_) of HisN and Cg0911 in the presence of Mg^2+^, as well as the HolP and Mg^2+^-concentrations necessary for half maximal enzyme activity (K_m_ values for HolP and Mg^2+^) were determined (Table 2). The parameters were obtained by non-linear curve fitting of the data points to the Hill-equation [27].

**Table 2:** Kinetic parameters of HisN and Cg0911 at 30 °C and pH 7.35, *n* = 6

		HisN	Cg0911
	k_cat_ [s^−1^]	1.04 ± 0.06	0.013 ± 0.002
for HolP	K_m_ [μM]	23.6 ± 1.4	638.3 ± 70.5
	k_cat_/K_m_ [s^−1^ M^−1^]	4.41 × 10^4^	1.98 × 10^1^
	Hill coefficient	1.47 ± 0.18	1.83 ± 0.13
for Mg^2+^	K_m_ [μM]	644.8 ± 22.4	5155.8 ± 117.9
	k_cat_/K_m_ [s^−1^ M^−1^]	1.61 × 10^3^	2.46 × 10^0^
	Hill coefficient	2.44 ± 0.20	3.04 ± 0.12

HisN was very specific towards HolP with a K_m_ value of about 25 μM. The k_cat_ value was around 1 s^−1^ resulting in a catalytic efficiency of the enzyme of 4.41 × 10^4^ s^−1^ M^-1^. The Hill coefficient of HisN regarding HolP was around 1.5 indicating only a little cooperative effect.

The HolPase activity of Cg0911 was almost 80-times lower compared to HisN and the K_m_ value for HolP was around 650 μM, resulting in a catalytic efficiency of 1.98 × 10^1^ s^−1^ M^−1^. The Hill coefficient of around 1.8 hints to some cooperative effect of HolP on Cg0911 HolPase activity. The kinetic parameters for Cg0911 indicate that HolP is not the preferred substrate of this protein and the ability to hydrolyze HolP might reflect only a side activity of the enzyme.

We also tested the affinity of the two enzymes towards bivalent magnesium ions. The K_m_ values for Mg^2+^ were about 650 μM and 5000 μM for HisN and Cg0911, respectively. They were about 30-times and 10-times higher compared to the K_m_ values for HolP, respectively. The Hill coefficients regarding Mg^2+^ were around 2.4 for HisN and around 3.0 for Cg0911, indicating pronounced cooperativity of both enzymes in respect to the metal ion. This assumption is reinforced by the observation that no HisN or Cg0911 activity was measurable at Mg^2+^ concentrations ≤ 100 μM or ≤ 625 μM, respectively (data not shown).

Neither HisN nor Cg0911 showed any phosphatase activity against the general phosphatase substrate *para*-nitrophenyl phosphate (data not shown). The ability to hydrolyze other natural phosphatase substrates was not tested.

Finally, the potential inhibition of HisN and Cg0911 by l-histidine or the two direct reaction products l-histidinol and P_i_ was examined. No inhibitory effect of l-histidine was observed with concentrations up to 60 mM l-histidine (data not shown). A different effect of the addition of P_i_ and l-histidinol to the reaction mixture was observed for HisN and Cg0911. While HisN was not inhibited by P_i_ up to a concentration of 250 μM (higher concentrations were not tested for HisN), activity of Cg0911 decreased to 40% at 375 μM P_i_ (Fig. 4c). Unfortunately, it was not possible to test the effect of higher P_i_ concentrations, since the addition of external P_i_ interferes with the detection of P_i_ released during hydrolysis of HolP. It cannot be excluded, that HisN is inhibited by P_i_ concentrations > 250 μM.

HolPase activity of HisN was also not affected by the presence of l-histidinol (Fig. 4d). The enzyme was fully active up to 20 mM l-histidinol. The slight reduction to 80% activity at 40 mM most likely reflects a pH artifact, since HisN activity is optimal at pH 7.35 and rising l-histidinol concentrations cause a drop in pH even in 1 M TEA buffer (pH 7.5 and pH 7.0 at 0 mM and 200 mM l-histidinol, respectively; estimated with pH indicator stripes at RT).

Surprisingly, we observed a stimulating effect of l-histidinol on the HolPase activity of Cg0911. The activity increased almost five-fold at l-histidinol concentrations ≥ 10 mM. Half maximal stimulation was reached at 0.86 ± 0.06 mM l-histidinol. Since no release of P_i_ was detectable if the substrate HolP was omitted from the assay (data not shown), any contamination of the l-histidinol reagent with P_i_ or other phosphorous substances can be excluded. It appears therefore, that Cg0911 is positively feedback regulated by l-histidinol.

### Identification of sequence motifs for the discrimination of IMPase-like HolPases from other IMPase-like proteins in *C. glutamicum* and other bacteria

The presence of several IMPase-like proteins in one species (e.g. five in *C. glutamicum*) complicates the discrimination between an IMPase-like HolPase and IMPase-like proteins with different substrate specificities. Within the class *Actinobacteria*, it is rather easy to identify the HolPases due to a much higher sequence similarity to HisN_*Cg*_ than to the other IMPase-like proteins. However, this becomes more difficult in other bacterial phyla or even in different kingdoms. Therefore, we were interested in the identification of amino acid motifs that allow the unambiguous discrimination of IMPase-like HolPases purely based on the protein sequence.

For each of the five IMPase-like proteins *in C. glutamicum* (HisN, Cg0911, SuhB, ImpA, and CysQ) we performed a multiple sequence alignment of potential orthologs from a wide range of bacteria to identify highly conserved amino acid residues. The comparison of the highly conserved residues in each group of orthologs allowed the determination of six amino acid motifs distributed over the entire protein sequence that can be used for the discrimination of HisN orthologs from other IMPase-like proteins. Orthologs were identified by a BlastP search using the respective protein sequence from *C. glutamicum* as query. A BlastP score ≥ 125 was set as cut-off for identification. This cut-off was chosen, because it was sufficient to reliably distinguish between HisN and the other IMPase-like proteins in *C. glutamicum*, *M. tuberculosis,* and *S. coelicolor* (data not shown). With very few exceptions, maximum one (HisN, SuhB, and CysQ) or three sequences (Cg0911 and ImpA) per genus were randomly chosen for the multiple sequence alignment (see Additional file 2 for a complete list of used sequences).

Since we were most interested in motifs for the identification of IMPase-like HolPase, only the HisN motifs will be described in detail below.

Motif 1 consist of a strictly conserved lysine (Lys36), a highly conserved aspartate (Asp38), a threonine or serine at position 40, followed by a highly conserved proline (Pro41), a strictly conserved valine (Val42) and threonine or serine at position 43. An aspartate at position 46 is strictly conserved in all analyzed IMPase-like proteins and can be used for positioning of motif 1. Interestingly, motif 1 is completely absent in some of the HisN orthologs (11 out of 147 analyzed sequences mostly from *Alpha*- or *Gammaproteobacteria*). However, a different conserved motif is present in these cases consisting of lysine at position 34 or 35, an aromatic amino acid at position 40 and aspartate at position 41 followed by valine (Val42) and threonine (Thr43) (not shown).

Motif 2 consist of a highly conserved glycine (Gly68) followed by two strictly conserved glutamate residues (Glu69 and Glu70). Motif 2 is very similar in all analyzed groups of orthologs, with the exception of CysQ, where the conserved glycine is replaced by a strictly conserved serine (Ser64) and preceded by a highly conserved leucine (Leu63). Therefore, motif 2 is most suitable for the discrimination of HisN homologs from CysQ orthologs.

Motif 3 contains four of the active site key residues typical of all IMPase-like proteins (HisN_*Cg*_: Asp85, Ile87, Asp88, and Thr90) [28, 29]. But not only these four residues are strongly conserved in HisN and the other IMPase-like proteins, but all residues ranging from positions 82 to 90. Therefore motif 3 is most suited for the identification of IMPase-like proteins in general. Striking differences between the different ortholog-groups within motif 3 appear only at position 91. There is a preference for a lysine at this position in HisN orthologs.

A highly conserved motif 4 is only present in CysQ orthologs. However, there exists a motif 4 in HisN orthologs, too. It consist of a moderately conserved arginine (Arg95), a strongly conserved glycine (Gly96) and proline (Pro98), followed by an aromatic amino acid at position 100, a strongly conserved threonine (Thr102) and a strongly conserved leucine (Leu103). Especially the combination of the aromatic amino acid at position 100 followed by Thr102 and Leu103 is very typical of HisN orthologs.

Motif 5 is the most characteristic motif of HisN orthologs. It consists of a highly conserved arginine (Arg187; replaced by Val or Leu in many alphaproteobacteria), a highly conserved glycine (Gly190) and an almost strictly conserved aspartate (Asp191). Only in some sequences of *Gammaproteobacteria* Asp191 is replaced with glutamate. Neither aspartate nor glutamate was found at this position in any other of the analyzed IMPase-like protein sequences. Moreover, several aromatic amino acids are present in motif 5 of HisN orthologs. One of these aromatic amino acids is present at position 188 or more likely 189, with the respective other position being occupied by a small residue (mostly glycine or alanine). Two more aromatic residues are usually present at position 193 and 195. Especially in actinobacterial HisN orthologs, there is also an additional aromatic amino acid at position 192. Whereas usually only phenylalanine, tyrosine or tryptophan residues are present at positions 188, 189, 192 and 193 the aromatic amino acid histidine might be present at position 195. No aromatic amino acids are present at the positions 192–195 in the corresponding motifs of the other IMPase-like proteins. Therefore, this motif is very specific for HisN orthologs. Next to the already described characteristics, the HisN-specific motif 5 is lacking a highly conserved aspartate followed by a leucine residue that are present in Cg0911, SuhB and ImpA orthologs (Asp203 and Leu204 in SuhB_*Cg*_). A specific motif 5 can also be identified in the other analyzed groups of IMPase-like proteins. Two consecutive arginine residues (Arg195 and Arg196 in SuhB_*Cg*_), followed by the sequence GSAAL, are very typical of SuhB orthologs. On the other hand, two arginine residues interspaced by a non-conserved amino acid (Arg179 and Arg181 in ImpA_*Cg*_) are very typical of ImpA orthologs.

The last motif, motif 6, is very similar in all analyzed IMPase-like ortholog groups. It contains the strictly conserved aspartate residue (Asp215) involved in coordination of the metal ions in the active site [28, 29]. Most interesting for discrimination between HisN and the other groups is position 219. Whereas a very highly conserved glycine (Cg0911, SuhB or ImpA) or a proline (CysQ) is usually present at this position in the other groups of orthologs, no glycine was present at position 219 in any of the analyzed HisN orthologs.

Although we included only sequences of bacterial IMPase-like HolPases in our motif search, all six identified HolPase motifs can also be found in the protein sequence of HISN7 from the plant *A. thaliana* (Additional file 1: Figure S2). HISN7_*At*_ has been previously experimentally confirmed as IMPase-like HolPase [9], despite its low overall sequence similarity to HisN_*Cg*_ (24% identity, 36% similarity, BlastP-score: 103 bits).

### Identification of IMPase-like HolPases based on the described sequence motifs and experimental validation of HolPase activity by complementation experiments

In order to prove the value of the identified HolPase motifs, different potential HisN orthologs were tested for their ability to complement a *C. glutamicum* Δ*hisN* strain, thus demonstrating HolPase activity of the respective gene products (Table 3). The potential HolPase genes from the actinobacterium *Dietzia* sp. strain Chol2 (genome announcement in preparation; preliminary locus tag *Dietzia_sp.-Draft_1801*, here referred to as *hisN*_*Dz*_) and the alphaproteobacterium *Zymomonas mobilis* ZM4 ([30]; locus tag *ZMO_RS06805*, here referred to as *hisN*_*Zm*_) were chosen, because the HolPase motifs are conserved in the respective gene products despite a relatively low overall sequence similarity to HisN_*Cg*_ *.*

**Table 3:** List of potential HisN orthologs. Characteristic amino acid residues of HolPase motif 5 are underlined. HolPase activity was inferred from the ability of the corresponding gene to complement a *C. glutamicum* Δ*hisN* mutant

potential HisN ortholog	protein length [aa]	sequence similarity on protein level (identity, similarity, BlastP score) [%], [%] [bits]					HolPase motif 5	HolPase activity
		HisN	Cg0911	ImpA	SuhB	CysQ		
*Dietzia* sp. strain Chol2 HisN_*Dz*_	278	29, 47 105	25, 40 76	23, 33 69	26, 38 49	23, 35 46	yes R**F**G**GD**C**Y**A**Y**	yes
*Zymomonas mobilis* HisN_*Zm*_	259	32, 48 101	22, 35 69	22, 36 58	26, 40 58	24, 37 62	yes LLG**GD**C**Y**N**Y**	yes
*Actinoplanes utahensis* HisN_*Au*_	266	55, 68 261	27, 40 76	26, 40 88	26, 40 71	26,32 56	yes RA**YGDFY**G**Y**	yes
*Actinoplanes utahensis* HisN2_*Au*_	259	36, 51 119	24, 35 70	24, 39 61	27, 36 67	24, 37 58	no ------QPS	no

In addition, we investigated potential HisN orthologs from *Actinoplanes utahensis* NRRL 12052 [31]. This actinobacterium possesses two genes encoding IMPase-like proteins that are most similar to HisN_*Cg*_ *.* The first gene product (locus tag *MB27_13025*, referred to as HisN_*Au*_) is characterized by a high sequence similarity to HisN_*Cg*_ and the presence of all six HolPase motifs. The second gene product (locus tag *KHD72131.1*, for convenience reasons referred to as HisN2_*Au*_) is also more similar to HisN_*Cg*_ then to another IMPase-like proteins in *C. glutamicum* and five of the six identified motifs are at least moderately conserved. However, motif 5 is absent in this protein (Table 3, Additional file 1: Figure S3).

**Fig. 5:**
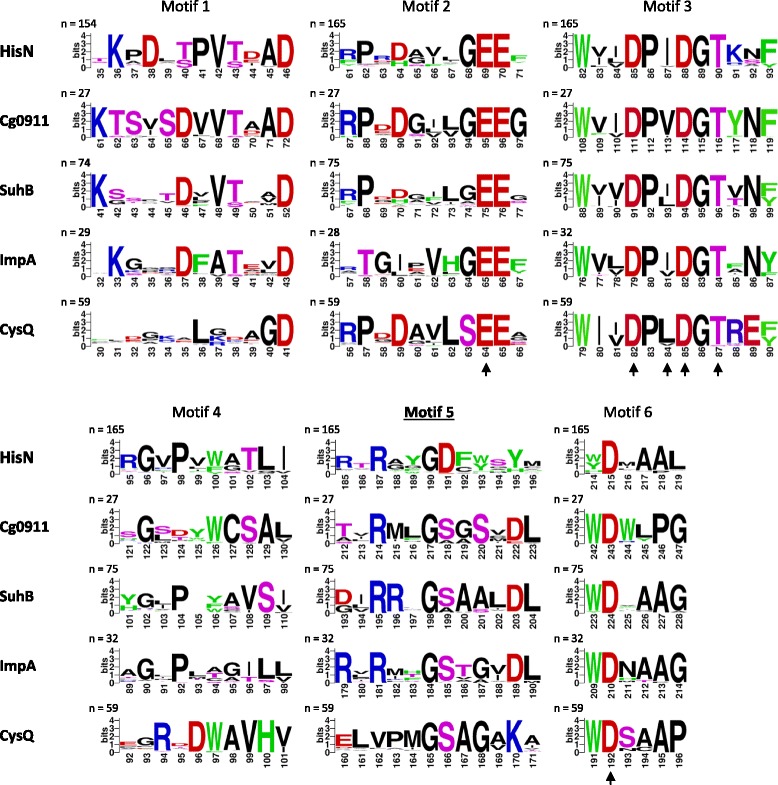
Conserved amino acid residues in groups of potential HisN, Cg0911, SuhB, ImpA, and CysQ orthologs that can be used for the discrimination of the different protein groups. Numbering of the amino acids corresponds to the five proteins from *C. glutamicum*. The arrows indicate active site key residues that are involved in binding of the three catalytic metal ions, the phosphate group of the substrate and/or the activation of the water molecule for hydrolysis of the phosphate ester bond as derived from the solved crystal structures of various IMPases [28, 29]. Coloring: blue = Arg, Lys; red = Asp, Glu; pink = Ser, Thr; green = His, Phe, Trp, Tyr; black = all remaining

The above described genes were cloned into the constitutive pZMP vector and tested for their ability to complement the l-histidine bradytrophic growth phenotype of the *C. glutamicum* Δ*hisN* mutant. As expected, *hisN*_*Dz*_, *hisN*_*Zm*_, and *hisN*_*Au*_ were able to fully complement the *C. glutamicum* Δ*hisN* mutant (data not shown). In contrast, the expression of *hisN2*_*Au*_ failed to complement the *C. glutamicum* Δ*hisN* mutant, even though the overall similarity of the gene product to HisN_*Cg*_ is higher than that of HisN_*Dz*_ and HisN_*Zm*_. These results underline the importance of the motif 5 (Fig. 5) for HolPase activity of IMPase-like proteins.

Our continuing analyses revealed that many IMPase-like proteins within NCBI’s non-redundant protein database are either not classified in more detail (mostly only as IMPases or IMPase-like proteins) or are even wrongly classified. Some examples of misclassified IMPases are given in Table 4. By comparing the amino acid sequence of these IMPase-like proteins to the five IMPase-like proteins in *C. glutamicum* and by checking for the presence of the expected motifs, we were able to assign a more accurate function to these proteins.

**Table 4:** List of randomly chosen bacterial IMPase-like proteins that are most likely wrongly annotated with a focus on HolPases. All proteins have been compared to the five IMPase-like proteins in *C. glutamicum* and reannotated according to the detected motifs

Organism	GI-number	current annotation	protein with highest similarity in *C. glutamicum*	presence of expected motifs	similarity to HisN_*Cg*_ (BlastP score) [bits]	new functional assignment
Class 1						
*Pseudomonas pseudoalcaligenes*	489546409	IMPase	HisN_*Cg*_	yes	117	HisN
*Rudaea cellulosilytica*	648601632	IMPase	HisN_*Cg*_	yes	108	HisN
*Marinobacter psychrophilus*	860341079	IMPase	HisN_*Cg*_	yes	110	HisN
*Rubrobacter radiotolerans*	627778235	FBPase- or IMPase	HisN_*Cg*_	yes	123	HisN
Class 2						
*Streptosporangium roseum*	502655523	IMPase	HisN_*Cg*_	no	120	-
*Rubrobacter xylanophilus*	499884516	IMPase	HisN_*Cg*_	no	120	-
Class 3						
*Sediminibacterium salmoneum*	739492005	HolPase	SuhB_*Cg*_	yes	102	SuhB
*Flavihumibacter solisilvae*	743029970	HolPase	SuhB_*Cg*_	yes	91	SuhB
*Desulfatibacillum aliphaticivorans*	654864062	HolPase	SuhB_*Cg*_	yes	102	SuhB
*Leptospirillum ferriphilum*	738123182	HolPase	SuhB_*Cg*_	yes	89	SuhB

This list demonstrates that many IMPase-like HolPases are not recognized as such in the databases (class 1). By checking for the presence of the HolPase motifs it is possible to accurately classify even those HisN homologs that show only a moderate overall similarity to HisN_*Cg*_ (BlastP score < 125 bits). On the other hand, there are also many examples of IMPase-like proteins that have been wrongly annotated as HolPases. Two classes can be distinguished here. The first class (class 2) consists of proteins which indeed are most similar to HisN_*Cg*_, however the overall sequence similarity is rather low (BlastP scores usually < 125 bits). Most importantly, HolPase motif 5 is missing in these proteins. Next to the two examples given in Table 4, HisN2_*Au*_ (Table 3) also belongs to this class 2 of misclassified proteins. Since *hisN2*_*Au*_ was unable to complement the *C. glutamicum* Δ*hisN* strain, all HisN homologs belonging to class 2 most likely do not exhibit HolPase activity. Their substrate specificity remains to be elucidated. The second class of wrongly annotated HolPases (class 3) includes sequences which have been simply misclassified. They exhibit a comparably low sequence similarity to HisN_*Cg*_ (BlastP scores usually < 100 bits), are indeed more similar to SuhB_*Cg*_, and possess the motifs typical of SuhB orthologs.

### Survey of the crystal structure of HisN_*Zm*_ focusing on the conserved HolPase motifs

To get a better understanding of the putative function of some of the conserved residues within the six detected HolPase motifs, we had a closer look on the IMPase-like HolPase from *Z. mobilis* (HisN_*Zm*_). HisN_*Zm*_ is only moderately similar to HisN_*Cg*_ but all HolPase motifs are present (Additional file 1: Figure S2) and we were able to experimentally verify its HolPase activity (see above). The crystal structure of this protein has been solved recently by Hwang et al. in 2014. Up to date, it represents the only solved crystal structure of an IMPase-like HolPase. There is evidence from the crystal structure as well as from gel filtration experiments that native HisN_*Zm*_ is a homodimer [32]. Notably, Hwang et al. did not recognize HisN_*Zm*_, which they refer to as CbbF, being a HolPase. The protein has been crystallized by Hwang and coworkers in its *apo* form without metal ions, which are needed for enzymatic activity, or any substrate [32]. However, the crystal structure contains a sulfate ion at the position which most likely resembles the binding site of the substrate’s phosphate group [32].

**Fig. 6:**
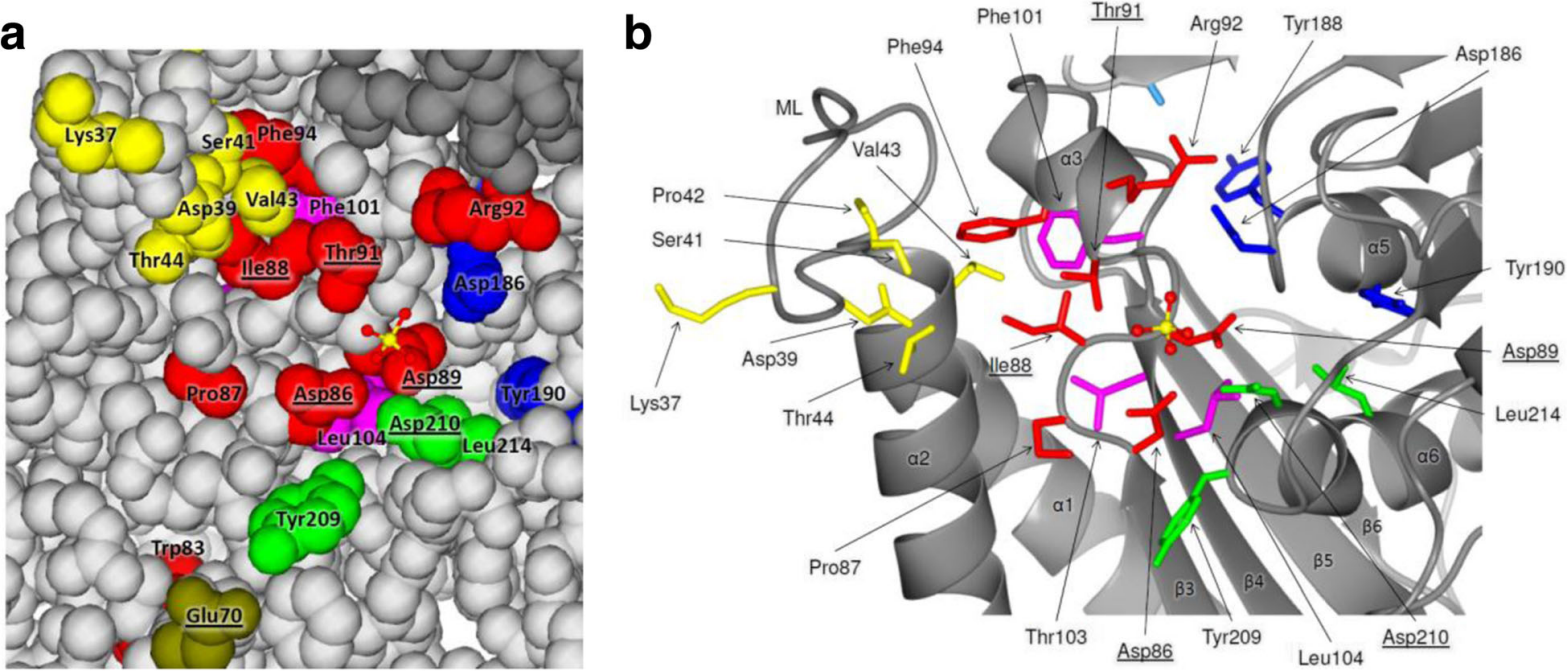
Probable active site of HisN_*Zm*_ depicted as space-filling model (**a**), illustrating surface exposed residues, and as ribbon diagram with stick representation of selected residues (**b**) based on the crystal structure of the homodimer as determined by Hwang et al. (2014) [32]. The two monomers are depicted in different gray shades. The side chains of highly conserved residues typical of IMPase-like HolPases are highlighted: yellow = motif 1, olive = motif 2, red = motif 3, pink = motif 4, blue = motif 5, green = motif 6 (compare Fig. 5). The location of the sulfate ion (ball-and-stick model) represents the most likely binding site of the phosphate moiety of the substrate HolP. Key active site residues involved in binding of metal ions (not present in the HisN_*Zm*_ crystal), the substrates phosphate moiety and activation of the water molecule for ester hydrolysis, as derived from the structures of different IMPases [28, 29], are underlined

We examined the localization of the highly conserved residues of the six HolPase motifs in the HisN_*Zm*_ crystal structure and investigated their putative interactions with other residues. Fig. 6 shows a part of the HisN_*Zm*_ homodimer, centered on one of the two identical supposed active sites depicted as space-filling model (**a**) and as ribbon diagram with stick representation of selected residues (**b**).

Many of the highly conserved residues within the HolPase motifs are located close to the active center as indicated by the location of the sulfate ion. This sulfate ion is forming hydrogen bonds with Asp86, Asp89, Gly90, Thr91 (corresponding to position 85, 88, 89 and 90 of the HisN-specific motif 3; Fig. 5) and Asp210 (motif 6; position 215). A part of these residues, as well as Ile88 (motif 3; position 87) and Glu70 (motif 2; position 69), are supposed to be involved in coordination of three catalytic Mg^2+^ ions according to the known structures of different IMPases [28, 29].

The side chains of most of the highly conserved residues of motif 1 (Lys37, Asp39, Ser41, Val43 and Thr44; corresponding to positions 36, 38, 40, 42, and 43 in Fig. 5) point to the side of the enzyme where the active site is located. This part of the enzyme (residues 29–41) has been recognized as a mobile catalytic loop in different IMPases-like proteins which changes its spatial position in response to binding of metal ions or the substrate [33]. Therefore, these residues might play a crucial role in recognition of the substrate HolP.

Several of the conserved aromatic amino acids within the different motifs seem to play an important role for the formation of the tertiary and quaternary structure of HisN_*Zm*_ and IMPase-like HolPases in general. For example, Phe94, corresponding to the conserved aromatic amino acid at position 93 in the HisN-specific motif 3 (Fig. 5), has hydrophobic interactions with nine other amino acids (including residues from the motifs 1, 3 and 4). Two of these interactions are additionally stabilized by aromatic-aromatic interactions. All these interactions connect the α-helices 1, 2, and 3 with the active site key residue Ile88 and thereby contribute to the formation of the active site. A similar structural function might be attributed to some of the conserved branched chain amino acids. Leu214, for instance, which is very typical of IMPase-like HolPases (motif 6, position 219), has hydrophobic interactions with the likewise conserved Leu103 (motif 4, position 103) and three additional residues. One of the residues interacting with Leu214 is Tyr190, one of the aromatic amino acids highly conserved within motif 5 of IMPase-like HolPases (motif 5, position 195). Therefore, this aromatic amino acid might primarily have a structural function. However, Tyr190 is the only of the typically three conserved aromatic amino acids within motif 5 that is at least partially exposed to the surface and located close to the supposed substrate binding site (Fig. 6a). It is therefore possible that Tyr190 is additionally involved in substrate recognition, possibly by aromatic interaction with the likewise aromatic substrate HolP. The two other highly conserved aromatic amino acids within motif 5 of IMPase-like HolPases correspond to Tyr188 (motif 5, position 193) and Leu183 (motif 5, position 188) in HisN_*Zm*_. The side chains of both residues are located on the “back side” of the enzyme and distant from the active site. Leu183 is interacting with several other hydrophobic amino acids from the second subunit of the HisN_*Zm*_ homodimer (among others with Leu183 itself). Tyr188 has some intramolecular hydrophobic and aromatic interactions stabilizing the tertiary structure, but it additionally forms a hydrogen bond with Arg29 from the second subunit. An important role of Leu183 and Tyr188 might therefore be the stabilization of the quaternary structure of the HisN_*Zm*_ homodimer.

Most important, the analysis of the HisN_*Zm*_ crystal structure suggest a direct involvement of Asp186 (motif 5, position 191) in substrate recognition. The Asp186 side chain is accessible to the solvent, points towards the supposed substrate binding site (Fig. 6a), and there is no indication that the carboxylic group is involved in the formation of any H-bonds or salt bridges. Consequently, Asp186 would be available for interaction with the substrate HolP, for instance by the formation of H-bonds between the amino group of HolP and the carboxylic group of the aspartate. This is in good agreement with our observation that replacement of the conserved Asp191 in HisN_*Cg*_ with alanine, serine, or asparagine, but not glutamate, results in a considerably reduced ability of the gene products to complement a *hisN* deletion in *C. glutamicum* (Additional file 1: Figure S4). However, since there is no crystal structure available of any IMPase-like HolPase in complex with catalytic metal ions and the substrate HolP or at least the products l-histidinol or P_i_, any interaction between HolP and Asp186 (and possibly Tyr190) remains speculative.

### Distribution of HisN and Cg0911 orthologs within *bacteria*

**Fig. 7:**
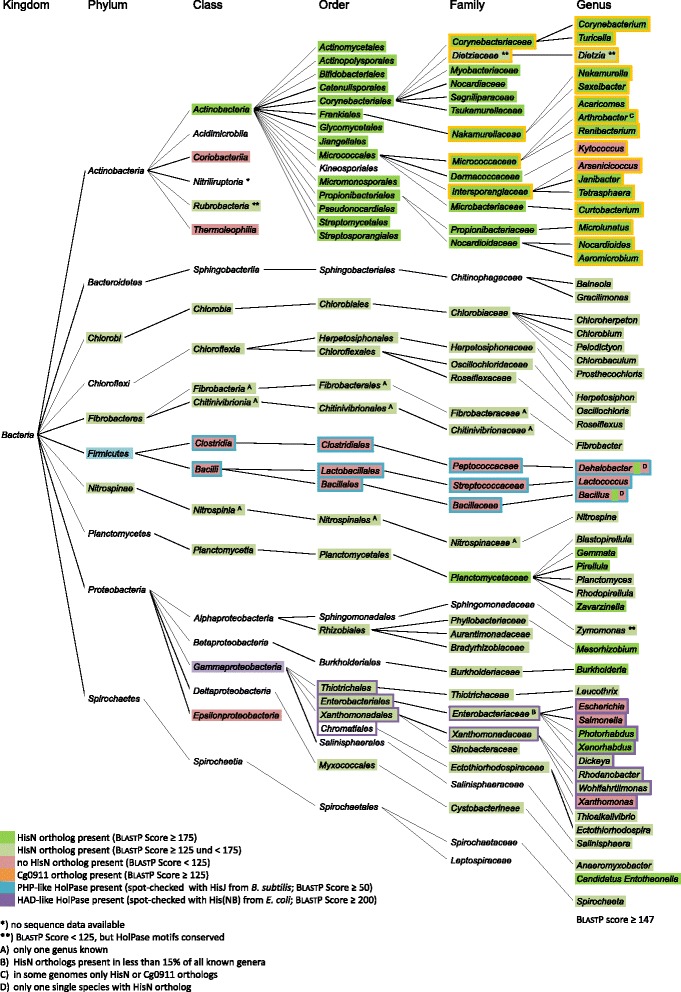
Distribution of putative HisN and Cg0911 orthologs within *Bacteria*. Orthologs were identified by BlastP using the *C. glutamicum* ATCC 13032 HisN and Cg0911 protein sequences as query within NCBI’s non-redundant protein sequences database (nr). A BlastP score ≥ 125 and a sequence coverage ≥ 80% were set as cut-off for identification. All hits were additionally checked for the HolPase motif 5 identified in this study (Fig. 5). Presence of a HisN or Cg0911 ortholog in at least one species is indicated on genus level by a *green* background or an *orange* surrounding, respectively. Since the actinobacterial branch focuses on the distribution of Cg0911 orthologs, display of HisN orthologs is reduced to family level and above. The same colors were applied on the family level and above if HisN or Cg0911 orthologs were identified in at least three or half of the entities of the lower level. A red background indicates that no HisN ortholog was found with the applied cut-off or did not possess the HolPase motif. The expected presence of a PHP-type HolPase [20] is marked with a *blue* surrounding and was spot-checked using the *B. subtilis* HisJ protein sequence via BlastP. The expected presence of a bifunctional HAD-type HolPase [12] is marked with a *purple* surrounding and was spot-checked using the *E. coli* His(NB) protein sequence via BlastP. Other kingdoms were not included in the analysis

The presence of an IMPase-like HolPases has so far only been experimentally proven in *C. glutamicum* and *S. coelicolor* [7, 10], but there is evidence, that this type of HolPase is a general feature of the *Actinobacteria* [5]. According to this assumption, we were able to prove the in vivo HolPase activity of HisN homologs in the actinobacterial genera *Actinoplanes* and *Dietzia* in the present study. However, the recent identification of the IMPase-like HolPase in the plant *Arabidopsis thaliana* [9], the results of our extensive Blast-analysis in order to identify the HolPase motifs, and finally our experimental confirmation of a functional IMPase-like HolPase in the alphaproteobacterium *Z. mobilis* suggests that this type of HolPases might be more widespread than initially assumed. Therefore, we systematically examined the distribution of HisN orthologs within the bacterial kingdom and additionally extended the analysis to Cg0911 orthologs. The results are depicted in Fig. 7.

In this analysis, HisN and Cg0911 homologs were identified by a protein Blast search (BlastP) within NCBI’s non-redundant protein sequences database. A BlastP score ≥ 125 was set as cut-off for identification. This cut-off was chosen, because it was sufficient to reliably distinguish between HisN, Cg0911, and the other IMPase-like homologs ImpA, SuhB, and CysQ in *C. glutamicum*, *M. tuberculosis,* and *S. coelicolor* (data not shown). In addition, all putative HisN orthologs were checked for the presence of the HisN-specific motif 5. A HisN or Cg0911 ortholog was regarded a general feature of the genus if it was present in at least one species belonging to this genus. It was regarded a general feature of the family, if it was present in at least three or half of all genera, and the same criteria applied to the higher taxonomic levels.

According to this analysis, HisN orthologs are a general feature of all orders of the class *Actinobacteria* (BlastP scores > 250 bits). The only exception are the *Kineosporiales*, however this is most likely attributed to the lack of sequence data for some of the genera. Indeed, HisN orthologs are present in *Kineococcus* and *Angustibacter*. HisN orthologs with an unusually low similarity to HisN_*Cg*_ within the class *Actinobacteria* are present within the *Dietziaceae* (BlastP scores ≤ 108 bits). However, despite the overall low sequence similarity we could identify all HolPase motifs (alternative motif 1) in all potential HisN orthologs within *Dietziaceae*. Additionally we proved the in vivo HolPase activity of the HisN homolog from *Dietzia* sp. strain Chol2 (see above). A BlastP query revealed highest sequence similarity of these HisN orthologs to HisN orthologs from different species of the order *Rhizobiales* (max. BlastP score: 210 bits), indicating a recent horizontal gene transfer event.

Although widely distributed within the class *Actinobacteria*, HisN orthologs are not generally present in all classes of the phylum *Actinobacteria*. They can be identified in *Acidimicrobiia* (genus *Ilumatobacter*; max. BlastP score: 179 bits), *Nitriliruptoria* (genus *Nitriliruptor*; max. BlastP score: 174 bits), and *Rubrobacteria* (genus *Rubrobacter,* max. BlastP score: 123 bits), however with considerably lower BlastP scores as compared to the class *Actinobacteria*. In contrast, no HisN orthologs were identified in the classes *Coriobacteriia* and *Thermoleophilia*, despite the availability of complete genome sequences.

IMPase-like HolPases were also identified outside the phylum *Actinobacteria*. The presence of HisN orthologs seems to be a general feature of the phyla *Chlorobi* (green sulfur bacteria), *Fibrobacteres* (cellulose-degrading bacteria), and *Nitrospinae* (marine nitrite oxidizing bacteria). It is also generally found in the class *Chloroflexia* within the phylum *Chloroflexi* (green non-sulfur bacteria) and the class *Planctomycetia* within the phylum *Planctomycetes* (aquatic bacteria). The HisN orthologs from *Planctomycetaceae* exhibit particularly high similarity to HisN_*Cg*_ (BlastP scores: 162–194 bits). HisN orthologs were also identified in some members of the family *Chitinophagaceae*, phylum *Bacteroidetes*, and the order *Spirochaetales*, phylum *Spirochaetes*.

We also identified HisN orthologs within the phylum *Proteobacteria*, with the exception of *Epsilonproteobacteria*. They are generally present in the alphaproteobacterial order *Rhizobiales*, in the deltaproteobacterial order *Myxococcales*, and in the betaproteobacterial family *Burkholderiaceae*. Interestingly, HisN orthologs are also present in many *Gammaproteobacteria*, which are known for the presence of a bifunctional HAD-type HolPase [12]. Five families with a general occurrence of HisN orthologs were observed. In three of them, the *Thiotrichaceae*, *Sinobacteraceae*, and *Ectothiorhodospiracea*, our analysis did not reveal the additional presence of a bifunctional HAD-type HolPase. In the other two, *Enterobacteriaceae* and *Xanthomonadaceae*, a bifunctional His(NB) homolog was identified in all genera with a putative HisN ortholog. However, in the case of *Enterobacteriaceae*, HisN orthologs are present in less than 15% of all genera listed in NCBI taxonomy (11 of 76, including “candidatus” genera). No HisN orthologs were identified in *Escherichia* and *Salmonella*, two genera from *Enterobacteriaceae* with a well characterized HAD-type HolPase [13–15].

Within the phylum *Firmicutes*, HisN orthologs were only identified in two single species, namely *Bacillus* sp. EGD-AK10 (draft; AVPM00000000.1) and *Dehalobacter sp.* FTH1 (draft; AQYY00000000.1). This might be attributed to a recent horizontal gene transfer, according to sequence similarity most likely from an actinobacterial species from the orders *Micrococcales* and *Propionibacteriales*, respectively. Apart from that, no HisN orthologs were identified in any other member of the *Firmicutes*, which is in accordance with the supposed presence of a PHP-type HolPase in this phylum [20], which was positively spot-checked during our analysis using the HisJ protein sequence of *B. subtilis*.

Unlike the HisN orthologs, which are spread throughout various bacterial phyla, Cg0911 orthologs are restricted to a few actinobacterial genera (Fig. 7). They can be generally found in the families *Corynebacteriaceae*, both in *Corynebacterium* and *Turicella*, *Dietziaceae*, *Nakamurellaceae*, *Micrococcaceae*, and *Intersporangiaceae*, but their presence is not restricted to these families. Interestingly, only a Cg0911 but not a HisN ortholog was identified in *Kytococcus* for which at least one complete genome is available (CP001686.1). In those species that contain both orthologs and where genome data was available (finished and draft genomes), we checked for the gene organization. In *C. glutamicum* the *cg0911* gene is directly followed by *hisN* and the two genes form an operon [5]. The same organization in such a *cg0911-hisN* homolog tandem was also found in almost all other available *Corynebacterium* genomes with very few exceptions (data not shown). The *cg0911*-*hisN* homolog tandem was also present in *Turicella otitidis* (draft; CAJZ00000000.1), however not in any other genus possessing both homologs, and can be therefore considered a characteristic of *Corynebacteriaceae* only.

In order to validate the BlastP results, the tool jackhmmer was used to create and refine HMMs based on all functionally validated orthologs of HisN (HisN_*Cg*_, HisN_*Au*_, HisN_*Dz*_, and HisN_*Zm*_) respectively Cg0911_*Cg*_ using a E-value cutoff of 1e-65. In both cases the results corroborate the BlastP results. In addition, the searches with the HisN HMM revealed HisN to be generally present in the alphaproteobacterial orders *Rhodobacterales*, *Rhodospirillales*, *Caulobacterales*, and *Sphingomonadales*. Additionally, HisN orthologs were identified in several species within the phyla *Cyanobacteria* and *Verrucomicrobia*. While below the BlastP cutoff, motif 5 (as well as the others) was found to be present in 1687 out of 1695 sequences identified by the HMM, with the exeption of several sequences from "Candidatus Curtissbacterium" species and a few others (close to the gathering threshold). The HMMs obtained after the final iteration can be found in Additional file 3 (HisN) and Additional file 4 (Cg0911) and can be used for an easy classification of these two groups in the future.

## Discussion

Of the four genes encoding HisN paralogs within the genome of *C. glutamicum* (namely *cg0911*, *impA*, *suhB*, and *cysQ*) only *cg0911* is capable of at least partially complementing the growth defect of the Δ*hisN* strain in l-histidine free medium in vivo. The results with the purified Cg0911 enzyme confirmed its HolPase activity also in vitro. However, the very low catalytic efficiency k_cat_/K_m_ of only 1.98 × 10^1^ s^−1^ M^−1^ indicates, that the HolPase activity of Cg0911 might represent only a side activity of this enzyme. The actual substrate of Cg0911 remains to be elucidated. Known substrates of other IMPase-like proteins are, e.g., inositol-1-P, inositol-2-P, inositol-3-P, glucitol-6-P, glycerol-2-P, 2’-AMP, and l-galactose-1-phosphate [25, 34, 35].

Particular surprising was the fact that *impA* does not encode a protein with HolPase activity. This gene is part of an operon with other l-histidine biosynthesis genes in *C. glutamicum* [5] and a similar gene arrangement is also observed in many other species of different genera including *Corynebacterium*, *Dietzia*, *Gordonia*, *Mycobacterium*, and *Nocardia* (data not shown). The substrate of ImpA remains to be elucidated, but its involvement in mycobacterial cell wall biosynthesis is discussed [36].

The concurrent deletion of *hisN* and all its four paralogs in the *C. glutamicum* quintuple mutant demonstrates two things: Firstly, the absence of all five IMPase-like proteins does not result in complete l-histidine auxotrophy. Thus, at least one additional protein with HolPase activity must exist in *C. glutamicum*. Such an alternative non-IMPase-like HolPase has been identified in *S. coelicolor* [10], however a homolog is not present in *C. glutamicum* (data not shown). A HolPase side activity has been demonstrated for alkaline phosphatases in *S. cerevisiae* [17] and *Neurospora crassa* [37] and has also been reported in *E. coli* [38]. Such a side activity might also be present in *C. glutamicum.* Eventually, one should also consider the possibility of non-enzymatic dephosphorylation of HolP within the cell.

Secondly, the activity of all this five IMPase-like proteins is totally dispensable for growth of *C. glutamicum* on minimal medium under the tested conditions. IMPases are thought to synthesize *myo*-inositol from IMP. *Myo*-inositol is supposed to be mainly used for the synthesis of the coryne- and mycobacterial cell envelope phospholipids phosphatidylinositol and phosphatidylinositol dimannoside [11, 36, 39–41]. The results of the *C. glutamicum* quintuple mutant suggest that the two phospholipids mentioned above are dispensable for *C. glutamicum* or that *myo*-inositol synthesis is carried out by a yet unknown enzyme. The IMPase-like proteins might be additionally involved in other reactions than the synthesis of *myo*-inositol. A high in vitro activity with the substrates sorbitol-6-phosphate, next to IMP itself, has been demonstrated for SuhB from *M. tuberculosis* [25]. CysQ from *M. tuberculosis* exhibits a more than tenfold higher turnover number with the substrate 3′-phosphoadenosine-5′-phosphate (PAP) as compared to IMP and accepts also 3'-phosphoadenoside-5'-phosphosulfate (PAPS) as a substrate [42]. Therefore, it has been suggested that CysQ primarily functions as regulator of the sulfur assimilation in *M. tuberculosis* [42]. Based on high sequence similarity of CysQ_*Mt*_ to CysQ_*Cg*_ (47% identity, 58% similarity) the same enzyme function can be assumed. However, since *C. glutamicum* uses a PAPS independent sulfur assimilation route [43], the function of CysQ_*Cg*_ remains uncertain.

Our results with the purified HisN prove the in vitro HolPase activity of an IMPase-like HolPase (Table 2) for the first time. The catalytic efficiency k_cat_/K_m_ of 4.41 × 10^4^ s^−1^ M^−1^ is four orders of magnitude lower compared to that of the HAD-type HolPase from *E. coli* [13] or *T. onnurineus* [16], but it is in good agreement with the values of several PHP-type HolPases [20]. The k_cat_ of HisN fits very well to the k_cat_ reported for HisG_*Cg*_, the ATP-PR transferase catalyzing the first step of l-histidine biosynthesis in *C. glutamicum* [44], demonstrating an equal catalytic rate for at least two of the nine enzymes involved in l-histidine biosynthesis.

The low k_cat_ of HisN_*Cg*_, especially as compared to that of the HolPase from *E. coli*, might be partially compensated for by the high affinity of the enzyme to its substrate. The K_m_-value of HisN_*Cg*_ for HolP of only roughly 25 μM is the lowest value reported for any HolPase so far. The absence of inhibition of HisN_*Cg*_ by l-histidine and l-histidinol reflects another strategy to deal with the low turnover number. A resistance to inhibition by l-histidine and l-histidinol can also by observed with the PHP-type HolPase of *S. cerevisiae* (K_i_ for l-histidinol: 5–10 mM [18]), which, based on kinetic data of other PHP-type HolPases [20], have a rather low k_cat_. HAD-type HolPases on the other hand exhibit a high k_cat_ and are strongly inhibited by these two substances (e.g. K_i_ for l-histidinol = 52 μM in *S. enterica*) [14]. HisN_*Cg*_ is also not inhibited by P_i_ at least not up to a concentration of 250 μM P_i_ (a concentration that cannot be easily exceeded with the applied HolPase activity assay). However it was demonstrated for the HAD-type HolPase from *S. enterica* that it is not affected by P_i_ up to a concentration of 25 mM P_i_ [14].

Both HisN_*Cg*_ and Cg0911 are strictly dependent on addition of bivalent metal ions to the reaction buffer for HolPase activity, with Mg^2+^ being the preferred ion and reduced activity with Mn^2+^ and Co^2+^ (Fig. 3). This is in accordance with results from a general study on IMPases in *Mycobacterium smegmatis*. This study demonstrated that IMPase activity is maximal with Mg^2+^, is inhibited by Zn^2+^_,_ and about 25% of activity can be obtained with Mn^2+^ [34]. The need for metal ion addition to the in vitro assay has also been observed for HAD-type HolPases that exhibit a binuclear metal cluster in the active center [13, 16]. In contrast to HisN_*Cg*_ and Cg0911, some HAD-type HolPases are also active with Zn^2+^, Cu^2+^ or Ni^2+^ [13, 16]. Interestingly, PHP-type HolPases, although exhibiting a trinuclear metal cluster in the active center, do not rely on addition of external metal ions for activity [20, 23]. This suggests a very tight binding of the metal ions in the metal cluster, resulting in a retention during the protein purification process. In contrast, binding of metal ions in the active site of HAD-type and IMPase-like HolPases seems to be much weaker, resulting in the need of metal ion addition after the protein purification process. This weak binding is supported by the relatively high K_m_ values of Mg^2+^ for HisN and Cg0911 (Table 2) and actinobacterial IMPases in general [34].

The tertiary structure of IMPase-like HolPases, as shown using the example of HisN_*Zm*_, is very similar to that of various mammalian IMPases (data not shown) including the IMPases of *Homo sapiens* [29] and *Bos taurus* [28]. Three Mg^2+^ ions have been identified in the active site of these two intensively investigated proteins coordinated by five highly conserved amino acid residues [24, 28, 29]. These five residues are conserved in HisN_*Zm*_, HisN_*Cg*_, and all analyzed HisN orthologs. It is therefore very likely that the proposed three-metal mechanism for hydrolysis of inositol monophosphate in eukaryotic IMPases might be also employed for hydrolysis of HolP in IMPase-like HolPases. In that case, binding of the second Mg^2+^ ion would be cooperative [28], fitting well to the determined Hill-coefficients of 2.5-3 that indicate a cooperative effect of Mg^2+^ on the HolPase activity of HisN_*Cg*_ and Cg0911.

The optimal pH for the HolPase activity of HisN_*Cg*_ (pH ~7.5) and Cg0911 (pH ~8) reflects the internal pH of *C. glutamicum* (7.5 ± 0.5 [26]). The unusually high optimal pH for HisG activity of around 10 [44] is therefore no general attribute of enzymes involved in l-histidine biosynthesis in *C. glutamicum*. Moreover, the pH optima of Cg0911 and HisN_*Cg*_ differ significantly from the pH optima of HAD- or PHP-type HolPases. HolPases of the PHP-family are most active at pH 8.5-9 [18, 20]. In contrast, HolPases from the HAD-family exhibit their maximal activity at a slightly acidic pH [14, 16].

Overall, there are significant differences in regard to K_m_-values, turnover numbers, inhibition behavior, metal ion preference and pH-optima between HisN_*Cg*_ (as one example of an IMPase-like HolPase) and HolPases of the HAD- or PHP-type. Therefore, IMPase-like HolPases do not only differ in protein sequence and tertiary structure from the two other HolPase families, but their differing enzymatic properties might reflect an adaptation to their host organism.

There are also interesting differences in some aspects of HolPase activity between HisN_*Cg*_ and Cg0911. Next to the very obvious differences in k_cat_ and K_m_, the two enzymes also differ in their pH and temperature profiles. The HolPase activity of Cg0911 does not account significantly for the in vivo l-histidine biosynthesis in *C. glutamicum* under the tested conditions. However, since the catalytic properties of HisN_*Cg*_ and Cg0911 are not identical, their might exist some growth conditions, where the HolPase activity of Cg0911 becomes relevant for the cell, for instance under alkaline stress conditions.

The most interesting observation concerning Cg0911 is the almost five-fold stimulation of HolPase activity by l-histidinol. This kind of a positive feedback by the direct reaction product on enzyme activity has been recently described for the RelA protein of *E. coli,* which synthesizes guanosine tetraphosphate (ppGpp) during the stringent response and is activated by ppGpp via positive allosteric feedback regulation [45].

The analysis of 165 potential bacterial HisN orthologs resulted in the formulation of six sequence motifs (Fig. 5) that can be used for the discrimination of IMPase-like HolPases from other IMPase-like proteins. This is of special interest, since there exist several IMPase-like protein families in bacteria (five are present in *C. glutamicum*) and there are substantial differences in their substrate specificity. The preferred substrates of the ImpA and Cg0911 orthologs still remain to be elucidated. However, at least in *Actinobacteria*, IMP is supposed to be the main substrate of SuhB orthologs [25], PAP and PAPS that of CysQ orthologs [42] and HolP that of HisN orthologs. The different substrate specificities illustrate that each of these IMPase-like proteins is involved in very different metabolic processes and underlines the need to clearly distinguish between the different paralogs. The motifs presented in this study ideally serve this purpose. Moreover, they can also be used for the identification of IMPase-like HolPases in plants.

Within the *Actinobacteria*, it is rather easy to distinguish the different IMPase-like proteins by comparing the amino acid sequences to the corresponding orthologs in *C. glutamicum*. However, since the amino acid sequences of all IMPase-like proteins share a big degree of similarity (approximately 20-30% sequence identity between the different IMPase-like proteins in *C. glutamicum*), it gets harder to classify IMPase-like proteins in more distantly related bacteria based on the overall sequence similarity alone. The motifs described in the present study are therefore of great help in assigning a specific function to a not yet characterized IMPase-like protein. We demonstrated this by proving HolPase activity of HisN orthologs from the genera *Dietzia* and *Zymomonas* that are only moderately similar to HisN_*Cg*_ but possess the expected motifs. We could also demonstrate that a potential HisN_*Cg*_ homolog from *A. utahensis*, with rather high overall sequence similarity but entirely lacking the HolPase motif 5, is not a functional HolPase.

The last result underlines the importance of motif 5 for HolPase activity of HisN orthologs. The detailed examination of the recently solved structure of the IMPase-like HolPase from *Z. mobilis*, suggests that the carboxylic group of the aspartate present in HolPase motif 5 might be involved in substrate recognition. A similar function might be also attributed to at least one of the aromatic amino acids present in motif 5 (Fig. 5: HisN motif 5, position 195). However, since no IMPase-like HolPase has been crystallized in the presence of the substrate HolP or the products l-histidinol and P_i_, any interaction of the conserved residues with the substrates or the products remains speculative.

The application of the here presented motifs for the classification of IMPase-like proteins can help to improve the annotation of IMPase-like proteins. If a suspected HisN ortholog exhibits all HolPase motifs, and especially motif 5, it is very likely that this protein is a HolPase. Vice versa, if the motifs typical of one of the over IMPase-like proteins are identified in a suspected HisN ortholog, it is very likely that this protein is exactly this IMPase-like protein and not a HolPase. Consideration of the overall sequence similarity to HisN_*Cg*_ is not sufficient to identify IMPase-like HolPase. HisN_*Zm*_ is only moderately similar to HisN_*Cg*_ (BlastP score: 101 bits) but possesses all HolPase motifs and we were able to experimentally prove its HolPase activity. In contrast, the SuhB ortholog from *Sediminibacterium salmoneum* has an overall equal degree of similarity to HisN_*Cg*_ (BlastP score: 102 bits) but it is very unlikely a HolPase, since all the HolPase motifs are absent. This demonstrates the benefit of the presented motifs for the functional classification of IMPase-like proteins. The HMMs supplied as Additional file 3 (HisN) and Additional file 4 (Cg0911) should greatly facilitate the correct classification of IMPase-like HolPases.

Our analysis of the distribution of HisN homologs within the bacterial kingdom revealed, that IMPase-like HolPases are not restricted to the phylum *Actinobacteria* (Fig. 7). They appear to be generally present in different phyla like *Chlorobi*, *Fibrobacteres* and *Nitrospinae*. However, HolPase activity of these potential HisN orthologs has not been demonstrated so far. Interestingly, we also detected many putative IMPase-like HolPases in *Gammaproteobacteria*. This is surprising as many gammaproteobacteria are known to possess a bifunctional HAD-type HolPase homologous to the His(NB) protein from *E. coli* [12]. In those gammaproteobacterial families where we did not detect such an additional His(NB) homolog, it is likely that the IMPase-like proteins are the main HolPases. In contrast, the role of the putative HisN orthologs within those gammaproteobacterial families with concurrent occurrence of His(NB) and HisN homologs is less evident. It is possible, that these genera simply possess two different HolPases or that the HisN homologs from these families do not possess HolPase activity. It is also possible that a second, yet unknown activity of HisN orthologs is required in these genera. It has been demonstrated for the IMPase-like HolPase HISN7 from *A. thaliana*, that it additionally catalyzes the dephosphorylation of d-inositol-1-phosphate, d-inositol-3-phosphate, and l-galactose-1-phosphate [35].

Since we used a rather restrictive cut-off for the identification of HisN orthologs (BlastP score ≥ 125 bits with HisN_*Cg*_ as query), Fig. 7 does not give the exhaustive picture of the distribution of IMPase-like HolPases within bacteria. Indeed, the HMM for HisN reveals additional species that possess such an ortholog, however with a BlastP score below the cut-off.

The distribution of Cg0911 orthologs is restricted to the four actinobacterial orders *Corynebacteriales*, *Frankiales*, *Micrococcales*, and *Propionibacteriales* and even within these orders only a few genera possess such a homolog. We identified only a Cg0911 ortholog but no HisN ortholog in *Kytococcus*. It is possible, that Cg0911 is the main HolPase in *Kytococcus*, since presence of other *his* genes indicates the general possibility of l-histidine biosynthesis (data not shown).

The occurrence of the genes encoding HisN and Cg0911 orthologs as a tandem within *Corynebacteriaceae* implies a recent gene duplication event. However, HisN and Cg0911 share only the same degree of similarity as do HisN or Cg0911 with one of the three other paralogs in *C. glutamicum* (Table 1). This indicates that all five paralogs evolved more or less simultaneously and are not a result of a recent duplication event. Although we currently do not know which phosphorylated substance might be the preferred substrate of Cg0911, one could assume that both enzymes are needed in the same context most of the time. However since the genes encoding HisN and Cg0911 orthologs are not clustered in all other genera besides *Corynebacterium* and *Turicella*, this clustering might be simply a result of chance or indicate a special, yet unknown function of Cg0911 orthologs in *Corynebacteriaceae*.

## Conclusions

Here, using the example of the histidinol-phosphate phosphatase HisN from *C. glutamicum,* we present for the first time kinetic data on an IMPase-like HolPases with its natural substrate l-histidinol-phosphate. Based on this data, IMPase-like HolPases show remarkable differences in enzyme properties as compared with HAD- or PHP-type HolPases. Moreover, six sequence motifs have been presented in this study that can be used to reliably differentiate between IMPase-like HolPases and IMPase-like proteins with no such activity (like SuhB or CysQ), with the potential to enhance current and future genome annotations. A phylogenetic analysis reveals that IMPase-like HolPases are not only present in *Actinobacteria* and plants but can be found in further bacterial phyla, including *Chlorobi*, *Fibrobacteres*, and *Proteobacteria*.

## Methods

### Bacterial strains and cultivation conditions

All strains and plasmids used in this study are given in Additional file 1: Table S1 and Table S2, respectively. *Escherichia coli* DH5α MCR [46] was used for general cloning works and plasmid maintenance. *E. coli* ER2566 (New England Biolabs, Ipswich, MA) was used for heterologous gene expression in the context of protein purification. *E. coli* strains were grown in lysogeny broth (LB) Lennox medium. Solid medium contained 1.7% agar. Kanamycin (50 μg ml^−1^) and ampicillin (200 μg ml^−1^) were added where appropriate. *E. coli* strains were incubated at 37 °C if not stated otherwise.

*Corynebacterium glutamicum* ATCC 13032 [47, 48] and all thereof derived mutants (this work) were incubated at 30 °C. Caso broth (Carl Roth, Karlsruhe, Germany) or MM1 minimal medium was used for solid cultivations, supplemented with 1.7% agar. Nalidixic acid (50 μg ml^−1^), kanamycin (25 μg ml^−1^), l-histidine (100 μM), and sucrose (10%) were added where appropriate. The MM1 minimal medium (MMYE medium without yeast extract [49]) was constituted as follows: glucose (20 g l^−1^), (NH_4_)_2_SO_4_ (10 g l^−1^), urea (3 g l^−1^), K_2_HPO_4_ ∙ 3 H_2_O (1.3 g l^−1^), MgSO_4_ ∙ 7 H_2_O (400 mg l^−1^), thiamine (500 μg l^−1^), biotin (50 μg l^−1^), FeSO_4_ ∙ 7 H_2_O (2 mg l^−1^), MnSO_4_ ∙ 7 H_2_O (2 mg l^−1^), NaCl (50 mg l^−1^).

DNA of *Dietzia* sp. strain Chol2 [50], *Zymomonas mobilis* ZM4 [30], and *Actinoplanes utahensis* NRRL 12052 [31] was used for the amplification of *hisN* homologs from these bacteria.

### Recombinant DNA work

A complete list of primers used in this study is given in Additional file 1: Table S3. Phusion high-fidelity DNA polymerase (Thermo Scientific, Dreieich, Germany) was used to amplify DNA fragments for cloning or for sequencing. To improve the amplification of GC-rich DNA from *Dietzia* and *A. utahensis* the GC-buffer provided by the supplier was used and dimethyl sulfoxide (DMSO) was added to the PCR mixture to a final concentration of 8.3%. Plasmids were constructed in two different ways: In the first method, vector and insert were cut with restriction enzymes and joined by a DNA ligase according to standard cloning procedures [51]. Restriction sites needed for cloning of the insert were included in the 5’ overhang of the primers used for the amplification. All restriction and DNA-modifying enzymes were purchased from Thermo Scientific. In the second method, vector and insert were assembled in an isothermal enzymatic reaction by taking advantage of complementary DNA sequences at the end of the DNA fragments as described by Gibson (2011) [52]. For this purpose, vector DNA was linearized in a PCR reaction using the KOD hot start DNA polymerase (Novagen, San Diego, CA). Overlapping DNA-sequences (20–30 bp) were generated by including sequences complementary to the ends of the linearized vector within the 5’ ends of the primers used for amplification of the insert. Specific mutations in an insert were introduced by including the desired mutation in the primers used for amplification of the DNA. All plasmids were constructed and propagated in *E. coli* prior to transfer to *C. glutamicum*.

Competent *E. coli* cells were prepared according to an optimized CaCl_2_ method and transformed with plasmid DNA by applying a heat-pulse [53]. Competent *C. glutamicum* cells were prepared as described previously and transformed with plasmid DNA via electroporation [54] at 2.5 kV, 200 Ω and 25 μF.

### Gene deletion in *C. glutamicum*

Gene deletion in *C. glutamicum* relied on homologues recombination and a double cross-over event using the non-replicating pK18*mobsacB* vector [55] as described before [4]. The genomic regions flanking the deletion of interest were amplified from genomic DNA of the *C. glutamicum* wild type. These fragments (approximately 500 bp) were either fused via the gene splicing by overlap extension (gene SOEing) technique [56, 57] and used for ligation into the pK18*mobsacB* vector or they were directly used for the isothermal enzymatic assembly with the vector (Gibson assembly, [52]). The deletion plasmids constructed in either way were used for transformation of *C. glutamicum*. After selection for the double cross-over event, desired deletions were confirmed by PCR using primers binding to genomic sequences up- and down-stream of the deletion and that were not part of deletion plasmid. All deletion mutants generated in this study are listed in Additional file 1: Table S1.

### *C. glutamicum* Δ*hisN* complementation experiments

The constitutive expression vector pZMP [58] was used for expression of putative HolPase genes in the *C. glutamicum* (plasmids listed in Additional file 1: Table S2). A SD-sequence exactly matching the 3’ end of the 16S-rRNA in *C. glutamicum* was included within the 5’ extension of the primers used for gene amplification. Gene expression from pZMP is under control of the *tac* promoter. Approximately 15 copies of the plasmid are present per *C. glutamicum* cell (unpublished observation). The DNA sequence of the inserts was confirmed by sequencing. Plasmid DNA was isolated from *E. coli* and used for transformation of *C. glutamicum* Δ*hisN*. Successful transformants were identified by selection for the plasmid-encoded kanamycin resistance. Presence of the insert was additionally confirmed by amplification of the insert using vector-specific primers and comparing the size of the PCR product with the expectation.

The *C. glutamicum* Δ*hisN* complementation experiments were conducted on MM1 minimal medium plates either supplemented or unsupplemented with l-histidine. The different mutants were diluted in liquid MM1 medium and drops containing the same amount of cells were applied to the plates. The plates were incubated for several days at 30 °C and pictures were taken in 24 h intervals. The ability of a putative HolPase gene to complement the genomic *hisN* deletion of *C. glutamicum* was assessed by comparing the growth of the expression mutants to the *C. glutamicum* Δ*hisN* or wild type strain.

### Purification of the HisN_*Cg*_ and Cg0911 enzymes

The commercial IMPACT^TM^ system (New England Biolabs, Ipswich, MA) was used for the tag-free purification of HisN and Cg0911 according to the manufacturer’s instructions. In this system, a self-cleavable intein tag is translationally fused to the protein of interest. The tag binds specifically to chitin beads and its self cleavage activity is induced by thiol reagents, allowing the elution of unmodified protein.

The coding DNA sequences (CDS) of *hisN*_*Cg*_ and *cg0911* were amplified from genomic DNA without the start and stop codon (the start codon ATG is present on the vector) and inserted into the pXTB1 (New England Biolabs) vector by isothermal enzymatic assembly. This resulted in the translational fusion of the intein tag to the C-terminus of HisN_*Cg*_ and Cg0911, respectively. The inserts were sequenced to exclude undesired mutations. The plasmids were transferred to *E. coli* ER2566 for heterologous gene expression. Details regarding cultivation, induction of protein expression, cell lyses, protein purification, concentration, and quality control have been described previously [4]. Diverging from this description, column and cleavage buffers used during affinity purification and on column cleavage contained 20 mM TRIS as buffering substance instead of Na_2_HPO_4_. Additionally, a different storage buffer was used (10 mM NaCl, 20 mM TRIS, pH 7.5). The purified proteins were mixed with glycerol (final concentration: 50% (w/v)) and stored at -80 °C.

### HolPase activity assay

HolPase activity can be assayed by the release of inorganic phosphate (P_i_) from the substrate HolP. However, conditions needed for the colorimetric detection of P_i_ are not compatible with the standard conditions for enzymatic catalysis. Therefore, catalysis and P_i_ detection had to be separated.

The reaction conditions for the catalysis step of the HolPase activity were based on the conditions described for the HolPase of *S. enterica* [59]. If not stated otherwise, reactions were conducted in 1.5 ml polypropylene microcentrifuge tubes in a water bath tempered to 30 °C. The standard reaction volume was 100 μl. 80 μl of reaction buffer (100 mM triethanolamine (TEA), pH 7.5 at 22 °C (corresponding to pH 7.35 at 30 °C)) were supplemented with MgSO_4_ resulting in the desired final metal ion concentration (5 mM in standard settings) and were mixed with 10 μl protein solution (freshly diluted with reaction buffer to 0.1 μg μl^−1^ (HisN) or 1 μg μl^−1^ (Cg0911), respectively) and preincubated for 10 min. The reaction was started by addition of 10 μl HolP solution (5 mM; HolP purchased from Paragos, Herdecke, Germany). Samples (30 μl) were taken in appropriate time intervals (e.g. after 1, 2, and 3 min). The reaction was stopped by immediately mixing the withdrawn sample with 30 μl of ice-cold EDTA solution (20 mM). Each enzymatic reaction was performed in at least three replicates.

Some modifications of the catalytic part of the assay were made to access individual enzyme parameters. (1) Temperature optimum: Variation of incubation temperature from 20 °C to 50 °C. The pH of the reaction buffer was adjusted to be 7.35 at every tested temperature. (2) pH-optimum: The pH of the reaction buffer was varied (pH 6–10). The buffer substances (always 100 mM) were changed according to their optimal buffering range (pH 6 [22 °C]/5.98 [30 °C]: 2-(N-morpholino)ethanesulfonic acid (MES); pH 7/6.90: 3-(N-morpholino)propanesulfonic acid (MOPS); pH 8/7.94: 4-(2-hydroxyethyl)-1-piperazineethanesulfonic acid (HEPES); pH 9/8.83: 2-(cyclohexylamino)ethanesulfonic acid (CHES); pH 10/9.83: CHES). (3) Metal ion preference: The metal salts CaCl_2_, CoCl_2_, CuSO_4_, FeSO_4_, MnSO_4_, NiCl_2_, or ZnCl_2_ (5 mM final concentration) were used instead of MgSO_4_. (4) Determination of K_m_ and k_cat_: The HolP concentration was kept constant at 500 μM in the measurements for the determination of the K_m_ value for Mg^2+^. At least nine different Mg^2+^ concentrations were tested ranging from 312.5 μM to 5 mM (HisN_*Cg*_) or from 312.5 μM to 10 mM (Cg0911). The Mg^2+^ concentration was kept constant at 5 mM for the determination of the K_m_ value for HolP. At least seven different HolP concentrations were tested ranging from 17.5 μM to 200 μM (HisN_*Cg*_) or from 125 μM to 1 mM (Cg0911). Every concentration was tested in at least six replicates.

The detection of released P_i_ was based on the complex formation of malachite green with phosphomolybdate under acidic conditions [60]. The method was adopted for measurement in transparent flat bottom 96-well plates in an Infinite M200 plate reader (Tecan, Männedorf, Switzerland). 90 μl H_2_O, 20 μl reagent A (1.75% (w/v) ammonium heptamolybdate tetrahydrate in 6.3 N sulfuric acid), and 10 μl of a P_i_-containing sample were mixed directly in a 96-well plate and incubated at RT for 10 min. Then, 20 μl of reagent C (0.35% (w/v) polyvinyl alcohol (MW ≈ 30 kDa, fully hydrolyzed), 0.035% (w/v) malachite green) were added. The mixture was incubated for additional 45 min at RT. The absorption at 610 nm was measured three times in 3 min intervals. Every P_i_ containing sample was measured in three technical replicates. Samples with known P_i_ concentrations (0.1 - 3.13 mM KH_2_PO_4_) were used to record a calibration curve. Linearity of the measurement was ensured in the range from 0.07 - 0.85 absorption units at 610 nm.

The enzyme activity corresponds to the slope of the linear regression curve through the data points in a plot of released P_i_ against the time. Values for K_m_, k_cat_ and the Hill-coefficients were determined by non-linear curve fitting of the data points in a plot of enzyme activity against the varied substrate concentration to the Hill equation [27] using OriginPro9.0 (function “Hill”; Origin Lab Corporation, Northampton, MA). The molecular masses of HisN_*Cg*_ (27893.16 g mol^−1^) and Cg0911 (30695.27 g mol^−1^) were used for the calculation of the turnover numbers (k_cat_) and were based on the pure amino acid sequence of the monomers.

### Bioinformatics analyses

The online BlastP suite of the National Center for Biotechnology Information (NCBI) was used for the identification of homologous protein sequences. If not stated otherwise, NCBI’s non-redundant (nr) protein sequence database was queried using the default parameters [61].

The online tool Emboss Needle, provided by the European Bioinformatics Institute (EMBL-EBI, [62]), was used in its default settings in order to determine the degree of identity [%] and similarity [%] between two homologous proteins (pairwise sequence alignment). Multiple sequence alignments were conducted with EMBL-EBI’s online tool Clustal Omega in the default settings [62].

The jackhmmr tool was used online on the HMMER webserver, provided by the European Bioinformatics Institute (EMBL-EBI, [63]), to create HMMs for HisN and Cg0911. The UniProt RefProt database was used for an iterative search with either a multiple alignment of all validated HisN orthologs (HisN_*Cg*_, HisN_*Au*_, HisN_*Dz*_, and HisN_*Zm*_) or the single Cg0911 sequence, respectively. Significance E-value cutoffs for sequence andd hit were set to 1e-65 and the the search was iterated until no new hits were found.

The graphical representations of amino acid motifs were created with the online tool WebLogo [64]. The homologous sequences used for these logos were identified by a BlastP search and aligned using Clustal Omega. The multiple alignments were improved manually if appropriate (local rearrangement of gaps or removal of whole sequences from the alignment).

The Ccp4mg molecular-graphics software (version 2.10.5) was used for visualization and analysis of protein structures [65]. Additionally, the online Protein Interactions Calculator (PIC) was used to determine intra- and intermolecular interaction within the protein structures including hydrogen bonds, salt bridges, hydrophobic interactions, as well as aromatic interactions [66].

## Additional files

Additional file 1: Supplementary Figures and Tables. The supplementary figures comprise the SDS-PAGE of purified HisN_*Cg*_ and Cg0911, several alignments of IMPase-like proteins in *C. glutamicum* and other species, and the complementation assay for various *hisN*_*Cg*_ gene mutants. Furthermore, supplementary tables carry the used strains, plasmids and primers. (DOCX 200 kb)

Additional file 2: Data collection for motif analysis. This data collection contains all sequence data of IMPase-like proteins belonging to the group Cg0911, CysQ, HisN, ImpA, and SuhB used for motif analysis. Furthermore, HisN orthologues not used for the motif analysis were listed. (DOCX 120 kb)

Additional file 3: HisN-HMM. Hidden Markov model of 1681 HisN sequences based on an iterative approach using Jackhmmer. (HMM 121 kb)

Additional file 4: Cg0911-HMM. Hidden Markov model of 103 Cg0911 sequences based on an iterative approach using Jackhmmer. (HMM 131 kb)

## Abbreviations

HolP: l-histidinol-phosphate
HolPase: Histidinol-phosphate phosphatase
IMPase: Inositol monophosphatase
