# Supplementary material for: Sequence-based identification of inositol monophosphatase-like histidinol-phosphate phosphatases (HisN) in Corynebacterium glutamicum, Actinobacteria, and beyond
Source: BMC Microbiol. 2017 Jul 18;17:161. doi: 10.1186/s12866-017-1069-4 (PMC5516325; doi:10.1186/s12866-017-1069-4)
Supplement: Supplementary file 1 — Supplementary Figures and Tables. The supplementary figures comprise the SDS-PAGE of purified HisNCg and Cg0911, several alignments of IMPase-like proteins in C. glutamicum and other species, and the complementation assay for various hisN Cg gene mutants. Furthermore, supplementary tables carry the used strains, plasmids and primers. (DOCX 200 kb) [file 12866_2017_1069_MOESM1_ESM.docx]

# Additional File 1 – Supplementary Figures and Tables

| HisN*_Cg_* Cg0911  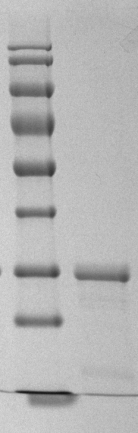 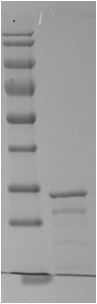  ~35 kDa  ~25 kDa |
| --- |

**Fig. S1**: SDS-PAGE of purified HisN*_Cg_* and Cg0911. The observed molecular weight of Cg0911 in the SDS-gel fits well to the expectation of 30.7 kDa. However, the molecular weight of HisN*_Cg_* appears higher (>30 kDa) than the expected 27.9 kDa. Such an abnormal migration behavior in the SDS-PAGE has also been made with several other IMPase-like proteins [25,67,68]. Identity of the expected proteins was confirmed by MALDI-TOF-MS analysis. The two protein bands below the main Cg0911 band were identified as Cg0911, too, suggesting some unexpected degradation of the protein. Since these bands were weak in comparison to the main band, purity of the Cg0911 fraction was regarded sufficient for the subsequent measurements.

HisN*_Cg_* ------------------------------------------------------------ 0

HisN*_Sc_* ------------------------------------------------------------ 0

HisN*_Au_* ------------------------------------------------------------ 0

HisN*_Dz_* ------------------------------------------------------------ 0

HISN7*_At_* MLAQSHFFSKSFDLIPPQSPALRSANPSLRISSSYSNSRLSFLSSSAIAVPVSRRRFCLT 60

HisN*_Zm_* ------------------------------------------------------------ 0

HisN*_Cg_* -------------------MSKYADDLALALELAELADSITLDRFEASDLEVSSK----- 36

HisN*_Sc_* -------------------MPDYLDDLRLAHVLADAADAATMDRFKALDLKVETK----- 36

HisN*_Au_* -------------------MAGYADDLALAHILADSADAISMARFRALDLKVEEK----- 36

HisN*_Dz_* MTARPDRP-------------LDPDLIEHLDELLAATGRIARRHFHG-ELEALVAGDKGG 46

HISN7*_At_* MASNSKRPNISNESPSELSDTELDRFAAVGNALADASGEVIRKYFRK-KFDIVDK----- 114

HisN*_Zm_* -----------------MSRSAYEDDIRLAHRLADVAADIIRPFFRA-PLTIDLK----- 37

* : *. :

HisN*_Cg_* -PDMTPVSDADLATEEALREKIATARPADSILGEEFGGDVEF---SGRQWIIDPIDGTKN 92

HisN*_Sc_* -PDMTPVSEADKAAEELIRGHLSRARPRDSVHGEEFGV----AGTGPRRWVIDPIDGTKN 91

HisN*_Au_* -PDLTPVSDADTAVEKAIRATLARARPRDGVLGEEFGRTTATAGPGNRYWVIDPIDGTKN 95

HisN*_Dz_* GRGYDPVTEADRDIEALLRAGISSMAPGDRVVGEENGESG-P-ADAPRTWYLDPIDGTKA 104

HISN7*_At_* -DDMSPVTIADQMAEEAMVSIIFQNLPSHAIYGEEKGWRCKE-ESADYVWVLDPIDGTKS 172

HisN*_Zm_* -ADHSPVTKADRGAEQAMRAILEQERPEDGIFGEEMGVSR---PDARRLWVLDPIDGTRA 93

**: ** * : : * . : *** * . * :******:

HisN*_Cg_* YVRGVPVWATLIALLDN----GKPVAGVISAPALARRWWASEGAGAWR-TFNGSSPRKLS 147

HisN*_Sc_* YVRGVPVWATLIALMEAKEGGYQPVVGLVSAPALGRRWWAVEDHGAFT-GRSLTSAHRLH 150

HisN*_Au_* FVRGVPIWATLIALMEG----DTPVAGLVSAPALGRRWWAGRGLGAFA-GRNQHSATRIN 150

HisN*_Dz_* FLTGMAGWGTLVGVVED----GRAVAGWMDQPVLGETFAAVHGRATVRRRSDGPEAFDLH 160

HISN7*_At_* FITGKPVFGTLIALLYK----GKPILGLIDQPILKERWIGMNGRRTKL------NGEDIS 222

HisN*_Zm_* FIGGRASFGTLIALVED----GRPVLGIINQPIHQERWVGVKDLPTSF------NGEVIH 143

:: * :.**:.:: : * :. * . : . . : . :

HisN*_Cg_* VSQVSKLDDASLSFSSLSGWAERDLRDQFVSLTDTTWRLRGYGDFFSYCLVAEGAVDIAA 207

HisN*_Sc_* VSQVSTLSDASFAYSSLSGWEEQGRLDGFLDLTREVWRTRAYGDFWPYMMVAEGSVDLCA 210

HisN*_Au_* VSAVRKLTDASFCYASLNGWAENGRLEQMMDILLGVWRSRAYGDFYGYMLLAEGALDAMA 210

HisN*_Dz_* VSGCDELSEAIMYTTHPSMFGDGELRRRYDDLATRVRLQRFGGDCYAYCMLAAGRVDLVV 220

HISN7*_At_* TRSCPKLSQAYLYTTSPHLFSE-EAEKAYSRVRDKVKVPLYGCDCYAYALLASGFVDLVI 281

HisN*_Zm_* TRSCPALDHALLATTSPWLFEK-EGEVHFDKIRLKCRDTLLGGDCYNYGLLSLGHCDLVV 202

. * .* : : : . : * : * ::: * *

HisN*_Cg_* EPEVSLWDLAPLSILVTEAGGKFTSLAGVDGPHGGDAV----------ATNGILHDETLD 257

HisN*_Sc_* EPELSLWDMAANAIIVTEAGGTFTGLDGRPGPHSGNAA----------ASNGRLHDELLG 260

HisN*_Au_* EPELSLWDMAALIPIVTEAGGKITDLDGRPTADKSSVV----------GTNGLLHESVLT 260

HisN*_Dz_* ESDLKSYDIVALIPIIEAAGGVITGPDGRQPLDGGT---------VVAAAT-----PALA 266

HISN7*_At_* ESGLKPYDFLALVPVIEGAGGTITDWTGKRFLWEASSSAVATSFNVVAAGDSDIHQQALE 341

HisN*_Zm_* EQGLKFYDFAALVPIVEGAGGIMRDWQNRPLNKNSVG-------EVIAAGDHHLIEPALS 255

* :. :*: :: *** : . . *

HisN*_Cg_* RLK--------- 260

HisN*_Sc_* YLNQRY------ 266

HisN*_Au_* ALARRA------ 266

HisN*_Dz_* EQAWAVLGADPR 278

HISN7*_At_* SLEWH------- 346

HisN*_Zm_* AMEL-------- 259

**Fig. S2**: Alignment of the protein sequences of all experimentally confirmed IMPase-like HolPases. *Cg* = *Corynebacterium glutamicum* [7], *Sc* = *Streptomyces coelicolor* [10], *Au* = *Actinoplanes utahensis* (this study), *Dz* = *Dietzia* sp strain Chol2. (this study), *At* = *Arabidobsis thaliana* [9], *Zm* = *Zymomonas mobilis* (this study). Amino acids most characteristic for the HolPase motifs are highlighted (motif 1 = yellow, alternative motif 1 = gray, motif 2 = olive, motif 3 = red, motif 4 = pink, motif 5 = blue, motif 6 = green). Key active site residues involved in binding of metal ions, the substrates phosphate moiety and activation of the water molecule for ester hydrolysis, as derived from the structures of different IMPases [28,29], are underlined.

HisN2*_Au_* MGDVADDLQLAQEAALAGAAEALRHFAALAELPRELKQDGTVVTAADRAVEDRIRAVLTA 60

HisN*_Cg_* MSKYADDLALALELAELADSITLDRFEAS-DLEVSSKPDMTPVSDADLATEEALREKIAT 59

HisN*_Au_* MAGYADDLALAHILADSADAISMARFRAL-DLKVEEKPDLTPVSDADTAVEKAIRATLAR 59

*. **** ** * . : :: :* * :* . * * * *: ** *.*. :* ::

HisN2*_Au_* ARPGDAILGEEHGETLG---TDGRRWIIDPIDGTHLFVEGDDRWLVLIALEDQGEITVGV 117

HisN*_Cg_* ARPADSILGEEFGGDVEF---SGRQWIIDPIDGTKNYVRGVPVWATLIALLDNGKPVAGV 116

HisN*_Au_* ARPRDGVLGEEFGRTTATAGPGNRYWVIDPIDGTKNFVRGVPIWATLIALMEGDTPVAGL 119

*** *.:****.* * *:*******: :*.* * .**** : ..*:

HisN2*_Au_* AAVPAQGRIWWAVRGGGAWEAEVRDARIEDERRIQVA---ETGPARARLGVVPHWGREPY 174

HisN*_Cg_* ISAPALARRWWASEGAGAWRTFNGSSP----RKLSVSQVSKLDDASLSFSSLSGWAERDL 172

HisN*_Au_* VSAPALGRRWWAGRGLGAFAGRNQHSA----TRINVSAVRKLTDASFCYASLNGWAENGR 175

:.** .* *** .* **: : ::.*: : * . : *...

HisN2*_Au_* EAFIAAADERQWPV------QPSLMVARGDLDLSLQTGGKIWDFAATSLIVTEAGGDYRG 228

HisN*_Cg_* RDQFVSLTDTTWRLRGYGDFFSYCLVAEGAVDIAAEPEVSLWDLAPLSILVTEAGGKFTS 232

HisN*_Au_* LEQMMDILLGVWRSRAYGDFYGYMLLAEGALDAMAEPELSLWDMAALIPIVTEAGGKITD 235

: * ::*.* :* : .:**:* :******.

HisN2*_Au_* VDGRTVPGSGASLFGRSAQVNDIALEQLRVR- 259

HisN*_Cg_* LAGVDGPHGGDA-VATNGILHDETLDRLK--- 260

HisN*_Au_* LDGRPTADKSSV-VGTNGLLHESVLTALARRA 266

: * . .. .. :.: .* *

**Fig. S3**: Alignment of the protein sequences of HisN*_Cg_* and the two HisN homologs from Actinoplanes utahensis HisN*_Au_* and HisN2*_Au_*. Only *hisN_Au_* but not *hisN2_Au_* is able to complement a *C. glutamicum* Δ*hisN* strain (this study). Amino acids most characteristic for the HolPase motifs are highlighted (motif 1 = yellow, alternative motif 1 = gray, motif 2: = olive, motif 3 = red, motif 4 = pink, motif 5 = blue, motif 6 = green). Key active site residues involved in binding of metal ions, the substrates phosphate moiety and activation of the water molecule for ester hydrolysis, as derived from the structures of different IMPases [28,29], are underlined.

|  | **cultivation duration** |
| --- | --- |
|  | 24 h 48 h 72 h 144 h |
| WT  Δ*hisN*  Δ*hisN* pZMP::*hisN_Cg_*  Δ*hisN* pZMP::*hisN_Cg_*-D191A  Δ*hisN* pZMP::*hisN_Cg_-*D191E  Δ*hisN* pZMP::*hisN_Cg_-*D191N  Δ*hisN* pZMP::*hisN_Cg_-*D191S | 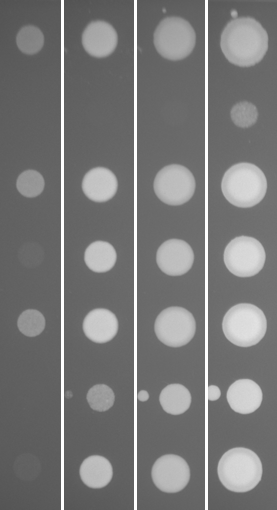 |

**Fig. S4** Comparison of the complementation ability of various *hisN_Cg_* gene variants mutated at the position corresponding to Asp191. 10^4^ cells per spot were placed on MM1 minimal medium plates without l-histidine and incubated at 30 °C.

In total, four *hisN* variants were constructed (*hisN-*D191A, *hisN-*D191E, *hisN-*D191N and *hisN-*D191S). These *hisN* gene variants were cloned into the constitutive pZMP vector and the *C. glutamicum* Δ*hisN* mutant was transformed with the respective plasmids. Growth of the Δ*hisN* pZMP::*hisN*-D191E mutant did not differ from the growth of the Δ*hisN* pZMP::*hisN* mutant or the WT, indicating that the replacement of Asp191 with glutamate has no major impact on the HolPase activity. The replacement of Asp191 with alanine, serine, or asparagine resulted in a slower growth of the corresponding overexpression mutants, however growth was still better compared to the Δ*hisN* mutant, demonstrating some residual HolPase activity. Replacement of Asp191 with asparagine resulted in the slowest growth of the corresponding mutant, suggesting a strong negative effect of the D191N mutation on the HolPase activity. Taken together, the results of the mutational study of the HisN HolPase motif 5 demonstrate an important function of the highly conserved Asp191 for HolPase activity.

**Table S1:** Bacterial strains used in this study. Strains with an additional plasmid (see Supplementary Table 2) are not particularly listed.

| strain | characteristic traits | references |
| --- | --- | --- |
| ***Corynebacterium glutamicum*** |  |  |
| ATCC 13032 | wild type, Nx^r^ | (1), (2) |
| All strains used in this study were derived from strain ATCC 13032 and possess the same traits. | | |
| Δ*hisN* | *ΔhisN* (*cg0910*) | this study |
| Δ*impA* | Δ*impA* (*cg2298*) | this study |
| Δ*suhB* | Δ*suhB* (*cg2090*) | this study |
| Δ*cysQ* | Δ*cysQ* (*cg0967*) | this study |
| Δ*cg0911* | Δ*cg0911* | this study |
| Δ*hisN* Δ*cg0911* | *ΔhisN*(*cg0910*), Δ*cg0911* | this study |
| Δ*hisN* Δ*cg0911* Δ*impA* Δ*suhB* ΔcysQ | *ΔhisN*(*cg0910*), *Δcg0911*, Δ*impA* (*cg2298*), Δ*suhB* (*cg2090*), Δ*cysQ* (*cg0967*) | this study |
| Δ*cg1700* | Δ*cg1700* | this study |
| Δ*hisN* Δc*g1700* | *ΔhisN* (*cg0910*), Δ*cg1700* | this study |
|  |  |  |
| ***Escherichia coli*** |  |  |
| DH5αMCR | F^-^, *endA1*, *glnV44*, *thi*-*1*, *recA1*, *relA1*, *gyrA96*, *deoR*, Φ80‘*lacZ*ΔM15, Δ(*lacZYA*-*argF*)U169, Δ(*mrr*-*hsdRMS*-*mcrBC*), λ–, *mcrA* | (3) |
| ER2566 | F-, λ-, *fhuA2*, *lon*^-^, *ompT* *lacZ*::T7, *gene1*, *gal*, *sulA11*, Δ(*mcrC*-*mrr*)114::IS10, R(*mcr*-*73::miniTn10*-Tet^S^)2, R(*zgb*-*210::Tn10*-Tet^S^), *endA1*, *dcm*^-^ | (4) |

Nx^r^ = resistant to naladixic acid

(1) American Type Culture Collection, Rockville, MD

(2) Kinoshita *et al.,* 1958 [47]

(3) Grant *et al.*, 1990 [46]

(4) New England Biolabs, Ipswich, MA

**Table S2:** List of Plasmids used in this study.

| plasmid | description | references |
| --- | --- | --- |
| pK18*mobsacB* | Vector for gene deletions in *C. glutamicum*, Km^r^ | (1) |
| pK18*mobsacB*::Δ*hisN* | plasmid for deletion of *hisN* | this study |
| pK18*mobsacB*::Δ*impA* | plasmid for deletion of *impA* | this study |
| pK18*mobsacB*::Δ*suhB* | plasmid for deletion of *suhB* | this study |
| pK18*mobsacB*::Δ*cysQ* | plasmid for deletion of *cysQ* | this study |
| pK18*mobsacB*::Δ*cg0911* | plasmid for deletion of *cg0911* | this study |
| pK18*mobsacB*::Δ*cg0911*-*hisN* | plasmid for concurrent deletion of *cg0911* and *hisN* | this study |
| pK18*mobsacB*::Δ*cg1700* | plasmid for deletion of *cg1700* | this study |
|  |  |  |
| pZMP | size reduced derivative of pZ8.1 | (2) |
| pZMP::hisN*_Cg_* | *hisN_Cg_* expression plasmid | this study |
| pZMP::*hisN_Cg_*-D191A | *hisN_Cg_*-D191A expression plasmid | this study |
| pZMP::*hisN_Cg_-*D191E | *hisN_Cg_*-D191E expression plasmid | this study |
| pZMP::*hisN_Cg_-*D191N | *hisN_Cg_*-D191N expression plasmid | this study |
| pZMP::*hisN_Cg_-*D191S | *hisN_Cg_*-D191S expression plasmid | this study |
| pZMP::*cg0911* | *cg0911* expression plasmid | this study |
| pZMP::*cg0911* -F216L | *cg0911*-F216L expression plasmid | this study |
| pZMP::*cg0911-*F216V | *cg0911*-F216V expression plasmid | this study |
| pZMP::*cg0911-*F216Y | *cg0911*-F216Y expression plasmid | this study |
| pZMP::impA | *impA_Cg_* expression plasmid | this study |
| pZMP::suhB | *suhB_Cg_* expression plasmid | this study |
| pZMP::suhB^HPM^ | *suhB*^HPM^ expression plasmid (replacement of SuhB*_Cg_* motif 5 with HisN*_Cg_* motif 5) | this study |
| pZMP::cysQ | *cysQ_Cg_* expression plasmid | this study |
| pZMP::*hisN_Au_* | plasmid for expression of HisN from *A. utahensis* NRRL 12052 (WP_043524633.1) | this study |
| pZMP::*hisN2_Au_* | plasmid for expression of HisN2 from *A. utahensis* NRRL 12052 (WP_043533694.1) | this study |
| pZMP::*hisN_Dz_* | plasmid for expression of HisN from *Dietzia* sp. strain Chol2 | this study |
| pZMP::*hisN_Zm_* | plasmid for expression of HisN from *Z. mobilis* ZM4 (WP_011241290.1) | this study |
|  |  |  |
| pTXB1 | expression vector for protein purification using the IMPACT^TM^ system, C-terminal fusion of protein of interest to the intein tag, T7 promoter, IPTG inducible, Amp^r^ | (3) |
| pTXB1::*hisN_Cg_* | expression plasmid for purification of HisN*_Cg_* | this study |
| pTXB1::*cg0911* | expression plasmid for purification of Cg0911 | this study |

Km^r^ = kanamycin resistance encoded; Amp^r^ = ampicillin resistance encoded

(1) Schäfer *et al.*, 1994 [55]

(2) Walter *et al.*, 2015 [58]

(3) New England Biolabs, Ipswich, MA

**Table S3**: Primers used in this study.

| intended use | primer name | sequence (5‘->3‘), sequences in front of the hyphen represent 5‘ overhangs | characteristics (bold; underlined; italic) | | |  |  |
| --- | --- | --- | --- | --- | --- | --- | --- |
| **deletions (*pK18mobsacB* system)** | | |  | | |  |  |
| deletion of *hisN* (672 bp) | *hisN*-d1 | ACGTGC**GGATCC**-AGTGTCGTCGGTTGCTGTAG | ***Bam*HI** | | |  |  |
|  | *hisN*-d2 | CTTGCCGAACTTGCCGATTC-AGTAGCCACCAACGGCATCC |  | | |  |  |
|  | *hisN*-d3 | GGATGCCGTTGGTGGCTACT-GAATCGGCAAGTTCGGCAAG |  | | |  |  |
|  | *hisN*-d4 | ACGTAT**CTGCAG**-TGCAAGCCTCGAAGACATGA | ***Pst*I** | | |  |  |
| testing primers *hisN* deletion | *hisN*-del-t1 | GTCGAGGAGTGGTGCACATA |  | | |  |  |
|  | *hisN*-del-t2 | GACGGCATGCACAATGGAAG |  | | |  |  |
| deletion of *cg0911* (862 bp) | *cg0911*-d1 | A**GGTCTC**GTCGAG-GCCAGGTGGTATCAGTTAGG | ***Eco*31I,** *Sal*I overhang * | | |  |  |
|  | *cg0911*-d2 | CTTGGCCGACGGCATGCACA-GCGCACTAGACTAACAACAC |  | | |  |  |
|  | *cg0911*-d3 | GTGTTGTTAGTCTAGTGCGC-TGTGCATGCCGTCGGCCAAG |  | | |  |  |
|  | *cg0911*-d4 | CTATGC**ATGCAT**CAAGCTTGGTACCGAG-AGTCCGCAGCATCGACACCA | ***Nsi*I** (vector: *Pst*I) | | |  |  |
| testing primers *cg0911* deletion | *hisN*-del-t1 | GTCGAGGAGTGGTGCACATA |  | | |  |  |
|  | *cg0911*-del-t2 | TAGTCGCCGTGGACCACGAA |  | | |  |  |
| deletion of *cg0911+hisN* (1637 bp) | *cg0911-N*-d1 | TAGCTC**GGATCC**GAG-AGTCCGCAGCATCGACACCA | ***Bam*HI** | | |  |  |
|  | *cg0911-N* -d2 | AACGATCCAGCGTCTCATCG-TGTGCATGCCGTCGGCCAAG |  | | |  |  |
|  | *cg0911-N* -d3 | CTTGGCCGACGGCATGCACA-CGATGAGACGCTGGATCGTT |  | | |  |  |
|  | *cg0911-N* -d4 | TAGACT**CTCGAG**-AGCTCTTCCAGCGTTCCATT | ***Xho*I** (vector: *Sal*I) | | |  |  |
| testing primers *cg0911+hisN* deletion | *hisN*-del-t1 | GTCGAGGAGTGGTGCACATA |  | | |  |  |
|  | *cg0911*-del-t2 | TAGTCGCCGTGGACCACGAA |  | | |  |  |
| deletion of *impA* (829 bp) | *impA*-d1 | GATCTA**GAATTC**-TTCCAGCAGCTCTAGGTCAA | ***Eco*RI** | | |  |  |
|  | *impA* -d2 | ATGGGCGTGGCAATTCGAGT |  | | |  |  |
|  | *impA* -d3 | ACTCGAATTGCCACGCCCAT- GTATTAACCGAGCTTTTCTACTGC |  | | |  |  |
|  | *impA* -d4 | GATCTA**AGATCT**-GGTATCCGTGATGATGAGTC | ***Bgl*II** (vector: *Bam*HI) | | |  |  |
| testing primers *impA* deletion | *impA* -del-t1 | GCGATTACAGGAATGGATAC |  | | |  |  |
|  | *impA* -del-t2 | AGATGATGGCGGAAATTGTC |  | | |  |  |
| deletion of *suhB* (871 bp) | *suhB*-d1 | GATCTA**CAATTG**-GAACCCAAGCTCTCGAACAA | ***Mun*I** (vector: *Eco*RI) | | |  |  |
|  | *suhB* -d2 | CCTCTGACCTGGCAGTATGA |  | | |  |  |
|  | *suhB* -d3 | TCATACTGCCAGGTCAGAGG-CCATAAGACCATCATCGTTG |  | | |  |  |
|  | *suhB* -d4 | GATCTA**AGATCT**-TGGCCATCCACAAGGAATGC | ***Bgl*II** (vector: *Bam*HI) | | |  |  |
| testing primers *suhB* deletion | *suhB* -del-t1 | CTACCGCCTCTGGTTGCTTT |  | | |  |  |
|  | *suhB* -del-t2 | GATGCTGCTGCAAGGTGTTC |  | | |  |  |
| deletion of *cysQ* (374 bp) | *cysQ*-d1 | GATCTA**GAATTC**-AGCTCGCCGTCCTCATCTGC | ***Eco*RI** | | |  |  |
|  | *cysQ* -d2 | GATTGCTCGAGTGTTGGAGC-TACGACGCCTACATCCACGC |  | | |  |  |
|  | *cysQ* -d3 | GCGTGGATGTAGGCGTCGTA-GCTCCAACACTCGAGCAATC |  | | |  |  |
|  | *cysQ* -d4 | ACGTGC**GGATCC**-CAACGGTGCACGCAGATTAT | ***Bam*HI** | | |  |  |
| testing primers *cysQ* deletion | *cysQ* -del-t1 | GCCACTGGACACCATAAACA |  | | |  |  |
|  | *cysQ* -del-t2 | GAACCGCCACAGTGATATTG |  | | |  |  |
| linearization of pK18*mobsacB* for assembly cloning | pK18msB-gi-fw | AAGCGGAACACGTAGAAAGCCAGTCCGCAG |  | | |  |  |
|  | pK18msB-gi-rv | GGTGCCTAATGAGTGAGCTAACTCACATTA |  | | |  |  |
| deletion of *cg1700* (612 bp) | *cg1700*-gi-d1 | *TAGCTCACTCATTAGGCACC*-CGTTGAGGGATTTGGAATCT | *complementary to vector* | | |  |  |
|  | *cg1700* -gi-d2 | GACATGGACG-AGATGGTCACTGCCTGGTAC |  | | |  |  |
|  | *cg1700* -gi-d3 | GTGACCATCT-CGTCCATGTCCCAGAAAATC |  | | |  |  |
|  | *cg1700* -gi-d4 | *GCTTTCTACGTGTTCCGCTT*-GGTCACCATGGGTAATCCTA | *complementary to vector* | | |  |  |
| testing primers *cg1700* deletion | *cg1700* -del-t1 | GCGAAGGAAGAAGAATTCAA |  | | |  |  |
|  | *cg1700* -del-t2 | GTGCATCGCGGATTTCATTC |  | | |  |  |
|  |  |  |  | | |  |  |
| **plasmid-based overexpression (pZMP vector; assembly cloning)** | | | | | |  |  |
| linearization of pZMP for assembly cloning | pZMP-gi-fw | GGTTGTCCTCCTTTC-AATTCTGTTTCCTGTGTGAA | SD sequence (rev.-compl.) | | | | |
|  | pZMP-gi-rv | GATCCGTCGACCTGCAGCCA |  | | |  |  |
| overexpression of *hisN_Cg_* | *hisN*-ov-gi1 | ACAGGAAACAGAATTGAAAGGAGGACAACC-ATGAGCAAATATGCAGACGA | SD sequence | | |  |  |
|  | *hisN*-ov-gi2 | ACAGAAGCTTGGCTGCAGGTCGACGGATCG-CTATTTTAAACGATCCAGCG |  | | |  |  |
| primers for the introduction of point mutations in *hisN_Cg_* | *hisN* -D191A-1 | ACGAGGCAGTAGGAGAAGAA**CGC**-GCCGTAGCCGCGGAGTCGCC | **codon** for D191A mutation (rev.-compl.) | | | | |
|  | *hisN* -D191A-2 | GGCGACTCCGCGGCTACGGC**GCG**-TTCTTCTCCTACTGCCTCGT | **codon** for D191A mutation (forw.) | | | | |
|  | *hisN* -D191E-1 | ACGAGGCAGTAGGAGAAGAA**TTC**-GCCGTAGCCGCGGAGTCGCC | **codon** for D191E mutation (rev.-compl.) | | | | |
|  | *hisN* -D191E-2 | GGCGACTCCGCGGCTACGGC**GAA**-TTCTTCTCCTACTGCCTCGT | **codon** for D191E mutation (forw.) | | | | |
|  | *hisN* -D191N-1 | ACGAGGCAGTAGGAGAAGAA**GTT**-GCCGTAGCCGCGGAGTCGCC | **codon** for D191N mutation (rev.-compl.) | | | | |
|  | *hisN* -D191N-2 | GGCGACTCCGCGGCTACGGC**AAC**-TTCTTCTCCTACTGCCTCGT | **codon** for D191N mutation ( (forw.) | | | | |
|  | *hisN* -D191S-1 | ACGAGGCAGTAGGAGAAGAA**TGA**-GCCGTAGCCGCGGAGTCGCC | **codon** for D191S mutation (rev.-compl.) | | | | |
|  | *hisN* -D191S-2 | GGCGACTCCGCGGCTACGGC**TCA**-TTCTTCTCCTACTGCCTCGT | **codon** for D191S mutation (forw.) | | | | |
| overexpression of *cg0911* | *cg0911*-ov-gi1 | ACAGGAAACAGAATTGAAAGGAGGACAACC-ATGACTAATCCAGAGCAGAC | SD sequence | | |  |  |
|  | *cg0911*-ov-gi2 | ACAGAAGCTTGGCTGCAGGTCGACGGATCG-TTAGTCTAGTGCGCTTAAAG |  | | |  |  |
| primers for the introduction of point mutations in *cg0911* | *cg0911*-F216L-1 | AAATCGATGGAGCCGGCGCC**TAG**-CATGCGCAGCGTTGCAGGGT | **codon** for F216L mutation (rev.-compl.) | | | | |
|  | *cg0911*-F216L-2 | ACCCTGCAACGCTGCGCATG**CTA**-GGCGCCGGCTCCATCGATTT | **codon** for F216L mutation (forw.) | | | | |
|  | *cg0911*-F216V-1 | AAATCGATGGAGCCGGCGCC**TAC**-CATGCGCAGCGTTGCAGGGT | **codon** for F216V mutation (rev.-compl.) | | | | |
|  | *cg0911*-F216V-2 | ACCCTGCAACGCTGCGCATG**GTA**-GGCGCCGGCTCCATCGATTT | **codon** for F216V mutation (forw.) | | | | |
|  | *cg0911*-F216Y-1 | AAATCGATGGAGCCGGCGCC**ATA**-CATGCGCAGCGTTGCAGGGT | **codon** for F216Y mutation (rev.-compl.) | | | | |
|  | *cg0911*-F216Y-2 | ACCCTGCAACGCTGCGCATG**TAT**-GGCGCCGGCTCCATCGATTT | | **codon** for F216Y mutation (forw.) | | |  |
| overexpression of *impA* | *impA*-ov-gi1 | ACAGGAAACAGAATTGAAAGGAGGACAACC-ATGGATGCTCGTGGGATGTT | | SD sequence | | |  |
|  | *impA*-ov-gi2 | ACAGAAGCTTGGCTGCAGGTCGACGGATCG-TTACTTGTACTCCTCATTTAACG | |  | | |  |
| overexpression of *suhB* | *suhB*-ov-gi1 | ACAGGAAACAGAATTGAAAGGAGGACAACC-ATGGAACAACAATCGTTTAA | | SD sequence | | |  |
|  | *suhB*-ov-gi2 | ACAGAAGCTTGGCTGCAGGTCGACGGATCG-TTAACGGTCTAAAGCCTTAA | |  | | |  |
| overexpression of *cysQ* | *cysQ*-ov-gi1 | ACAGGAAACAGAATTGAAAGGAGGACAACC-ATGACTGCTCAGATTGATGA | | SD sequence | | |  |
|  | *cysQ*-ov-gi2 | ACAGAAGCTTGGCTGCAGGTCGACGGATCG-TTAGTAAGTTCCATTCTCCT |  | | |  |  |
| overexpression of *cg1700* | cg1700-ov-gi1 | ACAGGAAACAGAATTGAAAGGAGGACAACC-ATGATTAAGGCGATTTTCTG | SD sequence | | |  |  |
|  | cg1700-ov-gi2 | ACAGAAGCTTGGCTGCAGGTCGACGGATCG-TTATTTTGCGACACCTGCCG |  | | |  |  |
| overexpression of *hisN from A. utahensis NRRL 12052* | *hisN_Au_*-ov-gi1 | GAATTGAAAGGAGGACAACC-ATGGCCGGATATGCCGACGA | SD sequence | | |  |  |
|  | *hisN_Au_*-ov-gi2 | TGGCTGCAGGTCGACGGATC-TCAGGCGCGGCGAGCCAGCG |  | | |  |  |
| overexpression of *hisN2 from A. utahensis NRRL 12052* | *hisN2_Au_*-ov-gi1 | GAATTGAAAGGAGGACAACC-ATGGGTGACGTGGCAGACGA | SD sequence | | |  |  |
|  | *hisN2_Au_*-ov-gi2 | TGGCTGCAGGTCGACGGATC-TCACCGAACCCTGAGCTGCT |  | | |  |  |
| overexpression of *hisN from Dietzia sp. strain Chol2* | *hisN_Dz_*-ov-gi1 | GAATTGAAAGGAGGACAACC-ATGACTGCGAGGCCCGACCG | SD sequence | | |  |  |
|  | *hisN_Dz_*-ov-gi2 | TGGCTGCAGGTCGACGGATC-GTCGGTCACCGCGGGTCCGC |  | | |  |  |
| overexpression of *hisN from Z. mobilis* | *hisN_Zm_*-ov-gi1 | GAATTGAAAGGAGGACAACC-ATGTCCCGAAGCGCTTATGA | SD sequence | | |  |  |
|  | *hisN_Zm_*-ov-gi2 | TGGCTGCAGGTCGACGGATC-TTATAATTCCATGGCAGACA |  | | |  |  |
|  |  |  |  | | |  |  |
| **protein expression (pTX1 vector; IMPACT^TM^ system)** | | | | |  |  |  |
| linearization of pTXB1 for assembly cloning | ptXB1-gi-fw | TGCATCACGGGAGATGCACT |  | | |  |  |
|  | ptXB1-gi-rv | CATATGTATATCTCCTTCTTAAAGTTAAAC |  | | |  |  |
| HisN*_Cg_* protein expression | *hisN*-imp-gi1 | GTTTAACTTTAAGAAGGAGATATACATATG-AGCAAATATGCAGACGATTT |  | | |  |  |
|  | *hisN*-imp-gi2 | TAGGGCAACTAGTGCATCTCCCGTGATGCA-TTTTAAACGATCCAGCGTCT |  | | |  |  |
| Cg0911 protein expression | *cg0911*-imp-gi1 | GTTTAACTTTAAGAAGGAGATATACATATG-ACTAATCCAGAGCAGACACA |  | | |  |  |
|  | *cg0911*-imp-gi2 | TAGGGCAACTAGTGCATCTCCCGTGATGCA-GTCTAGTGCGCTTAAAGTTT |  | | |  |  |

* is generated after cleavage with *Eco*31I

**Additional References**

67. Chen L, Roberts MF. Overexpression, purification, and analysis of complementation behavior of *E. coli* SuhB protein: Comparison with bacterial and archaeal inositol monophosphatases. Biochemistry. 2000;39:4145-53.

68. Gu X, Chen M, Shen H, Jiang X, Huang Y, Wang H. Rv2131c gene product: an unconventional enzyme that is both inositol monophosphatase and fructose-1,6-bisphosphatase. Biochem Biophys Res Commun. 2006;339:897-904.
