# Supplementary material for: Sequence-based identification of inositol monophosphatase-like histidinol-phosphate phosphatases (HisN) in Corynebacterium glutamicum, Actinobacteria, and beyond
Source: BMC Microbiol. 2017 Jul 18;17:161. doi: 10.1186/s12866-017-1069-4 (PMC5516325; doi:10.1186/s12866-017-1069-4)
Supplement: Supplementary file 2 — Data collection for motif analysis. This data collection contains all sequence data of IMPase-like proteins belonging to the group Cg0911, CysQ, HisN, ImpA, and SuhB used for motif analysis. Furthermore, HisN orthologues not used for the motif analysis were listed. (DOCX 120 kb) [file 12866_2017_1069_MOESM2_ESM.docx]

**Additional File 2 – Data Collection for Motif Analysis**

**Cg0911 orthologs used for motif identification**

>Cg0911_Corynebacterium_glutamicum |500226826|ref|WP_011896919.1| fructose-1 6-bisphosphatase []

MTNPEQTHPAASLEDMIKTITKTFVIAHDQDSDEHLAQALVYNAGRLAWRMRENGVDTDYKTSVSDVVTD

ADRAAEAFVAGVLEALRPEDGVLGEEGADRASKSGKTWVIDPVDGTYNFTQGSDYWCSALALVEGDPSAP

SRVLFGAVHRPAMGYTWFGGPGIRTTLDGKELDLLVDAPLNQISLATYIHPSRIAEPDIQKAWMSVATHP

ATLRMFGAGSIDLANIADGSIGAWVQHSVADWDWLPGRALIEGVGGACIKVTAGGVEWSVAGNAEAVSEI

SETLSALD

>Cg0911_Corynebacterium_diphtheriae |489947283|ref|WP_003850590.1| fructose 1,6-bisphosphatase [Corynebacterium diphtheriae]

MENSSDPRTQSAATASLSEMIATITKTFIIAHVDDSDEHLAQALVYNAGRLAWRMREQGVHTDYKTSLSD

VVTDADRAAEEFVSGVLTTLRPEDGIVGEEGATRNSTSGRTWVIDPVDGTYNFVNGSDFWCSALALIEGE

PEDPTRIIFGAVHRPAMGYTWFGGPEIPTTRDGQPVAKLENKSTELINFATYLNPPYLQQPEIVTRWLTI

AQAFLSWRMMGSGSVELANVADGTIGAYVQHSVKDWDWLPGRALVEGAGGKCIKVESGGVTWSIASNPQA

TDEVAAMLSR

>Cg0911_Corynebacterium_resistens |503653685|ref|WP_013887761.1| fructose-1 6-bisphosphatase []

MASDNSPIPQDMLDAIIKTFMVAHVDDSDEHLAQALVFNAGRLAWRMRESGLTTETKTSVSDVVTAADRA

AEKFIADVLRAFRPEDGLLGEEGTQQESTSGRTWVIDPVDGTYNFTIGSDYWCSALALVEGSPDNPDRVI

FGAVHRPAMGQTWFGGPDIPTTLDGKPVQASTATTNETCLGGYLHPTDFANKDVATAWQSVVQEFATIRM

FGSASVDLGSVAAGQLGCWMQRSVKSWDWLPGRALVEGAGGKAVQIEAQGTTWSIAGSTAAVEAASKKIQ

NP

>Cg0911_Corynebacterium_capitovis |516648721|ref|WP_018017710.1| hypothetical protein []

MLGTMTELQHFVEAHQRSDDAELAAALVRHAGALALQMRTTGLDTDIKTSISDVVTDADRTAEAFVASAL

AILRPDDGLLGEEGASRESRSGRTWVVDPVDGTYNFSRGLDYFCSALAYVEGEAVLIGAVNRPSTDTTWL

AHRGVLTRDGQPVPPLADAPLSEVALATYLHPTSIRREHVRAAWQEVAQDAATVRMLGAGSLDLAAVATG

ELGGWLQHSVADWDWFPGKALVEAAGGEAIKVDAGGRTWCVAGNRLFVADVTARLGKHVSLR

>Cg0911_Corynebacterium_freiburgense |652648070|ref|WP_027011428.1| fructose 1,6-bisphosphatase []

MQNSQNLEEMIGTITKTFVIAHEADTDEHLAQALVFNAGRLAWRMRETGITTQRKTNISDVVTEADHAAE

QFISGALLALRPNDGVLGEEGASRPAETGRIWVIDPIDGTYNFASGSDYFCSALALQDNGELRFGAVHRP

TMGYTWFGGKHIPTTRDSQKLAPLQDTPLSEVSLGSYLHPSYMNSLSATWINVAKHAATVRMLGAGSIDL

ASVADGTLGAWMQHSAPAWDWLPGKALIEGAGGRCETADAGGVTWHIAGNTRAVAEILDALH

>Cg0911_Turicella_otitidis |490739610|ref|WP_004601918.1| fructose-1 6-bisphosphatase []

METMSDTQDSPTLPGGHSPLELADAMAKTQLAAHGDRDDEGLARDLVLNAGRLAWRLREIGLSVDHKTGL

TDVVTDADRAAEHFVSRVLSEARPDDGIVGEEGAAAKSSSGRTWVVDPVDGTFNFTRGSDYWCSALALVE

GDPDNPERIILGAVHRTAMGYTWLGGPEIPTTRDGKRLNAPADVPLEEAAVATYLDPKFLGDTPQRRGYE

AVVARAATQRMLGAGSVDLADTASGGHDLWLQHTVTSWDWLPGRALVEGAGGTTRTVEAAGVSWRLAGRP

RAVEDAARVLEWVL

>Cg0911_Dietzia_cinnamea |494907836|ref|WP_007633877.1| fructose 1,6-bisphosphatase []

MSIDTGHPRPLPDEHTERLSDVDLAARLVVDAGTLARRLREDGLDVRRKTSVSDVVTEADDAAERHITTL

LATHRPDDGVLGEEGTLVAGGHDRRWVVDPVDGTYNFVSGSDYWCSAVALSGPDDYLLGAVHRPATGETF

VGGSETPTRRNGVPVTPLSPGDLAAGSLATYLHPPFLTDPDLAEPFHRVSRGAATIRMLGSGSVDLASVA

CGIHAVWCQHSCPEWDWLPGRALVEGAGGVCATVEVNGYDWFVAGRPGAVDQAVALLRG

>Cg0911_Dietzia_alimentaria |498227810|ref|WP_010541966.1| fructose-1 6-bisphosphatase []

MSIDTRQAGLRGGAAAEMNDADLAAHLVVDAGNLARRLREDGLSVRHKTSVSDVVTQADKAAERHITSVL

AAHRPADGVLGEEGTLVNGDRERRWVVDPVDGTYNFVSGSDYWCSAVALAGPDDRLVGAVHRPVTGETFV

GGSGVPTRRNGVPVPRLEPGDLADGSLATYLHPPFFDDPDLADPFHRVSRGAATIRMFGSGSVDLASVAC

GIHTVWCQHSCPEWDWLPGRALVEGAGGVCATVEVNGYDWFVAGRPGAVEQAVGLLLG

>Cg0911_Dietzia_sp._UCD-THP |516447666|ref|WP_017836578.1| fructose 1,6-bisphosphatase [Dietzia sp. UCD-THP]

MPPETRHRRTASDEATRAMTDVDLAARLVVDAGSLARRLRDDGLDVHHKTSVSDVVTEADRAAERQITAL

LAAHRPEDGILGEEGTLVPGRHERRWVVDPVDGTYNFVSGSDYWCSAVAVTGPDDYLLGAVHRPVTGDTF

VGGSEVPTRRNGVPVTRLTPGDLAAGSLATYLHPPFFADPALADPFHRVSRGAATIRMLGSGSVDLAAVA

CGVHSVWCQHSCPEWDWLPGRALVEGAGGLCATVEVNGYDWFVAGRPGAVEDAVTLLKG

>Cg0911_Rhodococcus_sp._R04 |518115411|ref|WP_019285619.1| hypothetical protein [Rhodococcus sp. R04]

MTSADDLALAADLVRSAGTLALEMRAAGLEGTRKTSVSDVVTAADHAAEELVGSRLRAERPDDGLVGEEG

TSAPATSGRTWFIDPVDGTYNFLHGMTHWCSALALVDAEGPILGAVYHPESDQLWLGGRDHPTTCNGVPV

GPVRDLSLDLVSAATYLHPTRFGRPEMIAPWAEATRLCATVRMLGSASVDLAWTSDSRVGVWYQADCHPW

DWYPGQALVEAAGGVTRLVPVGGHTWHVAGSPTAVDQVAEAMITCLPRDEREAAAR

>Cg0911_Kytococcus_sedentarius |506259644|ref|WP_015779419.1| fructose 1,6-bisphosphatase [Kytococcus sedentarius]

MPGAPANPSLTDDAALAASLVRDAGQLAQRMRTEGVETRHKTSASDVVTAADLAAEELVVGRLCSERPAD

GIVGEEGAAREGTSGRTWVIDPVDGTFNFVQGLDWWCSALALADGGEVALGAVHHPGGDHVWVGGRDLPT

TRDGVELPPLADKPLAECALATYLHTSWWGVADVADPFAAVARRARALRMNGSGSMDLAAVADGRMDLWL

HHSVPEWDWMPGYALVEGAGGVARQVEVRGYTWSVAGPATAVGEVLELLLGE

>Cg0911_Janibacter_hoylei |495201215|ref|WP_007926001.1| fructose 1,6-bisphosphatase [Janibacter hoylei], 237

MDDHLADLPTDIDDAALAAALTRQAAMLAARMRSEGLRAQHKTSISDVVTAADHAAEELVESTLRRLRPD

DGIVGEEGAEAPSRSGRTWVIDPVDGTYNFLSGLGTWCSALALRDGEGLVLGAVHQPAVDESWIGGRGLP

TRLNGTAVGELADLPLAEVAMATYLHPAVLPDDSLREPWLAALSGAATVRMFGSGSCDLAAVAAGRLGVW

AQQSVPEWDWLPGAALVTAAGGRADQVEARGHVWSVAGRPTAVGQVAAALQS

>Cg0911_Tetrasphaera_jenkinsii |872697231|ref|WP_048549095.1| hypothetical protein [Tetrasphaera jenkinsii]

MLPVDDWLADLARAAERDDDRWLAEALVRRGGVIGSHWLGGELTRQTKTSVSDFLTQADAQAEADIAAAI

RALRPADGILGEEGTSTTGASGRRWIIDPIDGTYNYASRLESWCSAVALQGPGANLIGAIRQDSVEGTWL

GHVTEDGAAAWLNGSRLPRLADVPLAQLSLATYLHPTYLANPDALQPWLAACAGSATLRMNGSGSCDLAR

VATGRLGAFLQHSTAEWDWYPGVALVLGAGGVAETVEHRGFRWHVAGGAGAVAQLIERLRSA

>Cg0911_Arsenicicoccus_bolidensis |656266324|ref|WP_029212248.1| fructose 1,6-bisphosphatase [Arsenicicoccus bolidensis]; 208

MTSADDLALAADLVRSAGTLALEMRGAGLEGTRKTSVSDVVTAADHAAEELVGSRLRAERPDDGLVGEEG

TSAPATSGRTWFIDPVDGTYNFLHGMTHWCSALALVDAEGPILGAVYHPESDQLWLGGRDHPTTCNGVPV

GPVRDLSLDLVSAATYLHPTRFGRPEMIAPWAEATRLCATVRMLGSASVDLAWTADSRVGVWYQADCHPW

DWYPGQALVEAAGGVTRLVPVGGHTWHVAGSPTAVDQVAEAMITCLPRDEREAAAR

>Cg0911_Arsenicicoccus_sp. |913003915|ref|WP_050349016.1| fructose 1,6-bisphosphatase [Arsenicicoccus sp. oral taxon 190]

MTFAEDLELAAALVRTAGRLALQMRSAGVEGERKTSVSDVVTAADHAAEELVGSRLRAERPQDGLVGEEG

TSSPADSGRTWFIDPVDGTYNFLHGMTHWCSALALVDDDGPILGAVYHPESDQLWLGGREHPTTCNGVPV

EPVRDLPLDLVSAATYLHPTRFGRPEMIAPWAEATRLCATVRMLGSASVDLAWTSDSRVGVWYQADCHPW

DWYPGQALVEAAGGVTRLVPVGGHTWHVAGSPTAVDQVADAMMQCLPRDEREAAAR

>Cg0911_Arthrobacter_crystallopoietes |491409761|ref|WP_005267580.1| inositol monophosphatase [Arthrobacter crystallopoietes]

MTLPETPDHLNDADLAAWLVRSAAQLAGRMRADVKTFEEKTSISDVVTAADHAAEKLVVDTLRRVRPEDS

IFGEEGSSYTGTSGRTWVIDPVDGTYNFFTGATYWCAALALKDDGGVRLGAVYHPAEDALWLGGQDLPTT

RNGIRSGFALSAPLDQSCAAAYLHHTYLRDEAAAEPYLAAVLGAAAYRTWGSASCELAATSAGIFGVWFQ

QGLPEWDWLPGKALVEAAGGDTAVVQVKGYRWHVAGGKQQVADAVRLLQSQA

>Cg0911_Arthrobacter_phenanthrenivorans |503365323|ref|WP_013599984.1| fructose-1 6-bisphosphatase [Arthrobacter phenanthrenivorans] MTIGRHSAVELDPSLDDHQLAAALVREAGQLALLMRMAGLQSQQKTSISDVVTAADHAAEAYVLEQLQRC

RPHDGILGEEGASVQGTSGRTWVIDPVDGTYNFLHGSTYWCSAIALKDQSDVLLGAIFQPEEDKLWLGGR

AHPATLNGDPLTVFHDNKGERNATPVAELGAATYIHPTWLMDPLCAMPWHAAATSAAALRMLGSGSCDLG

RVADGQIGCWFQHSCPEWDWLPGKAIVRAAGGAVDTVRVNGLEWFMAGGTTAVRELRAALESGSVA

>Cg0911_Arthrobacter_alpinus |928486023|gb|ALE91362.1| fructose 1,6-bisphosphatase [Arthrobacter alpinus]

MTEQLDHRLSDVQLAAALVRNAGGLALRMRQRGLQELRDTAAQKSSVSDVVTSADLAAESYVVEQLRRCR

PEDSILGEEGAGYTGTSGRIWVIDPVDGTYNFYSGSTYWCSALALTDAAGVWPAGPDSLTDAAVLLGAIY

QPQEEKLWLGGTLQAATLNGKPIVVDPCQHVGQLSAGTYIHPKWLGDPQAGGPWQRAAQLPAALRMLGSG

SCDLGRVAQGELGMWFQHSAPSWDWLPGKGIVHAAGGDTAVVRVNGLDWFVAGPAVAVAELKAALAAGAL

>Cg0911_Renibacterium_salmoninarum |501200793|ref|WP_012243811.1| fructose 1,6-bisphosphatase [Renibacterium salmoninarum]

MPLPENLSPDLDDAQLASALVRNAGRLALAMRAEGLQGERKTSVSDVVTAADKAAEKYVMSQLRTVRPDD

GILGEEGARYLGNSGRTWVIDPVDGTYNFLSGSTYWCSAIALQIDGADALDPEVALGAVYQPQEEKLWLG

GKDIPATLNGEPLQPPSQAGLDQLSAGTYLHPTWLGRPEAATPWLAAASRLATFRLLGSGSCDLSRVAQG

ELGCWFQHSTALWDWLPGKAIVLAAGGSTAVVQVNGLNWFLAGGSTAVAELAEALGVPAS

>Cg0911_Nakamurella_multipartita |752723532|ref|WP_041370655.1| fructose 1,6-bisphosphatase [Nakamurella multipartita]

MRAASPDAEIAAALVRTAGRLAARMRSSGLLVEEKTSISDVVSDADKAAEALIVDRLTGIRPDDGVLGEE

GAAAPGRRTWVIDPVDGTYNFVSGLPAWCSALALTDGPEGAGATVLGAIYQATTDELWLGGPHLPPSLNG

VPLPALADRELHEIAITTYLHPPRLLDPRLRDGLVRAIAGAASVRIIGSGSIELAAVAAGRLGVWLHADP

PLWDWLPGSALVLGVGGVTDIFEYGGHRWHVAGPPTALAQAKAAVLGLG

>Cg0911_Nakamurella_lactea |656115349|ref|WP_029138831.1| fructose 1,6-bisphosphatase [Nakamurella lactea]

MAADPLDIDPSLSDLDLAATLVTEAGKLAAQMRAGGLEGFRKTSVSDVVTAADHAAEKYVLESLRRARPD

DSILGEEGASHQGSSGRTWVIDPVDGTYNFLSGLAYWCSALALTDAGTVALGAVYQPTAGELWLGGPGHP

TSCNGEPVVPLIDRALDQVSLATYVNPGTMTNDDALAPFLALVSGSAAPRILGSGSVDLAGVAGGRIGAW

AQHSCPDWDWLPGKALVEAAGGRTDVIEHRGRRWHLAGNAAAVTDLTERLLAS

>Cg0911_Nocardioides_alkalitolerans |918159103|ref|WP_052336879.1| fructose 1,6-bisphosphatase [Nocardioides alkalitolerans]

MSLEVGAGPAVDADDVRLAADVVRAAGRLAATMRDAGVEGLDVEQKTSVSDVVTAADHAAEELVASRLRT

ERPGDGLLGEEGVDEPGTTGRRWVIDPVDGTYNFLSGLSWWCSALALVDDGELVLGAVYHPHDDELFVGG

PGLPTTCNGVALPPLVDVPLERACAATYFHPRALAGEAGQAFGRATARAAAVRMLGSGSMDATAVARGRL

GLVFQHSVPLWDELPGAALVRGVGGRTRHEQAGGVDWYAVGAPTAVAEACAALAGR

>Cg0911_Nocardioides_luteus |780300434|ref|WP_045551733.1| fructose 1,6-bisphosphatase [Nocardioides luteus]

MTRPTEADVRLAAELVRGAGSLAAKMRHAGIGDSIETKTSVSDLVTAADKAAEKAIVDRLATEHPGDGIL

GEEGSTRESATGRVWTIDPVDGTYNFVRGLDWWCSALALTDGDDLVLGAIYSPAEDAVYVGGPELPTTRN

GVRLDQVLDTPLALACLTTYLHPAKLKHPVGEAYVRVASQAASIRILGSGSMDLTAIAQGKLHLFCQHSV

PDWDRLPGAALVLGVGGTTAQVEAGGVLWSLAGAPAAVAEAVEALAS

>Cg0911_Nocardioides_insulae |916610624|ref|WP_051217715.1| fructose 1,6-bisphosphatase [Nocardioides insulae]

MSGSPRAAHPRAADAELAGDLVRGAGELAHRMRADGVEVETKSSISDLVTAADHAAEEYILTRLARERPD

DGVLGEEGAARPSASGRRWVIDPVDGTYNFVAGLPWWCSALALVEGGPDEEEILLGAVHHPYADLLYLGG

PGLPTTRNGLPLAPLRDRPLAESCLATYLHPPYWGNAAGAVFGEMVTGAATLRMLGSGSMDLAAIAEGTL

QVSCQHTVPEWDRLPGVALVLGLGGAHRRVQAGGVEWSVTGVPTAVAEVCDRLLSGAGAGDPARGR

>Cg0911_Aeromicrobium_marinum |494137950|ref|WP_007077703.1| fructose 1,6-bisphosphatase [Aeromicrobium marinum]

MTVDDDALLAHALVTDAAALAARMRREPGLEINRKTSVSDVVTAADHAAEDLVVRRLVEAHPDDAILGEE

GASHTGTTGRRWVIDPVDGTYNFASGSDYWCSALALLDGDDLVLGAVAHHGSGTVVVGGPELPTTVNGTP

VDLLDAAADLSHLSAATYLHPAGVQRDEIREPFLRAAARPATIRMLGSGSMDLVGVATGRLGCWFQHTTP

EWDWYPGAAIVQGAGGVARQVVAHGVTWSVAGPAEAVGELARSLEGAGS

>Cg0911_Aeromicrobium_massiliense |908664324|ref|WP_049798255.1| fructose 1,6-bisphosphatase [Aeromicrobium massiliense]

MTVPRVRPRWASVGPVPTDDDARLAAELVTQAAALAARMRAGDALTVSRKTSVSDVVTDADHAAEELVLG

TLLRERPDDGVLGEEGASVESRSGRRWVIDPVDGTYNFASGSDYWCSALALVAGDELVLGAVAHHATGSV

VVGGPGLPTTRDGEPVGPVHDDPLGLVSAGTYLHPGFFAEPSVHDPWTRAVSRPATIRMLGSGSMDLVGV

ATGRLGCWFQHTTPAWDWLPGAAIVAGAGGTTRQVEVGGRTWSVAGGARSVAEVVAALEG

>Cg0911_Microlunatus_phosphovorus |754170342|ref|WP_041790144.1| fructose 1,6-bisphosphatase [Microlunatus phosphovorus]

MRTTTLAPVDTSVPVADSDVAASLVKEAGTLAATMLAEGLDTHYKTSISDVVSAADHAAEELVVRRLAEW

RPDDGLVGEEGASRSGKRTWYIDPVDGTYNFLSGLPYWCSAVGLTDESGSLAGAVYYPARDELWVGGRDR

PTTLNGVPLARLTDRPLAEVSVSSYFHPPRMSDEQQLADWRSVVGKAATVRVLGSASVDLGGVATGRIGL

FFQSGLGPWDWWPGVALVLAVGGAAEIVELHGSTYYIAGNTQSVSEAVTALRQPRP

**CysQ orthologs used for motif identification**

>CysQ_Corynebacterium_glutamicum |19552072|ref|NP_600074.1| inositol monophosphatase [Corynebacterium glutamicum ATCC 13032]

MTAQIDDSILTHRLAQGTGEILKGVRNVGVLRGRNLGDAGDELAQSWIARVLEQHRPNDGFLSEEAADNP

DRLSKDRVWIIDPLDGTKEFATGRQDWAVHIALVENGVPTHAAVGLPDLGVVFHSADARAVTGPYSKVIA

ISHNRPPKVALSCAEQLGFETKALGSAGAKAMHVLLGDYDAYIHAGGQYEWDSAAPVGVCKAAGLHCSRL

DGSELTYNNKDTYMPDILICRPELADELLEMCAKFYEENGTY

>CysQ_Corynebacterium_diphtheriae |544926445|ref|WP_021335613.1| inositol monophosphate phosphatase [Corynebacterium diphtheriae]

MTAQFDDATLTKRLAKGTGEILKGVRNVGLLRGRELGEAGDDLAQNWIARVLEQHRPDDGFLSEEAADNP

ERLGKDRVWIIDPLDGTKEFATGRQDWAVHIALVENGVPTHAAVNLPDLGVVFHSSEVRAVGGPYAKKIA

ISHNRPPAVATHIAESLGFTAEPMGSAGAKAMHVLLGDYDAYIHAGGQYEWDSAAPVGVSLAAGLHCSRL

DGTPLNYNNKDTYLPDVLICRPELADDILAMAAAFREENGSY

>CysQ_Corynebacterium_resistens |503653673|ref|WP_013887749.1| MFS transporter [Corynebacterium resistens]

MTAEIDDATLVQRLAQGTGEILKGVRNVGLLRDKKLGEAGDAIAQDWIARALELHRPEDSVLSEEAEDDR

TRLDNHRVWIIDPLDGTREYAGGRQDWAVHIALAIDGKIEHAAVGMPDLGMVFHTGDIRAVGGRPTNRLV

ISQNSTPEVATFIAEDLGMELVRMGSCGAKTTSVILGDNDVYVHAGGQYEWDNAAPVGIAQAAGLFTSRL

SGEKLKYNCSDPYLPDLLVCRPDASERVLASAAKFLEQTGSFK

>CysQ_Mycobacterium_tuberculosis |15609268|ref|NP_216647.1| 3'-phosphoadenosine 5'-phosphate phosphatase [Mycobacterium tuberculosis H37Rv]

MVSPAAPDLTDDLTDAELAADLAADAGKLLLQVRAEIGFDQPWTLGEAGDRQANSLLLRRLQAERPGDAV

LSEEAHDDLARLKSDRVWIIDPLDGTREFSTPGRDDWAVHIALWRRSSNGQPEITDAAVALPARGNVVYR

TDTVTSGAAPAGVPGTLRIAVSATRPPAVLHRIRQTLAIQPVSIGSAGAKAMAVIDGYVDAYLHAGGQWE

WDSAAPAGVMLAAGMHASRLDGSPLRYNQLDPYLPDLLMCRAEVAPILLGAIADAWR

>CysQ_Rhodococcus_equi |550238674|ref|WP_022593395.1| 3'(2'),5'-bisphosphate nucleotidase CysQ [Rhodococcus equi]

MTTRAVTPTTSDSASASGADSLAAVEIATEAGKLLLDLREGLATPGVDAAEIRAAGDRRSHELITGLLRD

RFPQDAVLSEEGADDLARLDAERVWIVDPLDGTREFGEADRADWAVHVALVDRGALTVGAVAMPAAGVTY

STMDGPVDLPPLRVVPRVVVSRTRRPAPVMELAAALDAELVEMGSAGAKAMAVVRGDVDVYAHAGGQYEW

DSAAPVAVARAAELHVSRIDGSELVYNQENPWLPDLLICRPEWADKALEVLAR

>CysQ_Gordonia_araii |494533021|ref|WP_007322471.1| inositol phosphatase [Gordonia araii]

MSSIPARGASASGPDSGLSDGELAARIAEGAGRILLGVRHGGLLDGALAGNAGDQLAQAWIGAALRRRRP

HDAVLSEEAEDVGDRATNDRVWIIDPLDGTSEFRHGTDDWAVHVALTVDGRPTAAAVAIPARGELFRSDD

IEGVGGPLTHRIAVSRFGGSYEAAHVANALGLQRVSIGSAGAKAMAVVRGDVDAYVHAGGQYEWDNCAPV

GVCAAAGLHCSRLDGSEIVYNQRIPYMPDFVICRTEIADDVLSALRGVW

>CysQ_Aeromicrobium_marinum |494138982|ref|WP_007078734.1| MFS transporter [Aeromicrobium marinum]

MTRDDAAFAADLAQQAGQLLLELQRTSGLAGKELGVAGDARSDELLLRLLAEGRPGDAVLSEESADTDAR

LSADRVWIIDPLDGTREYGMPGRSDWAVHVALWERGVTDPAITAAAVAQPALGTVYTTDDPAAAAVHRSR

PILLVSASRPPEFAAPVAEAVGGDLQTMGSAGAKAMAVVRGDADAYLHAGGQWEWDSAAPVGVALAAGLH

ASRVDGSPLTYNAAHPYLPDLLICRADLAPDLLAAIAAA

>CysQ_Tomitella_biformata |640112712|ref|WP_024795362.1| hypothetical protein [Tomitella biformata]

MTTTDFDLIADAALAAKIATSAGKLLLDLRLDASIQDPDELRDEGDRRAHEHIMALLDKHFPDDAVLSEE

GVDEKSRLRAARTWIVDPLDGTNEYGFAERGDWGVHIALVIDHIAVLGAVAIPSLGITYGSGTVTPPMFG

DTGRLKIAVSRSRRSTVAAVIADGLGAEIVRMGSAGAKAMAVVRGDVDIYAHSGGMYEWDSAAPVAVAAA

AGLHTSRIDGSPLRYNHDDPWLPDLLICRRDVAAEVLAIVNR

>CysQ_Blastococcus_saxobsidens |754151172|ref|WP_041776812.1| MFS transporter [Blastococcus saxobsidens]

MDDTAVAALAARAAADALVPLRSSGLTGRPLGDAGDAAGQRAIVAVLAAERPDDVVFSEEAVDDARRLSA

GRVWIVDPLDGTREYGNPERSDWAVHVALWADGDLVAAAVALPALGQVHVTDPAPPLPPRPDGPVRIAVS

RTRPPAEAQAVAAALGGELVPLGSAGYKTLAVVRGEADAYVHSGGMYQWDSAAPVAVARAAGLVTCRLDG

SPLVYNRPDPSLPDLVVTRPELADRVLAAVRSAR

>CysQ_Actinoplanes_utahensis |724740466|gb|KHD72834.1| MFS transporter [Actinoplanes utahensis]

MDAAFARWLADRAGQVLLQVRAEMGHADGKALKTAGDQAAHQLLRAELARWRPADAVLSEEDEHARVAWT

EDDTALARPDRLSANRVWIVDPLDGTREFSEEGRDDWAVHVALWTADSSAPARLTAGAVAMPARHCTLAT

DNPPAYPPMPLESATGGAIRIAASRTRPPAFVTALAEEIGAELVPMGSAGVKIAAVINGDADAYVHAGGQ

YEWDSAAPVAVALATGLHASRIDGSPLTYNQADTKLPDLVVCRKDLAPRLLAALQRHLPSQ

>CysQ_Dietzia_cinnamea |494905097|ref|WP_007631142.1| MFS transporter [Dietzia cinnamea]

MRDLDDHDLASHLATGIGDIVRGTRAGGLLHGRSLARVANDVAEDWTGEVLAVHRPDDGILSEGVADDRS

RLAKSRVWIIDVIDGTKEFSTGRSDWSVHVALVVDGRPTVAAVGLPDAGRVFRSDQVDHVDGPSSGAMVI

SRNNPPAGIHGAADELGLHVRPMGSAGAKMLSVLLGDADVYLHAGGQNEWDQAAPVGVALAAGLHASRID

GSPLEFNKSDTYSPDILVCRPDLTEKVLAVTAGHPVWTA

>CysQ_Actinopolyspora_iraqiensis |651331218|ref|WP_026455377.1| MFS transporter [Actinopolyspora iraqiensis]

MTTDARLAARLAEQAGEELLRLRDDVPAGAGPTDPKELGRRGDAAANELLVRSLREQRPGDAVLSEESAD

DRRRLGAERVWIVDPLDGTREYGLGRPDWTVHVALWEAAAAAREPGTGITAAAVARPDSGEVLVTDDIAA

GKAGAKIPGNRVRILVSDTRPPDFANAVAARLDADLVPRGSAGAKAVAVLRGEADAYLHAGGQYEWDSAA

PVGVAHAAGLYASRIDGSPLRYNAPDPYLPDLLICRPGLAGDLLAAVAAATTDTNDA

>CysQ_Smaragdicoccus_niigatensis |516910938|ref|WP_018161434.1| hypothetical protein [Smaragdicoccus niigatensis]

MNDAELAAHLAVEAGKLLKTLRADSGLHGWELGKKGDRLSDDYLLDQLAIERPNDAVLSEESADSEDRLT

SERVWIIDPLDGTREFGILGRPDWAVHVALWESGSLSACAVSQPDICEVYSTADAVLGPRTDGPLRIVCS

ASRPPAVVVRLRDELGAELIPMGSAGAKAMAVLRGDADAYVHGGGQWEWDSAAPVGVALAAGLHCSRLDA

SPLLYNQPYPYLPDLVICRPEFAEQILAVTRRH

>CysQ_Segniliparus_rugosus |494736155|ref|WP_007471593.1| inositol phosphatase [Segniliparus rugosus]

MHVTDEEFAARLAHDTGQLLLAVNNTSLLSSHDLGDAGDSLAQDFIARTLELHRPNDAVLSEEARDDAAR

LTAKRVWIVDPLDGTSEYRRRGEDWAVHIALVQNGVPTASAVALPPRGTVFRSDTVGKADGPLTRSMVVS

RGRPPMEALLVAKRLGMQLDPVNSAGVKAMAVVEGRADAYVHAGGQHQWDSAAPVGVALAAGLWCSRLSG

APLVYNTRSTYLPDLVICRQEVKDDILSVLHALRR

>CysQ_Frankia_alni |754542379|ref|WP_041940333.1| MFS transporter [Frankia alni]

MNTDDTRRPATVPVDIATDELDDDVLARRLATDAGKLLLRIREEVGFAEPATLRDAGDARSHQLLARALA

RHRPQDAVLSEEGADDPARLSAERVWIVDPLDGTREFAEPGRSDWAVHVALWRAGELIAGAVALPALNVT

LSAGGDAPAAARPAAAGPPRIVASRTRAPRIVGEVAEALGASVVPMGSAGAKAAAVIRGRAEIYLHAGGQ

YEWDSAAPVAVARAVGLHTSRLDGSPLRYNQPDLLLPDLVICEASLAPAVLAAIRAADPRP

>CysQ_Sporichthya_polymorpha |655914942|ref|WP_028985049.1| MFS transporter [Sporichthya polymorpha]

MDDLALARELAAAAGERLAELRAGWEGDEADLRKAGDAAAQDLLAARLSTARPDDAVLSEEAKDNPIRLD

ADRVWIVDPLDGTREFGEGRHDWAVHVALWERSAGAAGDLTVGAVALPGRGLVLCSDGAQRVPEAADGSP

IRIAVSRTRPPAVAVAVAEALGAELVPMGSAGYKVTAVVLGEVDAYVHGGGQYEWDSAAPVAVARAAGVH

TSRLDGSPLVYNRPDPLLPDLVIARAELAEPLLAAIAAASTT

>CysQ_Modestobacter_marinus |504551451|ref|WP_014738553.1| 3'(2'),5'-bisphosphate nucleotidase CysQ [Modestobacter marinus]

MTPSDHQTAAQLATEAGELLLQVRARDFADATARKAAGDAEAHRLLLDRLAALHPDDAVLSEEGADDPIR

LVSRRVWIVDPLDGTREFSELDRADWAVHVALWERGELTAGAVALPAEGTTLSTVDVAETPSRSDGPMRL

AVSRSRPPALVADLATQLGAELVAMGSAGVKAMSVVRGDTDAYVHGGGQYEWDSAAPVAVARAAGLHTSR

LDGSPLRYNRPDPYLPDLLICRPELADRLLTALSPR

>CysQ_Glycomyces_arizonensis |916785754|ref|WP_051392810.1| hypothetical protein [Glycomyces arizonensis]

MDHAPEADGEPTATPDAATLEDTAFAAELATGAGELLLKLRAELGFGDPRALKSAGDNGANDHLLGRLAA

ERPDDAVLSEEGKGESGRIGTVRLGADRVWIIDPLDGTREYAEAGRDDWAVHVGLWQRGLGLTAAAVALP

AQGVTLRTDERHPVAGPRPDRPRIAVSRTRPPAEAEAAAAALDAELVPWGSAGAKAMAVVAGKVDAYVHA

GGQYEWDSAAPVAVALASGWHASRIDGSPLRYNNPDPYLPDLLLCRPDLAEPLLEAIAEAS

>CysQ_Stackebrandtia_nassauensis |290568038|gb|ADD41003.1| inositol monophosphatase [Stackebrandtia nassauensis DSM 44728]

MLDQSDAALARSLAQQAGQELLLLRDKLGFGDATVLRREGDQRAHEFLMSALAEFRPGDAILSEEGKGEQ

GRTGASRLNAERVWIVDPLDGTREYAEAGRDDWAVHVALWERGTLSAGAVALPAQDTVLGADAPPTPLPP

QGRIRMVVSRSRPAAFLTALAEVIDAELVPMGSAGAKTAAVITGKVDAYVHAGGQYEWDSAAPVAVARAA

GLHTSRIDGTDLTYNNSDPKLSDLVVCRPELSALILKGIAAVRLHPLPTQGSINQFIKGGADERLRLSRQ

SS

>CysQ_Jiangella_gansuensis |916818693|ref|WP_051425749.1| MFS transporter [Jiangella gansuensis]

MASTDAALAARLATEAGGLLSELRRGHLDANEPGTGTRELRDAGDRAAQRFLATELARERPGDAVLSEED

DDDAARLSASRVWIVDPLDGTREFAEGRDDWAVHVACWAGGDLVAGAVALPALDTTLVSEPAPGVPARRG

GPVRIAVSRSRPPAVAEHIAGVLGAELVPIGSAGFKAAAVVRGEVDAYLHAGGQFEWDSAAPVAVARAAG

LHASRVDGSALRYNQPSPYLPDLLVARPELADALLAATAGHDAQPATPDSRRTGRSAP

>CysQ_Kineosporia_aurantiaca |918641036|ref|WP_052526882.1| MFS transporter [Kineosporia aurantiaca]

MTIEPGGDDARLARTLAHAAGLALLDVRAGSAEGKDLKDAGDARAQAVLASLLEFHRPDDAVLSEEAADS

AARLTAGRVWIIDPLDGTREFSERPRTDWAVHVAFWQGGDLAAGAIALPARDVTYGTDDPPALPPRAEGP

IRLAVSRSRPPAFVTQVAEELGAELVPMGSAGVKCASVWLGEADAYLHAGGQFEWDSAAPVAVARAAGLH

TSRIDGSELVYNRPDPKLPDLLVCRTEFAATILAAVSRHMSAEVSEVITR

>CysQ_Arthrobacter_arilaitensis |503114030|ref|WP_013348751.1| MFS transporter [Arthrobacter arilaitensis]

MSDVQFAAEIAKESGRVLLQLRAQARSKAMTESELKVAGDRASQEFLNRRLVESYPDDSILSEEATDDLT

RLSNDRVWIIDPLDGTREYSEGRDDWVVHVALWKSGQLVVGAVALPGLDELLVSRSEPMGALNRGMRPVR

IAVSRTRPPQIVAELSNHLDIEFVPMGSAGFKACAVARGEVDAYIHAGGQYEWDSAAPVAVAASRDLHVS

RIDGSPLEYNQENPYLPDLLICRKELRAALLGAMAGITLQTT

>CysQ_Microbacterium_indicum |656174203|ref|WP_029151071.1| MFS transporter [Microbacterium indicum]

MVPAVGPHDLPDAEIARTLATEAARRLVEVRNDNPDVEGKALKDLGDQSAQALLAARLAELAPGDAVLSE

EAADSAARLGADRVWIIDPLDGTREFSEHRDDWAVHIALVVDGSLALGAVALGGPDVVLVSSEVDGHAAP

SGDRIRIAVSRSRAPQLVQQVAESLGAELVPMGSAGVKICSVVRGEADAYVHGGGQYEWDSAAPVAVART

AGLYTSRLDGSELIYNQPNPYLPDLVVCRPEISGDVMNAVGAALASQPE

>CysQ_Paraoerskovia_marina |656320976|ref|WP_029252974.1| MFS transporter [Paraoerskovia marina]

MTPVRSELDDHELAVHLAEGAGAVLLALRAEVDASGGVFDPRALKDEGDRRAQAWLATELAATAGDDAVL

SEEAVDGAARLDADRVWIIDPLDGTREFAERAESGEWRDDFAVHVALWCRGSGLCAGAVALPARGLVRST

KDAQPVDAERLADVAAGRRPLRIAASRSRPPEFVTDLAARGDVELVPMGSAGVKVMAVVDGTVDAYVHGG

GQYEWDSAAPVAVARAAGLECTRLDGAPLEYNRENPWLPDLFVIHPVLAPQLRAALTAVGVDQKGSV

>CysQ_Lysinimicrobium_mangrovi |754853944|ref|WP_042214664.1| MFS transporter [Lysinimicrobium mangrovi]

MTYALTDAALSALLANAAGDELLRVRRESGLEGKELGKAGDAAAQALLAGLLAEHRPDDAVLSEEAKDSE

ARLSADRVWIIDPLDGTREYSEGRADWAVHIALWERGELTLGAVGIPGEELVLVSDTVAPAPAPADGAQI

SLAVSRSRPPAVTEPVREALDASLLPMGSAGVKIAAVVRGQVDAYVHAGGQYQWDSAAPVAVARAAGLHT

SRIDGSPLVYNDADPYLPDLVVCRPELAPRILAAIAENTDGDS

>CysQ_Kytococcus_sedentarius |506260019|ref|WP_015779794.1| MFS transporter [Kytococcus sedentarius]

MTDDLQTAVDLATGAGQQLLAVRAQWRGADPKALKDLGDRTAQAWLAAELARRCPEDAVLSEEAEDVLDR

SAARRVWIIDPLDGTREFSEPPRDDWAVHVALWEDGELTTGAVARPGAGITYHSAEGQPLAERSEGPIRL

AVSRSRPPAFVDDLKNRLDGELVPMGSAGVKVMSVVSGECDAYVHAGGQYEWDSAAPVAVARAHGLVTHR

VDGSPLVYNRENPWLPDLVVARPEVHDEVVAALAEINLPD

>CysQ_Phycicoccus_jejuensis |738945253|ref|WP_036830162.1| MFS transporter [Phycicoccus jejuensis]

MTTPSADDHAVAAEVAEQAGALLLALREEHADLEGQELKDLGDRRSHELMMRLLGELRPDDAVLSEEGAD

DPVRLSASRVWIVDPLDGTREFSERPRDDWAVHVALWQDGDLVAGAVGIPARGVVHRMDRPSPLPARPDG

PVRLAVSRSRPPAFVVSLAESLGAELVAMGSAGVKAMAVVDGTVDAYVHAGGQYEWDSAAPVAVALGHDL

APTRLDGSPLEYNRENPWLPDLVVARPEVLAEVREALDRLA

>CysQ_Tetrasphaera_japonica |872703637|ref|WP_048555291.1| MFS transporter [Tetrasphaera japonica]

MTSATAYIDRIDTSGYADLDDHALARALADAAGRLLVELREGWDGEAGDLRKAGDAQSHELLVRLLAQAR

PDDAVLSEEGADDTARTRARRVWIVDPLDGTREFGEVPRDDWAVHVALWEDGELAGGAVARPARRSTLAT

DETLSVGERAKGPLRLAVSRSRPPEFVTRLAEILDAELVPMGSAGIKATAVVDDIVDAYVHAGGQFEWDS

AAPVAVARAAGLHTSRIDGSPLVYNREDPTLPDLVICRPEFSGRILDAISSLS

>CysQ_Humibacter_albus |703185399|ref|WP_033371011.1| MFS transporter [Humibacter albus]

MFTELAAGAGEVLLGLRGEIGDAPYDVKQLRDAGDRRSQAWLAAELTKRAAGDAVLSEEASDDPARVGAD

RVWIIDPLDGTREFAERGPDGVWRTDWAVHIALWERGRGLVGGAVGMPARGLILHTGPGHPEAPVESWDG

SRPLRLAVSRTRPPAVVDAIAARHDVELVPMGSAGIKAMAVVTGEVDAYVHGGGQFEWDSAAPVAVARSA

GLHTSRLDGTQLEYNRENPWLPDLAICAPVAWSTLSKWIAEASAAT

>CysQ_Isoptericola_variabilis |503605409|ref|WP_013839485.1| MFS transporter [Isoptericola variabilis]

MSRQSPPPPDASHDDASLAVALARSAGRVLLGLRDDAGRTGLAGRALRDAGDAAAQAWLAAALGHARPGD

AVLSEEAADDPARLLADRVWIVDPLDGTREFAERTPDGGWRDDFAVHIALWERGAGLSAGAVALPARGVV

HGTVGAGPQQPADDVDHVDHVDDVLAGRRPLRLAASRSRPPAFVTALAARGDVELVPMGSAGVKVAAVVE

GTVDAYVHAGGQYEWDSAAPVAVASAAGLVCTRLDGSPLEYNRPDPWLPDLFVCHPALAPHLRAALSQTG

PRSKEGAQP

>CysQ_Longispora_albida |517162912|ref|WP_018351730.1| hypothetical protein [Longispora albida]

MITPAADAAFARWLARAAGDALLSLRADMGFDDGEALKNSGDKVSHELLMTALARWRPADAVLSEEGADD

HARLAAERVWIVDPLDGTREFSEAGRTDWAVHIALWVRGELVAGAVGMPARGVILGTDTPPAFPVRAAGP

LRLACSRSRPPAFVAELAEAVGGELVPMGSAGVKASAVVTGEVDAYVHAGGQYEWDSAAPVAVARATGLH

TSRIDGSVLNYNQPDPKLPDLLICRPDLSQVLLAQLSSI

>CysQ_Micromonospora_carbonacea |763079658|ref|WP_043960858.1| MFS transporter [Micromonospora carbonacea]

MTEDGTGRLDDQAFAEWLAGEAGVALTALRAEQGFADAKALKDAGDKVSHDLMSTALARWRPADAVLSEE

QADSRRAWADDPSAGDRPTRLTADRVWIIDPLDGTREFSESGRDDWAVHVALWQRSAGPDGALVAGAVGM

PARTGPDGTPLVLGNRVPAAPAVDGPLRIAVSRSRPPAFVSELVEGIGARAVPMGSAGVKVCAVVTGEVD

AYVHAGGQYEWDSAAPVAVALGAGMHASRIDGSPLRYNRDDPRLPDLLVCRPEHAGSLLAAIDRYR

>CysQ_Nocardioides_alkalitolerans |655023960|ref|WP_028472868.1| MFS transporter [Nocardioides alkalitolerans]

MPTSSSPSAGDLASYDDHALAAALATLAGERLVDVRTTGVAESWEPSELKDTGDRVAHELLVALLAEHRP

GDAVLSEEGSDDKVRLGSDRVWIVDPLDGTREFGEPPRDDWAVHVALWERGAGDGSQVGDLTAGAVAQAA

LGRTFSTAHPSVVPPRTAPRPRVVVSRSRPPAVVEPVVAALGADLVPMGSAGAKVMALVRDVADVYVHAG

GQYEWDSAAPVAVARAAGLVTCRLDGSPLLYNQDDVYLPDLLVCRPELADAVLAALRPHLPR

>CysQ_Actinomycetospora_chiangmaiensis |916361754|ref|WP_051086064.1| hypothetical protein [Actinomycetospora chiangmaiensis]

MTPGSPAYAEDAALARELADATGRLLLDLRDATPTGRDREYAGDEQAHEYLTRELARRRPDDAVLSEEGS

LDPRDRRGRRLWIVDPLDGSSGFGRGTDEWGVHVALVVDGQLAAGAVAVPGLGALVDSDTVKHIADAPVG

DELAVTVSRSRPPHELRAVERAFPVRLVKRSAAGVKTLAVLDGEADVYLHSGGQYEWDNAAPMAVAMAAG

LLATRIDGSPVPYGQDDPYSPDLLIARPSVHAAVLSVLTGGR

>CysQ_Streptomyces_ochraceiscleroticus |664546387|ref|WP_031061638.1| hypothetical protein [Streptomyces ochraceiscleroticus]

MPTRDRADHELAHRLAEAAGALLLALRAEPGRTTRLSAEGDRRSNEFLLAALAAKRPGDAVLSEESADDP

SRLSARRVWIIDPLDGSREFGEEGRTDWAVHVALWIDGALAAGAVALPAVGRTFSTWQAVQAAPDPEQLT

LLVSRSRPPALVGDLAERLNARLHPMGSAGAKAMAVLRGAGSAYVHAGGQYEWDSAAPVAVALAHGLRVR

RLDGSAPRYNQPDPSLPDLVICRPSAAPELWGALDALASHM

>CysQ_Nocardiopsis_lucentensis |648435571|ref|WP_026127322.1| MFS transporter [Nocardiopsis lucentensis]

MKSIRNDHEVARDLATEAGQLLLRLRARHGFAEPDVLRTLGDRTSHEFLFSALGRLRPSDAVLSEEGADD

LSRLRARRVWIIDPLDGTREFAEAGRTDWAVHVALWENGDLVAGAVALPAQGSTVSTVDPPWLPDERPGD

QRLRITASRSRPPAFVQRMANQLGAEVIPMGSAGAKICAVMLGIADIYVHAGGQYEWDSAAPVAVARAAG

LHTSRIDGAPLRYNVSDPLLPDVLVCRSELSGMLLASIRDGADLDSAGPHAGG

>CysQ_Nonomuraea_coxensis |648523022|ref|WP_026214773.1| MFS transporter [Nonomuraea coxensis]

MMTIRDDHAVAADLANEAGERLLQVRRRLGFDDAAALKAEGDRSSHVFLMESLARLRPSDSVLSEEATRE

ERLDPRRLAAERVWIVDPLDGTREFSEEGRADWAVHVALWERGRLTAGAVALPAQGRTLTTAEPPKLPAF

SGGRFRIAVSRTRPPEFVQKLAYLAGADLVPIGSAGAKISAVLTGEVEAYVHAGGQYEWDSAAPVAVAQA

AGAHASRVDGAALAYNQADPSLPDILVSLPELATSLLAGIRDLHR

>CysQ_Actinomadura_oligospora |651282404|ref|WP_026414381.1| MFS transporter [Actinomadura oligospora]

MTDLGTDRTTDHALAAELATEAGRLLLAVRDELGFGDARTLKDTGDLRAHEYLMARLAERCPGDAVLSEE

GRASVAGKRSAGSSAVPTANTAVEERLAAPRVWIVDPLDGTREFSEEGRLDWAVHVALWTGDHLAAGAVA

LPARGVTLHTTASGAAVLPTGPAASGWTADTLGSTRAEAAGLVVPEPAEPGRLRIAVSRTRPPAFLEALA

GRVPVELDLVPIGSAGAKISAVLLGEVDGYVHAGGQYEWDSAAPAAVALAAGAHASRVDGSPLVYNRADP

RLPDILVCHPGSASTLLDGIREPSESAP

>CysQ_Promicromonospora_sukumoe |518861811|ref|WP_020017701.1| 3'(2'),5'-bisphosphate nucleotidase CysQ [Promicromonospora sukumoe]

MNAPSPTLPPASLDDDSLARALASGAAEVLLALRAEVDAAGGSFVARELKDAGDAAAQAWLAAALSAARP

GDAVLSEEAKDDAARTGADRVWIIDPLDGTREFAERHADGPLAGQWRDDFAVHVALWERGPGLTSGAVAL

PARGQVLTTADGVVRDEDSAAVLSGDRPLRIAASRSRPPAFVAALAERDDVELVPMGSAGVKMMAVADAT

VDAYVHGGGQYEWDSAAPVAVAFSRGLVCTRLDGSELEYNRENPWLPDLFVCPPALAPRLRELLDTVGVA

SVAGADRKEGARS

>CysQ_Pimelobacter_simplex |917531878|ref|WP_052138295.1| MFS transporter [Pimelobacter simplex]

MTFEPGTPAPEVVADDHLFAAWLAEAAGARLLEVRGEGLEGKELKDAGDRAAHVLLMELLAAYRPDDAVL

SEEALESAADKDARLTAAKVWIVDPLDGTREFSEPPRDDWAVHVALWQDGTLVAGAVAQPVIGETFNTGT

PPVVPARTSQRPRIAVSRTRPPAFVQALAEELDAELVPMGSAGVKMMSVVRDVADAYVHAGGQYEWDSAA

PVAVARAAGLFTSRVDGSELVYNQDVYLPDVIVCRPELADKILSFIAANGTD

>CysQ_Lechevalieria_aerocolonigenes |772750819|ref|WP_045313940.1| MFS transporter [Lechevalieria aerocolonigenes]

MTETDGELAARLATGTGRLLVRVRPGDGDRIAQEFLAAQLKEHRPHDAVLSEEAPDDRRRLTSERVWIID

PLDGSREYAEPGRTDWAVHVALWHAGRLVAAAVALPALARTFGTDQPVALPAPHARPRIAISRTRPPAFA

QRVADAVHGVLVPMGSAGAKAMAVVRGEADAYLHAGGMYEWDSAAPVAVARAAGLHASRVDGRPLEYNQP

DPWLPDLLICRPELAGVLR

>CysQ_Streptacidiphilus_jiangxiensis |755107190|ref|WP_042457958.1| MFS transporter [Streptacidiphilus jiangxiensis]

MTADDHRLAADLAEAAGEQLLALRSAQGFADAALLRERGDRTSHEYLAARLARERPDDAVRSEEARDAEP

GRDAADRVWIVDPLDGTREYAEEGRDDWAVHVALWERGVLSAGAVAIPARGAVLTTAFPPKVPSVWPGPP

RIAVSRSRPPEFVTRLGAALNAQLVPMGSAGVKIASVVQGLTDVYVHAGGQYEWDSAAPVAVALAAGLHC

SRVDGSPLRYNQPDPWLPDLLVCRPQDAEQLLAAIAETR

>CysQ_Thermobifida_fusca |740201261|ref|WP_038043217.1| MFS transporter [Thermobifida fusca]

MSTIRNDHEVARDLAFEAGQRLLRLRERHGFDEPEVLRTLGDRVSHEFLFSALGRLRPSDPVLSEEGVED

PRRFRAHRVWIIDPLDGTREFGEAGRTDWAVHVALWENGELVAGAVSLPAQGSTLSTVDPPWLPEERPAG

QRLRITTSRTRPPEFVRRLANQLGAELVPMGSAGAKISAVMLGIADIYIHAGGQYEWDSAAPVAVARAAG

LHTSRIDGSSLTYNRPDPYLPDILVCRSELSGMLLASIRNVADMDNAGPHT

>CysQ_Streptosporangium_roseum |759974220|ref|WP_043658079.1| MFS transporter [Streptosporangium roseum]

MMTIRDDHVLAMDLATEAGERLLALREKQGFADPSALRKEGDASAHVFLMDALARLRPSDSVLSEEATRE

ERLDPRRLKAERVWIVDPLDGTREFAEEGRGDWAVHVALWENGRLSAGAVALPAQGRTLSTLEPPVLPAV

RPEARPRIAVSRTRPPEFVQNLAGLAGADLVPIGSAGAKIAAVLTGEVEAYVHAGGQYEWDSAAPVAVAL

AAGAHASRIDGSPLTYNQDDPSLPDILVSLPGLAPTLLAGIRDLHR

>CysQ_Spirillospora_albida |663124577|ref|WP_030165466.1| MFS transporter [Spirillospora albida]

MTEKTADDHALAAELAMVAGGLLLGVRAEVGFADARNLKDTGDLRSHEYLMAALRERCPGDAVLSEEGRS

SVAAKRSDGDDRAPTANSSDADRRSAGRVWIIDPLDGTREFSEAGRRDWAVHVALWTRDETGAGRLAAGA

VALPAQGLTLRTSATGGALLVRDDPADQPGEVPLHAPAPDAGKLRIAVSRTRPPAFVQRLAERLDLEVEL

VPIGSAGAKISAVLLGEVDAYVHAGQYEWDSAAPAAVALAAGAHATRIDGSVLAYNREDPRLPDILVCHP

VSASTLLTGIRDVSDALPA

>CysQ_Ilumatobacter_coccineus |505255185|ref|WP_015442287.1| 3'(2'),5'-bisphosphate nucleotidase [Ilumatobacter coccineus]

MPVDGTAPETDAELATRLAVQTGQILVKLREELFGQGASTWDVKDAGDAAAQAFLARELHEHRPDDAVLS

EEEGKTDQRRFTSDRVWIIDPLDGTREFGEPGRFDWAVHVALWTADRFGAAAVSLPAINAVFSTDPAPPH

PVFERERPRLVTSRSRAPYSAILVSEGLDCDAVRLGSAGAKAMSLVMGEADIYVHDGGMYQWDSAAPAAV

ALAAGFHVSRLDGSPIVYNERDPWLPDFIVCRPELAEPVLKSIWG

>CysQ_Sphingobium_ummariense |544909505|ref|WP_021319568.1| MFS transporter [Sphingobium ummariense]

MSGAITDADLAAHLAETAGRILLEVRASGMFGGKSLGKAGDQTANQFLCHALREQRPDDGLLSEEEKDSA

ERLNKTRVWIVDPVDGTREYGEARADWAVHVGMAVDGEPEIGAVALPGLDGGVVLRTDQPRAVPPAPATL

RMVVSRTRPAREAVDVAEQLGAELVPMGSAGAKAMAIILGQADIYLHSGGQYEWDSMAPVAVARAHGLHC

SRIDGSPLIYNQADVYMPDLLICRKEHARQVLDLVAAARI

>CysQ_Erythrobacter_longus |736964196|ref|WP_034960677.1| inositol phosphatase [Erythrobacter longus]

MNQPDLTDAQLAAQLAEVAGRIALNVRASGLLAGRSLGDAGDATANEFLCSAIRSNRPDDGLLSEEEKDN

PARLEKSRVWIVDPVDGTREYAEERSDWAVHVGLAIDGVATTGAVALPDLNGGIVVATDEVKPLPAAASK

PRFLVSRTRPAKEAVAVSKAMDGELVGMGSAGAKAMAVLRGEAEIYLHSGGQYEWDSCAPVAVAQAHGLH

CSRIDGSPIRYNQADTYLPDLLICRTEYAAEVLALVAGL

>CysQ_Hirschia_baltica |506307617|ref|WP_015827392.1| MFS transporter [Hirschia baltica]

MSELSDAQLAAELADAAGKILLEVRASGLVDGKALGKTGDETSNAFLMQALAHQRPDDAVLSEESKDDKS

RLEKSRVWIIDPVDGTREYGEGRTDWAVHVALSIDGKPSIGAVALPGMDLVLRSDQPPALKPMNNPPKML

VSRSRPGKEATAVAEAMGAELLPMGSAGAKAMAVVRGEADIYLHSGGQYEWDNCAPIAVANAAGLHTCRM

DGSEFKYNCDDTYLPDLLICRPELADAARKAIDALNLD

>CysQ_Hirschia_maritima |517826140|ref|WP_018996348.1| 3'(2'),5'-bisphosphate nucleotidase CysQ [Hirschia maritima]

MSDLSDAELAAKLAEAAGKILLEVRASGLVEGKALGKAGDETSNAFLMQAIQAQRPDDAILSEESKDDKS

RLDASRVWIIDPVDGTREYGEGRTDWAVHVALSIDGKPSIGAVALPGMDLVLRSDTPPALKPINNPPKML

VSRSRPGKEAVAVAETMGAELLPMGSAGAKAMAVVRGEADIYLHSGGQYEWDNCAPVAVAAAAGLHTSRL

DGSEFKYNCDNTYLPDLLICRPELAEAARKAIDELDLAD

>CysQ_Burkholderia_sprentiae |652940802|ref|WP_027194286.1| inositol phosphatase [Burkholderia sprentiae]

MNAVSTALSIDDDHRLAVHIATAAGRALNAIRASAVLAGKPLGALGDATANELIAGILARSRPEDGFLSE

ESAPDPKRLALDRVWIIDPLDGTREYGERLEGREDWAVHVALTVGGEPRACAVALPARGVTFGTLSPPEL

PPAPEGRLKILVSRSRAPKLALMVAQRLNAELVPMGSAGAKAMAVLRGEAHAYLHAGGQYEWDSCAPVGV

ALAAGLHASRIDRSPCLYNGADVSMPDLLICRREIAESMLAAIAAAQ

>CysQ_Sphingopyxis_fribergensis |746542260|ref|WP_039574496.1| inositol phosphatase [Sphingopyxis fribergensis]

MGAGETDEQLAERLAAAAGAILLDLRAEGELEGKALGLAGDQQANAMLCREIRAARPDDALLSEEEKDSL

VRCGQSRVWIVDPLDGTREYGERRGDWAVHVALAVDGVATVGAVALPALGVTLTSGTPVALKPANEPLRM

LVSRTRPAAEAVFVAEKLGAELVAMGSAGAKAMAVVRGEADIYLHTGGQYEWDNCAPVAVAQAAGLHVSR

VDGSPIRYNNPDTYLPDLLICRKEFADEVLRLAGQYSPD

>CysQ_Altererythrobacter_marensis |831206344|gb|AKM08181.1| Putative inositol monophosphatase [Altererythrobacter marensis]

MTAMTDADLAAHLAKVAGRILIEVRDSGMFKGKQLGSAGDEVSNRYLLHALRAQRPDDGILSEESRDTLT

RIDKSRVWIIDPVDGTREYGEARADWAVHVGLSIDGEAKVGAVALPGLNGGEVLRSDRVAPLASAAERPR

LVVSRTRPAPQAEAVAQAIGGELVEMGSAGAKAMAVVRGAAEIYLHAGGQYQWDNCAPVAVAAAHGLHCS

RIDGSPLRYNGRDTSLPDLLICRPEWAERVLAEVRQTI

>CysQ_Acidocella_aminolytica |890068535|ref|WP_048878551.1| inositol phosphatase [Acidocella aminolytica]

MTDLELAKIISEGAGKILLELRATGLFEGKALGKVGDRMANEFIMTALRETRPDDAVLSEEEKDNADRLS

AERVWIVDPLDGTREYGECRTDWAVHIALSIGGKPAIGAVALPGLGQVLLSEPQPLPEIAPKLRVLVSRT

RPAKEALAVAEALNAELVPMGSAGAKAMAVLRGEADIYLHTGGQYEWDNCAPAAVALAAGLHATRADGSP

LVYNNADPYLPDLLICQKELAPKVLEALAKVPA

>CysQ_Chromobacterium_subtsugae |828152567|ref|WP_047244291.1| inositol phosphatase [Chromobacterium subtsugae]

MPDNDHQLAADIAAQAAELLNQLRQGNTAGDKALGALGDAKSDALIAHLLRQARPDDGLLSEESAPDDAR

LRLRRVWVIDPLDGTREYSERERERSDWAVHVALTEDGLPTACAVALPALGQLFSTAAPALAGAPPGPIR

ILVSRSRPPAIAERVAAMLDAELAPMGSAGAKAMAVLRGEAHAYLHSGGQYEWDSCAPVGVALAAGLHAS

RLDGSPCRYNQPDPFMPDLLICRRELAPALLDAIARAQAESA

>CysQ_Chromobacterium_violaceum |860373289|ref|WP_048403946.1| 3'(2'),5'-bisphosphate nucleotidase CysQ [Chromobacterium violaceum]

MPDDHQLAAHIAAAAAQRLLDLRRRHPEGGKALGARGDTEANALILDMLRAARPDDGLLSEESMPDPARL

GQRRVWIIDPLDGTREYCEPGRDDWAVHVALAVDGAPAAAAVALPARGELFSTPAPRLAAAPPGRLKILV

SRSRAPALAERVAARLDAELVPMGSAGAKAMSALRGEAHAYLHAGGMNEWDTCAPVGVALAAGLHASRID

GSPCAFNQADVKMPDLLICRPELAETLLAAITAERMPGD

>CysQ_Porphyrobacter_cryptus |653237796|ref|WP_027443509.1| inositol phosphatase [Porphyrobacter cryptus]

MTDAELAARLAAAAGELLRGVRASGVFSGKALGKAGDATANQFLCHALRHMRPEDALLSEEEKDSPARLG

KNRVWIVDPLDGTREFGEDREDWAVHVALAIDGAAAVGAVALPGLGAGVVLRSDAPAPLPLMAACPRFVV

SRTRPAAQAEAVAAALGGTLVPMGSAGAKAMAVVRGEAEVYLHSGGQYEWDSCAPVAVAAAHGLHCSRLD

GSPLRYNQADTYIPDLLICRREWAASVLAEIARLAG

>CysQ_Sandarakinorhabdus_limnophila |550931849|ref|WP_022680261.1| MFS transporter [Sandarakinorhabdus limnophila]

MTDAELARHLAHVAGQMLLELRSGPAEGKALGAEGDRISNAFLVAELRRLRPDDAILSEEEAPDLARLQY

ARVWLVDPLDGTREYGERRDDWAVHVALSIDGRIADGAVAQPALDQTICTQHPPSLHPEAARLRIVVSRS

RPAACADALAASIGCELVPMGSAGAKTLAVLRGEADVYLHQGDMHEWDSGAPAAVAQAAGLWVSRVDGSP

LVFNQPRPLTPDILICHPGRAPALLAAIAPLL

>CysQ_Blastomonas_sp._AAP25 |930032589|ref|WP_054136388.1| inositol phosphatase [Blastomonas sp. AAP25]

MSMTDGDLAAHLAHTAGRILLDVRQSGLVSGASLGKAGDQTANQLLVHALRQQRPEDGLLSEEEKDNLER

LAHRRVWIIDPVDGTREYGEERADWAVHIGLSIDGVASIGAVALPALDVVLRSDRPQPLAPGAPIPRMVV

SRTRPAKEALAVAETLAAELLPMGSAGAKAMAIVRGEAEIYLHTGGQFEWDSCAPVAVAAAHGLHVSRAD

GTPLVYNQADTYMPDLLICRPEWTERVLMAMRALTD

**HisN orthologs used for motif identification**

>HisN_Ilumatobacter_coccineus |505253683|ref|WP_015440785.1| histidinol-phosphate phosphatase []

MTMSDTPLELAAELALALEVADAADAYTLPHFVDRDFSVDWKTNATEVTEIDRGAESLIVEALVAARPGH

GVFGEEHGLAGDHDAPWRWVIDPIDGTSGFVRGIPVWATLIALTYQGDAVMGVVSAPAIGTRWWGGVGLG

AHMRSPIVERELGVSTVDDLAEAQVSVTHSPGWDDVGRTPNLVALQQRARRSRGMGDFWQHMLVAEGSMD

VAVDAIGVAPYDLAAVKPIVEAAGGTFTDRFGEVTHQHDSAVSSNGRLHRTVIDLLA

>HisN_Trueperella_pyogenes |640530597|ref|WP_024964487.1| histidinol phosphatase []

MLKGMKPRYADDLQVALGIADRVDTITLERFRAADLRVQTKPDMTPVTDADRAAERHIRDLLRQFRSRDG

VIGEEEDNVEGLSGRRWIIDPIDGTKNYVRGVPVWATLIALEDEGEIVLGIVSAPALKQRWYAAKDLGAF

AGRSTAAARRIHVSQVGRISDASLSYSSLSGWSERNQLRGFLRLAQNSWRTRAYGDFWSYMLVAEGAVDI

ATEPELNLYDMAALVPIVTEAGGRFTALDGKEGPWGGNAIATNGFLHDEALGILNSVSDID

>HisN_Actinomyces_turicensis |493732516|ref|WP_006681775.1| histidinol-phosphatase []

MPESRYADDLKIALDLADHADKITMARFQASDLVVESKPDLTPVSDADRGVEDMIRTALATRRPDDAIYG

EERGSSGAGTRRWIIDPIDGTKNFVRNVPVWATLIALVDGSDVVVGVVSAPALGRRWWASQGGGAWEQFG

SNAPHTLHVSTINALSEASLSYSSLTGWQQVGRIDEFVDLHARVWRTRAYGDFFSYMLVATGAVDVACEP

ELELYDMAALVPIVTEAGGRFTSLSGVAGPWGANALASNGLLHDQILDHFHWPKD

>HisN_Mobiluncus_curtisii |298345522|ref|YP_003718209.1| histidinol-phosphate phosphatase [ATCC 43063]

MSSRWEDDLQLALIMADQVDALTTKRFLAPDLKVSDKPDLTPVTDADQAAEKLIRKHLSHSRSRDAVVGE

EEGSTGLASRKWVIDPIDGTSNFLRGVPVWATLIGLMEDGKMVMGVVSAPALGFRWWAAAGSGAWTGRSR

TSARQLRVSRISNLNQASFSYSLIGEWAEAHRLRGFMNLSQQVWRTRAYGDFWSYMLLAEGAVDVAAEPD

LNLWDMGALYPIVTEAGGKFTSLTGEDGVNGPGAVVTNGRLHSQVLQYIGSETD

>HisN_Actinobaculum_massiliense |494058551|ref|WP_007000636.1| histidinol-phosphate phosphatase HisN []

MNKELDVLLSILDKVDELTMDRFLASDLRVETKPDMSPVSDADKAAEELIRSELSQAFPDDFVFGEEQGG

NTDWEKGRHWIIDPIDGTKNFVRGVPVWATLVGLADSKDIILGAASAPAIGRRWYASRGEGAWVTSAGIG

AGRFHEARRLHVSRVSEVENASFSFSSLEGWRDAGKLQQMVDFQGRAWRIRGYSDFWNYLLVAEGAVDGA

AEPELDVYDMAGLVPIVEEAGGTFTGLDGKTPGPWGGGALVSNGLLHAQMQEALG

>HisN_Catenulispora_acidiphila |502435770|ref|WP_012785613.1| histidinol-phosphatase []

MTAFDDDLRLAHVLADSADAITMGRYKARDLVVESKPDMTPVSDADKAAEEAIRSALSRARPRDAMLGEE

FGETAAGATGRKWIIDPIDGTKNYVRGVPVWATLIGLMEGEEVVAGVVSAPALGRRWWAAKGGGAFTGRS

LASAQRCHVSKVAAIADASLAYSSALSWEEYGKLEAFLDLSRACWRTRAYGDFWSHMLVAEGACDMAAEP

ELSLWDMAALAVIVTEAGGMFTSVDGAPGVLGGSAAVSNGLLHEELLFYVGGQQLEG

>HisN_Alloscardovia_omnicolens |551236877|ref|WP_022857346.1| histidinol-phosphatase []

MTEFYDDLIHALEYADVADEISMNRFGALDLHIDTKPDNTPVTDADRAVERAIRNKIASHNPSDSIYGEE

LGKQESTGRRWIIDPIDGTKNFVRGVPVWATLIGLQVGEDLVASVVSAPALGMRWFAAQGAGAFMGTDMN

NARQIHVSKVSRLEDASLSLSSLSGWKERGNRDKVIDLTDQVWRLRGYGDFWQYMLVAQGAVDIAAEPEL

DLYDMAALVPIITEAGGSFTDLNGNPGPWGGCGLATNGLLHAQVLEKLQ

>HisN_Metascardovia_criceti |516878139|ref|WP_018143577.1| hypothetical protein []

MNEKIFDSPFYEDLMHALEFADIADAISLERFGALDLKVETKSDNTPVSDADRAVERAIRKAIALYYPTD

TVYGEELGKQESAGRRWILDPIDGTKNYVRGVPVWASLIALQVDDELVVSVVSAPALGMRWFAVQGAGAY

MGKNFEDARRIHVSQVSQLHDASLSLSSLSGWKDRGNRDHLIALTDEVWRVRGFGDFWQYMLVAQGAVDA

AAEPELDLYDMAALVPIVTEAGGRFTDLEGNPGPWGGCALGSNGLIHEDILERLR

>HisN_Bifidobacterium_asteroides |408501152|ref|YP_006865071.1| histidinol-phosphate phosphatase [PRL2011]

MSIEDERQTWQEDLDLAIAMADQADQVTVGYFKSPDLKVERKADNTPVTQADKEAEAVIRRVLARARPND

TIYAEELGKQESNGRRWIIDPIDGTKNYVRGVPVWATLIGLEVDHQMVVGTVSAPMLGTRWYAARGQGAY

MARQGEAPKAIHVSRVDRMEDASMSLSSLTGWKAIGRREQLLDLTDRIWRLRGFGDFWQYMLLAQGAIDL

AAEPELDLYDMGALVPIVTEAGGSFTDLAGNPGPWGGNGLASNGLLHKEALEALGD

>HisN_Mycobacterium_tuberculosis |15610273|ref|NP_217653.1| histidinol-phosphatase [H37Rv]

MSHDDLMLALALADRADELTRVRFGALDLRIDTKPDLTPVTDADRAVESDVRQTLGRDRPGDGVLGEEFG

GSTTFTGRQWIVDPIDGTKNFVRGVPVWASLIALLEDGVPSVGVVSAPALQRRWWAARGRGAFASVDGAR

PHRLSVSSVAELHSASLSFSSLSGWARPGLRERFIGLTDTVWRVRAYGDFLSYCLVAEGAVDIAAEPQVS

VWDLAALDIVVREAGGRLTSLDGVAGPHGGSAVATNGLLHDEVLTRLNAG

>HisN_Mycobacterium_smegmatis |489990395|ref|WP_003893452.1| histidinol-phosphatase []

MSTVADDLTLALELADQADALTMDRFGALDLRVETKPDLTPVTDADRGAEETLRAALAKARPADTVFGEE

FGGTTALTGRQWVIDPIDGTKNFVRGVPVWCTLIALLDDGVPRVGVVSAPALARRWWAAEGQGAFGSFNG

TTRKLSVSGVSDLSAASLSYSDLTTGWDDRRERFVELTDAVWRVRAYGDFWSYCMVAEGAVDIACEPEVK

LWDIAPLDVLIREAGGTFTSIDGADGPHGGSALATNGVLHDAVVSRLAVR

>HisN_Gordonia_namibiensis |493920454|ref|WP_006865492.1| histidinol-phosphatase []

MSADSGPKNPSTSQSYDDDLELALSLADSADVLTMDRFGAVDLRVDDKPDLTPVSDADLACETLIRERLA

ARRPADMVLGEEFGGDAALSGRQWVIDPIDGTKNFVRGVPVWATLIALLVDGVPTVGVVSAPALRRRWWA

AGGLGAHTCFDGEDARELSVSGVADLSSSSLAFSSLSGWADRGIRDRFVDLTDQVWRVRGYGDFFNYCLV

AEGAVDIAAEPEVSLWDLAPLDILVREAGGRFTALDGTPGPAGGSAVATNGLLHDEVLAALRP

>HisN_Smaragdicoccus_niigatensis |516913654|ref|WP_018163175.1| hypothetical protein []

MYSADLQLAHTLADAADAITRERFLALDLQIDSKPDLTPVSDADLAVERKLRAILETERPDDAVAGEEFG

GDQAHTGRQWVIDPIDGTKNFVRGVPVWASLIALLEDGVPVVGVVSAPALGRRWWASKDGGAFAKAGHQE

SRRITVSKVKDLDHASLSFSSLSGWRDLGLRDQLIALTDEVWRVRAYGDFLSYCFVAEGAVDLAAEPEVN

LWDLAPLDILVREAGGMFTALDGTPGPHHGNAVASNGQLHHAILARLAK

>HisN_Nocardia_farcinica |499524386|ref|WP_011211026.1| histidinol-phosphatase []

MLAAVAAHSSDLELALRLADEADAITKARFGAIDLKVDAKPDLTPVSDADLAVEESIRRLLAHARPEDAV

LGEEFGGDAEFRGRQWVIDPIDGTKNFVRGVPIWASLIALLEDGVPVVGVVSAPALARRWWAASGSGAWT

SFHPGAPRPITVSAVGELDAASLAFSSLSGWRERGLRERFLDLTDAVWRVRGYGDFFSYCLLAEGAVDIA

TEPEVSLWDLAALDILVREAGGRFTALSGQPGPHGGDAVATNGLLHEAVLSRLSRD

>HisN_Rhodococcus_opacus |491382330|ref|WP_005240212.1| histidinol-phosphatase []

MTDLHADLQLALRIADEADAITRARFGALDLSVDDKPDLTPVTDADLAVERAVRATLEAERPADAVLGEE

FGGDAQFTGRQWVVDPIDGTKNFVRGVPIWATLIALLEDGVPTVGVVSAPALARRWWAASGAGAWSTFDG

SEPTRLAVSAVDRLGSASLTFSSLSGWKELGIREQFLSLTDDVWRVRGFGDFFSYCLLAEGAVDIAAEPE

VSLWDLAPLDVLVREAGGRFTNLAGADGPHGGSALATNTLLHDEALSRLRSN

>HisN_Segniliparus_rugosus |543937463|ref|WP_021030860.1| histidinol-phosphate phosphatase HisN []

MSISTWQDDLDVALRLAEAADAITTRRFLASDLSITAKPDLTPVTEADLAVETRIREILAEVRPADAVLG

EEFGGEAAFAGRQWVVDPIDGTKNYVRGVPVWATLVALLVDGVPVVGVVSAPALHRRWWAAEGAGAFVAA

PGSAPRRAKVSEVANLHDASLSVSDWREGAFGERIRALESKVWRLRGYGDFYHYCLLAEGAVDLAAEPEV

SLWDLAALDVLVREAGGRFTSLTGQDGPHGGSALASNGRLHGMALEALGGPGA

>HisN_Segniliparus_rotundus |502903683|ref|WP_013138659.1| histidinol-phosphatase []

MSTPTPWRDDLGAALRLAEAADAITMSRFLASDLVVNAKPDLTPVTEADLAVETRIRELLAEERPEDAVL

GEEFGGTAVFAGRQWAVDPIDGTKNYVRGVPIWATLIALLVDGEPVVGVVSAPALHRRWWAASGSGAFVS

SAGGAPRRVRVSQVRDLHDASLSVSDWREGRFGESIRALEASVWRLRGYGDFYHYCLLAEGALDLAAEPE

VSLWDIAAPDVIVREAGGFFSSLEGQAGPHGGSALASNGILHDAALGAMRGAGA

>HisN_Tsukamurella_paurometabola |502892653|ref|WP_013127629.1| histidinol-phosphatase []

MPSAPSNPNPDLQLALALADAADALTMARFGALDLKVDAKPDLTPVSDADLATETLIRERLAADRPGDAV

LGEEFGGEAVFAGRQWVLDPIDGTKNFVRAVPVWSTLIALLEDGVPTVGVVSAPALRRRWFASAGAGAFA

QFDGGTPVPISVSGVLRIADASISFSDLSYFADRAARDRFLALADDAWRLRGYGDFWSYCLVAEGAVDVA

VEPEVSLWDLAAVEIVVREAGGLFTGTDGTPGPHAGSAVAANPALHPQVLARLRD

>HisN_Frankia_alni |111225362|ref|YP_716156.1| inositol monophosphatase [ACN14a]

MGTASTEDSDAVAVADDLALALSLADAADRITLSRFQAVDLHVESKPDNTPVSDADTAVESMIRERLAVA

RPGDAVLGEEEGLVGAGARRRWILDPVDGTKNFVRGVPVWGTLLGLEVDGQMVVGVASAPAMSRRWWGAR

GTGAFTRDATGDTRALKVSSVSRLGDAFLSFASVEGWRTADRLDQFLHLADQVWRTRGYGDFWSHMMVAE

GAVDLACEPEVSLWDMAALQVIVEEAGGRFTDLAGRRGPGHGTVLTTNGYLHDVALRSFND

>HisN_Blastococcus_saxobsidens |504190619|ref|WP_014377721.1| histidinol-phosphatase []

MTSGRGYSEDMRLAHVLADQADSISLDRFKAQDLTVETKPDLTPVTDADRAVEEQLRITLGRARTRDAVL

GEEFGTTGHGSRRWVLDPIDGTKNFVRGVPVWATLIALFDGDEPVVGLVSAPALNRRWWAAKDVGAWTGR

RLESATRCHVSEVARLEDASLSYSSLSGWEQRGGLDGFLDLTRSVWRTRAYGDFWSYVLLAEGAVDIACE

PEVSLWDLAALDVIVREAGGRFTDVTGAPGPAGGSALASNGKLHDAALERLGLPETVHPLTSAPPVPPS

>HisN_Modestobacter_marinus |504555441|ref|WP_014742543.1| histidinol-phosphatase []

MTSGQRGYNEDMRLAHVLADQADALSLDRFRAQDLVIDTKPDLTPVTDADRAVEEQLRSTLARARTRDAV

IGEEFGSTGRGDRRWVIDPIDGTKNFVRGVPVWATLIALLDGDVPVVGLVSAPALHRRWWAAAGTGAWTG

RRLENATRCTVSQVSTLADASLSYSSLSGWEEQGRLDAFLDLTRTVWRTRAYGDFWSYMMVAEGAVDVAC

EPEVSLWDLAALDVIVREAGGTFTDLAGTPGPAGGSAVASNGALHPDVLTALAPGAGRAPSR

>HisN_Geodermatophilaceae_bacterium |652462477|ref|WP_026857261.1| histidinol phosphatase [URHB0062]

MTSGRGFSEDMRLAHVLADQADSISLDRFKAQDLKVDTKPDLTPVTDADRAVEEMLRITLSRARTRDAVM

GEEFGVTGHGSRRWVLDPIDGTKNFVRGVPVWATLIALLDGDEPVVGLVSAPALNRRWWAAKDVGAWTGR

RLESASRCRVSDVTTLDDASLSYSSLSGWEQRGGLDGFLDLTRSVWRTRAYGDFWSYVLLAEGAVDVACE

PEVSLWDLAALDVIVREAGGRFTDLDGVPGPAGGSALATNGHLHDAALGLLRPPRPAA

>HisN_Nakamurella_lactea |656112011|ref|WP_029135519.1| histidinol phosphatase []

MTTDADDLALALRLADTADAISLPRFGATDLRVDSKPDLTPVSDADLAVERAIRELLAEQRPADSVLGEE

FGDSLPGAERRWVIDPIDGTKNFVRGVPVWATLIALMDGPDLVVGVVSAPALARRWWARSGGGAFSSFNG

APARPIRVSGVTNLSDASLSYSDYAEWAVAPEQARGFRTLLDHCWRTRAYGDFYSYVLVAEGAVDVAAEP

ELSLWDLAALVPIVTEAGGQATDVSGEPLTAASTSLAVSNGPLHPAVIETLNTIPA

>HisN_Sporichthya_polymorpha |518715142|ref|WP_019876155.1| hypothetical protein []

MSDFQDKYADDLSLALSLADAADAITTARFRALDLRIESKPDLTPVTDADRDTEQHLRDLLADARPVDAV

HGEEFGDAGSGARRWVIDPIDGTKNFVRGVPVWATLIALMDGDDVVVGVVSAPALGRRWWASAGGGAWTR

SGAAEPARCQVSAVATLADASLSYSSLSGWEERGLLPGFLDLTRACWRTRAYGDFWSYVLLAEGTVDLAA

EPEVALHDLAPLALIVSEAGGTFTDVSGRPGPDGGSAVATNGLLHSAVLAHLT

>HisN_Acidothermus_cellulolyticus |500038651|ref|WP_011719369.1| histidinol-phosphatase []

MSDPTAADLALALRLADLADEISLSRFQAMDFRVETKPDLTPVSDVDLSVEREVRRVLAADRPGDAVLGE

EFGGEPVDGRVWVIDPIDATKNFVRGVPIWATLIALLDAGEPVIGVVSAPALASRWWAGRGLGSWTARLG

AAPRRNQVSAVRNLSDASLSYSGLGGWGTRVSDFLNLTKAVWRTRAYGDFFSHVLVAEGAVDISAEPEVS

LWDTAALVVIVTEAGGRVTGVDGGPSPAASSILCTNAWLHDAALSHLAGSARRGG

>HisN_Actinomadura_flavalba |648622474|ref|WP_026314225.1| histidinol phosphatase []

MAGYSDDLRLAHVLADGADDITTKRFRALDLDIETKPDLTPVSDADRSVEEQIRGTLKRARPRDAVLGEE

YGRTGYGQRCWIIDPIDATKNFVRGVPVWATLIALMENDEVVVGVVSAPALNRRWWAARGNGAWTGRSLT

RASRIQVSSVTDLANASLSFSSLSGWEEQERLDRFLDLTRSVWRTRAFGDFWSHMMVAEGVVDISAEPEV

SLWDLAALQVIVEEAGGMFTDLSGVPGPDGGSVVVTNGHLHADVLRTLGGGQLSLRV

>HisN_Microbispora_rosea |663735249|ref|WP_030511419.1| histidinol phosphatase []

MTEYRDDLHLAHLLADAADDITMRRFKAVDLKVDTKPDLTPVSDADRAVEEAIRGTLRRARPRDAVVGEE

FGMTGYGMRSWIVDPIDGTKNYVRGVPVWATLIALMEHGKVVVGVVSAPALGRRWWAAKESGAWSGRSLS

KATRCEVSSVGRLDDASFSFSDLDEWEKAGRLDPFLDLTRECWRTRGYGDFWSHMMVAEGAVDISAEPEL

SPWDMAALTIIVEEAGGSCTSLDGAAPLDGGNLVCTNGLLHGEVLKRLSPR

>HisN_Spirillospora_albida |663124111|ref|WP_030165002.1| histidinol phosphatase []

MAGYSDDLRLAHVLADGADDITTKRFRALDLDIETKPDLTPVSDADRSVEEQIRGTLSRARPRDAVIGEE

YGRTGYGNRCWIIDPIDATKNFVRGVPVWATLIALMENDQVVVGVVSAPALHRRWWAARGGGAWTGRSLT

RATRMSVSSVAEMPDASLSFSSLGGWEEGGRLERFLDLTREVWRTRAFGDFWSHMMVAEGVIDISAEPEV

SLWDLAALQVIVEEAGGMFTDLSGVPGPDGGSVVCTNGRLHADVLRTLGGGQLSLRID

>HisN_Nocardiopsis_kunsanensis |516143524|ref|WP_017574104.1| histidinol-phosphatase []

MASFDDDLRLAHVLADAADDIALKRFRALDLVVDTKPDLTPVTEADRRVEETLRSVLDRARPRDAVIGEE

YGQSGNSNRVWVIDPIDGTKNYVRGVPVWATLIALLEGDRPVVGVVSAPALHRRWWASQGSGTWTGRSLS

KASRCQVSQVTELSDASLSFSSLTGWEEQGRLESFLGLTRSVWRTRAYGDFWSHVMVAEGTVDIAAEPEL

SVWDAAPLPILLEEAGGRATNLRGGDFEDGGPLVCTNGALHDRTLTWLNGGPTPLRPT

>HisN_Streptosporangium_roseum |502658557|ref|WP_012894707.1| histidinol-phosphatase []

MTGYNDDLRLAHVMADAADDLTMRRFKAADLRIDTKPDLTPVSDADRAVEEAIRGTLSRARPRDAVIGEE

FGKTGYGARSWVIDPIDGTKNYVRGVPVWATLIALMDQGRVVVGLVSAPALGRRWWAARDSGAWTGKSLT

KATRCQVSSVTRLEDASFSYSSFGGWEEAGKLNEFLDLNRSVWRSRAYGDFWSHMLVAEGAVDLSAEPEL

SPWDIAALTVIVEEAGGIWTDLSGVPGLDGGSLVCTNGSLHSEVLKRLGGGPLTLPV

>HisN_Thermobifida_fusca |499610255|ref|WP_011290989.1| histidinol-phosphatase []

MAAFDDDLRLAHVLADAADDISLRLFRSLDLKVDTKPDLTPVTEADRAVEETVRSILSRARPRDAVVGEE

YGTSGNSARRWVVDPIDGTKNYVRGVPVWATLIALLEGDQPVVGVVSAPALNRRWWASKGGGAWSGRSLA

KATRCRVSAVSRLEDASLSFSSLTGWEEQGRLDYFLDLTRSVWRTRAYGDFWSHVMVAEGAVDLSMEPEL

SLWDAAPLPLILEEAGGRATSLNGGPFTDGGALVCSNGLLHDAVLTQLNGGPIPLRRT

>HisN_Streptomyces_purpeofuscus |663397116|ref|WP_030392898.1| histidinol phosphatase []

MADYHDDLRLAHVLADSADSVTLERFKALDLRIETKPDLTPVSDADKAAEELVRSILQRARPRDAVLGEE

FGLQGTGPRRWVIDPIDGTKNYIRGVPVWATLIALLEDGPDGVERPVVGIVSAPALNRRWWAAKGLGAYA

GRSLAKASRIHVSGVSRIEDASFSYSSLSGWEERGRLEPFLDLTRACWRTRAYGDFWSYMMVAEGAVDIA

AEPELSLWDMAANSVIVEEAGGRFTGLDGVPGPGGADAVASNGLLHQEALDRLSV

>HisN_Streptomyces_coelicolor |21223576|ref|NP_629355.1| monophosphatase [A3(2)]

MPDYLDDLRLAHVLADAADAATMDRFKALDLKVETKPDMTPVSEADKAAEELIRGHLSRARPRDSVHGEE

FGVAGTGPRRWVIDPIDGTKNYVRGVPVWATLIALMEAKEGGYQPVVGLVSAPALGRRWWAVEDHGAFTG

RSLTSAHRLHVSQVSTLSDASFAYSSLSGWEEQGRLDGFLDLTREVWRTRAYGDFWPYMMVAEGSVDLCA

EPELSLWDMAANAIIVTEAGGTFTGLDGRPGPHSGNAAASNGRLHDELLGYLNQRY

>HisN_Kitasatospora_setae |503903996|ref|WP_014137990.1| histidinol-phosphatase []

MADYHDDLRLAHVLADSADSITMERFKALDLKIETKPDLTPVSDADKAAEELVRSVLQRARPRDAVLGEE

YGLQGSGPRRWVIDPIDGTKNYVRGVPVWATLVALLEEQPDGEHVPVVGIVSAPALGRRWWAAKGLGAFA

GRSLAKAQRIHVSKVSRLADASLSFSSLSGWEERARLDAFLDLTRVCWRTRAFGDFWSYMMVAEGAVDIA

AEPELSLWDMAAPCVIVQEAGGRFTGLDGIDGPTGADAVATNGILHEAALRSLSV

>HisN_Streptacidiphilus_jeojiense |663220715|ref|WP_030257522.1| histidinol phosphatase []

MADYHDDLRLAHVLADHADSVTLERFKALDLKIDTKPDLTPVSDADKAVEDLLRNDLKRARPRDGVMGEE

FGSTGHGPRRWVIDPIDGTKNYIRGVPVWATLIALMELGEGGERPVVGMVSAPALNRRWWAAKGTGAFTG

RSLTKASRIQVSDVSGLEDASFAYSSLTGWEERGRIDAFLELSRTVWRTRAYGDFWPYMMVAEGSVDICA

EPELSLWDMAATCVIVEEAGGRFTGLDGVPGVHSGNAAASNGLLHGELLQRLG

>HisN_Rubrobacter_radiotolerans |627778235|gb|AHY47456.1| Archaeal fructose-16-bisphosphatase and related enzymes of inositol monophosphatase family []

MQPEVSPEKLREFRDFAAETAYAAGRLTLGYFRGGVRAEFKGDDTPVTVADREAEALIRSRIERRYPDHA

LVGEEHGQTGPEEATCRWYVDPIDGTKAFVRGVPLYAVLVALEVSGEVCVGAAYFPALDEMLVAAAGLGC

HLNGRPARVSGVRELRKSVVCFTETATFFQEGRKASFDAVSEAVYSFRGWSDAYGHALVATGRAEAAVEA

AMNPWDCAPFLPILREAGGYFGDWSGEETVHRNEAVSANPHVKEELLALLGRTDGRPADAAARRGPG

>HisN_Amycolatopsis_taiwanensis |654471898|ref|WP_027942124.1| histidinol phosphatase []

MPEDLTLARHLADIADAITTARFRARDLQVSSKPDRTPVTDADTAVEDAIRERLATERPADAVVGEERGG

TSWSAGRAWVVDPIDGTKNFLRGVPVWATLIALVEDGTPVVGVISAPALGRRWWAARGEGAWSSDASGER

SISVSGVSALQDAYLSTTHLGSWTEHHSREAYLALTDACWENRAFGDFWSHCLVAEGAIDLAAEAIVNPW

DVAAVQVLVTEAGGRFSDLSGKPRFDGGSAVSSNGLLHDEALQILQR

>HisN_Saccharomonospora_marina |496443724|ref|WP_009152569.1| histidinol-phosphatase []

MTVSRYSSDLTLAGELADTADAITTARFRALDLAVGSKPDRTPVTDADTAVEDAVRDLLARRRPGDDVAG

EERGGAVGDQGRVWVLDPIDGTKNFLRGTPVWATLIALVENGTPVVGMISAPLLGRRWWAGEGGGAWLRD

SSGERRLAVSRVSALADAYVSTTDLGSWVRHHSRAAYLALVDACWESRAFGDFWQHCLVAEGAIDVAVEP

IVNPWDVAAVRVIVTEAGGRFTDLAGRDRIEGGSALSTNGLLHDDTLATIAGARPG

>HisN_Pseudonocardia_asaccharolytica |655581615|ref|WP_028930408.1| histidinol phosphatase []

MDADLSLALRLADIADATTLPRFRATDLRVTRKPDRTPVTDADTATEDALRAELATRRPGDGVLGEERGG

DVPESGRGWLLDPIDGTKNFSRGLPVWASLIALTMHGKPVIGVASAPGLGRRWWASAGAGAWTTDTPGAE

PRRLAVSGVAELGDVYVSTTDPDVFRKHGTFDSWLALTDRCWETRAFGDFWQHCLVAEGLLDLAVEPEAS

SWDLAALQVIIEEAGGRFSDLTGAATFAGGDAMSSNGLVHDAALSCFGP

>HisN_Thermocrispum_municipale |655468237|ref|WP_028851171.1| histidinol phosphatase []

MTRAYTDDIDLAKRLADVADAITVPRYTSRDLTVRTKPDRSPVTDADLAVEEAIRGVLATERPQDAIAGE

EGGGSIGAGRTWVLDPIDGTKNFLRGVPVWATLIALVDDGKPVVGMISAPLLGRRWWADAGGGAWVADPS

GQRRLRVSAVAELSDAYLSTTNLNTWDEYHSKQAYLDLVAACWENRAMGDFWSHCLVAEGVIDIAAEPIV

NPWDVAAAQILVTEAGGRFTDLTGAERFDGGNALSTNGVLHDAALRVLSSGLVDR

>HisN_Kribbella_catacumbae |521057560|ref|WP_020389511.1| histidinol-phosphatase []

MPSHTDDLRLAHILADDADSTTMDRYKALDLHVATKPDLTPVSESDRKVEDVMRKTLARARPRDAFVGEE

EGTTGWGVRRWVVDPIDGTKNYIRGVPVWATLISLMIEDQVVVGVVSAPALGRRWWASYGDGAWTGRALM

SAQPCRVSDVSSIDDASLSYSSLKGWEKLGKLDQFADLMESAWRTRAYGDFWSYMLVAEGAVDIAAEPEL

NLYDMAALAIIVDEAGGKFTSLDGVPGPNGANAVATNGRLHAEVLERLQR

>HisN_Nocardioides_sp._URHA0020 |655230029|ref|WP_028642498.1| histidinol phosphatase []

MPKNPTDYTDDLRLAHVLADDADSISESRFKALDLHVMSKPDLTPVTDADQAVEESIRRTLSRARSRDAI

TGEEQGSTGHSQRRWIIDPIDGTKNFVRGVPVWATLISLAVDDEVVLGVVSAPLLQRRWWASKGNGAWTG

RSLLKAVRCQVSDVRRLEDASLSYSSLSGWDERDRLDDFESLMRRCWRTRAYGDFWSYMLLAEGAVDLAA

EPELEVYDMAALDIIVREAGGRFTSLDGTDGPFGGNALASNGHLHEAALSFLGALPDDDDDPDSRRSGPG

SVSDLRSHRRD

>HisN_Aestuariimicrobium_kwangyangense |651238868|ref|WP_026378025.1| histidinol phosphatase []

MTRMAGDFTDDLRLAHLLADNADSITMSRFKARDLEVSMKPNMTHVTDADTAVEQGIRATLKKARPRDAV

HGEEGDDTGWGPRRWIIDPIDGTANFVRGVPVWATLIALSVDGEIVASVVSAPALARRWWASKGGGAFSG

TTLLRGAPIHVSKVSRVEDSSLSYASIDGWVRTGRGQQFVDLMRECWRTRAFGDFWSYMLVAEGAVDIAT

EPELELYDMAALDVIVREAGGRFTNVDGADGVGGPGALATNGLLHDEVLARLAEDAD

>HisN_Aeromicrobium_marinum |494137507|ref|WP_007077263.1| histidinol-phosphatase []

MAHSYADDLRLAHVLADSADNLSMDRFGAIDLEVSTKPDLSWVTESDQAVEESIRRTLRSARTRDAVFGE

EMGAHEGSTGSAERRWIIDPIDGTSNFVRGVPVWATLIALEEAGEIVAGLVSAPALGRRWWAHKGSGAYT

GKSVMNAREIRVSRVSDLENASFSFSSTESWEDIGKAEAFTALTRRCWRTRAYGDFWSYMLLAEGAVDIA

AEPELKLWDMAALDVIVREAGGRFTSLAGDDGPWGANALATNGRLHDAAMAYLGHFPDDGPHDETWADEP

EAADLPATPRNNVRSLDFGRPDAFDQ

>HisN_Propionibacterium_propionicum |504659330|ref|WP_014846432.1| histidinol-phosphatase []

MVASKELTDDVRLAHVMADDADSISMSRFKAQDLRIKTKPDSTPVTEADTAVEESIRRTLSRTRPRDAVH

GEEMQDTGESNRRWIIDPIDGTANYLRGVPVWATLIALEIDGQIAASVVSAPALGRRWWASLEGGAYTGR

SILNATQIHTSRVSQLSDASLSYASINGWVESGRGQGFVDLMRECWRSRGYGDFWSYMLVAEGAVDIACE

PELALYDMAALDIIVREAGGRFTSIDGEPGPYHGNALATNGLLHDIVLERLRPSEAG

>HisN_Microlunatus_phosphovorus |503629902|ref|WP_013863978.1| histidinol-phosphatase []

MSGPTSPSTSSASTASAPTDPAFRNLTDDLRLAHLLADDADSITTNRFKALDLHVVSKPDLTPVTDADTA

VEEALRRTLSRARPRDAVHGEELPDTGWGPRRWVIDPIDGTKNFVRGVPVWATLIALMINDEVAVGVVSA

PALGRRWWASLGGGAYAGKSLMSATPCRVSDVATIEDSSLSYSSVSGWVESGRGQPFVDLMRRCWRTRAF

GDFWSYMLLAEGAVDIACEPELALHDMAACSIVVTEAGGQFTDLTGVPGPRGGNAVATNGRLHQAVLEQL

ASPVEDHDAPDADD

>HisN_Pilimelia_anulata |517878345|ref|WP_019048553.1| histidinol-phosphatase []

MAAYSADLDLALRLADEADAISRVRFGAVDLKVDAKPDLTPVSDADLAVEQAIRRVLGAERPGDAVLGEE

FGGDAEFTGRQWVVDPIDGTKNFVRGVPVWASLIALLVDGVPVVGVVSAPALGRRWWAAAGSGAWTQVHP

GAPKPISVSAVGELDAASLAFSSLSGWHERGLRETFVDLTDAVWRVRGYGDFFNYCLVAEGAVDIAAEPE

VSLWDLAALDILVREAGGTFTALDGQPGPHAGSAVATNGLLHDEVIGRLRG

>HisN_Actinoplanes_utahensis |759828803|ref|WP_043524633.1| histidinol phosphatase [Actinoplanes utahensis]

MAGYADDLALAHILADSADAISMARFRALDLKVEEKPDLTPVSDADTAVEKAIRATLARARPRDGVLGEE

FGRTTATAGPGNRYWVIDPIDGTKNFVRGVPIWATLIALMEGDTPVAGLVSAPALGRRWWAGRGLGAFAG

RNQHSATRINVSAVRKLTDASFCYASLNGWAENGRLEQMMDILLGVWRSRAYGDFYGYMLLAEGALDAMA

EPELSLWDMAALIPIVTEAGGKITDLDGRPTADKSSVVGTNGLLHESVLTALARRA

>HisN_Hamadaea_tsunoensis |653095636|ref|WP_027345846.1| histidinol phosphatase []

MARYADDLALAHVLADTADSLAMSRFRALDLHVSSKPDLTPVSDADTAVEKALRSTLARTRPRDGVLGEE

YGSTGATGSRQWVIDPIDGTKNFIRGVPVWGTLISLMEGDEPVVGLVSAPALGRRWWAARGLGAFAGRHQ

AAAQPIRVSEVARLADASFCYSSLNSWEQTGRLASMLEIMRKCWRSRAYGDFYGYMLLAEGALDVMVEPE

LSLWDIAALIPIVTEAGGTFTDLSGRATPGGGSAIATNGKLHSDILARLTPGGLRAL

>HisN_Longispora_albida |517162588|ref|WP_018351406.1| hypothetical protein []

MTRYTDDLGLAHLLADTADAISLSRFRALDLRVDAKPDLSPVTDADTAVEKAVRSTLARSRPRDGVLGEE

FGSTPAAAGPGNRQWVIDPIDGTKNFVRGVPIWATLISLLEGGEPVLGLVSAPALGRRWWAARGHGAFAG

KHASNATRIHVSEVGKLSDASFCYSSLGGWEEAGKLDAMLDLMRGSWRSRAYGDFYGHMLLAEGAVDAMV

EAELALWDMAALIPIITEAGGRCTNRAGRPLNHGDGVIMSNGQLHEVLLDHLGD

>HisN_Arthrobacter_sp._35W |651503090|ref|WP_026555458.1| histidinol phosphatase []

MTHEPQGYNDDLRLAHVLADAVDSLTMARFKALDLAVETKPDLTPVTDADKAAEESIRSQLSRVRPRDAV

LGEEFGSSGHGSRRWIIDPIDGTKNFVRGVPVWATLIALVDEGQPVVGLVSAPALGKRWWAAKGTGAYTG

KSLAAASRLHVSNVARLSDASLSYSSLTGWKERGNRDEFIALTDDVWRTRAYGDFWSYCLVAEGAVDIAC

EPELNLYDMAALVPIVVEAGGRFSSLEGEDGPFGGNALATNGILHAEVLNRLNPGLDDLL

>HisN_Curtobacterium_sp._S6 |662047722|ref|WP_030014747.1| histidinol phosphatase []

MTSMSPYNDDLRLAHVIADAVDRYTLGRFQAQDFTVDTKPDLTPVTDADREAEQLIRSHLSRARTRDAVL

GEEFGATGSSPRQWVVDPIDGTKNFVRGVPVWATLIALLDQGKPVVGLVSAPALQRRWWAAEGTGAFTGR

SLAKASRLNVSRVSSLHDASLSFSSLSGWKAAGLRERFLDLTDDVWRVRAFGDFWSYCLLAEGAVDIACE

PELALHDMAALVPIVTEAGGRFTSLEGEDGPHGGNAVATNGALHRKVLEKLAPLSEAPEHDQTDTTIGAS

>HisN_Renibacterium_salmoninarum |501201780|ref|WP_012244798.1| histidinol-phosphatase []

MNFAPQGYNDDLRLAHVIADSVDAQTMDRFKALDLKIETKPDLTPVTDADKSAEEAIRSQLSRSRPRDAI

LGEEFGSSGHGSRRWIIDPIDGTKNFVRGVPVWATLICLVDEGVPVVGLVSAPALGKRWWASKGTGAFTG

KSLAAASRLRVSNVSKLADASLSYSSLKGWRERGNLSEFTTLMDSTWRSRAYGDFWSYCLVAEGAVDIAC

EPELNLHDMAALVPIVTEAGGRFTSLEGTEGPFGGNALATNGILHSEVLRLLNPQLDPLI

>HisN_Acaricomes_phytoseiuli |516861246|ref|WP_018134763.1| histidinol-phosphatase []

MVHAPQGYNDDLRLAHVLADAVDSQTMSRFKALDLHVETKPDMTPVTDADRSAEEAIRSQLSRSRPRDAV

LGEEFGSSGHGTRRWIIDPIDGTKNFIRGVPVWATLICLVDEGVPVLGVVSAPALGKRWWAAQGTGAYTG

KSLASAQRIRVSNVSRINDASLSYSSLSGWREHGSLDDFLRLMDATWRTRAYGDFWSYCLVAEGAVDLAC

EPELNLYDMAALVPIVTEAGGRFTSLEGADGPFGGNALASNSILHSEALHLLNPKLDPLLDPLR

>HisN_Kocuria_rhizophila |501366440|ref|WP_012398006.1| histidinol-phosphatase []

MRAPSNHTDDLRLAHILADSVDGLTMKRFKSQDLTVETKPDLTPVTDADREAEQVIRSQLGRVRNRDAVI

GEEFGTTGSGGRQWVVDPIDGTKNYVRGVPVWATLIGLVEDGEVVLGVVSAPALNRRWWAATGSGAFTGR

SLSQAQRISVSSVSTLEDASLSYSSLSGWRELGIRDRFVDLTDRVWRTRAYGDFWSYCLVAEGAVDLACE

PELNLYDMAALVPIVTEAGGRFTSLAGQDGPFGGNALASNGALHDAALDALGTREA

>HisN_Actinotalea_ferrariae |601041251|gb|EYR62238.1| histidinol phosphatase [CF5-4]

MTIRSGYDDDLRLAHVIADQVDGLTTSRFKALDLRVETKPDTTPVTDADRQAEEIVRSQLARTRPRDAVV

GEEMPTTGHGPRQWVIDPIDGTKNFVRGVPVWATLIALVDAGRPVLGVVSAPALGRRWWAAEGSGAWTGR

SLASATRLQVSQVSRLEDASLSYSSLSGWEEQGRLEGFLDLTRATWRTRAYGDFWSYVLVAEGAVDIAAE

PELALHDMAALVPIVTEAGGRFTSLSGVPGPFGGSALATNGVLHDQVRAFLDPFGE

>HisN_Micrococcus_luteus |612084590|gb|EZP43314.1| Histidinol-phosphate phosphatase HisN []

MERPENPATRGRDLTEDLRLAHMLADNVDSITMSRFKAQDLEVSTKPDLTPVTDADRAAEESIRSTLSRA

RARDGIVGEEFGGSLSRSGRQWVVDPIDGTKNFVRGVPVWATLIALLIDGEPVAGVVSAPALHRRWWAAA

GQGAFAGTSLTRAQRITVSGVDRVEDASLSFSSIEGWRERGSIRAFLQLTSDVWRVRGFGDFWSYMLVAE

GAVDIAAEPELELHDMAALVPIVREAGGRFTSLDGEDGPFGGHALATNGLLHDDVLARLRTGDETEDGA

>HisN_Oerskovia_turbata |663108879|ref|WP_030149995.1| histidinol phosphatase []

MPTYDDDLRLAHVIADQVDSHTMSRFKALDLSVESKPDNTPVSDADRAAEEIIRSHLSRARGRDAIVGEE

FGETGHGARRWIVDPIDGTKNFVRGVPVWATLIALADGDEIVVGLVSAPALGRRWWAAKGTGAWSGRSLA

AASRLRVSQVGRLDDASLSYSSLSGWEERGKLDAFLDLTRETWRTRAYGDFWSYMLVAEGAVDAAAEPEL

ELYDMAALVPIVTEAGGTFTSLDGSPGPWGGNAVATNGLLHDEILGRLS

>HisN_Georgenia_sp._SUBG003 |660685604|gb|KEP23195.1| histidinol phosphatase []

MTVQGASRFTDDLRLAHVVADQVDGQTMARFGAQDLHVETKPDLTPVSDADRSAEEIVRAQLARTRPRDS

VLGEEFGTTGHSQRQWVVDPIDGTKNFVRGVPVWATLIGLVEDGEVVLGVVSAPALNRRWWAAKGAGAWT

GRSLSAARQIHVSGVSDLADASLSYSSLDGWAERGSLRSFLGLAQQVWRTRAYGDFWSYMLVAEGAVDIA

CEPELELYDMAALVPIVTEAGGRFTSLDGADGPWGGNAVVTNSLLHDAVLAQLSSELD

>HisN_Janibacter_hoylei |495203156|ref|WP_007927938.1| histidinol-phosphatase []

MASSPYADDLRLAHVLADQVERITMSRFQADDLVVESKPDLTPVSDADRSCEEAIRAQLARSRGRDSILG

EEFGSTGESQRRWIIDPIDGTKNFVRGVPVWATLIGLVDGEDCVVGLVAAPALGRRWWAAKGAGAWSGRS

LAQAKQIGVSRVGRLGDASLSYSSLDGWREGGRGRSFLNLTSDMWRTRAYGDFWSYMLVAEGAVDVAAEP

ELEVYDMAALVAIVEEAGGRFTSLEGTPGPWGGNAVATNGLLHDEVLARLAPDA

>HisN_Citricoccus_sp._CH26A |497830137|ref|WP_010144293.1| histidinol-phosphatase []

MSDAPKNYTDDLRLAHIMADSVDSQTMSRFRAQDLRIDTKPDLTPVTDADRAAEELIRGNLSRARPRDAV

LGEEFGETGHGPRRWVIDPIDGTKNFVRGVPVWATLIALIDDGEPVVGVVSAPALNRRWWAAKGTGAFMG

KSLASATRIHASNVCRIEDASLSYSSLEGWRERGNIRDFLELTSTVWRTRAYGDFWSYMMVAEGAVDIAC

EPELELYDMAALVPIVTEAGGRFTSLEGEDGPFGGNALATNGLLHPDALEALNPDLIGQTQA

>HisN_Intrasporangium_oryzae |586964470|gb|EWT00496.1| histidinol phosphatase [NRRL B-24470]

MTSYDDDLRLAHVLADAVERVTIARFRAADLVVESKPDLTPVTDADRAAEELVRSQLKRTRPRDAVEGEE

FDTTGHGPRRWIIDPIDGTKNFVRGVPVWATLIGLVDDGKPVLGIVSAPALQRRWWAATGSGAWTGRSLS

SARRIAVSKVASLTDASLSYSSLSGWRDAGRQDDFLDLMDSCWRTRAYGDFWSYMLVAEGAVDIAAEPEL

AVHDMAALVTIVQEAGGRFTGLDGRDGCWSGNAVATNGLLHDEVLRRIGTVEG

>HisN_Brevibacterium_sp._VCM10 |647371407|ref|WP_025776944.1| histidinol phosphatase []

MNDLDLAIELADLADAISHPHFRAQDFAVETKPDLTPVTECDRAVEKAIMDRLAEVRPDDSVLGEEFGAH

GSSSRRWIIDPIDGTKNFVRGVPVWATLISLYDDDRPLLGVVSAPALGRRWWAESGLGAYATELGSAAER

ISVSSVDALDDASLSYSSLSGWKELGILDEFLGLCDSLWRTRGYGDFYSYMLLAEGAVDLACEPELALYD

MGAIVPIVLEAGGTFTNTAGVPGPFGGNAVASNSRLHEAALDRLGRVAPAQVS

>HisN_Cellulomonas_sp._KRMCY2 |635640802|ref|WP_024287695.1| histidinol phosphatase []

MTVRSGYDDDLRLAHVIADQVDSMTMSRFRAQDLVVETKPDTTPVTDADRYAEEIVRSQLARTRPRDAVM

GEELPDTGHGPRQWVIDPIDGTKNFVRGVPVWATLIALIDAGQVVLGLVSAPALGRRWWAANGSGAWTGK

SLAAASRLQVSQVDRLEDASLSYSSLSGWEDQGRLDVFLDLTRSVWRTRGYGDFWSYVLVAEGAVDIAAE

PELALHDMAALVPIVTEAGGTFTSVSGVPGPFGGSALVTNGLLHDQVLALLAPTVEQ

>HisN_Brachybacterium_paraconglomeratum |498235468|ref|WP_010549624.1| histidinol-phosphatase []

MSSRYADDLRLAHVLADAVDQLTTSRFKAQDLEISTKPDLTEVTDADRAAEQLVRSQLARARSRDQVIGE

EFGSTGASPRQWVIDPIDGTSNFVRGVPVWGTLIGLIEDGRPVVGLVSAPALGRRWWGGEGAGAWTGSRL

SSATRLQVSTVSTLEEASLSYSSLHGWADHELLPEMLNLMQRFWRTRAYGDFWSYMLVAEGAVDVACEPE

LKLHDMAALVPIVTEAGGRFTSLDGEPGPFGGNAVATNSLLHEEVLEALVARER

>HisN_Paraoerskovia_marina |656321161|ref|WP_029253159.1| histidinol phosphatase []

MPSYDDDLRLAHVIADQVDSHTLARFKALDLSVETKPDNTPVTDADRAAEEMIRSTLSRARGRDSILGEE

FGSAGHGSRRWVVDPIDGTKNFVRGVPVWATLIALVDEGEPVVGLVSAPALGRRWWAAQGSGAWTGRSLP

KATRIQVSGVKELGDASLSYASLGGWEEAGKLDALVDLTRDCWRTRAYGDFWSYMLVAEGAVDIATEPEL

ELYDMAALVPIVTEAGGRFTSLDGTPGPFGGNAVATNGLLHDAVLERLG

>HisN_Phycicoccus_jejuensis |663752104|ref|WP_030527740.1| histidinol phosphatase []

MSRVPGYDDDLRLAHVLADAVERLTMERFRAEDLHVETKPDLTLVSDADRAAEELLRAQLKRTRPRDAVI

GEEMPDTGASPRQWVVDPIDGTHNFVRGVPVWATLISLVDHGVPVVGLVAAPALGRRWWASQGTGAWSGR

SLTSARRLRVSGVSTLDDASLSISSTTSWEAVGRLDPMLDLARACWRERAYGDFWSYMLVAEGAVDIAAE

PELSTYDMAALVPVVTEAGGRFTSLDGVDGPFGGNAVATNGLLHDEVLARLSR

>HisN_Sanguibacter_keddieii |502629320|ref|WP_012865964.1| histidinol-phosphatase []

MIARPHYDDDLRLALVIADQVDAQTMARFKALDLHVESKPDHTPVTDADRSAEEIIRGQLSRSRGRDAVL

GEEFGATGHGSRRWIVDPIDGTKNFVRGVPVWATLIALAEGDEIVVGVVSAPALGRRWWAAQGTGAWTGR

SLSSATRLQVSGVRDIADASMSYSSLSGWEERGRLPHFLDLSRQTWRTRAYGDFWSYMLVAEGAVDIAVE

PELELYDMAALVPIVTEAGGRFTSLAGVDGPFGGNAAVTNGHLHDHVLATVGFGAH

>HisN_Cellulosimicrobium_cellulans |640254256|ref|WP_024839334.1| histidinol phosphatase []

MTPPSTRGYDDDLRLAHVIADQVDAHTLSRFKALDLRVESKPDSTPVSDADRTAEEIIRAQLARARGRDA

IVGEEYGETGHGSRRWIVDPIDGTKNFVRGVPVWATLIALADGDEVVVGLVSAPALGRRWWASKGTGAWT

GKSLAAATRLQVSGVSSWSDASFSYASLDGWEERGRLEPFLDLLRGAWRTRGYGDFWSYMLVAEGAVDAA

AEPELELYDMAALVPIVTEAGGRFTSLDGADGPWGGNAVATNGLLHDEVLDRLG

>HisN_Yaniella_halotolerans |551250970|ref|WP_022871247.1| histidinol-phosphatase []

MQLSPPRQGYREDLNLALVIADAVDGLTMDRFQSVDLEVTTKPDLSPVSDADRAAEELIRQQLKRARPRD

AVTGEEFGTDGASKRRWIIDPIDGTKNFVRGVPVWATLIALVEDDRPVVGVVSAPALSRRWWAYEDGGAY

TGKSLSNAKRIHTSDVTQLEDASMSFSSLEGWRDRGNISEFLQLTSDIWRVRAFGDFWSYMLVAEGSVDI

ACEPELELYDMAALVPIVTEAGGTFTSLDGEPGPWGGHGLATNTHLHADVLERLNPELRGQSRA

>HisN_Ruania_albidiflava |653035028|ref|WP_027286716.1| histidinol phosphatase []

MRLAHVIADQVDAQTMARFKAIDLHVETKPDTSPVTDADRSAEEIVRAQLARTRPRDAVLGEEFGTTGHG

PRQWVIDPIDGTKNFVRGVPVWATLIALIDDGTPVLGVVSAPALNRRWWAATGSGAWTGRSLSAATRMHV

SGVRELEDASLSYSSLAGWEDADLLDEFLSLEASVWRSRAYGDFWSYMLVAEGAVDIACEPELELYDMAA

LVPVVTEAGGRFTSLDGQDGPFGGNAVVSNSHLHDLVLAQLHRA

>HisN_Beutenbergia_cavernae |506362042|ref|WP_015881761.1| histidinol-phosphatase []

MTTGPTRLGAARPRYDDDLRFAHVIADQVDAHTLSRFRALDLEVTTKPDDTPVTDADRSAEEMIRGQLGR

ARPRDAVLGEEFGSTGRGQRQWIVDPIDGTKNYLRGVPVWATLIALVDDGEPVLGLVSAPALDRRWWAAL

GSGAWTGRSLSKAQRIQVSGVPDVADASLSYSELGEWHAAGLLPEFLELDDACWRTRAYGDFWSYMLVAE

GAVDIAAEPELAVHDMAALAPIVTEAGGSFTSLAGEDGPFGGSAVATNGLLHADVLRRLTPRPR

>HisN_Austwickia_chelonae |493549377|ref|WP_006503072.1| histidinol-phosphatase []

MPTYDDDLRLAHVLADAAERVTMERFKAADLHVDTKPDLTPVSDADRAAEELIRNQLGRTRPRDSVLGEE

FGETGNSPRRWIIDPIDGTKNFVRGVPVWATLIALVDGDDPVVGLVAAPALNRRWWAAKGTGARTGRSLF

SSTPLAVSGVQRVEDASLSYSSLTGWRTSGRQEGFLGLAEDCWRTRAYGDFWSYMLVAEGACDIACEPEL

ELYDMAALAPIVTEAGGLFTSLEGIPGPWGPGAVASNGPLHDEVLARLSVPAS

>HisN_Promicromonospora_sukumoe |518860598|ref|WP_020016488.1| histidinol-phosphatase []

MPVAAVTKDNYADDLRLAHVIADQVDSITMSRFRALDLKVETKPDLTPVSDADRAAEDFIRGQLARARTR

DAIVGEEYGSAGSGARRWIVDPIDGTKNFVRGVPVWATLIALADGDEVVVGLVSAPALGRRWWAAKGSGA

WTGKSLASASRMSVSGISRVADASFAYSSLSGWEERGKLDGFLDLTRACTRTRGFGDFWSYMLVADGAVD

IAAEPELEVYDMAALVPIVTEAGGTFTSLDGKPGPWGGNAVATNGKLHEEALDFLS

>HisN_Knoellia_sinensis |700173994|gb|KGN34941.1| histidinol-phosphatase [KCTC 19936]

MTDVPTYDDDLRLAHVLADAVERLTVSRFRAEDLEVETKPDLTLVSDADKAAEELVRSQLKRTRPRDAVI

GEEMEPTGHGPRQWVIDPIDGTHNFVRGVPVWGTLIGLIDAGQPVLGLVAAPALNRRWWAAQGSGAWAGR

SLTSAKRISVSKVTKLEDASFSYSSATSWEAVGRQEAVLDLARSCWRERAYGDFWSYMLLAEGAVDIAAE

PELNLHDMVALAPIVTEAGGRFTSLKGEDGPFGGNAVATNGLLHDEVLARIGL

>HisN_Microbacterium_sp._KROCY2 |635643922|ref|WP_024290813.1| histidinol phosphatase []

MRPHGSYTDDLRLAHVLADTVDSLTMARFRSQELQVETKPDLTPVTDADRDAEQLIRAQLARVRNRDSVL

GEEFGTTGSGSRQWVVDPIDGTKNFVRGVPVWATLIAMIDDGVPVVGLVSAPALQRRWWAGAGTGAWAGR

SLAKAERLSVSSVDRLEDASLSYSSLSGWKDRGRRDDFLELTDRVWRTRAYGDFWSYCLVAEGTVDLAAE

PELNLYDMAALVPIVEEAGGRFTGLDGAPGPFSGNAVASNGLLHDQALELIGD

>HisN_Nesterenkonia_alba |551252082|ref|WP_022872352.1| histidinol-phosphatase []

MSTASSPYTEDLRLAHMIADSADDLTMSAFTSMDFEVETKPDLTPVTEADRSAEELIRGQLSRARGRDAV

YGEEFGSSGSGPRRWVIDPIDGTKNFVRGVPVWATLIALVDEGEPVVGLVSAPALGRRWWAAKGGGAYTG

RSMSQARQLQVSSVSTLQDASFSYSSLGGWRDIGRSRSFLEFTEQVWRTRAYGDFWSYCLVAEGAVDVAA

EPELALYDMAALVPVVTEAGGRFTSLEGEPGCSGANALASNGLLHEAALNALGPDAGA

>HisN_Dermacoccus_sp._Ellin185 |494003467|ref|WP_006946007.1| histidinol-phosphatase []

MGAMSDTLRDPSPYHDDLRLAHVLADSVEEITMSRFGAADLQVESKPDLTPVTEADREAERVIRENLKRA

RPRDAIKGEEFGTSGDADRLWIIDPIDGTKNYVRGVPVWATLIALVDAGTPVVGLVAAPALHRRWWAAQG

TGAWTGRSLTSATSISVSKVDRLADASLSYSSFNGWAERGKDMDLFRLSKECWRSRAYGDFFSHMLVAQG

SVDIAPEPELEDYDMAALVPIVTEAGGRFTGLDGRPGCWSGNGVSTNGLLHDEVLARLGDDSPAPSLIPG

E

>HisN_Isoptericola_variabilis |503605374|ref|WP_013839450.1| histidinol-phosphatase []

MLAHRVRVRDRYRVTVASFQHSPSPAGPVRTYEDDLRLAHVIADQVDSITMSRFRAVDLHVESKSDDTPV

SDADRTAEEVIRGQLARARTRDAIVGEEYGSAGSGARRWIVDPIDGTKNFVRGVPVWATLIALADGDEVV

MGLVSAPALGRRWWAARGSGAWTGKSLAAATRLQVSGVRTLADASFSYSSLTGWEEHDKLNGFLDLSRQV

WRTRGYGDFWSYMLVAEGAADIAAEPELEVYDMAALVPIVTEAGGRFTSLEGEPGPWGGNAIATNGHLHD

QALVYLG

>HisN_Tetrasphaera_japonica |665504359|emb|CCH77292.1| putative inositol monophosphatase [T1-X7]

MAGYDDDLRLAHVLADAVERVTMERFKAVDLRVETKPDLTLVSDADTAAEELIRSQLRRTRPRDAVEGEE

FETTGHGPRRWVIDPIDGTHNFVRGVPVWATLIALVDNETPVLGLVAAPALGRRWWAAIGSGAWSGRSLT

SAKRIHVSGVTSLSDASISYSSVSSWRKVDRREELLALLDDCWRTRAYGDFWSYMLLAEGAVDIATEPEL

ALYDMAALVPVVTEAGGRFTSLAGRDGPWGGNALATNGLLHDEVLARLGTA

>HisN_Jonesia_quinghaiensis |656029249|ref|WP_029068442.1| histidinol phosphatase []

MNASRYEDDLRLALVLADQVDNITMSRFKALDLRVESKPDTTPVSDADRTAEEVIRSQLSRSRSRDAIVG

EEYGTTGHSSRRWIIDPIDGTKNFVRGVPVWATLIALAEGDDVVVGVVSAPALGRRWWAAQGSGAWSGRS

LSTATRLNVSKVSSLADASLSYSSLTGWSGLGLRDEFLTLTDSVWRTRAYGDFWSYMLVAEGAVDAAMEP

ELELYDMAALVPIVTEAGGTFTSVDGSVSGPWGPNAIASNGVLHGSILEALTPGR

>HisN_Kineosphaera_limosa |493642000|ref|WP_006593686.1| histidinol-phosphatase []

MPDYNDDLRLAHVLADSVERITMARFRAADLRVDAKPDLTPVTDADTDAEELIRTQLKRARSRDAVLGEE

GGETGGGSRKWIIDPIDGTKNYVRGVPVWATLIALYDNNEPAIGLVAAPALGRRWWAALGTGAWTGRSLS

SAQRLGVSGVRAVGDASLSYSSLGGWRKAGRFDQFVTLADRCWRTRAYGDFWSYMLLADGGVDIACEPEL

AVYDMAALVPIVTEAGGRFTSLDGQPGPWGNNALATNGHLHDAVLESLQASAEAESAPQESVSRT

>HisN_Terracoccus_sp._273MFTsu3.1 |518987161|ref|WP_020143036.1| histidinol-phosphatase []

MTSYDDDLRLAHVLADAVERVTMARFRAADLVVESKPDLTPVTDADRAAEELVRHQLKRTRPRDAVEGEE

FDTTGHGPRRWIIDPIDGTKNFVRGVPVWATLIGLVVDNVPVLGLVAAPALQRRWWAATGSGAWSGRSLS

SAKRISVSQVGRLGDASLSYSSLASWRVRGRRDDFLDLMDDCWRTRAYGDFWSYMLVAEGAADIAAEPEL

AVHDMAALVAIVQEAGGRFTGLDGKDGCWSGNALATNGLLHDEVLSRIGTASA

>HisN_Glaciibacter_superstes |652456898|ref|WP_026851739.1| histidinol phosphatase []

MTPPASYSLDDDLAVAIRLADAADTISRDRFRALDLVVTTKPDRTPVTDADQAVERVIRSGLADSRPSDG

ILGEEYGTEGPTDRQWIIDPIDGTANFLRGVPIWATLISLAIDGVPVLGVVSAPALGKRWWATTGGGAWM

TDERTPGAAPIRLQVSRVSDLTDASLSYNSIQQWDQAGHLDQLVALSRQVWRTRAYGDMWSYMLLAEGLI

DIAGEFDLQPYDMAALAPIVEESGGRFTSVDGQDGPWHGSALATNGILHQATLAALQAPTNRAPTNRTPR

T

>HisN_Kineococcus_radiotolerans |501032384|ref|WP_012084450.1| histidinol-phosphatase []

MPAPTTAPAGQRPRYDDDLRLAHVIADQVDGVTTDRFKAQDLRVDTKPDLTPVSDADQSAEELIRSQLRR

TRPRDAVLGEEFGLVGHGARQWVVDPIDGTKNFVRGVPVWATLIALLDDGEPVVGLVSAPALGRRWWAAT

GSGAWTGRSLSAASRMSVSAVADLSDASFAYSSLSGWEESGSLEGFLGLSRAVWRTRGFGDFWSYMLLAE

GAVDIAAEPELALHDMAALVPIVTEAGGRFTSRAGVDGPHGGNAVATNGLLHEAALGFLGTPAQP

>HisN_Jiangella_gansuensis |652480508|ref|WP_026875138.1| histidinol phosphatase []

MAHDDDLRFAHVLADDADSQTTSRFRAADLRVETKADQSPVTDADKATEEAIRRTLSRARPRDAVVGEEF

GAEGYGARRWVVDPIDGTKNYLRGVPVWATLIALMVEDEVVAGVVSAPALGRRWWAARGTGAWTGRSLSS

ATRCRVSGIDTLDEASLSFSSLAGWEERGRLPAFLDLTRACWRTRGYGDFWSYMLLADGAVDIAAEPELS

LHDMAAPSIVVEEAGGRFTDLAGRPGPLGGDALATNGLLHDTVLDRLGADRDPDQS

>HisN_Corynebacterium_glutamicum |499323406|ref|WP_011013898.1| histidinol-phosphatase []

MSKYADDLALALELAELADSITLDRFEASDLEVSSKPDMTPVSDADLATEEALREKIATARPADSILGEE

FGGDVEFSGRQWIIDPIDGTKNYVRGVPVWATLIALLDNGKPVAGVISAPALARRWWASEGAGAWRTFNG

SSPRKLSVSQVSKLDDASLSFSSLSGWAERDLRDQFVSLTDTTWRLRGYGDFFSYCLVAEGAVDIAAEPE

VSLWDLAPLSILVTEAGGKFTSLAGVDGPHGGDAVATNGILHDETLDRLK

>HisN_Corynebacterium_diphtheriae |504085913|ref|WP_014319907.1| histidinol-phosphatase []

MSYHDDLAFALQLANAADAITLARFAASDLKVSSKPDMTPVSDADIATERELRDLIAQRFPEDAILGEEF

GGTAEFTGRQWIIDPIDGTKNYVRGVPVWATLIALLVDGHPVVGVVSAPAIAHRWWAAQGEGAWRSNPAQ

ESRRIHVSNVAQLTDASVSFSSLDGWKERGLLNNFVALSDETWRLRGFGDFFSYCLVAEGAVDIAAEPEV

SLWDLAPLSILVEEAGGVFSSLDGTLGPHGGNALATNGLLHDEVRTMLTSSR

>HisN_Corynebacterium_resistens |503653684|ref|WP_013887760.1| histidinol-phosphatase []

MTDSPSLAPHGHSADLTLALSLADAADHITMSRFESSDLRIDTKPDLTPVSDADTAVEKKLREIIAAQAP

GDAILGEEFGGTATFEGRQWVIDPIDGTKNYVRGVPVWATLIALLEDGKPVVGAVSAPALTRRWWAANGM

GAWRSFSTPAATGADHPIPSAEPRQLRVSGVSEIGDSSIAISSLSGWAACGLREQLISLTDDAWRLRGYG

DFWSYMLVAEGAVDIAAEPEVSLWDLAALVPIISEAGGRFTSINGEEGPHGGSAVATNGALHSAVLGYLS

SDA

>HisN_Turicella_otitidis |817679558|ref|WP_046644234.1| histidinol phosphatase []

MTSYESDLAVALELADAVSDYTLDAFRGRTRLAVRAKPDDTPVTEADVATEETIRRLLSERRPDDALLGE

ELGGEPELAGRQWVIDPIDGTKNFVRGVPVWATLIALVDAGEPVVGIVSAPALHRRWYAARGAGAFLTEE

DADPVRIGVSKVGKIEDASIAISSLSGWRERNLRDRLVELTDRAWRLRGYGDFFNYCLLAEGAVDVAAEP

EVSLWDLAAPAAVVAEAGGTFTDLDGNPGPAGGSAVATNGPLHEEVLEALTGDWRNYIVSPSR

>HisN_Dietzia_sp._Chol2 (locus_tag_Dietzia_sp.-Draft_1801)

MTARPDRPLDPDLIEHLDELLAATGRIARRHFHGELEALVAGDKGGGRGYDPVTEADRDIEALLRAGISS

MAPGDRVVGEENGESGPADAPRTWYLDPIDGTKAFLTGMAGWGTLVGVVEDGRAVAGWMDQPVLGETFAA

VHGRATVRRRSDGPEAFDLHVSGCDELSEAIMYTTHPSMFGDGELRRRYDDLATRVRLQRFGGDCYAYCM

LAAGRVDLVVESDLKSYDIVALIPIIEAAGGVITGPDGRQPLDGGTVVAAATPALAEQAWAVLGADPR

>HisN_Gracilimonas_tropica |521069925|ref|WP_020401876.1| hypothetical protein []

MIQELLQAATEIAKIGGHHTLKYFQQEIDIISKADDSPVTIADKETEKVMRAEIQKRFPEHGIIGEEFGK

TNPDSNIQWILDPIDGTKSFIHGVPFYTTLIGVLVDNEPQVGIIYAPALEELCAAAIGHGATLNGEPCKV

RDTKTLEEATFLVTEIDRFREMGQQDLFLELLSKTKIHRTWGDAYGHMMVATGRADLMYDPELNIWDAAA

LLPVVKESGGVFSDTHGKQTIHSGNGYSTNKNLFPLVQEIMEKHFK

>HisN_Balneola_vulgaris |516844338|ref|WP_018127608.1| hypothetical protein []

MDINSLKEAAIEIARIGGAHTLNYFKKDFKVISKADDSPVTIADRETEQLMRQEILKRFPDHGIIGEEFE

AVKSESRVQWVLDPIDGTKSFIYGVPFYTTLIGIMIDNEPQVGIIYAPALDELCAAAIGYGATFNGKHCK

VNDTSTLDESLLLVTEINRFNAMGMQPMFQELMDETRIHRTWGDAYGHMMVAIGRAEIMIDPELNLWDAA

ALLPVVKESGGVFADLNGKETIHSGNGYSTNKSLYPKVQEIIDAHLS

>HisN_Gemmata_obscuriglobus |497731428|ref|WP_010045612.1| histidinol-phosphate phosphatase, putative []

MNADWRNRYDLAVNVAQKAGDLARAYYESTFQVEHKADSSPVTEADKNAEKLIREAVTAAFPDDGFLGEE

FGDQPGASGYRWIIDPIDGTKSFIRHVPIWATLVGLEHKGELIGGVVYIPVFGMTYRALRGDGAYHNERR

IRVSNVSSLAECSLCYSSMGWFTRSGREQAFHNLYKQTQRQRGHGDFYGFVLVAEGAADVMIEHGVNPWD

VAATKAIVEEAGGTFTDWGGVPTIHTPDVVATNGKLHSDVLAILRG

>HisN_Pirellula_staleyi |502677198|ref|WP_012912917.1| inositol monophosphatase []

MTTSEISATVLAEVSRRLPLVLSAGKEAGQLTLRYFQQDNFTVEKKGDASPVTIADRSAEQLIRQRVAEH

FPTDGIIGEEFGRTEGTSGFNWILDPIDGTKSFISGVPMYGTMVGVEFEGRSLAGLVYIPGLDEGVYASS

GQGTFHFKGTSQPRRCFVSKKPQLSDGLFVTSQVDTFAKRGGQGAFEAVQKLAYITRTWGDCYGYMLVAT

GRAEVMIDPILNVWDAAAVQPIIEEAGGTFTDWNGVPTIHTGDAVATNGLVADEVLSITKRFPRTL

>HisN_Zavarzinella_formosa |521957636|ref|WP_020469241.1| hypothetical protein []

MNSEWTPRYLAMQQLGREAGKIALDYYNKGAHVETKPDDSPVTIADKSAEKHLRDQLAKLFPGDGFLGEE

YGNEPGTTGYRWIIDPIDGTRCFIRKIPHWATLVGLEFNGEIVAGIAYEPVFDRMYQAAKGTGAFCDGQP

IRVSGIPKLADCLACYSGFRFFQQAGKDSQFHKLLNAVDRARGFGDYYGFMLVAQGSCDLMVDHGVHIWD

IAALKIIVEEAGGAFSDWSGGNDLERPDCVGTNGKVHAETLAILRG

>HisN_Blastopirellula_marina |488728938|ref|WP_002652369.1| inositol monophosphatase []

MNTPQLFADRLELARTLAVEAGKGTLAHFQTDDLIVDRKSDDSPVTVADREAEELIRARVLERFPDDSVL

GEEYGETNGSSPFRWIVDPVDGTKAFVSGVPLYGTMIGVEYEGKAAIGAIYIPGLDELVYAATGHGAWHV

RQGAAPKPAKVNNGAPLEEGLFVTSQASKFAARGAKEAYDRLESTAWLTRTWADAYGYVLVATGRAVVMV

DPIMSVWDASAAQPILEEAGGIFTDWNGDPTARHHEGVGCSQRVHEEVLEILRQFPRTA

>HisN_Planctomyces_limnophilus |502875586|ref|WP_013110562.1| histidinol phosphate phosphatase []

MAYTNADLASRLDFALAVYAEARQLIMSFYQHSSLEVEEKTDFSPVTEADRGAEKLIRERLDVAFPEDGV

LGEEFPEKPGQSAFRWILDPVDGTKSFVHGVPLFGTLIGLEVIEGEHRHCVMGVCGFPALNEVVYASEGN

GTYWKIGDQPPRRVHVSQVSTIEECTFLTTNMQRWQKIGKWEAYTEILERCKLSRGWGDCYGHVLVATGR

ADLMVDPALNPWDAAALVPILQEAGGHFVDWKGVPTIYGKNGISVTGKLKDEVLQILNKS

>HisN_Rhodopirellula_maiorica |495981822|ref|WP_008706401.1| Histidinol-phosphate phosphatase, putative, inositol monophosphatase []

MTSKPDDWRSMHDGRLSAMIEVARAAGQHTLKYFGTSELVVDAKKDDSPVTVADREAEQLVRKLLGERFP

DDTLQGEEFAEKAGTSSYRWVVDPIDGTKSFVCGVPLYSTLLAVECDGEVIGGAIFIPALDEMIVAGVGQ

GSWHRKTSNSDWNQAAVSSKTDLSTAVFVTSEVGSFAKRGNPNAYAQLERDAWVTRSWGDGYGYLLVATG

RADVMVDPICNAWDVAAIMPVIQEAGGKFTDWKGTSTVRGGDGVGTNGHLHAAVLERLA

>HisN_Chloroherpeton_thalassium |501492390|ref|WP_012500828.1| histidinol phosphatase []

MSPELDFALTAAKEAGQITLKYFRQKSLKVDKKRDRTPVTQADREAETKIREQLQKFFPKDGILGEEFDE

KGTENQRRWIIDPIDGTKSFIHGVPLYGVMIGFEDAGALKLGVVNFPALSLCYYAEKGKGAFMNDEKISV

SEIADFDEATLACIGGEYLMDTESTHPFDTIKTKAGLVRGWGDCYAHMLVASGQADLAIDPEMNSWDCAA

IIPIMEEAGGKCFDYNGVNTMSGRGLVSTNAQLGDKLLSMLYK

>HisN_Chlorobium_phaeobacteroides |500067327|ref|WP_011745623.1| histidinol phosphatase []

MSPELQLALELADRAGRLTLEYFNRKSLQVFSKRDATPVTEADRNAEILIREGISARYPRDGLLGEEFDE

KHADNGRRWIIDPIDGTKSFIHGVPLYGVLIALEEEGLPRLGAVGFPALGQLFYAETGCGAFLDGTRIEV

SSLSSLDDATVVFTEKEYLLDPLSEHPVDRLRVEAGLVRGWGDCYGHMLVASGRAEFSVDKVMSPWDCAA

LVPVVNEAGGCCFDLNGRATIYGDGMVSANRILGMELVKSIEKQRLGS

>HisN_Pelodictyon_phaeoclathratiforme |501499487|ref|WP_012507730.1| histidinol phosphatase []

MTPLTSDLQLAVEMAEQAGQLTLSYFSRKSLQVFTKRDASPVTEADRKAEELIRSAIASRNPEDGVLGEE

FEERPSGNNRRWIIDPIDGTKAFIHGVPLYGVMIALEIDGTVNLGVVHFPALGELYYAEKGGGAFLNGSP

IAVSSISDLKDATVLYTEKEYLLDTLSQHPVDQLRHKAGLVRGWGDCYGHMLVASGRAEVSVDKVMSPWD

CAALIPIVTEAGGCCFDYRGKTTISGEGLVSANKAIGRALLAEIEG

>HisN_Chlorobaculum_tepidum |499235557|ref|WP_010933097.1| histidinol phosphatase []

MPMTPDLQLALELAEKAGKLTLDYFGRRSLQVFSKRDDTPVTEADRKAEELIRQGISAKFPGDGLFGEEF

NERPSGNGRRWIIDPIDGTRSFIHGVPLYGVMIALEVDGVLRLGVINFPALGELYHAEIGAGAFMNGSSV

QVSAIAETAAATVVFTEKEYLLDPPSTHPVDLLRSSAGLVRGWGDCYGHMLVASGRAEVAVDKIMSPWDC

AAVIPIVTEAGGCCFDYRGRRSIIDGEGLVSANRSMGEALIEAIGKGERAR

>HisN_Prosthecochloris_aestuarii |501497836|ref|WP_012506095.1| histidinol phosphatase []

MTDDLQLAIELADEAGKLTLTYFQNKSLKIDEKRDDTPVTEADRKAEQLIRKGIEARYPEDGIFGEEFGE

KIAVNGRRWILDPIDGTRSFIHGVPLYGVMIALEVNRAMQLGVIFFPALQEMYYAQSGCGAFMNGEPLKV

SEIGDTREATVVYTEKEYLLDPPSDHPVDRLRYDAGLVRGWGDCYGHMLVASGRAEVAVDKVMSPWDCAA

VIPVVTEAGGCCFDYNARTTIYGEGLVSANRKIGDELVRDILG

>HisN_Candidatus_Entotheonella_sp._TSY1 |575404898|gb|ETW92460.1| hypothetical protein ETSY1_43535 []

MPVLPMQLEAAIHAVQAAGEVILPYFRTELQVETKADQSPVTAADRAAEQVIVETLQSQFPDYGFLGEEF

GEQAGNTDARWIIDPIDGTKNFIRGIPYFATLLALEESGDITHGVIYAPAEQTLFYAAKGFGAFTNGHQP

LEVSSIDELSQAMLVHGGLDILRQGGYWDGFTRLIDATARQRGFGDYFAHTFVFRGQAEVMVEADVKPWD

LAPLKIIAEEAGGRFTDFTGQPTIYGGNALVSNRHVHAEVLKLLGPISGTS

>HisN_Anaeromyxobacter_sp._Fw109-5 |501045468|ref|WP_012097271.1| inositol phosphatase []

MPDLDLDLALETARRAVEAATAASLAHFRRGVRVEVKPDRTPVTAADRESEAAILAVVRAAFPDHGFLGE

ETGAHAGAAATRWIVDPLDGTKGFTRGRGFWGPLVALEHEGAVVAGAMALPALGETYWAARGRGAWLRSG

EGAPLRLAVSRIAAWEDATLSLGEPHVLFRPPLLERVAALATSAQCARCYGDLAGAALVLQGKAEAWVEA

GVQLWDLGPLPVLAEEAGGRFTDLEGRPTPASGSCVLSNGLVHDHVLRALAGR

>HisN_Roseiflexus_castenholzii |501068400|ref|WP_012119489.1| histidinol phosphate phosphatase [] Genom; phyl=Chloroflexi; 4e-44

MASETLEALREFAADLAWHAGRLTLRYFQTGITPDIKEDRTPVTIADREAEQVMRRMIEARYPHHSILGE

EEGETRPGASHRWILDPIDGTKSFVQGVPLYGVLVGLERDGEAVVGAVSFPALGDFLTAAKGQGCQWNGR

QARVSQVREVRQATLLSSDAESMAPRGREAAYRRLAASVRLVRTWGDAYGYSLVATGRAEIMLDPVMSVW

DCAALFPIVTEAGGTFTDWHGAPTIHAGEAIGTNGLLLEQVLNIIRG

>HisN_Oscillochloris_trichoides |493608800|ref|WP_006561215.1| histidinol phosphate phosphatase []

MLDVAHTLAWQAGKITLRYFQSGVAVDHKADESPVTVADRETEAFLRQAILTRYPDHAILGEEAGLSGAD

DAEYRWILDPIDGTKSFVRGVPLYGVMVGVLYKNKPLIGVVNMPALNEIVYAGTGLGCWWNGRPCRVSAI

NNLRDSLVVATVAHGYEQYGKGAAFQRLLSQAGMFRTWADCYGYLLVATGRAEVALDPIMNVWDAAALAP

IMLEAGGTFTDWQGVASIEHNEGIGTNGAVLPEVLDLIG

>HisN_Herpetosiphon_aurantiacus |501143645|ref|WP_012191843.1| histidinol phosphate phosphatase [] Genom; 5e-42

MPSLRELLDVATEAAYLGGRRTLAYFGADVQVETKGDATPVTRADREAETIIRDYIGRYFPSHTIIGEEH

GEQKGDADYRWIIDPIDGTKTFIHGVPFYGVLIGLEIKGEASVGAVYLPAFDEMLAAANGLGCSWNGRPA

QVSKVDNLADATLLTTSVTSAMKRSNAYETLVSKTKLQRTWGDCYGYVLVATGRAEIMLDPAMNPWDCAP

MLPILREAGGHFTTWAGEATIWGADGMATNAALHQEVLAILATEQRHI

>HisN_Nitrospina_sp._SCGC_AAA288-L16 |516577070|ref|WP_017952134.1| hypothetical protein []

MDLLEAKNTALSCLKISSKEIMSWFRTNMTVESKSDQSPVTIADHKAEEILRKKISKAYPDHGIIGEEFG

EEGSQSDWVWTIDPIDGTRSFIRGLPLFATLIALLHKGDPVMGVISLPALGETAWAVKGRGAFSENQRLQ

VSSHGIAKGSFVGIADKYCFKEKKCLPLYNRIHREAKIVRTYPDAFGHLMAIRGAIDVMVDPWAYIWDYA

PCKIMVQEAGGEFANFTGSKAGIGVGNAISGNPKMVKAIRAMIREANSRTKK

>HisN_Fibrobacter_succinogenes |504359818|ref|WP_014546920.1| histidinol phosphate phosphatase []

MENREIAKAGVAPENLELLKIALKTAELAEENILKYYQNDVGVEWKADKTPVTIADKGTEELARKFWAKE

TPGFGVIGEEFGIESPDAEYQWVIDPIDGTKSFIHGVPLFGTLIGLYHKNVPIASVIRLPAMKSAVWAVN

GGGAFLDGREVRASKVSQLSDALVLSGTVNTMEDKGFGEGFTKLRRSARLHRGWGDCYGYYLVAAGRAEI

MVDPVVSMWDIAPFPLLMKEAGGKFSTIDGKTELFDVNGKPTAPIYEGFTSIATNGLLHDAALDCLLSSK

L

>HisN_Chitinivibrio_alkaliphilus |550655170|ref|WP_022635886.1| inositol monophosphatase family protein []

MNTYETELRHAADIAREVGEYQRRAQKEIGRIHIKEDSSPVTEVDTTCENIIYRALSRAFPEDGFFGEEG

GIRAGTSGRRWIVDPIDGTRPYIRGIPTYSTLIALEDHGEITVGVVNLPALGECYTARLGGGAFCNDTPI

SVSNTSSLEAAMGAFLGTVETAREPRGKKLFSLMEHIDYPYGFMDAYTYMAVASGKLDLAVGMIDFPWDR

ASAAIIVKEAGGKCTDSLNKETIYGDTFILSNKKIHPEIVPYLK

>HisN_Spirochaeta_alkalica |648553124|ref|WP_026244875.1| hypothetical protein []

MMFLDFAQNLARIGGSVSLDWFARHDLAVEQKSDDSPVTIADKATEAAIRREISRRFPDHAILGEEQGGT

LGDHTYQWVIDPIDGTKTFIRGVPLYTTLIALLREGEPVVGVIYAPATGEMVSAALGHGARDERGRAVKV

SSTTALKDAWFATTDPADLYKRDPRLSTGLLEHCLAARTWADAYGYMLLARGAIDIMTDPVMSPWDIAPL

GIIVREAGGVFCNLQGESPAIGTSALACATAELQRAVLALGKPEA

>HisN_Bacillus_sp._EGD-AK10 |545119541|ref|WP_021482063.1| histidinol-phosphatase []

MTPPSTRGYDDDLRLAHVIADQVDAHTLSRFKALDLHVESKPDSTPVSDADRTAEEIIRGQLGRARGRDA

IVGEEYGETGHGARRWIVDPIDGTKNFVRGVPVWATLIALADGDEVVVGLVSAPALGRRWWASKGTGAWT

GKSLAAASRLHVSGVSSWSDASLAYASLDGWEERGKLEPFLDLMRGAWRTRGYGDFWSYMLVAEGAVDAA

AEPELELYDMAALVPIVTEAGGRFTSLDGADGPWGGNAVATNGLLHDEVLGRLG

>HisN_Dehalobacter_sp._FTH1 |521977482|ref|WP_020488753.1| hypothetical protein []

MPDQALLDDLRLAHVLADQADAITMARFKASDLVVSTKPDMTHVTDADTAVEDAVRTMLSRTRPRDAVHG

EEREDSGWGPRRWVIDPIDGTANFVRGVPVWATLIGLMVGDEVVAGVVSAPALSRRWWAHKGGGAFTGRT

LLQGVPLRTSAVGDLGDAFLSYSSIAGWVESGRGQQFVDLMRDCWRTRAFGDFWSYMLVAEGSVDLAAEP

ELALHDMAAVSVIVTEAGGRFTNLDGRDGPHGPGAIATNGRLHDEAVERLAADD

>HisN_Zymomonas_mobilis |499560507|ref|WP_011241290.1| histidinol-phosphate phosphatase [ZM4]

MSRSAYEDDIRLAHRLADVAADIIRPFFRAPLTIDLKADHSPVTKADRGAEQAMRAILEQERPEDGIFGE

EMGVSRPDARRLWVLDPIDGTRAFIGGRASFGTLIALVEDGRPVLGIINQPIHQERWVGVKDLPTSFNGE

VIHTRSCPALDHALLATTSPWLFEKEGEVHFDKIRLKCRDTLLGGDCYNYGLLSLGHCDLVVEQGLKFYD

FAALVPIVEGAGGIMRDWQNRPLNKNSVGEVIAAGDHHLIEPALSAMEL

>HisN_Photorhabdus_temperata |544914949|ref|WP_021324694.1| Mono-phosphatase []

MKLDSKDDLRLAKRVAMEAATTALSLRESSEFQVTLKKDGSPVTPADIGAELRVRELIESERPNDLFFGE

ESGGLAVHKGRVWIIDPIDGTKNFVRGIPIWATLVALVEDGEPKIGVVFAPELKRCWWAEKGGGAYFSKS

PQGSGNRMHVRQISGLKDAYISTTAFDTWHKQGLLQQYQRLAEQTFCNRGFGDFLQHCLVAEGILDVAIE

PIVAPWDVAALVPIVEEAGGICTDLMGQPVLANKGVGIASTSQTLHKHVIDIFGE

>HisN_Xenorhabdus_nematophila |498913364|ref|WP_010848538.1| Histidinol-phosphatase []

MKLNSGDDLRLAQRVAVEAATTALSLRESGEFQVTLKKDGSPVTPADIGAELRVRELIESERPNDLFVGE

ESGGLAVQKGRVWIIDPIDGTKNFVRGIPIWATLVALVEDGEPKIGVVFAPELKRCWWAEKGSGAYVSKS

PQGSGNRMHVRQASGLKDAYISTTAFDTWHKQGLLQQYQRLAEQTFCNRGFGDFLQHCLVAEGILDVAIE

PIVAPWDVAALVPIVEEAGGICTDLMGQPVLANKGVGIASTSQTLHKHVIDIFGE

>HisN_Leucothrix_mucor |551333504|ref|WP_022952941.1| inositol-phosphate phosphatase []

MSPFLKTAIEAAQAAQLVIDKYYAGEFEVEIKPDQSPVTIADVETEETIKRIILNAFPDHGFFGEETGKV

NEDADYNWLIDPIDGTKSFVRGYPFFSTQIALMYKGELIVGVSNAPGFKEMAYAEKGQGAWLNGEKIQVS

DIQELSKSTLSLGNIATIAGKPQWQTLGGLIQDVHRIRGYGDFYHYHLLASGKIDIIIESDVNILDIAAL

SVIVNEAGGCFTDLSGNVLTLDTTTVLACNTKAMHQSMLDRVQYS

>HisN_Rhodanobacter_sp._OR444 |653329702|ref|WP_027493054.1| inositol-phosphate phosphatase []

MNPDIAATALAAAREAAAAAAEVIRYYWRRGVEVELKPDATPVTIADREAEQAIRKVLQAALPQASIYGE

EFGLDGERGGLLWLVDPLDGTKSFVRRTPFFSTQIALMDGDELVLGVSSAPVYGETMWASAGNGAWLDGE

RVRVADTAQMAQASISIGNVKTLTADVRWEALGALIRDSNRIRGYGDFCHYHLLSRGSLDLVIESDVNIL

DVAALAVIVREAGGAFTDLDGAPLALGTTSVLAGAPAIHAQALQRLRRRAAG

>HisN_Thiobacillus_prosperus |684048810|gb|KFZ89944.1| histidinol-phosphatase, inositol monophosphatase family [= Acidihalobacter prosperus]

MQGLPMQQSVYLQTALAAARAAEDVIRRYWAEGVEVRLKADQSPVTEADVAAEQAIRQVILEAFPTHGFY

GEETGRTREGAEYTWLIDPIDGTKSFIRRTPFFSTQIALMQGERLLLGVSNAPLYGEMAWAERGAGAFVN

GLAVRTGEVTALADAAISLGNIKSLAQGEGWARLGDIVAGVNRTRGYGDFCHYHMLAAGQLDLVIESDLN

ILDVAALSVIVEEAGGVFTTLDGGAIGLETGSVLAAGTRALHTLALDRLRAD

>HisN_Thioalkalivibrio_sulfidiphilus |501778521|ref|WP_012637228.1| inositol-phosphate phosphatase []

MSPFLQTAIEAAQAAQKVIQRYYRQEIPVELKADQSPVTIADVETEKTIKGIITGAFPAHGFFGEETGHS

TGSAPYTWLIDPIDGTKSFVRHYPFFSTQIALMHTRELVMGVSSAPEFEEIAYAEKGLGAYLDDKPIRVS

QVEDFAAATLSLGNIKTLAGSDRWEALGSLVRQVNRTRGYGDFYHYHLLASGRIDLVVESDVNILDIAAL

AVIVREAGGVFTDLDGRELTLETTSVLAAATPALHARALDLLKGS

>HisN_Salinisphaera_hydrothermalis |666934069|gb|KEZ76526.1| Inositol-phosphate phosphatase [C41B8]

MFKQGETVAEDLSRYLETAIEAARAAETVINRYYHGDFEVERKADASPVTIADVECERTIKRVLAEAFPD

HGFYGEELGRESIDADYVWLIDPIDGTKSFVRGYPFFSTQIALMHQGELIVGVSNAPAFGNGDMAYAARG

QGAFLNGERIHVSTIDEIAETSLSTGNIGALGASPAWPKIGELVGDVHRIRGYGDFYHYHLLASGAIDAV

IESDLNILDIAALTVIVREAGGDVTELGGGPIDLESRTALATNGRVRAPLAPYLAAWNEAEHAPRD

>HisN_Wohlfahrtiimonas_chitiniclastica |495590826|ref|WP_008315405.1| Histidinol-phosphatase []

MEQFLKVALLAAQESKKMIRKAYEAQGFNITIKGDYTPVTEVDVNVETLIRSIIVENFPEHGFWGEEGGM

INPESDYQWLIDPIDGTKAFIRRRPFFSTQIALMYKGEIILGVSSAPCFGEGEMAYATKGNGTFINGERV

HVSDITELKDAVFSSGNIKSLAKDSTKWQNYGQLLLAINSTRGFGDFLHYHYTAAGKVDIVVESDVTILD

VAALSIIVTEAGGKVTDLAGQPLSLESTNIVATNGALHDAVLSYLQ

>HisN_Ignatzschineria_larvae |652484755|ref|WP_026879357.1| inositol-phosphate phosphatase []

MEMQPYLDCALEASEIARQLIQTAYEENAFKIEIKADATPVTEVDVAVEKAIYQHISSQFPEHGFYGEES

GQKQMDSDFIWLIDPIDGTKAFVRGRPLFSCQIALMVRGEIILGVSTAPCFNGGERIYAVKGQGAFLKGK

KISVSDIDTLSQAVFSSGNLKRLTQDPEKWARYGNLVGQVNSTRGFGDFLQYHFLATGKVDLIVESDVNI

LDIAALSIIVNEAGGKMTALDGSPIDLSVSTILAATPSLHAQALEILQF

>HisN_Thiorhodospira_sibirica |493840605|ref|WP_006787748.1| inositol-phosphate phosphatase []

MSPFLQTALDAALNAQEIISHYYRQGVAVKLKADQTPVTVADIEAERAIKACILGMFPHHGFYGEETGRE

RADAEYVWLIDPIDGTKSFVRGYPFFSTQIALMRDGELVLGVSNAPEFQELAYAEKGLGAYLNQQPIQVS

HKEHLPEITLSLGNLTTLTQGERWQGLGRLIQKVQRTRGYGDFYHYHLLASGRIDLVIESDVNILDIAAL

TMLVNEAGGVFTDLDGQPIGLETTSVLAAANPVLHDKALRLLGIDED

>HisN_Pectobacterium_carotovorum |684996308|gb|KGA33979.1| inositol monophosphatase [subsp. brasiliense]

MSQSLPDIAFFHELATLASQETLPRFRSLTANQIETKPKEGFRFDPVTEADREAERVIREHITRYYPDHA

IMGEEFGLSGEGPMRWVLDPVDGTRPFLCGLPVWGTLIGLLHHKRAVMGMMSQPFTGERFWADGSQAWRS

DRQGEMRLSTRKGVSLEQAILHTTAPEALAMHPAVCFADLAESTLMTRYGGECYAMAMLAAGQIDICVEF

ALQPYDIVALIPIIEQAGGIITDLNGQRAEAGGTVVATGNPELHQQVLTILNGTRS

>HisN_Acinetobacter_baumannii |690994542|ref|WP_031963998.1| inositol monophosphatase []

MTNIFPDIYFLHKLADVADAETLPRFRSHIDLEIQTKLKKNVSFDPVTIADKAAEYAMRKMITEYYPDHS

IMGEEFGAIENGEIRWILDPIDGTRPYLLGIPVWGTLIGLEHNDKMCLGMLSQPFTNERFWSDGESSFHQ

YNKVQKKLKIQRNKKISEAILHINHPRSSNLYPHINFTELSEQVLMTRYGAECYAFAMLAAGYIDICFEF

SLQPYDIAALIPIIENAGGVISTLDGSSAHKGGTIVAASSQSLHEQALKILNS

>HisN_Nevskia_soli |659875744|ref|WP_029922551.1| inositol-phosphate phosphatase []

MPSALLNAALEAAEAASTLIRKAYRGNFQVNYKADASPVTEVDIAAEGAIKAVLKNHFPSHGFYGEELGR

ETGDAEHLWLIDPIDGTKAFVRGYPLFSVQIALMRRGELVLGVSSAPCWNGGIGETAWAEKGQGAWLDGE

RLRVSDNTDLAKATLSTGNLARLARSEAWARLGGLIPRLHRIRGYGDFLHYHWLAANKLDAVIESDVNIL

DIAACSVIVHEAGGKFTDLQGAPLGLDTTSVLAATPALHGQLLQELRYTPPA

>HisN_Dyella_japonica |651817924|ref|WP_026633519.1| inositol-phosphate phosphatase []

MSAPDLQRALSAAREAAEAAAEVIRHYWRRGVEVEIKSDATPVTIADREAELAIRKVLQAALPEAAIYGE

EFGLDDGARELLWLVDPLDGTKSFVRRTPFFSTQIALMHKGELVLGVSSAPVYGETLWASAGGGAWFEGD

RVRVAATDSMAQASISTGNVKTLTMDARWAELGAMIRDSNRIRGYGDFCHYHLLARGGLDLVIESDVNIL

DVAALAAIVREAGGLFTDLDGQPLTLDTRSVLAGTPALHAQALTRFRASLQS

>HisN_Frateuria_aurantia |504216588|ref|WP_014403690.1| inositol-phosphate phosphatase []

MDEAVLAHALAAAREAAAAAGDIIRHHWQRGVEIELKDDQTPVTVADREAELAIRAILQRALPEAGIYGE

EFGRQGADGRALWLVDPLDGTKSFVRRTPFFSTQIALMLDGELVLGVSSAPIYGETMWAVRGQGAWLDGQ

RVRVASTDNVAAASISTGNIKSLAAGPQWPLLGQLIGQCNRIRGYGDFCHYHLLARGSLDLVIESDVGIL

DIAALAVIVREAGGVFTDLRGGALDLDSRSVLAGTPVLHGLALRQLQSGAR

>HisN_Halothiobacillus_neapolitanus |502586328|ref|WP_012824087.1| inositol-phosphate phosphatase []

MSSIYLETALAAARAAETVVRKYWQQNVDITIKADASPVTVADVETEEAIKAVILEQFPDHGFYGEETGK

TRPDAEYTWLVDPIDGTKSFVREYPFFSTQIALMRGDQLILGVSSAPVFGEYAYAEQGGGAFWMDKAAKV

SQIDTMAAATLSVGNLKTLAANPTGWAGLASMVREVNRTRGYGDFYHYHLLARGAIDLVVESDVNVLDIA

ALSVIVLEAGGVFTDLEGKPVGLDTTTVCAAATPELHSEARRRLGY

>HisN_Solimonas_soli |654616354|ref|WP_028080900.1| inositol-phosphate phosphatase []

MASPHISAALEAARAATDIIRKAYRGNFSVEYKADASPVTEVDVAAERAIKAALRARFPDYGFYGEETGR

DIAAGSEYLWLVDPIDGTKAFVRGYPIFSVQIALMRGSELIMGVSCAPCWNEGLGEVAWAERGAGAWRGL

LETPLADYERLRVSTIDQLGKATLSTGNLATLARAPQWLELGRLIPQLHRIRGYGDFVHYHYLAAGKLDA

VVESDVNILDIAALAVIVEEAGGRFTDLNGRPPSLETRTVLATNGKLHEAFAGLAL

>HisN_Providencia_burhodogranariea |496186745|ref|WP_008911252.1| histidinol-phosphate phosphatase []

MNKHSLPDSFFLHELANIAGKASLEHYRTNELTVLENKPKADYRFDPVTQADKLAEKLMREHIEHIYPNH

SIMGEEYGITGEGPIQWVLDPIDGTRPFLCGLPLWANLIGLTVDGTATMGMMSQPYIGERFWADETGSWT

SNRQGQYRLQTRKNVALEQAILHTTSPEPIENHPNIHFTELTRKVLMTRYGGECYAMAMLAAGRIDVCFE

FALEPYDIVPLIPIIEQAGGRVTTLDGNRVEKGGAILATGCPILHEQILHILNHG

>HisN_Morganella_morganii |692181850|ref|WP_032098376.1| inositol monophosphatase []

MNTHHPDIAFFHQLANRAAQETLPRFLPGHTLSVDTKIKKGVSFDPVTDADRQAEVALREMISAAYPQHS

ILGEEFGLTGEGDYQWILDPVDGTRPFMLGLPVWGTLVGLICRGTPVMGMMSQPYTGERFWADGERSWHS

SPHGTYVMKTRKNITLANAILHTNSPEPMPRFPEIRLTDLMEKVLMTRFGGECYAMAMVAAGKIDLCFDY

ALQPYDIAAFIPIIEQAGGCVTTLDGGPAIQGGAVLASGCPSLHQQVLTILNKNI

>HisN_Beggiatoa_alba |488762939|ref|WP_002686139.1| histidinol-phosphate phosphatase []

MMKPVINAQLFDFINQMANASATIIKSYFRTHLTVDDKPDTSPVTLADKAVEQKLREMIEKNYPHDGIYG

EEFGTKNENAEFTWVIDPIDGTKSFITGKPLFGTLIALIHQGKPILGLLDQPILKERWIGGQHLPTTLNG

HPIHVRACTDIKKAALYATTPAMFLGADFEAFGRVRDTVKMTIYGGDCYAYALLATGFVDLVIESSLKPY

DYCALAPIVENAGGIMTDWQGQPLTIHSDGRVIAAGDAHTHQQALNLLQQ

>HisN_Brucella_abortus |672581994|gb|KFH18943.1| inositol monophosphatase [LMN1]

MLIDKAFFSEVAAAAAAQTLPRFRQLTEVDNKYSVGFDPVTEADRAAERAIRAVIGRTFPDHGILGEEYG

AENTDRSHVWIIDPVHGTRAFISGLPVWGTLVGLTVDGDARAGMMSQPFTGELFYSDGDGAYLQRGDGAP

RRLCVRKNAVLEDATLFTTTPALFKGDDRKAFDRLESAVRLSRYGVDCYAFAMLAGGFVDIVVEAGLQTY

DIAALIPIIEQAGGVVTRRDGGPAEQGGDIVAAATPALHQAALDLLHI

>HisN_Halotalea_alkalilenta |653099233|ref|WP_027349349.1| histidinol phosphate phosphatase []

MRFAQALADTASPLTLGRFRQSFEIDTKSDESPVTIADRETEAALRMMIAQRYPDHGILGEEHGRERLDA

EYVWVIDPIDGTRSFISGWPLWGTLIALLRDGRPLLGVIDAPALGERWSAQVGEPTRLNGEPCRVSGCET

LAEARFYTTSPFYFAADERNGLDQVMAQAALVRFGGDCYGYGLLASGHIDLIIETGLEPYDYLALVTIVE

GAGGVISDWRGRPLGLDSRGQVIAAASPELHRQALAMIAAAAR

>HisN_Hydrocarboniphaga_effusa |494335465|ref|WP_007184817.1| inositol-phosphate phosphatase []

MTTSPFLDAALAAADAASDLIRRAYRGNFSVEYKADASPVTEVDVAAEKTIKGVLQSRFPDHGFFGEETG

RSNNDSDYLWLVDPIDGTKSFVRGYPMFSVQIALMHKGELILGVSSAPCWNDGRGETLHAEKGQGAWLDG

QRLAVSSTSAIEKTTLSTGNLASLARQPEAWSRLGALIPRLHRIRGYGDFLHYHLLASGRIDAVVESDVN

ILDIAALTVILREAGAVFTDLRGEPVGLATTSVLATVPALYGPIKDAISF

>HisN_Kosakonia_radicincitans |494616506|ref|WP_007374749.1| inositol monophosphatase []

MNHIVPDSAFLHELADAADQQTLPRFRAQTNLHVGSKPKEGFRFDPVTDADREAERVMRDIITARYPEHA

IMGEEFGVTGSGPLQWVLDPVDGTRPFLCGLPVWGTLIGLTVEGRSRIGMMDQPFTRERFWADGEQAWHQ

GPQGTTQLQTRRGIALEQAIFHTTSPEPVSRFPAINFAALEASTLMIRYGGECYAMAMLAAGQIDLCLEY

ALQPYDIVALVPIIEQAGGVVTTLDGGRPESGGHILASGCPFLHEQALRILNR

>HisN_Mangrovibacter_sp._MFB070 |653405475|gb|KEA54227.1| inositol monophosphatase []

MPLKKPESDFLHELANIAEQRILSSYLKSHCIDISTKPKEGFRFDPVTNIDRETEIIIRRMISERFPEHA

IMGEEFGDTGSSTYKWVIDPIDGTRPFLCGLPVWGCLVGLLENGEAVMGMMSQPYTGDRFWSDGKCSWFS

GRFGTSIMKTRQDAELHQAILHTTSPESVKLYHNNKFDLLQNQVLMTRYGGECYAMAMLAAGHIDICVEY

SLQPYDIVAFIPIIENAGGVITTIDGRRAENGGAIVVSANEQLHEQVLNILNS

>HisN_Marinobacterium_rhizophilum |648599756|ref|WP_026291507.1| hypothetical protein []

MEKRLGFARDVAEDAGKIALRYFRQPLDVENKLAGEQFDPVTIADREVEAEIRQKLGAAYPQDSIIGEEE

GTSRGSNDIAWIIDPIDGTRAFISGVPAWGTLLGLMDGERCVGGLMHQPYIGETFIAGPAGAFLYHRGQQ

TVLRSRQNATLGDAILYCTHPKHFETEADLAGFYRVAQASRLHRFGGDCYSYCLLAQGQIDLVIEGGLQP

YDIIPLIPLIEAAGGVVTDRNGEPATRGGLIVAAANAKLHAEALALLNG

>HisN_Polycyclovorans_algicola |659836349|ref|WP_029888630.1| inositol-phosphate phosphatase []

MSYAPHLHAARSAAEAASALIRKAYRGQFDVQLKADASPVTEVDIAAEKVIRQTLSAAFPDHAFYGEELG

RGDTDAEYLWLIDPIDGTKAFVRGYPMFSVQIALMHRGELVLGVSCAPCWNGTGETAWAEKGQGAWLDGE

RIHVSEIDTLAKATLSTGNIATLAASADWAKLGALIPRLHRIRGYGDFLHYHLLASGRVDAVIESDLNIL

DIAALTVIVREAGGTLTELGGGAIGLDTTSVLATNGRLGVW

>HisN_Pseudomonas_putida |505083286|ref|WP_015270388.1| histidinol-phosphate phosphatase []

MSLSAEQIGEFRAFAEQLADAAAVAIKPYFRASLDVEDKGGRLYDPVTVADKAAEDAMRELIQARYPDHG

ILGEEAGVAVGSSPLTWVLDPIDGTRAFITGLPLWGTLIALNDGTHPVVGVMNQPFTGERFVGTPEGAWR

SGAPLKTRACADLASATLMCTTPDMFDTAARKAAFEAVAGKARLMRYGGDCYAYCMLASGFVDVIVEASL

QPYDVQALMPIIEGAGGVITAWDGSSAQNGGCVVACGDPALHAQVVEMLRHAM

>HisN_Pseudomonas_pseudoalcaligenes |489546409|ref|WP_003451057.1| inositol monophosphatase family protein []

MNDHTLPPEYLAFAEELADAAARVTLTYFRLPLEVENKEAERFDPVTLADKGAERAMRDLIAQRYPGHGV

LGEEEENLKGEEPWTWVLDPVDGTRSFISGIPLWGTLIALNDGTRPALGVMDQPFTRERFIGDGASASLN

GRPIRTRACRDLADATLMVTSPEHFLQPPYSDAFQRLSSQARLVRYSGDCYAYCMLALGLVDVVLDPGLK

PYDIQALMPIIQGAGGVVTRWDGGDAQDGGDVIACGDPRLHAQLLELLR

>HisN_Rudaea_cellulosilytica |648601632|ref|WP_026293383.1| inositol-phosphate phosphatase []

MPTPAFLDSALDVARQAAQAAAEVIARHWRAGTEVEIKSDQTPVTIADREAEAAIKHVLRAAFPEHAYYG

EEEGREGDGDFLWLIDPIDGTRSFVRGYPMFSTQIALMHRGELVLGVSSASEFGELAWARKGGGALLGGK

PIRVAATNAFDAQTAISFGNVKTLSRGPGWNALAGLVQRCGRTRGYGDFYHYHLLARGSLDLVIESDVNI

LDIAALAVIVREAGGTFTDLGGHALSLATTSVLAGTPALHAQALQLGLT

>HisN_Marinobacter_sp._BSs20148 |504685588|ref|WP_014872690.1| inositol monophosphatase []

MNMNESLLKVVHETLAESGTIARHYFRQSLAVTAKRDLSPVTLADQEIEAAMRKVIHQHFPEHSIVGEEG

EDQLGTGAYTWTLDPIDGTKSFISGLPLFGTLICVSREEQFLIGAIDMPILNERWIGVLGQGSTFNGTPC

KASDVTDIGSATLFSTEPNMFNTGQAERLKKLESAVRLRRYGGDCYSYGLLASGHIDLVVEASLHPYDWM

SAVPIIQEAGGVITDWQGNALHRNSEGSVIAAGTPELHSAALALLAGANH

>HisN_Salinispira_pacifica WP_024268128.1 Histidinol-phosphatase (alternative form)

MKNFPEPGELISYLEDAKAFARIGGNISSHYFGSQIDVHWKADDSPVTKADRESEQAMKKAILEKFPDHS

VLGEEFGESGTADSPWQWVLDPIDGTKSFIHGIPLYTVLVSLTYKKEPVIGVVYNPQSNEMLAAGMGMGC

WYNDSACRVSETSSLSEARLMTTDPGDLIRRSGNPGIELLKTSAISRTWADAYGYLLVATGRADVMLDPI

MSLWDIACLYPIISEAGGEITDIRGKRGISDSAVASNGILHQEIMSVLGNNEYHQH

>HisN_Turneriella_parva WP_014805274.1 inositol monophosphatase

MNHAQKFFEDFLPEATAISREVSRRFFKKPLGEIGMISKADASPVTAADLEIETRLRELIANKFPGHGII

GEEHGSENTEAEFVWVLDPIDGTKSFITGVPLFTTLIALQQNGEPQCGAIYQPILDELVWGNNRECFFNG

QPVRMRTTENLTDATLLITDARHAAEHHPKTNFNALAAESKFWRTWADGYGYTLLATGYADIMLDPVMNP

WDIMAAVPVIRGAGGTITDFAGNDAVKGKSIIAACPALHQKVLKKLSDA

>HisN_Sphingomonas_sanxanigenens WP_025294379.1 histidinol-phosphatase

MVSDSDIQLARRLADAAGAAIRPFFRQRFEIETKGDNSPVTEADRAAEAAIRALLATERPADGVIGEEYG

IDRGDAERVWVLDPIDGTRAFIGGRPIFGTLIALLEAGRPVLGVIDQPISGERWLGATGHPTLFQGAPAR

TRRCPVIPSALFATTAPWLFDEIGGHWFETLRMRCRDTLLGGDCYNYGLLASGHIDLVVEQGLKLYDFAA

LVPVVEGAGGRMCDWQGRALGDGSDGRVIAVGDPALADAVLTLFG

>HisN_Sphingobium_ummariense WP_021316639.1 histidinol-phosphatase

MTTRDDLALAHRLADAAGAAIRPFFRARYDLEFKSDKSPVTEADKAAEAAIRAILEKERPRDGIIGEEYG

TTREDAERVWVLDPIDGTRAFIAGRPIFGTLIALMQAGWPTVGIIDQPVTGERWAGMTGQPTTFNGTAVI

TRACRALDQAIVASTAPQAFPGCTAEHFSRLAGQCRDTIWGGDCYNYGLVASGHVDIVVEAGLKLYDVAA

LVPVVEGAGGRMCDWSGDPLTADSDGQVIALGDPARLDDVLEALAEGHHQHG

>HisN_Sphingopyxis_macrogoltabida WP_054732155.1 histidinol-phosphatase

MTLESDIALAHRLADAAGAAIRPLFRSAWAHEAKADASPVTEADRAAEAAMRRLLDAEAPRDGIIGEEYG

AERTEASRQWVLDPIDGTVSFMAGRPIFGTLIALLQDGWPVLGIIDQPIAGERWVGAVGQPTLFNGRPVR

TRSCRTLGEAVLATTGPQYFSDHDGEHFMALAAKTSHKRMVFGGDCYNYGLLASGHIDMVVEAGLKLHDF

AALVPVVEGAGGTMCDWNGDPLNADSAGHVIALGDPARLEDVIEGLACNH

>HisN_Novosphingobium_sp._FSW06-99 WP_067615868.1 histidinol-phosphatase

MKLDVEIALAHRLADAAGAAIRPHFRAAGFERKGDQTPVTAADRAAEEAMRALLRAEMPGDGVIGEEFGA

QDGSSGRAWVLDPIDGTTGFVAGRPLFGTLIALIVDGFPVLGVIDQPILKERWVGVIGQPTLFNGAPVRT

RPCPALAEAAIATTGPHYFSDHDGEHFMALAAQTDHKRMVMGGDCYNYAMLASGHLDVVCESNLKIYDWA

ALVPVVEGAGGVMADWNGEPLRQGSSGHVLALGDPARLDDVVEALACGH

>HisN_Erythrobacter_atlanticus WP_048884690.1 histidinol phosphate phosphatase

MVSNVDISLMHRLADVAGEEIRPFFRTIMDIDAKEDATPVTLADRNAESAMRKLIEAEAGADGIHGEEFG

VKNGASGRQWVLDPIDGTTAFLAGRPIFGTLIALLEDGFPVLGMIDQPILGERWLGAVGQGTTLNGQTIH

ARSCPDLRRATIATTGPHYFSVEQGDRFMALAGQTDHKRMVMGGDCYNYACLAGGHIDIVAEAALKLHDY

AALVPIVEGAGGIMCDWAGEPLHAQSDGTVLALGDPARLEDVLEVLG

>HisN_Phaeospirillum_molischianum WP_002725349.1 histidinol-phosphatase

MNACPLEFISLAQRLADAARPVVRTYFRTPIAVDTKADDSPVTIADREVEAAMRAILDVEYPGHGILGEE

QGQRDCDAEWVWVLDPIDGTAAFITGKPSFGTLIALAHRGRPVLGIIDQAFTDERWLGVAGQPTTLNGQP

VSVRACPDLAHARAFTTAPELFDDATRPGWDRIAARVRGVRYGCDCYAYALVATGFVDLVVEAGLKPYDY

SALVPVVEGAGGLMTDWQGQPLTIWSEGRVVAAGDARSHAEALAVLAG

>HisN_Azospirillum_lipoferum WP_085554926.1 histidinol-phosphatase

MTETCPAHLVALAQRLADSSASVIRRYFRTPFSIDDKADASPVTIADREAERIIRTIIEAQRPDDGIHGE

EYGTKNLDAEWVWVIDPIDGTKSFIAGRPIFGTLIALLRHGRPVLGVIDQPIVGDRWVGVAGRPTTHNGQ

TVRVRPCPGGLAASMFGTTSPDLFPGADYEAYRRVAAQVKTSAYGGDCYTYGLLASGYYDLVVESGLKLY

DFAALVPVVTGAGGLMTDWRGNPLDANSTGQVVAAGDPRTHREALAALAG

>HisN_Thalassospira_mesophila WP_085579094.1 histidinol-phosphatase

MDPYDQFVKIAHRLADAARPVVRKYYRTAVAVDVKADASPVTIADREVETVMRAILAEECPDHGILGEEH

GRQNIDAEYVWVLDPIDGTKSFIVGKPSFATLIALCHNGTPVLGIIDQAITDERWVGVTGQQTTFNGSPI

ATRPCPALDQAIFFTTAPELFRGPQILAYETIRNRCRQPMYGVDAYAFGLTALGLADVVVEANTQAYDFC

ALVPVVEGAGGKMSDWQGNPLTLHSDGCLVATGDRQCHEQVLTITKAAMP

>HisN_Enhydrobacter_aerosaccus SKA27337.1 histidinol-phosphatase, inositol monophosphatase family

MTGSVPAELVALAHRLADAARPIVARHFRTPVAIDDKSDKSPVTIADRDAETAMRTLLTQHVPAHGVFGE

EHGAERTDAEYVWVLDPIDGTKAFITGLPIFGTLIALLHRGQPVLGVIDQPILGERWLGVSGQASTFNGK

PITVRSCPTLDRAYMYSTAPIMFPGDLERRHMALAKQVKLFRWGGDCYAYGLLAAGHVDLVVENSLKLYD

FAALVPVIKGAGGLITDWQGRELDMHSDGSVLAAGDPAVHRAAQQALAG

>HisN_Altererythrobacter_atlanticus WP_046904000.1 histidinol-phosphatase

MNLTAETALAHRLADAAGEAIRPHFRSAVEAERKGDSTPVTIADRAAEEAMRRILTAEFPQDGIHGEEFG

VSEGRSGRQWVLDPIDGTTAFLSGRPIFGTLIALLVDGFPVLGMIDQPILGERWLGVAGAPTTFNGKPVK

TRACRDLSDAALATTGPQYFSQEEGDVFMALAAKTDHKRMIMGGDCYNYGLLASGFLDVVCEAGLKLHDY

AALVPVVEGAGGTMCDWNGDPLHGGSGGHVLALGDSARLEDVLEAMGGHHHH

>HisN_Magnetospirillum_magnetotacticum WP_009869890.1 histidinol-phosphatase

MTACPAPFIALAEKLADAARPVVKKYFRTPVAVDDKADASPVTIADREVEAAMRAILAAEVPSHGILGEE

HGAEHCDAEYVWVLDPIDGTAAFITGKPSFGTLISLAHNGKPILGLIDQAFTHERWLGAMGRPTTLNGQP

ARVRACPDLAHAYAFTTAPELFCEATRPGWDRIAKACKRPRYGCDCYAYALLSSGFCDLVVEAGLKPYDF

AALVPVVEGAGGIMTDWSGKPLSIHSDGRVCAAGDARLHAEALRVLNQ

>HisN_Blastomonas sp._CCH5-A3 WP_066213869.1 histidinol-phosphatase

MIHSKDIELAQRLADAAGEAIRPFYRADFTTDRKADASLVTEADRAAEAAMRAILDAEAPRDGVIGEEYG

EKPGTTGRVWVLDPIDGTTSFIAGRPIFGTLIALISDEWPVLGVIDQPIGRERWVGATGRGTTFNGRPVK

ARLCPEIGDAVLATTSPHLFSDHDAMHFMALAKLCDTRRLIWGGDCYNYALVAQGSVDLVCESGLKLHDF

AALVPVVEGAGGHMCDWNGDPLHKGSDGRVVAMGDPARLEDVLEALAQHDH

>HisN_Croceicoccus_marinus WP_066841999.1 histidinol-phosphatase

MRLTDDIALAQRLADAAGAAIRPHFRTALESDRKGDASPVTIADREAEQAMRAILAAERPGDGIIGEEFG

SERAGASRQWVLDPIDGTAGFLAGRAIFGTLIALMVDGWPLIGVIDQPIAGERWVGGSGLPTLLNGAPVR

CRPCARLDEATLATTGPHYFDDHDGEHFMALAAQTDHKRMVMGGDCYNYAMLASGHLDIVAEAGLKLYDY

AALIPVVEGAGGMMTDWNGDPLNADSDGHVLAVGDSARLEDVIEALACDH

>HisN_Porphyrobacter_mercurialis WP_039097007.1 histidinol phosphate phosphatase

MRLTAELALAHRLADAAGDAIRPHFRTGLDAERKGDASPVTIADRAAEEAMRRILTAEVPQDGVHGEEFG

TSEGRSGRQWVLDPIDGTASFLVGRATFGTLIALLVKGFPVLGIIDQPIQRERWVGVTGQPTTLNGVAVR

TRPCRELSGATIATTGPHYFTADEGDTFMALAQKTDHRRMIMGGDCYNYGLLAAGHLDLVCEAGLKLHDF

AALVPVVEGAGGLMCDWNGEPLHGGSAGHVLALGDPARLEDVLEAMGGGDGHDH

>HisN_Skermanella_aerolata WP_084720917.1 histidinol-phosphatase

MTASSPSTEPPVALPDPIVLPDGVADLAVRLADAAGSVIRRYYRTPFAVDDKPDSSPVTIADREAEAAIR

AILAAERPGDGIVGEEHGTTNGDADWLWVIDPIDGTKSFITGRPTFGTLVALLYRGRPVLGVIDQPITGD

RWVGAVGQPTTLNGKPARVRACPSLGRATLNTTSPDLFGPDDYTAFRRVATAVKLPMYGGDCYAYGLLAA

GFVDLVIEAGLKLYDYAALVPVVEGAGGVMTDWSGAPLGRGSDGRVVAAGDAAAHAEALARLGEAPLG

>HisN_Beggiatoa sp._IS2 OQW92214.1 histidinol-phosphatase

MSVSPALIGLAQRLADASATIVRDYFRVKLTIEDKADASPVTIADRAAEQMMRQLITEVYPNHGIFGEEL

GTLHRDADYLWVLDPIDGTKSFISGKPLFGTLISLLYQGKPILGIIDQPILRERWLGGEGLPTTLNGLPV

QVRTCPDLSQAILYATSPHMFTGNDALAFNRLREKVKFPLYGCDCYAYALLASGFIDLVVEASLSPYDYC

ALIPIITGAGGIITDWQGQPLGLQSDGRVIAAADSRIYQQALDQLIL

**ImpA orthologs used for motif identification**

>ImpA_Corynebacterium_glutamicum |19553292|ref|NP_601294.1| fructose-1,6-bisphosphatase [Corynebacterium glutamicum ATCC 13032]

MDARGMLAIAEAVVDDAEALFMQGFGAAPAHMKSPGDFATEVDMAIESHMRSMLNMMTGIAVIGEEGGGA

TSGTRWVIDPIDGTANFAASNPMSAILVSLLVDDQPVLGITSMPMLGKRLTAFEGSPLMINGEPQEPLQE

QSSLVSHIGFSSMASPRNTAFPVELRRDLLTELTESYLRPRITGSVGVDLAFTAQGIFGACVSFSPHVWD

NSAGVMLMRAAGAQVTDTEGHPWAPGRGVVAGTKRAHDVLLSKIEKVRLMHADAGNDQSLNEEYK

>ImpA_Corynebacterium_diphtheriae |504076584|ref|WP_014310578.1| fructose 1,6-bisphosphatase [Corynebacterium diphtheriae]

MTDVRDLYHIAEAILDDAERLFIQGIGSAPTTFKNGGDFATDMDLKIEQYLRTQLVMMTGIPVFGEEYGG

KLGTPMWVVDPIDGTANYAAGNPMSSILISLIADGEPVIGLTSVPMVGQRFGAYADSPLLLNSQVQPQMS

ARDQRHVSHVGFTSIASPRESSFPTVVRQGLLGALAQTYLRPRITGSVGIDLAYAAAGIFDAALSLSPNL

WDNAAGIMLVRAAGGVVTDLDGNQWTPTSQGVIVGSARSHEVLMATIDTMR

>ImpA_Corynebacterium_resistens |503654132|ref|WP_013888208.1| inositol phosphatase [Corynebacterium resistens]

MSGLDTRALLAVAEAAVDEAETTFSAAVGAEPEVIKSPGDFATEADLTVERQLRTLLTQYTGLPVHGEEF

GTVLPGEQPVERIPADDMADGLDGPRRRRKNQTGTEELPETYWVVDPIDGTANYAVGNPFACILVSLVHQ

GDIQLSVTEMPLLGKRITARRGHGLFVDGHPARPMPPSDPGVTQISFGSILSQRRGNLPISYRQDMLNEI

GKSYPRMRVTGSVGIDLAFTAAGVFGGTVTFSPNLWDNAAGILAVQENGGVATDFAGNPWKPGVSGLVAG

EPEVHATLLQHIQSVPIGTAARTAQDVMDRGGIR

>ImpA_Corynebacterium_pilosum |517408110|ref|WP_018580563.1| hypothetical protein [Corynebacterium pilosum]

MVDTRALLTVANDIVSHASGMFVKGVGAAPSLYKKDGDFATEMDLAIERFIREELAKESSIPVFGEEQGG

KFNPEACWIIDPIDGTTNYATGNPNCSILVSLVIKNEPRIAVTAMPLFDKHLSTRDDEPVYLNGKELPEL

VDDSSGGLIGLGSVGSPDSDKFPIEFRLKLMGWLTDTHLRPRITGSVGVDLALVASGTFKAAMSFSPNMW

DNTAGVLLARNAGAVVTDGLGRPWSPTSPGAIVGTRDAHATVMDTIMTILKL

>ImpA_Corynebacterium_humireducens |749040183|ref|WP_040086168.1| fructose 1,6-bisphosphatase [Corynebacterium humireducens]

MDARELLAVAEAVCDDAERMFLAGVGSDPAAMKSPGDFATEVDLAIEAYLRQTLTQFTGIPVLGEEAGGC

YSHDAVWVVDPVDGTANFAAGNPMCAILISLLVEDQPVVAITAMPLLGRRLTAYEGGPVRLNGQPITPLT

EANQLVAQVGFSSVASQVRSQFPSLLRQGLLAELSATYLRPRITGSVGVDLAFTAQGIFGGAVSFSPHVW

DNAAGVMLVRANGGVVTDIEGNEWTPRSIGVVAGTARAHQAIMSTMDGILNS

>ImpA_Turicella_otitidis |490737796|ref|WP_004600104.1| fructose 1,6-bisphosphatase [Turicella otitidis]

MAEARDTAGATTGLLRLAEEAVASVEPVFLDGLGARPKTMKGAGDFATEKDLEIEELLRHRLGEGSGLPV

LGEEHGGEPDRERYWVVDPIDGTTNYATANPLCAILVALVDGGRPVVAVTAAPTLGIRLSAAAGGRLRDR

GEELPPREESPDVMAQVGFSSISSHSHPTPSEDRRLELLDELGRSYLRPRITGSIGLDLSFAARGFYGAA

VSLSPHVWDNAAGALLVEAAGGLATDLAGNPWRVGADGLVAGAPRAHAEVLRRVEQVRRRGGGAG

>ImpA_Dietzia_cinnamea |494905968|ref|WP_007632012.1| inositol monophosphatase [Dietzia cinnamea]

MSDQTGNTLPTDLPVDPAEALALATRVLDEVTPRFVEGVGAPGVQNKGARNDFATELDLELERRISDALR

EGTGLEVHGEEFGGPPVNEGTLWVVDPIDGTANYSLGIPTAGILVALVHERQPVLGLTWLPLLGLTFTSI

AGGPLMENGVESPRMSDVSIRDVALGLGSLNTGARTTYPPAYRREVFEQLTLDAARTRKFGSTGVDLSFV

ASGRLSAAVSFGNYAWDNAAGASHIRAAGGVVTDLTGGPWSIDSPSLLAAAPRAHAEVLDTVRQLGEPGN

FFEAGRRRSTPEPESPRPWLAGER

>ImpA_Dietzia_alimentaria |498228353|ref|WP_010542509.1| inositol monophosphatase [Dietzia alimentaria]

MHDQTGNVLPTNLPVDPAEALALATRVLEDVTPRFIEGLGAKGVQSKGSRNDFATELDLELERKISGALR

EGTGLEVHGEEYGGPPVNEGTLWVVDPIDGTANYSLGIPTTGILVSLVHERQPILGLTWLPLLGMRFTSV

AGGPLQENDRELPALADVSIRDVGVGLGSLNTGARATYPPAYRREVLERLTLDSARTRKFGSTGVDLSFV

ASGRLSAAVSFGNHAWDNAAGASHIRAAGGVVTDLAGEPWSVESQSILAAGPRAHAEVLDRITQLGDPYN

FSEAGRRRSTPEPESRRPWLDSGR

>ImpA_Dietzia_sp._UCD-THP |516447157|ref|WP_017836069.1| inositol monophosphatase [Dietzia sp. UCD-THP]

MTDQTGNTLPTDLPVDPAEALALAARVLEEVTPRFVEGLGAEGTRVKGSRNDFATELDLELERRISDALR

EGTGLEVHGEEYGGPPVNEGTLWVVDPIDGTANYSLGIPTTGILVSLVHERQPVLGLTWLPLLGLRFTSL

AGGPLKENDEEIPRIADVSIRDVALGLGSLNTGARTTYPPAYRREVFEQLTLDAARTRKFGSTGVDLSFV

GSSRLSAAVSFGNYAWDNAAGASHIRAAGGVVTDLAGEPWSIDSQSILAAGPRAHSEVLGTIRELGEPGN

FFEAGRRRATPGPDGNRPWLDGER

>ImpA_Gordonia_otitidis |494447995|ref|WP_007240045.1| inositol monophosphatase [Gordonia otitidis]

MTTPSGRDLPSLLEVAGSILDGVSAEFVTGLGAPSAVAKGGTDFATQVDLDLEQRIRRELTERTGIAVHG

EEFGGPPVTDGAIWVLDPVDGTYNYSTGMPLTGILLGLLIDGEATLGLTWLPLTGTRYAGHVGGPLYCNG

VPTGPPPASALAQTAVAFGPFNVSHGGRYPGARRAQLLEALSVRAARLRMTGSTGVDMAYVAAGIFGAAI

AFGAHAWDNAAGAALVRAAGGVATDLAGNPWTVTSPSLVTAMPDVHRELMAVIDEVVGDERTSR

>ImpA_Gordonia_namibiensis |493922670|ref|WP_006867633.1| inositol monophosphatase [Gordonia namibiensis]

MSATESSLDLPRLLDTAGSVLDAAVETFVEGLGAPSAVHKGGTDFATQVDLDLERRISAELEDRTEIPVH

GEEFGGADPVDGPVWVLDPVDGTFNYSAGMPLTGILLGLVVDGEATLGLTWLPLLDRKYAAHADGPLMIN

GEVADPLPDLPLEEAAIAFGPFNAAGRGRYAGDRRADLLRALSSRVARIRMTGSTGVDMAFTASGVFGGA

VAFGRHPWDNAAGAALVRAAGGVATDIAGERWTVASPSLVAGTPTVHAGLMAVIEETVGEWAK

>ImpA_Gordonia_amarae |491331298|ref|WP_005189259.1| inositol monophosphatase [Gordonia amarae]

MSAGLDYARLLHTAGSILDGAVEEFRAGVGAPSAVAKSATDFATQVDLDLERRLSAELADQTQIPVHGEE

FGGPDLDSGAVWVLDPVDGTYNYSTGLPLTGILLGLLVDGDPVLGLTWLPLQNRRYAAHVDGPLLVDGEP

VAPLADTELATSVLAYGPFNAAHGGRYSGDVRADVLRGLSSRVARLRMTGSTGVDLSFTAAGIFGGAVIF

GRHAWDNAPGAALVRAAGGIATDLSGNPWTVTSSSLVVGAPGLHAELLDVITDASRGEWKDGE

>ImpA_Mycobacterium_tuberculosis |490008765|ref|WP_003911572.1| inositol 1-monophosphatase ImpA [Mycobacterium tuberculosis]

MHLDSLVAPLVEQASAILDAATALFLVGHRADSAVRKKGNDFATEVDLAIERQVVAALVAATGIEVHGEE

FGGPAVDSRWVWVLDPIDGTINYAAGSPLAAILLGLLHDGVPVAGLTWMPFTDPRYTAVAGGPLIKNGVP

QPPLADAELANVLVGVGTFSADSRGQFPGRYRLAVLEKLSRVSSRLRMHGSTGIDLVFVADGILGGAISF

GGHVWDHAAGVALVRAAGGVVTDLAGQPWTPASRSALAGPPRVHAQILEILGSIGEPEDY

>ImpA_Mycobacterium_kansasii |738449690|ref|WP_036400875.1| inositol monophosphatase [Mycobacterium kansasii]

MDLAAQLTPLVEQASAILDAAVPRFLDGHRADSAVRKKGNDFATEVDLAIERQVVDALVAATGIGVHGEE

FGGTPVDSPWVWVLDPVDGTFNYAAGSPMAAILLGLLHHGDPVAGLTWLPFIDERYTAVTGGPLMKNGVP

RPRLAPAKLADSLVGVGTFSADSRGRFPGRYRLAVLENLSRISSRLRMHGSTGIDLVYVADGILGGAISF

GGHVWDHAAGVAQVRAAGGVVTNLAGDPWTPAARSVLAAAPGVHDEILDMLRDTGQPEDY

>ImpA_Mycobacterium_triplex |738520344|ref|WP_036468821.1| inositol monophosphatase [Mycobacterium triplex]

MDLHALLAEASTILDAAVEPFVAGHRADSAVRKKGNDFATQVDLAIERQVVAALQAATGIGVHGEEFGGT

PVDSPWVWVLDPIDGTFNYAAGSPMAAILLGLLHDGEPVAGLTWIPFTDDRYTAVAEGPLVKNGVAQPSL

THTPLAEGLIGVGTFNADSRGRFPGRYRLAVLERLSRVSSRLRMHGSTGIDLVYVADGILAGAVYFGGQV

WDHAAGIAQVRAAGGTVSDLAGRPWTPASRSVLAAAPGAYEEILEILRSAGRPEDY

>ImpA_Amycolicicoccus_subflavus |752812317|ref|WP_041451641.1| inositol monophosphatase [Amycolicicoccus subflavus]

MSQSQLEELVRLAGEILDAASVRFREGIGAPSAVNKGGRDFATALDLELEKQIGGELRKRTGIPVLGEEF

GHGEKSAGGVPPDLLWVLDPIDGTLNYSAGLPMAGILLALLDNREPVAGLTWLPMLNEKFVALSAGCLIR

NGKDQPRLERVDVRESIIALGSFNLDSAGVYPGEYRQEVIGEISRVCSRLRLQGCTGADLAYTAAGSLGA

AISFGHHVWDHAAGAALVRAAGGIVTDLHGNPWEAGSPSLLAASPGVHEQLIAIIRAVNARTGFTQEALD

AE

>ImpA_Nocardia_rhamnosiphila |917108213|ref|WP_051714925.1| inositol monophosphatase [Nocardia rhamnosiphila]

MTGTESELATLLTTAAEILDGVSGRFLEGRGAPSAVAKGSRDFATELDLELERTISARLVDRTGIEVHGE

EFGGPALTSGTAWVLDPIDGTFNYSNGHPLTGTLLALVRDGEPVLGLAWFPPLGRRYAAVAGGPLLLDGK

PQPPLAPAVLDEAMIGFGAFNIESTGRVPGLFRHRVLGTLSALSSRVRMHGSTGIDLAFTAEGTLGGAIV

FGHHPWDNAAGALMVRCAGGVVTDLEGVDWTIESGSVLAAAPGVHAELLDMIAATRGSGYTAVSDRVDTA

GPRGEEER

>ImpA_Nocardia_vinacea |750406974|ref|WP_040688877.1| inositol monophosphatase [Nocardia vinacea]

MTANRLDTELPALLDVAAEILDAASPRFVEGVGAPSAVAKGRNDFATELDLELERTISAQLEQRTGIEVH

GEEFGGPQLTSGTAWVLDPIDGTFNYSSGHPLSGMLLALVHDGEPLLGLTWLPLLGQRYSAAVDGPVLLN

GEPLPRLPHGKLGEAMIGFGAFNVDSAGRIPGRFRFDLLGPLSRLSSRVRMHGSTGIDLAFTASGVLGGA

IVFGHHPWDNAAGVALVRAAGGVVTDLAGDPWTITSGSVLAAAPGVHEELLEMICTVADDA

>ImpA_Nocardia_testacea |916557433|ref|WP_051164524.1| inositol monophosphatase [Nocardia testacea]

MTGTDSELGTLLATAAEVLDGVSARFLEGRGAPSAVAKGSRDFATELDLELERTISAQLVERTGIAVHGE

EFGGPALTSGTAWVLDPIDGTFNYSNGHPLTGTLLALVRDGEPVLGLAWFPPLGRRYAAVVGGPLLLDGK

PQPPLAPAVLDEAMIGFGAFNIESTGRVPGLFRHRVLGALSALSSRVRMHGSTGIDLAFTAEGTLGGAIV

FGHHPWDNAAGALMVRCAGGVVTDLEGVDWTIESGSVLAAAPGVHAELLDMIAATRGSGYTPVSDRVETA

GPRGGEGNR

>ImpA_Rhodococcus_wratislaviensis |739376560|ref|WP_037237477.1| inositol monophosphatase [Rhodococcus wratislaviensis]

MTLSRDPRELLAVASDLLDGVHDRFVSGVGAPSAVHKGPDDFATAVDLELEKRLTGELRDRTGIDVHGEE

FGGPDLGSGPVWVLDPIDGTFNYSAGLPTAGTLLALLDDGVPVLGLTWLPLVGRRYAAVADGPLLEGGTP

LPALGRTSLASSIVGLGALNIDSRGRIPGAYRLQVLAQLSRVSSRIRIHGSTGVDLAFTAAGILGGAVVF

GHNAWDNAAGVALVRAAGGVVTDLGGKPWTVTSDSVLAGAPGVHGEILDILGSLGDPRSEPSGGRTQ

>ImpA_Rhodococcus_fascians |739244595|ref|WP_037107673.1| inositol monophosphatase [Rhodococcus fascians]

MSLPEDPQRLLEIAGRLLDGTHDRFVAGVGAPSAVQKGPDDFATAVDLELEKRLSAELFEHTGIAVHGEE

FGGPDLSTGTVWVLDPIDGTFNYSAGLPSAGTLLALLHDGVPVLGLTWLPLVGHRFAAAAGGQVHLNGEA

LPRLDSRALSDSMIGFGAFNIASRGRTPGLYRVRILEELSKVSSRLRMHGATGVDLAYTASGVLGGCVVF

GHRAWDNAAGALLVQAAGGVVTDLAGEPWTVDSTSVLAASPGAHDELLKVITDLGDPADFLEGRVR

>ImpA_Rhodococcus_equi |491655956|ref|WP_005512675.1| inositol monophosphatase [Rhodococcus equi]

MSPSRTPLDLLAIASEVLDGARERFVEGLGAPSAVRKGPKDFATAVDLELEKSVSAELERRTGIEVHGEE

FGGPELASGDVWILDPIDGTFNYSAGLPIAGILLALLRDGVPVLGLTWLPLTGERFAAAEGGPVYRNGVA

LPPLERSELSSAMIGFGAFNLDSRGRIPGTYRFELLGRLSRVSSRIRMHGSTGADLAYTAAGILGGAVVF

GHHVWDNAAGVALVRAAGGVVTDLRGDDWHVGSRSVLAAAPGVHGELLDILHSLGDPETRPEGGPPQ

>ImpA­­_Smaragdicoccus_niigatensis |750348691|ref|WP_040630609.1| inositol monophosphatase [Smaragdicoccus niigatensis]

MSLTRDPQELLGIASEVLDLVWDRFVDGLCAGSAVSKDNADFATSVDLEVERIVSAELRERTGIPVLGEE

FGGPDIHAGTTWVLDPIDGTFNYSAGISLAGTLLALVHEGRPIIGLIWMPLTGEKYAALEGGPVMKNGKP

LPMLEPRDLATSLIGFGVFTPPMAQFLSPAFRLELFNKLNAKTDRIRISGSTGIDLAYTAAGALGGSIVF

GFHPWDNAPGALLVQAAGGIVTDLAGEPWTIESTSMVAAAPRVHDELMDVIASTHCSDKSLRRISDN

>ImpA_Tsukamurella_paurometabola |754551146|ref|WP_041944937.1| inositol monophosphatase [Tsukamurella paurometabola]

MLATAGAVLDAVAPRFIEGVGAPASVAKGPADFATDLDLELERRIAGDIADATGIPVHGEEFGGPPVDQG

TVWVLDPIDGTFNYSAGFPATGTLLGLVHDGRPVAGLAWFPLVADAISGHVEGPVYNRGEALAPLPERSL

VDSMIAVGSFSRGSNGHFPGDYRFAILGEVSRRVARVRQFGSTGVDLAYTATAVFGGAVVFGHHPWDNAA

GAALVLAGGGTVTDLAGTEWHTGSDSVLAGGPGVYDELLDIVASVGAPSDYRGEPGHPGILSY

>ImpA_Tsukamurella_sp._1534 |518032849|ref|WP_019203057.1| inositol monophosphatase [Tsukamurella sp. 1534]

MAAFPAGFTGDPDALLAAAGRALDEAAPRFVEGLGAPASVDKGPADFATDLDLELERRIAGAVADATGVA

VHGEEYGGPAVDEGTVWVLDPIDGTFNYSAGYPATGTLLGLVHDGRPVAGLAWFPLVGDAFAGHVEGPVR

NRGEALPPLREGSLVDSMIAVGSFSRGSRGRFPGDYRFEILGEVSRRVARVRQFGSTGVDLAYTASGALG

GAVVFGHHPWDNAAGAALVLAAGGVVTDLAGGEWHVGSDSALAAAPGVYDELLDIVRSVGAPDDYRPAQG

KR

>ImpA_Williamsia_sp._ARP1 |797062286|ref|WP_045823040.1| inositol monophosphatase [Williamsia sp. ARP1]

MASTVVGTLPADLDPPRLLSIAADLLDEITPRFVEGLGAPSAVEKGGNDFATELDLELERLLSARLSDAT

GIAVHGEEYGGPDVTSGTVWVVDPIDGTANYSAGLPLAGTLVALLHDGAPVLGLTWLPLFDRRYAAFVDG

PVVCNGEELPPLRTTRLEDSMIAFGAFNVDSRGRYPGQWRVDILGQLSRRVSRLRLLGSTGIDMALCAAG

NIGGAVCFGHHAWDNAAGAALVLAAGGEVTDLAGAPWTVSSPSMLVGAPGVHAELASIIAEVGPPEGRS

>ImpA_Williamsia_sp._D3 |565879679|ref|WP_023960973.1| inositol monophosphatase [Williamsia sp. D3]

MTATDVPGFPADLDPNRLLATAADVLDSVTARFRDGLGAPSAITKGHTDFATEFDLELERIISAELQERT

GIGVHGEEYGGPDLTEGTVWVVDPIDGTFNYSAGVPVAGILLALLHNGLPVLGLTWLPLSDLKYAGFVGG

GLTCNGKLLDPLPTLRLDEAMLAFGAFNLDSRGRYPGSFRVELLGELSRRTARLRLFGSTGFDLALTAGG

QINGAISFGHHAWDNAAGAALVLAAGGQISDLAGEPWTVNSPSILAAGPGVHGELVELISQLGDPRAFEV

PKEKYR

>ImpA_Cryptosporangium_arvum |737890385|ref|WP_035856891.1| hypothetical protein [Cryptosporangium arvum]

MSLDASQLRELAGTAAQILDDSVPFFVDEIGAKEEVVKGVGDWATAADLALERTITAALAERTGLPMHGE

EFGGHDLHTGVTWVLDPIDGTANYSARIPLTGIALALLEEGRPVIGQIRLPLFGESYQAIDGGPLERNGE

ALPELAPARLEDVTIALGNVAPKGTHGGPTPRYPFRFRMALANAVAHQAMRVRMFGSSAVELAWASAGAV

GASLNFGNHAWDNAAGALMVRQAGGVLRDLEGDEWSLRSRSILVGRRGVVDELSELIAALGDPASYA

>ImpA_Micromonospora_sp._CNB394 |648576103|ref|WP_026267854.1| myo-inositol-1-monophosphatase [Micromonospora sp. CNB394]

MLYRELLPVALDAVGRAATLMRRRAPVTSASKGDRDLVSNVDYAIERDLRSFLRDATPGIAFLGEEEGVS

GAGDLQWTLDPVDGTINFVRGLPLCGISLGLLHGTRPALGVVDLPFLGARYWAAEGCGAFANGEPVKVSD

TTSLSEAVVGIGDFAVGTDAANKNRIRLALMARLAETVQRVRMSGSAAVDLVWLAEGKIDAAITLSNHTW

DMTAGVAIAREAGAVVVDSRGAEYSAESTTTLAVTPGLVGQMLPHLRENGDGVSR

>ImpA_Micromonospora_sp._M42 |585248937|gb|EWM68469.1| inositol-1-monophosphatase [Micromonospora sp. M42]

MGAEPATVTSYRELLPVALDAVGRAATFMRRQAPVTSTSKGDRDLVSDVDYAIERDLRSFLRDATPGIAFL

GEEEGVSGTGDLQWTLDPVDGTINFVRGIPLCGISLGLLHGTRPALGVVDLPFLGARYWAAEGCGAFANGD

PVKVSDTTSLDEAVVGIGDFAVGADAANKNRIRLALMARLAATVQRVRMSGSAAVDLVWLAEGKIDAAITL

SNHTWDMTAGVAIAREAGAVVVDISGAEYSAESTTTLAITPGLVGQMLPHLRENETA

>ImpA_Hamadaea_tsunoensis |653096071|ref|WP_027346274.1| myo-inositol-1-monophosphatase [Hamadaea tsunoensis]

MTDLQAMLAIAVTAADRAADVIRTHAPAELTAKGDRDYASDVDFRVEREVRAFLANATPGVGFLGEEEGH

TGTSGSLHWALDPIDGTVNFANALPLCAVSLALVDGDKPIAGVVHLPFLGNVYTAAAGQGAYRRGKPITV

ASPAKVHDAIVSIGDYAVGDEADSRNVPRLRLTQRLAGKALRVRMFGSAAIDLTWLAEGITGAMVMFSNK

PWDTAAGVIIAKEAGAAVVDIDGTPHTMQSSATIAAAPSIVDEVLDIVQSAAASTTS

>ImpA_Streptomyces_aureofaciens |703127831|ref|WP_033354778.1| hypothetical protein [Streptomyces aureofaciens]

MSPDLPVLLDLARSVTERAEQQLRDLTPDLITAKGDRDMVTNVDLAIERQARTALLDGAPGIGFVGEEEG

GDTGADTRWIIDPVDGTANFIRGLPLCGISLALVHHGVPVLGVIHLPLIGRRYWAAQGLGAFRDGQRISA

AGTSTLPEAMIAVGDYGTGPDGPERNRVALAIQARLAEKAQRVRMLGSAAVDLAFVADGTLDATITLGNH

DWDMAAGVVLAREAGAVVMDTDGSPHTPDSRTTIATTPGLRDAVLDILRVTTTGTAWSATSGSRAC

**SuhB orthologs used for motif identification**

>SuhB_Corynebacterium_glutamicum |41326086|emb|CAF20249.1| MYO-INOSITOL-1(OR 4)-MONOPHOSPHATASE [Corynebacterium glutamicum ATCC 13032]

MEQQSFNELRAIAAETATLTAVRIRDKRAELTNLWDYTNTKSSTVDPVTIVDTLAEDFIANRLQELRPKD

GLIGEEGTGTASISGVTWIVDPIDGTVNFLYDLPQYAVSIAAAIDGEVVAGAVINVVTGVLYTAARHEGA

TKYLPERDEIVPLKASAATVVSESLVATGFSYSALRRSLQADLLTKILPTVRDIRRMGSAALDLCHLADG

QVDIYYEHGLNCWDFAAGSLIAAEAGAFVRAPGLSIPGSSGEICFGAAPGVFDDANAHFDVVGAFKALDR

>SuhB_Mycobacterium_tuberculosis |15609838|ref|NP_217217.1| inositol-1-monophosphatase SuhB [Mycobacterium tuberculosis H37Rv]

MTRPDNEPARLRSVAENLAAEAAAFVRGRRAEVFGISRAGDGDGAVRAKSSPTDPVTVVDTDTERLLRDR

LAQLRPGDPILGEEGGGPADVTATPSDRVTWVLDPIDGTVNFVYGIPAYAVSIGAQVGGITVAGAVADVA

ARTVYSAATGLGAHLTDERGRHVLRCTGVDELSMALLGTGFGYSVRCREKQAELLAHVVPLVRDVRRIGS

AALDLCMVAAGRLDAYYEHGVQVWDCAAGALIAAEAGARVLLSTPRAGGAGLVVVAAAPGIADELLAALQ

RFNGLEPIPD

>SuhB_Corynebacterium_diphtheriae |504074404|ref|WP_014308398.1| hydrolase [Corynebacterium diphtheriae]

MAKTSLKEHIVEQLSVRVDSLREYSELAAIAAEVACQASDHIRRTMRELGDVTARMETKSSDVDPVTIVD

KAAEHFIADALSHLRPGDGLLGEEGAESSSSTGITWIVDPIDGTVNFIYGIPQYAVSVAACIGENVVAGA

VINVATSDLYVAACGSGAFVLRSDSDALKSIEASKCSDLQHALIATGFSYSSRRREDQAKLLVSLLPNVR

DIRRFGSAALDLCAVAEGCVDGYYEHGLNAWDFAAGALIAQEAGAKIRRPELKLASSAGALLLASSSTIF

RELELQFYPFDMRK

>SuhB_Segniliparus_rotundus |502902916|ref|WP_013137892.1| inositol monophosphatase [Segniliparus rotundus]

MQSSTKKSASASAQDYALLRGIACDVATSAAEMIRVRRAEVFASPGGRDSDSVRSKTSETDPVTIVDVES

ETLVRESLARLRPGDGFLGEEATGNTEGASETGVVWVVDPIDGTVNFMYGVPAYAVSIAAQIDGKTVAGA

VVDVPQGWVYSAALGAGATVSREGFGTRALHVSETESLGMALVSTGFGYKAERRIAQARLAGTLLPVVRD

LRRIGAAALDLCLVAAGSLDAHVEHGLNPWDFAAGSLIAQEAGALVRIPPTSARSHEGELVLIATPKVYH

ELAGVLEEIGALGPIE

>SuhB_Rhodococcus_rhodnii |498788338|ref|WP_010836474.1| inositol-1-monophosphatase [Rhodococcus rhodnii]

MNAPPPTTRPDTEGLDVGALRDVAVEIARTAADHVRRRRGELFGFAGTAHATSETVSAKTSATDPVTVAD

RESEDVVRSLLAQRRPGDAVLGEEGGGAASDDGVTWVVDPIDGTVNFLYGIPAYSVSVAAQVGGVSVAGA

VVDVVADTVYCAARGSGATAITAAGERVALTCNPVDDLSLALVATGFAYSAARRARQGELVAALLPRVRD

IRRIGSAALDLCMVASGRTDAHYEHGLSVWDWAAGALVAEEAGAAVRVPAATAPGSDGRLVVAAAPGIAD

ELAKVFTELGTDAPIPA

>SuhB_Nocardia_asiatica |916311657|ref|WP_051046703.1| inositol monophosphatase [Nocardia asiatica]

MEGVPETSSSVSDYTSDSFVSIVARGDEADLRRVAVDIAETAAAHVRARRPEVFGPGGAHAEGAVQSKSH

ATDPVTIVDTETEDLIRRLLAEQRPGDPILGEEGGGSIDDTSADVVHWVVDPIDGTVNFLYGIPGYAVSV

AALRGGRPVAGAVVDVARAATYSAGLGLGALRVDADGVVEHLSCTPVDTVAMALVATGFAYGRHRRSRQA

ELAAQVLPHVRDIRRFGAAALDLCHVAAGRVDAYYEHGLNAWDWGAGALIAAEAGARLTLPPATAPGADG

DLVVAAAPGIAGELGELLARVGATEPIPDR

>SuhB_Smaragdicoccus_niigatensis |516909241|ref|WP_018160734.1| hypothetical protein [Smaragdicoccus niigatensis]

MFTSNPPTGRLSAVATSREFALDLQRVAIEVGLEAAAHVRRRRVELYSGPREPDFASTKSSPTDPVTIVD

TESEEVIRKALNSRRPGDSVLGEEGGGPANTDAAVQWVVDPIDGTVNFMYGLPAYAVSIAAAHQGVFVAG

AVADVNSGETYTAALGAGAHRISPTGEWTPLRVNTPSSLQMSLVATGFGYAASRRAEQGRIVAGLLPHVR

DIRRLGSAALDLCLLAAGQVDAYYEHGLNVWDWAAGALIAQEAGAAVELPAADAAGNAGHIVAASAPSVA

EAFANTLRDLNAVGPIPV

>SuhB_Amycolicicoccus_subflavus |503572488|ref|WP_013806564.1| inositol monophosphatase [Amycolicicoccus subflavus]

MCCDGRVPEDSNIADLATDLRVVAEEVAMEAAAFVRRRRPEVFGAPGGCVATGAVQSKSTPTDPVTIVDS

ESEQLIRELLRKYRPDDAILGEEGGGDAKDTVGVRWVVDPIDGTVNFMYGIRAYAVSVAAQIDGRSVAGA

VVDVAADLLYSAATGQGATCRQGSSGAADPLSCSAETSLSHALVATGFSYSAQHRRRQGELVASILPVVR

DIRRIGSAALDLCMVASGQVDAHYEHSLNPWDWAAGSLIATEAGAVVKVPAATESGTSHVTVAAAPGIAT

EFLSLLEDKGALEPL

>SuhB_Kutzneria_albida |662166632|ref|WP_030111521.1| myo-inositol-1-monophosphatase [Kutzneria albida]

MVVAQTEALVEVAVQVAAEAAELVLATRGNAVTQVDTKSSETDVVTAADRAAEQLVRDRLAQLRPGEPVL

GEEAGGTQRLDGLVWVVDPIDGTVNYLYGLPWYAVSLAAQLDGVSVAGAVVEPVSGRVWTGLRGGGAWLD

GRPLRASAARRLDLSLLATGFAYATERRQRQAEVMGRVVGQVRDLRRNGAASLELCAVAAGWLDGYVEHG

LNRWDWAAGALIAEEAGAVVRLPGSDPEGLGAEATFAAAPGIAEQLRAVLVEAGINQV

>SuhB_Frankia_alni |499922506|ref|WP_011603240.1| inositol-phosphate phosphatase [Frankia alni]

MSDLPDPDTLLDVALTIAREAGGLLARGREGAVAAETTKSSPTDVVTALDRASEALVARRLGELRPGDGL

LGEEGSDSAGSTGVRWIVDPLDGTVNFLYRLPNWAVSIAAEVDGEVVAGVVHAPALGTTYTAVRGGGAFR

DGTALTGSTVTTLAGALVATGFGYLESRRAAQAAVLTRVVPRVRDIRRMGAASLDLCAAADGLVDAYYER

GLKPWDHAAGGLVAAEAGLRVGGLHGRPATEDLTVAAPPALFGPLTDLLAEDPPADRD

>SuhB_Tsukamurella_paurometabola |502891540|ref|WP_013126516.1| inositol monophosphatase [Tsukamurella paurometabola]

MEDVEHDRQQLSEIAVAVVREAAQRVVRRRAEVFGANAFEADPEEAVSTKSTPTDPVTVVDTETEHFIRE

RLAQVRPDDTVLGEELGGRSGEPGTVRWIVDPIDGTVNFLYGVPAYAVSLGAQIDGVSVAGAVADVVQGS

VYAAALGAGAREILADGTERTLRANPIVDPALALVATGFGYARERRERQGRVLAAVLPQVRDVRRIGSAA

LDLCMVAAGRADAHFEHGLSPWDWAAGSLIAAEAGATVIVPAPDSTSDQGALTLAAAPGIAEQLIEILRA

AGGLENL

>SuhB_Streptomyces_coelicolor |499340795|ref|WP_011030503.1| inositol-phosphate phosphatase, partial [Streptomyces coelicolor]

MTDVTPDPLRADLLKIAQEAAHRAGELLRDGRPADLAVAATKSSPIDVVTEMDIAAEKLITGLIAERRPD

DGFLGEEGAAMEGTSGSRWVIDPLDGTVNYLYGLPTWAVSIAAEQDGERVAGVVVAPMRGESYHAVLGGG

AWATGAWEGERRLVCRSAAPLDQALVSTGFNYVADVRAEQAEIARRLIPLVRDIRRGGSAAIDLCDVAAG

RLDGYYERGLHPWDLAAGDLIAREAGAVTGGRPGERPTGALTIAATPAVFEPLQRLLTDFGA

>SuhB_Actinoplanes_utahensis |759833220|ref|WP_043528954.1| inositol-phosphate phosphatase [Actinoplanes utahensis]

MPVVSTPGELLEIAVRVAREAAATARRMRDEAIGDVETKSTDTDVVTAADKAVERQVVRALAAERPGDGV

LGEEYGDSAEVAPGAVRWILDPIDGTVNYLYGLPQYAVSLAAERDGEVIAGVVINAATGDEWTATRGGGA

WRAGRRLSGSVRTTLDQALVGTGFGYDARRRAHQGTVLARLITRVRDIRRFGAAALDLCLAAEGSLDAYF

EKGLNLWDHAAGGLIAAEAGLIVSGLDGAPAGRDMLVAAPPALFPALHAALAELDAAGGP

>SuhB_Dietzia_sp._UCD-THP |516448804|ref|WP_017837716.1| hypothetical protein [Dietzia sp. UCD-THP]

MSEQTVEGLAGRDHPVDVDELRMFADRLAVSGGGVARRMLEQFDSTGLDPRAKSSATDPVTVIDTTVERH

LRELVTTERPGDRVLGEEGGEEGGEEGGEEPPAAGRGVVRWVVDPVDGTVNLLYGIPFTAVSVAAEVDGV

VLAGAVHNIVTGETWTAAVGRGSSLRSAAGTVTGLTASACDDLSMALVGTGFSYDADVRAEQGRAVAALL

PRIRDIRRCGSAALDLCMVATGRIDAYFERCLKPWDHAAGALIAAEAGAMVRVAADDEVPTVAAAPGVAE

DFLAALEGLGGPVSS

>SuhB_Ilumatobacter_coccineus |505252755|ref|WP_015439857.1| inositol-1-monophosphatase [Ilumatobacter coccineus]

MPSSVVPPSATEGDHLVVVAVEVARLAATLVRASVGAAASTRTKSSPTDVVTATDVESEALIRRELLERC

PGSTIVGEELDDDRGRNGVGWIVDPIDGTVNFLYDLPVVSVSIAATVGGVTVAGAVVDIVRDETFSAALG

RGARRDGVPVRARDTVALDQALIGTGFSYSSERRAQQSEILNRLLPVSRDIRCMGSAALNLCWVGCGRLD

GYYEHDTKLYDHAAGDLIASEGGARVDDPASNGEGLSIAASAAIFDELRAVVDG

>SuhB_Ferrimicrobium_acidiphilum |737407618|ref|WP_035388767.1| hypothetical protein [Ferrimicrobium acidiphilum]

MVGVNHELLPLALDALAEARTSLATQSRRVIDTKSSDTDPVTAADRALERAIRETIAKQRPHDSFIGEEY

GLTRQAQSNTRWIVDPIDGTVNYSYSIPSYAISIAAEVNGRIEVGLVFDVGHNELFQAVRGQGATCNGEP

IRPSDQTELAQALCGTGFGYSAATRKLQAQVLLEVIDEIRDIRRFGAAAIDICWAGAGRIDGYFETGLKV

WDYAAASLVATEAGANFITDLPGIGDDGLTIVAGPALFEALQARIAGLYRQANS

>SuhB_Nitriliruptor_alkaliphilus |919112101|ref|WP_052667744.1| hypothetical protein [Nitriliruptor alkaliphilus]

MMSMTTPPLELAVDVAHRAGALLVRYAAELRDGVDLGVSTKSSASDPVSEADRAAERLIAGGLLDARPDD

GLLGEEGQASRPGTSGYRWVVDPLDGTVNFLYGRPTWCVSIACEDADGTVVGVVHAPQLGETWTATRGGG

ARRHETGHVLAVSEVTELGECLVATGFSYDPLVRADQGRAVADLVPRARDVRRDGSAALDLAWVAAGRAD

GYVEAGLNPWDWAAGRLLVTEAGGRVSTASWTLGGRSRAAVVAGGAAAHDHLLAWLEERP

>SuhB_Solirubrobacter_soli |921290498|ref|WP_053227047.1| hypothetical protein [Solirubrobacter soli]

MAGVASGGGGVTQGELRALAESVAREAGALLREAFRSPDLRVSSKSTPTDLVSEADHNAERLIRERIGSA

RPDDGVLGEEGGDHRGTSGVRWVVDPLDGTVNFLFGIPQWAVSIAAEDEHGTLVGVVYDPTRDELFSAER

DGNPTLDGRPITASTKSDMATALVGTGFGYDAEVRRAQSVVAARLLPEVRDLRRFGAAAIDLAWTACGRL

DAYYEHGLNPWDLAAGGLICERAGLDVRPLEPVGPSNPGVLVAPSSLADALEPWLA

>SuhB_Acidothermus_cellulolyticus |500039522|ref|WP_011720240.1| inositol-phosphate phosphatase [Acidothermus cellulolyticus], 179

MTAMGADVAELAALAVAVAREAGEFLRTGSDHALEIATKSSPTDVVTEMDTAAEQLIRRRIHERRPADDI

LGEEGGGGAASGAGGVRWIVDPIDGTVNYLYGIPQWAVSIAVEVDGVIVAGVVFDPCKDELFTAIRGEGA

RLGDRPVQVRECHELGQALVGTGFGYAASRRARQAEVLTGVLPVVRDIRRLGSAALDLCAVACGRFDAYF

EQGLNLWDFAAAGLIAAEAGARVAGLRGAPPSNRLVLAATPGIFDALQELLAALGADEVETHDSASPLSY

FA

>SuhB_Actinomyces_neuii |636804371|ref|WP_024331212.1| hypothetical protein [Actinomyces neuii],

MESFALPISEATRDGLWALAKDVAIEAAQLAEKRKSEGKVTVAATKSSPVDPVTEVDREVEALIRQKISA

ARPEDGIMGEEAGIDGGKSGLFWIVDPIDGTVNYIRQVAPAAVSVACVYGSVDPTTWVPVVGAVAEIGTD

RLYHAGAGGGAYLGESPIGASSLTRLAPALVATGFGYVRERRQRQGKFLASLIGDIADIRRCGSAALSLC

AVASGTVDIYYEEGLKPWDMAAGELICAEAGATVSDGCSGRPSAKMLTAAPPELANIFNQRISSIYWE

>SuhB_Actinopolyspora_erythraea |693611071|gb|KGI80860.1| myo-inositol-1-monophosphatase [Actinopolyspora erythraea], 163

MTHQYDVAGLRATSVTVAREAAELALTMRSEIATTGVVATKSTETDVVTAADRAVEKLIRERLSELRPDE

PVLGEEEGGAAAEDGLRWVVDPIDGTVNYLYGYPNYAVSIAVQLDGASLAGAVVEPCSGRVWSAAAGQGA

ELDGRPLRVSDSGRLDLALLGTGFSYLRQRRIRQASLLRELLGEIRDIRRGGAASLELCAVAAGWLDGYY

EHGLSRWDWAAGALIAAEAGARLRLPGEGTTDGLGDEAILCASPGIADELTAVLLKHEAAGI

>SuhB_Catenulispora_acidiphila |502437511|ref|WP_012785840.1| inositol-phosphate phosphatase [Catenulispora acidiphila]

MSDNADPKELLELAVRLAAEAGRLLVEDRPRDLGVAETKSSPTDIVTVMDQRSEKLIVEGILAERPDDGI

LGEEGSERAGTSGVRWVIDPVDGTVNYLYELPMWAVSIGVEIDGEMAAGVVEIPMLRERFTAMRGHGAFL

NGEPIRAFSAQSEGKPVPLERALVATGFGYAAQRRAVQGAIVAELMPLVRDIRRGGSAAIDLCSVACGRV

DAYYERGAKPWDLAAGALIAQEAGAMVGGLYGAAASDEMTVAAADGLFQALREFLEGRNAARDS

>SuhB_Gordonia_effusa |494527809|ref|WP_007317261.1| inositol monophosphatase [Gordonia effusa]

MTESRQLRTDADDLALVAIELAQTAAAHVRRRRPELFGSTTVYESSDTASGSAAVSTKSTPTDPVTLADT

ETEALIADLLAQHRPGEEMLGEEGGGSRDIPSGVRWVVDPIDGTVNFMYGIPAYAVSVAAQINGVSVAGA

VVDVAREITYAAAAGSGAYRHAGGLRERLRVSGIDDPSLALVATGFAYDRQRRAQQAQIIAALLPRIRDI

RRVGAAALDLCMVASGAVDAHIEHGLSPWDWAAGGLIAAEAGAVVTLPPANSRATDGHPTIALSPGVAQE

MTRVLDEVGALVDLPSR

>SuhB_Modestobacter_marinus |754178858|ref|WP_041797147.1| inositol-phosphate phosphatase [Modestobacter marinus], 182

MTSGSTAPDLRTELLELARATAEEAAALVGAGRSTAADQVDTKSSPVDVVTAVDTASEALIVRRLTDARP

DDGVLGEEGAAREGTSGVRWVVDPIDGTVNFLYGFPAYAVSIAAEVDGRTEVGVVLNVATGELFTAARGR

GAWLTAPGAATMQLHGSGPVSLEQTLVATGFGYRVEQRRAQGAVVAQLVTEVRDIRRNGCASLDLCSAAA

GRVDAYYELDLKPWDHAAGALVAAEAGLVVTGTEGRPFAEPMAVVAAATIAEPLVALLERLH

>SuhB_Jiangella_alkaliphila |820817443|ref|WP_046770088.1| inositol-phosphate phosphatase [Jiangella alkaliphila]

MTADPADLLTLAEKTAREAGDLVRDRREAVERMAVAGTKSTPTDVVTESDTAAEALIRSRLFAARPDDGF

VGEEDGVVLGSTGVDWVVDPIDGTVNYLYGIPQYAVSIAAQVDGVVRAGVVHNPASGETWTAVLGGGALL

DGRPVRVSACTSLSLALVGTGFGYDAARRSRQAAVLLEIVPIVRDIRRAGAAALDLCAVASGRLDAYYER

GLNPWDLAAGGLIAAEAGAVVGGLHGAPAGNDLAMAATPGVAAELTALLERLGADHD

>SuhB_Kineococcus_radiotolerans |500725840|ref|WP_011981822.1| inositol-phosphate phosphatase [Kineococcus radiotolerans]

MNEELREVAVAVALAAGELVSAGRPDRVEVASTKSSPTDVVTAMDTASEQLLRTELARVRPEDGFLGEEG

GHRPGRSGLTWVVDPIDGTVNYLYGLRAYAVSVAVVRAADPAARPDPATWTVLAGAVVDPSQGETWSAAL

GGGARLTDARGTRALTAPTGPELGQALVATGFGYDARRRAEQAAVVARLLPRVRDLRRVGAASLDLCSVA

AGRVDAYYERGLKPWDLAAGALVAAEAGAVVTDFTGAAAGGHGLVAASRDLHPHLLRALADAGAGDPGVG

DPGVDDPGTDDGA

>SuhB_Beutenbergia_cavernae |506362711|ref|WP_015882430.1| inositol-phosphate phosphatase [Beutenbergia cavernae]

MGVTGAPTPAETADLVVLASELARAAGDVAARMRAERVDVASTKLNANDVVTRADLAAETLLRERIAAAR

PHDGILGEEGARAPSSSGLTWVVDPIDGTVNYLYGLPMYSVSVAVVVGPADDLREWTPIAGAVHAPGLGR

TYAAGRGLGATRDGEPIAPSATEDLASALVGTGFHYDPVLRERQGALAASLVGRVRDLRRIGSAALDLCF

VAEGALDAFYEERLNPWDVAAGVLVAAEAGARVQHLPLAGELLTLAAAPGLDEALAARLGDLARDLG

>SuhB_Cellulomonas_carbonis |918373526|ref|WP_052425801.1| inositol-phosphate phosphatase [Cellulomonas carbonis]

MGPVQTRPQDLGHLTPDEVAALAVLAERAAREAGALVRGGRPDRVDVSATKSSPVDVVTEMDLASERLLV

QVLLGERPDDGVLGEEDGLRAGTSGVTWVVDPIDGTVNYLYGVPAYAVSVAAVVGDPDPATWTVVAGAVH

AVPDGRTWTAARGHGARLDGRALTIGEPRPLAQSLCGTGFGYRAERRRAQARVLGHVLPLVRDIRRIGSA

AMDLCSVASGQLDVYYERGLNPWDLAAASLVAEEAGAAVVGLRGTRAGAAMTVAGHPDTVPALVEVLEAA

GADSDEPA

>SuhB_Kytococcus_sedentarius |752622075|ref|WP_041291436.1| hypothetical protein [Kytococcus sedentarius]

MDRLVAAVTAQEVAGLRGLAARLAREAGERLVAGRPEEVSVADTKSTATDVVTAADRASERWLRDELRRL

RPGDAVLGEEYGDVPEEAAAPSASVPEEAAAPGDPVPAGAPGAGDGPRLTWVLDPIDGTVNYLYGLPAWG

VSVACVVGDPQVPGAWTPVAGAVSAPVWGELFHAGTGQGAVVEGPRGGPRELAVSEPSDPAQALVATGFG

YAAERRAVQGRVAAELLPRVRDLRRMGSAAIDLCCVAAGRADAYYESGTKVWDHAAGLLVVTEAGGRVTG

LDGSPPGAPMVVAGGPAMTRWLHDELVRLGA

>SuhB_Mobilicoccus_pelagius |497132718|ref|WP_009483280.1| inositol-phosphate phosphatase [Mobilicoccus pelagius]

MTPTTESPVPQEELRELEELAVSVAAEAARFVVGSRAAELEVTTKSSDVDIVTVMDARSQELLGRRLREA

RPADGFFGEEGGADAAAAGATSGLTWVVDPIDGTVNYLYGFPQYAVSVALVECDPTTPGGWRPIAGAVAD

AVAGSVHHARLGGGAWTREPDGTEAPLHVSDATALATSLVGTGFGYDAAKRGRQARALVEVLPRVRDIRR

GGSAALDLCHVAAGRLDGYYEMGINPWDMAAGWIVLTEAGGSCTGAEGAPPSTDCVVAAAPGIREALTGL

VDEVVLIVRND

>SuhB_Intrasporangium_chromatireducens |736708616|ref|WP_034713683.1| inositol-phosphate phosphatase [Intrasporangium chromatireducens]

METAVDPADLLELERLCVEFASEAGRFIRDERPSTVRVADTKSSETDVVTVMDRRSEALLHGLIRDARPD

DGILGEEGADVAGRSGLTWVVDPIDGTVNYLYDIPAYAVSVAVVVGDPATPGAWRPVAGAVSDPCRGLVH

HARRGGGAWTRAAATPAPPAGASPPPSPERRRLAASTEEELGRALLATGFGYEAQVRAAQAEVLREVLPR

VRDIRRIGSAALDLVRVADGSVDAYAESGLKPWDLAAGWLVAEEAGARVVGVAGHPGPELTVAAGPALVE

PVRRLFTGR

>SuhB_Jonesia_quinghaiensis |916590169|ref|WP_051197260.1| hypothetical protein [Jonesia quinghaiensis]

MSSSPKTPLHTDSQPTALELAQLCRDLAELAQQHIAQHRPPRASVLSTKTTPTDVVTQMDRDVEALLRDR

LRVLRPEDGFLGEESVDHAVGGTSGLTWVVDPIDGTVNYLYGLPSYSVSIAVVEGQPDPQHWSIVAGAVI

RVPDAHLWWAARGEGAWRDGQRLACSSPASLGECLTGTGFGYDATARLAQAQWLPHVLPKVRDLRRIGSA

ALDLCALAEGALDLYYERGLSPWDVAAGQLIAEEAGAVSRGLHTEYPTSRMTIVGSQARVDDLQDILRRV

VVDDLPMP

>SuhB_Clavibacter_michiganensis |780231001|ref|WP_045529883.1| inositol monophosphatase [Clavibacter michiganensis]

MTTPGDTALLTIARDIAVRAGELALRRRREGVEVAASKSSPEDIVTHTDRETEDLIRRALEDVRPDDGFL

GEESEGTAGTSGLTWVVDPIDGTVNFLYGIPAWAVSIAVVEGEADPLTWTARAGCVVNPTLGEVYTATAG

GGSALDGRPLAVNSGVPLSLALVGTGFSYGAETRMRQGRVITDLLGEVRDIRRIGAASLDLCNVAAGRTD

AYFERGLKPWDHAAGALIAAEAGARVTGIGGGPASDELLIAADPELARALEERLERPRA

>SuhB_Arthrobacter_siccitolerans |910251221|ref|WP_050055787.1| inositol monophosphatase [Arthrobacter siccitolerans]

MTGVTELLEVAKQAAAAGARVLAGRNADALHASNKGDAGDWVTAFDVAAENAVRDVIAAARPADSITGEE

HGTTRPADPTGYRWSIDPLDGTTNFIRNIVYYGTSVAVADADGAWLAGVVNAPALGRIYYAARGQGAWLE

EAGTLTRLEGPVPGRKGQILATGFSYDPLVRSEQAARFAGLLEGFADVRRLGSAALDLCMVADGTHDAFG

ERGLNEHDFSAGALIAEEAGCWVRRPRLTSPLDGGPTDQERLDAWTCAASLELSGKFPL

>SuhB_Isoptericola_variabilis |503604849|ref|WP_013838925.1| inositol-phosphate phosphatase [Isoptericola variabilis]

MSTDPAPEPTPSAAPSAAPSVAPPAAAPGELPDDATVAALRALAADLAVEAGRLVREGRPERVVVAATKS

SAVDPVTEMDRAVEELLRARIAAARPDDAILGEEGVDVAGTSGLTWVVDPIDGTVNYLYGVASYAVSVAV

VAGPADPARWTALAGAVHSVVDGRTWTAARGQGATCDGRALRINEPQSLGACLVGTGFGYAAARRAHQAR

VLTHVLPRVRDIRRLGSAAIDLCLLAEGGLDLYYERGLNPWDLAAGALVAAEAGAAVTGLRGEPAGTTMA

VAGAAPRVAELVRLLEDAGADGPDQEA

>SuhB_Ruania_albidiflava |551297527|ref|WP_022917553.1| inositol-phosphate phosphatase [Ruania albidiflava], 125

MEHLSEAQLAVDDVEADQLLELAVDAARRGGGLAESARRAGVDVAATKTNDLDVVTRADIEVENLIRETI

LAQRPQDGFLGEETDEVPGHDDLTWLVDPIDGTVNYLYGDPGYAVSIAAYRRGTPLVGVVYGPALDELWA

ARSGGGATLNGLPVRTAGSVSPAQPLLATVFDYDPTARVRQMRRLADSAVVIRDVRVSGSTALDLCRVAA

GRVDVFCTDSVHWWDVGAGIVIAREAGCQAWARQDPVSRHVSCVVSNPTVSESVLSSLGYAVVPSC

>SuhB_Pimelobacter_simplex |740893253|ref|WP_038678501.1| inositol-phosphate phosphatase [Pimelobacter simplex]

MTGPAGLRGLAEDVAGEAAALVREHAGRGVAVAATKSSEVDVVTAADRASEELIRRLILEARPGDGFLGE

EGDDVTGTSGVRWIVDPIDGTVNFLYGLPEYAVSVAAEVDGEVVAGVVVDVAKQVVYAGHRGGAATRNGR

PIAARGPAPLAHRLVATGFNYTRPVRTVQAAAAARLLPEIRDLRRTGSCALDLCRVAEGALDGYVEEGVH

LWDHAAGGLIAQLAGARLLVTVGAAGTEAVVCGPEHGFDELLSAVRTAGFLRE

>SuhB_Granulicoccus_phenolivorans |916608365|ref|WP_051215456.1| hypothetical protein [Granulicoccus phenolivorans]

MAETVAAAAAEQAARLRVAGVEVAGVKSSQTDVVTLADQQTETWIRDELARLRPRDGILGEEGNSVTGTS

GITWIVDPIDGTVNYLYAIPQWAVSIAAVEGDPDPATWTALAGAVANPGPGEVYAAVRGEGAYLGEHRLE

LEPKHDLGRALVATGFGYAAKRRAAQARMLTAVLPRVRDIRRMGAASLDLCAVATGRVDAYYEVGLKPWD

HAAGVLVAREAGAILGGPGGAGTPPLEAFAWACNPGLAEPFADLLTAARADIPEADW

>SuhB_Amycolatopsis_alba |522126180|ref|WP_020637389.1| Myo-inositol-monophosphatase [Amycolatopsis alba]

MADVGVDETLLKSVAERVAGEAAELVHEAWTGMNAGREVRVDTKSADTDVVTAVDHESERLVRARLAELR

PDDAVLGEEGGGSAGDGVTWVVDPIDGTVNFLYGLPWFAVSVAAQVGGVSVAGAVVEPVSGRRWSAARGQ

GAFLDGRRLSVNAPERLDLTLVGTGFAYKVERRAKQARFVAGLATRVRDVRRNGAASLDLCAVAAGWLDA

YVEHGLGQWDWAAGALIAAEAGALVSLPGEDIELGPDATFAVAPSIAAPLRQALIDSGAASI

>SuhB_Nocardiopsis_salina |516210581|ref|WP_017614544.1| hypothetical protein [Nocardiopsis salina]

MTTTKPAPDPDDLRALAVRVATEAGELAAQGQEGITVLDTKSSPTDVVTKMDRATEELIRSRLLAERPGD

AFLGEEEGGDEAGTAPVRWIVDPIDGTVNYLYGGPDWGVAIAAEVDGQIVAAAVAQPGRRRLYEAVLGGG

AFLDGERLAAPAAVPLDRALVATGFGYLPERRRRQAQVLLEVVPRVRDIRRTGSAAVDLCGLASGQADAY

YERGLNPWDWAAPGLVASEAGLRVAGARGGPPAEDLVVAARPELFEELEPLLMELGADRDD

>SuhB_Nonomuraea_coxensis |522031634|ref|WP_020542843.1| hypothetical protein [Nonomuraea coxensis]

MTGFLTLAEDIAREAGDMLLAKRPTMSAEIETKSSPTDVVTALDKASELLIRERIEAARPGDRILGEEGG

EAPGESDVRWIVDPIDGTVNFLYGLPDWAVSIAVEVGGRVVAGVVNVPVRGEVFTAALGEGAWLGGARLR

CNTGVPLPQALVATGFGYSQAGRAVQGEVVAKVLPRVRDIRRGGSCAIDLCSLAAGRVDAYYERGINPWD

YGAAGLVATESGARLGGLNGRPVSPDFAISAAPGLFEELHDLLVSLDPERDA

>SuhB_Actinomadura_oligospora |651284845|ref|WP_026416801.1| inositol-phosphate phosphatase [Actinomadura oligospora]

MTDPSALLELALDIAREAARMLVDKRPADLGVAATKSSPTDVVTEMDRASERLITERILAARPRDAFLGE

EGGSTGGDSGVRWVVDPIDGTVNYLYDLPDWAVSIAAEVDGVAVAGVVEIPRRGETYTAVRGGGALLRTA

SGVRELKVNTGVPLDRALVATGFGYFPERRAHQGEVLAGVLPRVRDIRRGGSCCVDLCSLAAGRVDAYYE

RGVQAWDVAAGALIVEEAGGRVQGLAGAPVSPELCIAAAPDLFDALHAVLEPLNPARD

>SuhB_Escherichia_coli |16130458|ref|NP_417028.1| inositol monophosphatase [Escherichia coli str. K-12 substr. MG1655]

MHPMLNIAVRAARKAGNLIAKNYETPDAVEASQKGSNDFVTNVDKAAEAVIIDTIRKSYPQHTIITEESG

ELEGTDQDVQWVIDPLDGTTNFIKRLPHFAVSIAVRIKGRTEVAVVYDPMRNELFTATRGQGAQLNGYRL

RGSTARDLDGTILATGFPFKAKQYATTYINIVGKLFNECADFRRTGSAALDLAYVAAGRVDGFFEIGLRP

WDFAAGELLVREAGGIVSDFTGGHNYMLTGNIVAGNPRVVKAMLANMRDELSDALKR

>SuhB_Mesorhizobium_alhagi |496110141|ref|WP_008834648.1| inositol monophosphatase [Mesorhizobium alhagi]

MPRSAILNVMVQAAMKAGRSLARDFGEVQNLQVSLKGPGDFVSQADHRAEEILHNELSRARPGYAFLMEE

RGVVSGEDDQHRWIVDPLDGTTNFLHGLPIFSISIGLERQGQIVAGVIYNPAMDELYTAERGGGAFMNDR

RLRVASRTKLTDTVIGTGVPHLGRGHHGNYLIELRNVMAEVSGVRRLGSAALDLAYVAAGRMDGFWEHAL

SPWDMAAGILMIREAGGFVSDPAGGAGMLESGSIVAGNEAIHRALLKTLKKPLAPR

>SuhB_Flammeovirga_pacifica |763366126|ref|WP_044223633.1| inositol-1-monophosphatase [Flammeovirga pacifica]

MNLKELTHKVVKLTETVGQFIANERKHFDSSKIEYKGLNDLVSYVDKTAETKLVEGLTKLLPDAGFIGEE

GTNKPSNNGYTWIIDPLDGTTNFIHGVPIFSISIALVTEDDYCIGVVREVNLDECFYAWKNGGAYMNGAP

IHVSKQEVLNAGLIATGFPYYDFEKMPQYINILKGMMEGSHGLRRMGSAAVDLAYVACGRFEGFFEYNLN

PWDVAGGGIIVKEAGGCVTTFSNKDSYVKDREIIAASPAIHKEMQSIIYKKWNS

>SuhB_Belliella_baltica |504584712|ref|WP_014771814.1| inositol-1-monophosphatase [Belliella baltica]

MIDLNQILDQTKSIAKEAGAFIRKERQHFDLKKVEEKGFNDLVSYVDKEAEKIIVNKLSKVLPEAGFITE

EGTREEENKTYTWIIDPLDGTTNFIHGIPVFCVSIGLMKDNEIVLGVVYEVNLHECFYALKGHGAFCNDT

PIRVSAAPSLSQSLIATGFPYSAFAQIDEYLEIMKVLMQKSHGLRRLGSAAADLCYVACGRMDAYFEYNL

NSYDVAAGSLIVQEAGGEVTDFKKGNDFLFGREIIAGNKPVHEGLWKELDQIWNK

>SuhB_Paenibacillus_ginsengihumi |750460530|ref|WP_040742403.1| myo-inositol-1-monophosphatase [Paenibacillus ginsengihumi]

MIVLANHTLGSKSFTAVAINTASKAGEWIQSKLGDYNSLHTKSAMNDLVTEVDKGAETMIRNLIQTHFPK

HGILGEESVEPGPDASRQALAKLSGAEYLWIVDPLDGTTNFVHGFPFFAVSIALAYKGEVIVGVVYNPVH

NELFVAEKGKGAYVRGRRLRVSAEAKLSDSLVATGFPPDKDHAAVNMRGLQALSGKVRNIRVNGSAALHM

AYVAAGRLSGFWEVGLNPWDIAAGSLLISESGGMVTDPLGQPYNLNVRNVVASNGLIHQQFIDELKAAEA

AE

>SuhB_Geobacillus_thermoglucosidasius |335360969|gb|AEH46649.1| inositol monophosphatase [Geobacillus thermoglucosidasius C56-YS93]

MKEKMAIKMKQDSEQALYDAAIEYALQAGRLIKYHLGNKGKIKPKKNAFDLVTEVDKLSEDFLRGKIQED

YPDHWILSEENCGQDNAYEVLKNHNSGYGWIIDPIDGTTNFIHNIPHFSVSLGIVKDGKPIIGVVFNPIT

GDLYAARKSFGAYLNGRPIRVGEESTLAEAVVATGFQASDFQSGSRVIQQIDKLAGKSRNIRMFGAASLD

LCLVASGKITGFWHEGLNPWDTAAGILVLAEAGGRVTDKDGNPYQLFHDSLIASNGKIHDELMKTIKL

>SuhB_Butyricimonas_virosa |652946287|ref|WP_027199765.1| inositol monophosphatase [Butyricimonas virosa]

MNIDLENTLALAVEWAKEVGEVQRSYFRSGHLELETKSTVHDVVTKVDKLSESMLIERIGKCFPNHSVLG

EESGEHDAHSDYLWVVDPLDGTNNYSQGLPVFCVSIGLQYRGETLLGVVYAPYLNELYTAIRGKGAFLNN

VPIHVSGKTELDRSVLATGFPYDKGIHPVNNIDNLSRILPHLRGIRRMGSAAYDLCGVAAGFLDGYWELG

LKLWDVCAGVLIVQEAGGHVEPFREDRGIAILAGNAGIVEKMKEYIS

>SuhB_Desulfatitalea_sp._BRH_c12 |783160832|ref|WP_045675364.1| histidinol phosphate phosphatase [Desulfatitalea sp. BRH_c12]

MNLESIKQVGVRAAYRAGKVLNRYYGSTLRVTKKGVIDLVTEADVAAEKIIVETIREVFPDHALLAEESG

ASGENSEHCWIIDPLDGTTNFAHALPFFAVSIAYARTDEVLMGIVFNPVNGEFFSAVHGQGASLNGTPIH

ASSTKTVSDSLLVTGFPYTVRTTPPTGLLEQFSRCLTSAQGVRRLGSAALDLCYVGCGRFDGFWEENLKP

WDTAAGLLVAREAGARVTDYADHPFNFYDKQILATNTHIHQEMVDLLTIEDLNDR

>SuhB_Ketogulonicigenium_vulgare |503150293|ref|WP_013384954.1| inositol monophosphatase [Ketogulonicigenium vulgare]

MAIGSANLNVMIKVARSAGRALIKDFGEVENLQASMKGPGDFVSRADRRAEETIRNGLMEARPSYGFLGE

ETGMIEGEDPTRRWVVDPLDGTTNFLHGLPHWAVSIALEHKGQIVAGVVYDPVKDECFYAEKGAGAWMNE

QRLRVSSRTKLLESVFATGIPFATQRTLPATLQDLARIMPTCAGVRRMGAAALDLAYVAAGRFEGYWERE

VKIWDIAAGMIIVQEAGGFVGPIRDDHDPLVHGDIVAANGEIFDKFAKLVRARPEAAIPTE

>SuhB_Bacillus_hemicellulosilyticus |569810104|dbj|GAE29690.1| inositol-1-monophosphatase [Bacillus hemicellulosilyticus JCM 9152]

MNKQWNERFEKIQEWVQIAGEEQVKRMDDELKIEQKSANIDLVTEMDVWTDEFFQENIRTFFPSDAILSE

EAGASAGESGYEWVIDPIDGTTNYAHRFPMFAISVAVKYEGETVVGVVHAPKLGESYTALKGKGAYLNGK

KIIVSERTQLTDSVIATGFPYDRATDPLNNVEQFNAVILQIGGIRRTGSAALDLCQVAAGRFEGYWEYKI

NPWDFEAGVLIVKEAGGVIVKKH

>SuhB_Cyclobacterium_amurskyense |873969087|ref|WP_048640908.1| inositol-1-monophosphatase [Cyclobacterium amurskyense]

MELQEITRKVEKLAKKAGQFIREESKNFNLAKVEQKGFNDLVSYVDKGAEEIIVKELAEIVPEAGFITEE

GTRSDNNKDLTWIVDPLDGTTNFVHGIPMYAVSIALARGEEIIVGVVYEINLDECFAAYEGAPSTCNGQP

IKVSGASELAQSLVAAGFPYDDGGKLEQYLQLLKYFIKNSHGLRRLGSAAVDLCYVACGRVEGYIEYNLQ

SYDVAAGTLIVKQAGGRVTDFSGGNNFIFGGQILASNTKLHDTFEKALAEKFVF

>SuhB_Pannonibacter_phragmitetus |907658434|gb|KND19737.1| inositol monophosphatase [Pannonibacter phragmitetus]

MARTAILNVMVQAAMKAGRSLVRDFGEVENLQVSRKGPGDFVSAADRRAEEIIRAELTKARPTYGLLMEE

SGETAGEDGQHRWIVDPLDGTTNFLHGIPVFAISIALERQGQIVAGVIFNPVMDELYTAERGRGAFVNDR

RLRVAARTALPDSLIGTGLPFLGRGDHGRALKELRYVMPEVAGVRRIGAAALDLAYVASGRLDGFWEHSL

NPWDIAAGILLIREAGGYVSDIDGKDTMLETGSVVAGNEFIKAQLQKLLKSAAE

>SuhB_Virgibacillus_halodenitrificans |635571219|emb|CDQ32724.1| Inositol-1-monophosphatase [Virgibacillus halodenitrificans]

MEPSQRLAEAERIARLAGERIVAARESQAFQQRYKSGDELVTDADVEIDRLIASELDAHFQDDARLTEEI

SPDRDVLEDNDAVWIVDPIDGTVNFAYGHPHVAVSIAWASEGKLRLGVVHAPFLGETFTALRGEGAWLNG

EPIQASNASELSRSLVATGFPYRRDARAPLFRRLAGVLASCRDIRRNGSAALDLCHVACGRLDAYYESVS

PWDFAAGLLIAREAGAKTGHLYQCPDRIPPELYGENILVGAPGIYNALNDILLKADEGRLDEIEAQN

>SuhB_Maricaulis_maris |499963800|ref|WP_011644518.1| inositol monophosphatase [Maricaulis maris]

MGTLSPLLTVMTDVASRAGRSLNRDFRDIEHLQVSRKGPADFVSVADHKAEDIIYDRLSQARPGYGFLME

ERGIVEGSDRSHRWIVDPLDGTLNFLHGMPHFAVSIALEREGELVAGVVYNPATDEMFHAEKGRGAWLAD

RRLRVAERKHFDEAVIATGTPYIGKPGQARFLKELHQIMPHCAGIRRMGSAALDLAWVAAGRYDGFWERN

LKSWDIAAGIMIVREAGGFVAEADGAENMLETGNIVAGNEPMLAEVVERLKKAAA

>SuhB_Hyalangium_minutum |763339982|ref|WP_044198301.1| histidinol phosphate phosphatase [Hyalangium minutum]

MEQETPAALRRTAEEGARLAGRVLAERFQGERTIEFKRSSIDLVTDADTAAEEVLLRFLRERHPGHSILA

EESGASRGSELRWLIDPLDGTTNYAHHVPHFCVSVAVDGPDGVLAGVVYNPMLDELFSAARGEGATLNGR

PLKASATTELERALLCTGFPYDVREKPDGPVGLLNHFIRRAQGIRRTGSAALDLAYVAAGRFDGFFEFNL

KPWDIGAGSLLVTEAGGVMSQIDGSPFDVMKGNVLAGAPGLAAVLQRECRQALANLGWDPRP

>SuhB_Halomonas_zincidurans |667762381|ref|WP_031383445.1| inositol phosphatase [Halomonas zincidurans]

MQLAERLAIATDIAEEAGREILAARANPSYEKRYKKGSELVTDVDVAIDRMISQRLEQHFPGEPRLSEEL

TPDRETLDQRGPLWVVDPIDGTVNFAQGLPHVAVSIGWALDGKIQVGVVHAPILGETFTAIRGQGAKRDD

RQIRASLASRLERSLIATGFPYRRDGRAPLLRRFSAVMQNCQDIRRCGSAALDLCNVACGRLDAYYESVS

PWDFAAGLLIAREAGARTGHVYACPEGITEDLYGENIVVAAPDIYSSLRKLLKDADEARHAGDDAAS

>SuhB_Flexibacter_litoralis |752595574|ref|WP_041264961.1| inositol-1-monophosphatase [Flexibacter litoralis]

MLDLPFLRKEVSELSEKVAEFIYKEAQDFDKDDIEVKSFNSLVSYVDKEAEKKIVERLRELLPQAGIIAE

EGTGTPKEEGFNWIIDPLDGTTNFVHGIPVYSISIALAFTEKKEGKTKTELLVGVVYEVSRKECFAAHQN

GGATLNGKKITVSGENELGKSLIATGFPYEDFDRIEDYLFMMGKMMKAAHGLRRLGSAAVDLSYVAAGRF

EGYFEYNLNSWDVAAGALLVKEAGGIVSDFEGNEIVENYIFGRQIIAATQIHSQILKLVKDVFLKK

>SuhB_Marinobacter_hydrocarbonoclasticus |504235677|ref|WP_014422779.1| inositol phosphatase [Marinobacter hydrocarbonoclasticus]

MSDSVSLQEIADFTEALAREAGELIRHEREENTLRTDYKHQTELVTHADVMADEFITGTIRERFPHHRIL

SEETMPDLSQAEELETPLWIVDPIDGTVNYAYGHPQVAVSIAYAEKGKVRVGVVHAPFPGETFRATESEG

ATLNNQPIRHSGATDPRQSLFATGFPYTKDNLEPLVRRLDAMIHQCRDLRRIGSAALDICWVACGRLDIY

YENVSPWDFAAARLIAKEAGATVGHFGEVPEGYPADLYGRDILISAPAVWAPVRSILRTASGYE

>SuhB_Indibacter_alkaliphilus |496323830|ref|WP_009033008.1| inositol-1-monophosphatase [Indibacter alkaliphilus]

MIELSNILKQTIDTAKTAGAFIRQESKTFDRNKIESKGLNDLVSYVDKEAEKIIVSQLREILPEADFIAE

EGTAGQSGKDYTWIIDPLDGTTNFIHGIPIFAVSIGLKYKDEIVLGVVYEVNFDECFYASKGNGAYLNEK

KIQVSKPKTLSESLVVTGFPYSAFSKIDNYLKILRSLMETCHGLRRMGSAATDLCYVACGRSEGFFEYDL

KPYDVAAGVIILQEAGGKVTDFEGDNDYLFGKTILASNSHVHTELMGEIQKVW

>SuhB_Myxococcus_fulvus |503704450|ref|WP_013938526.1| histidinol-phosphate phosphatase [Myxococcus fulvus]

MSQDSPEILRRTAEEGARLAGRILADRFLGERTIEFKGGIDLVTDADKASEEALLAFIRERHPEHAILAE

ESGATQGTDSLRWLVDPLDGTTNYSHRVPHFCVSVAVEGPGGVLAGAVHDPMLDELFSAARGQGATLNGR

PLRASTVSTLDRALLCTGFPYDVRERPEGPVGLFTRLILHAQGMRRTGSAAMDLAYVAAGRFDGFFEFGL

KPWDIAAGSLLVEEAGGVIRHISGAPFDVLRGDVIASAPALAPALLAEAKRFVDGLREQPPRG

>SuhB_Desulfococcus_oleovorans |501124321|ref|WP_012173433.1| inositol-phosphate phosphatase [Desulfococcus oleovorans]

MSVDIAYVRQVGIGAAFKGARALRAFFRGRFSVDKKGVRDLVTEADRASEKAIVDEIHYRFPDHAVLAEE

SGATGTRSEYRWIVDPLDGTTNFAHGLGLFCVSIAFAENGEVTAGVVLNPETGELFTATVDHGAELNGAP

IAVSGTSTLSDSLLATGFPYDVDKRLDPVMARLSRCMAASRGIRRLGSAALDLCYVACGRFDGFWEEGLY

PWDTAAGMLIARRAGATVTDFSGKAFVPEQKTILATNGLIHNEILKEMRE

>SuhB_Desulfatibacillum_aliphaticivorans |654864062|ref|WP_028316352.1| histidinol phosphate phosphatase [Desulfatibacillum aliphaticivorans]

MADFEKIEHTALAAAHGAAKVLLKYYGRARVVKKKGEIDLLTQADKASEEIIVEAIARAFPDHAILAEEG

SGKEADSEYTWIVDPLDGTTNYAHGLPLFSISIGLARAEKTIFGLVLNPVTQELFVAKEGQGATLNSRPI

SVSDQTDLQDSLVVTGFPYGLKTMMPELMDRFSAVLPKVQGVRRLGSAALDLCYVACGRFDGFWEQNLAP

WDTAAGECIVREAGGKVTDFAENEFSPGGSQILATNGKIHDAFLPLIC

>SuhB_Butyricimonas_synergistica |517147034|ref|WP_018335852.1| hypothetical protein [Butyricimonas synergistica], score 134

MNICLESTLELAIVWAKEVGEVQRSYFRGGHLDIETKSTVHDVVTKVDKLSESMLLERIGKHFPDHSVLG

EESGEHDLHSDYLWVIDPLDGTNNYSQGLPVYTVSIGLQYRGETVLGVVYAPYMDELYTAIRGKGAFLNG

KPIRVAAKTDLDQSVLATGFPYDKGINPINNIDNLSRILSHLRGIRRMGSAAYDLCCVAAGFLDGYWELG

LKLWDVCAGVLIVQEAGGTIEHFRYDRGIAIMTANPQLLQRIKEYIR

>SuhB_Cystobacter_violaceus |759681965|ref|WP_043399115.1| histidinol phosphate phosphatase [Cystobacter violaceus], score 134

MSDESPATLRRIAEEGARLAGKVLSERFPGERIIEYKGGIDTDLVTDADRAAEAAVLGFIRQRYPGHAIL

AEESGVSQGVGLRWVVDPLDGTTNYAHRVPHFCVSVGVEGPDGVLAGAIYNPMLDELFSAARGQGATLNG

RPLRASGCTELSHALLCTGFPYDVHQKPEGPMGLLRRFIVRAQGIRRTGSAALDLAYVAAGRFDGFFEFG

LKPWDVAAGSLLVQEAGGAMVRIDGAPFQVGVGDVLACAPGLADALITESKGFLTDLGWTPIPSPLGRGS

G

>SuhB_Thiothrix_lacustris |655039304|ref|WP_028488057.1| inositol monophosphatase [Thiothrix lacustris], score 133

MHPMLRKAIEAAREAGESIRHHANKVQKLDVENKAHNDFVSNVDREAEQIIVRLLQRAYPDHAFIGEESG

KHGPNSDYEWVIDPLDGTTNFLYGIPQYSVSVALKHNGRLLVGVVYDPLRDETFAAARGEGATLNGRRIR

VSERTTMQSALLGTGIPFRANQNLDLYLQTLKALLPDTAGVRRPGSAALDLAYVASGRFDGFWEFGLNEW

DMAAGVLLVQEAGGLIGDMRGDNTFLKTGDVVAANPKVFKEMIKRLHPVMAKR

>SuhB_Thermobaculum_terrenum |502638683|ref|WP_012875162.1| inositol monophosphatase [Thermobaculum terrenum]

MQKELEVAIEAAYKAGKVLRSMFEKGVQVHYKGRVDLVTTADFESEKTIIETIHKAFPNHGIYAEESGIK

EGTQPLRWIIDPLDGTTNFAHNFPLFAVSIALEHEEEMILGVVYNPISDEMFTAARGEGSRKNDKPIQVS

DTEDLIHSLVVTGFPYDRSEVSSIIELWTYFTLHAQGVRRLGSASLDLCYVATGQMDAYYERYVFPWDIA

AGAIIVQEAGGKVTDFYGGKFTSYKNEIVASNGKLHEAMINVTSRV

>SuhB_Paracoccus_sanguinis |738847076|ref|WP_036736425.1| inositol monophosphatase [Paracoccus sanguinis]

MASANLNVMIKAARKAGRSLVKDFREVENLQVSSKGPGDFVTRADREAERIIKEELRVARPNYGWCGEET

GTEDGEDPTRRWIVDPLDGTTNFLHGLPHWAVSIALEHKGEVVAAVVFDPAKDELFTAEKGDGAFVNDKR

LRVSGRRDMNSAIFATGVPFGGRGTLPATLRDLARLMPVCAGVRRWGSASLDLAYVAAGRYDGYWERGIN

AWDVAAGWLLVREAGGFVEPLRAHEGAGVDAGDIVAANAQLFAGFAEIVRSRD

>SuhB_Halanaerobium_saccharolyticum |750172930|ref|WP_040477004.1| inositol monophosphatase [Halanaerobium saccharolyticum], 130

MVLAKKMAINVGKMQKQKLRKNNFKINTKSTMSDLVTEIDLLSEDMIRAKIRNNYPEHNIMGEENKFEDK

KSDYTWVIDPLDGSNNYASSYPIYCISIALKYKNEVVMGVIYIPEFDEIYSAIKGKGAYKSGKAINISHK

TALRHSLIATGFPYDKNKSKIDNLAPFNKILKEIRGLRRSGSAAFDLVSVASGRIDAFWEFKLKEWDYAA

GELLVREAGGKVYQSEIEGAPLLIAGSKELVSELRKIIEDIYL

>SuhB_Microvirgula_aerodenitrificans |655049522|ref|WP_028498105.1| inositol monophosphatase [Microvirgula aerodenitrificans]

MHPMLTIAVKAARRAANIIQRASNNLDLIRAEQKQHNDFVSDVDRAAEAAIIDMIRDAYPKHAILAEESG

GASLSDAEYLWIIDPLDGTTNFLHGYPQYCVSIALAHKGQIQQAVVYDPNRNDLFTASKGGGAFLNDRRI

RVSKRISLADSLISTGFPYTDVSYLDQYLAMFADMIRKTAGVRREGSAALDLCYVASGRVDGYWELNLKS

WDIAAGSLIAQEAGAIVTDVFGEQGWLESGDVVAANPKVLAQMLHTLAPHVR

>SuhB_Methylomonas_methanica |503585474|ref|WP_013819550.1| inositol monophosphatase [Methylomonas methanica]

MPITLETLEKIVRAAGSVAMTYFNDLDQLAINKKSARDLVTDADVAVENYLKQALHEHSPEYGFWGEESG

KTANQTSRWIVDPIDGTHSFSKGQYFWGVSVALEIDGELLLGAVYGPALDDYYCAEKGKGAFKNGKPIRV

SDETSLATSMVSTGFACLRQYLEDNNLARFGRIAQATTGQRRLGSAALDLCTVADGQVDAFWEQELNLYD

VAAGALIAMEAGGTVTDFKGNPGVFPKQILATNGKILDQILPLM

>SuhB_Vibrio_litoralis |653847587|ref|WP_027696763.1| inositol monophosphatase [Vibrio litoralis]

MHPMLNIAIRAARMAGNHIAKSLENTDKIESTQKGQNDFVTNVDKEAEQLIIDTIKKSYPDHCIVGEEGG

SIEGKDTDVQWIIDPLDGTNNFVKGFPHFSVSIAVRMRGKTEVACVYDPIRNELFTAQRGSGAQLNNARL

RINPIKDLNGAILATGFPFKQKQHAESFVKIVGGLFSECSDFRRTGSAALDLCYLASGRVDGYFELGLKP

WDIAAGELIAREAGAILTDFAGGTNYLASGNIVASSARGVKHILKHVREHSNEGMMK

>SuhB_Glaciecola_pallidula |492855696|ref|WP_006009650.1| myo-inositol-1(or 4)-monophosphatase [Glaciecola pallidula]

MHPMLNIAVRAARSAGNVIARGFETFDDLQIEQKGENDFVTKIDREAEQTIIYKIKQSYPEHTFVGEEGG

IVAGDDDYKWIIDPLDGTTNFIKGIPHFAVSIALQYKGRLDQAVVFDPIRGELFTASRGNGAQLNGHRIR

ASQAKELGNTILATAFPYKNKTSLNEYMSSFNKIFAECGDIRRGGSAALDMAYVAAGRFDGYWERGIKPW

DIAAGELLVRESGGLVTDFAGGNDPLLSGEIVAGSPRVVQAIVKRLK

>SuhB_Meganema_perideroedes |517460858|ref|WP_018631604.1| hypothetical protein [Meganema perideroedes]

MYAPSANLKIMVDAARKAGRGLMRDFAELENLQASVKGPGDFVSAADRKAEASIKKDLMTARPAYGWLGE

ETGEEKGADETRRWIVDPLDGTTNFLRAIPHWAVSIALEHKGEIIAGVIYDPAKQDLFVSEKGGGAWLND

RRIRVAPTRDLSRALLGTGVPFGAKNTLPQTARELTRLMPQTAGLRRMGSAALDLAYVAAGRLDFFWERE

INAWDMAAGLLLVREAGGILADLSGGDDPLASGWVAAGTPDLFEKIREHLI

**Additional HisN orthologs checked for HolPase motif 5 but not used for motif definition (position of motif 5 indicated by coloring)**

>HisN_Ilumatobacter_nonamiensis |916327940|ref|WP_051062986.1| hypothetical protein [Ilumatobacter nonamiensis]

MSADMDGVDLEGELELALRVADAADLFTLPHFVDRDFTVDWKQNQTEVTEVDRHAESLIVDGLVRARPDHGVFGEEHGLTGATDSEWRWVIDPIDGTSGYVRGIPVWATLIALTHAEAGAVLGVVSAPALGMRWWGGVGLGAHTAAHDATREMRVSSVTLLDEAQLSVTHNSGWDDLGLTDRLIALQQRARRSRGMGDFWQHMLVAEGAVDVAIDAVGVAPYDLAAVKPIVEAAGGRFTDRLGEVTHEHDTAISSNGALHDEVLALLA

>HisN_Actinomyces_dentalis |916678761|ref|WP_051285852.1| histidinol-phosphatase [Actinomyces dentalis]

MRRYPRGMSTPATPASPTTERRWREDLDLAHAIADQVDPVTRAHFERQDFEVQTKPDLTPVTDADRQAERMIRESLSRARARDCVLGEEFGQTGRSPRQWVIDPIDGTKNFVRGVPVWATLIGLVEDGEVVVGLVSAPALERRWWAVAGGGAWTGRSLSASRRLRVSGVSGLEDASLSYASLSGWARRRRLGAMLSLMRDCWRTRAYGDFWSYMLVAEGAVDLAAEPELELYDMAALVPIVTQAGGRFTSLAGEAGPWGGDAVATNGLLHDVVLERLAAETD

>HisN_Actinomyces_cardiffensis |492780687|ref|WP_005961937.1| histidinol-phosphatase [Actinomyces cardiffensis]

MTTDARYNDDLRLAHVLADQADAITLARFGAQDLVVDSKPDLTPVTDADRAVEDMIRSQLAHTRSRDSVVGEERGTSGHASRQWIIDPIDGTKNFVRGVPVWATLIGLVEDGEVVMGVVSAPALKRRWWAAKGQGSFAGTSLFKGRKLSVSSVAELSDASLSYSSLDSWLHAERGGEFLRMQSQFWRTRAYGDFWSYMLVAEGAVDAACEPELELYDMAALVPIVTEAGGRFTSLRAEEGPWGGNALATNGLLHERLLRILDWPGFAASDEE

>HisN_Actinomyces_slackii |916673936|ref|WP_051281027.1| histidinol-phosphatase [Actinomyces slackii]

MRASLTPARYRGVMSDTAAPTPPVTERRWRDDLHLAHMIADQVDRLTQARFDARDFTVETKPDLTPVTEADREAERAIREHLGRARGRDSVLGEEMPTTGRSPRQWVIDPIDGTKNFVRGVPVWATLIALVEDGQVVVGLVSAPAMTRRWWAVKGGGAWTGRSLSSARRLSVSRVSHLADASLSYSSLSGWATAKRLRGMLGLMQACWRTRAYGDFWSYMLLAEGAVDLAAEPELELYDMAALVPIVTEAGGTFTSLSWQAGPFGGNAVASNTLLHGAVMEHLGTETD

>HisN_Actinomyces_israelii |759874510|ref|WP_043561361.1| histidinol-phosphatase [Actinomyces israelii]

MIETASPPPAGERRWRDDLHLAHTIADQVDPLTRAHFDNQDFEVETKPDLTPVTAADREAERLIRDYLSRARTRDSVLGEELGVTGRSPRQWVIDPIDGTKSFVRGVPVWATLISLVEDGEVVVGLASAPALDKRWWAVRGGGAWSGRSLALARRLHVSSVTALQDASMSYSSLSGWAERRRLRGMLSLMQSCWRTRAYGDFWSYMLLASGAVDLAAEPELEVYDMAALVPIVTEAGGRFTSLAGEPGPWGGDAVATNSLLHDAVLARLAEETD

>HisN_Actinomyces_sp._oral_taxon_848 |496491505|ref|WP_009200075.1| histidinol-phosphatase [Actinomyces sp. oral taxon 848]

MSLTRVNDDLAFASSIIDRVDSVTLRHFQTEDFSAQTKEDFSPATGVGREADRIIRAMLGRSRSRDAIYGEEHGGILLRSARRWIIDPIDGTKNFVRGVPVWGTLLALEEEGEIVVGIVSAPSLGRRWWAAKGVGAFTGKSWVGARRLRVSEVSDLAEASMSYSSLKGWADRGQLRAFLRLAQRVWRSRAYGDFWSFMLVAEGAVDLACEPELELFDMAALVPIVTEAGGSFTSLHGEPGPWGGCALATNGALHPEVLEILGSIRDEDAVGEGAPNLSASAARSQSGHN

>HisN_Actinobaculum_sp._oral_taxon_183 |749925919|ref|WP_040317570.1| histidinol-phosphatase [Actinobaculum sp. oral taxon 183]

MASLTRLSDDLAFARSIIDRVDSLTLERFTAGDLAVETKADDTPVTDADREAERVIRAMLGRSRSRDAVYGEEEGGVTVRAARRWIIDPIDGTKNYMRGVPVWATLLALEEEGEIVLGIVSAPSLGRRWWGAKGLGAFTGRSWVGARRIRVSEVADLADASISYSSLSGWATRGQLRAFMRLTQQAWRTRAYGDFWSYMLVAEGAVDVAAEPELQLYDMAALVPIVEEAGGSFTSLHGEPGPWGGCALATNGLLHADVLEVLDSVRDG

>HisN_Alloscardovia_criceti |516878139|ref|WP_018143577.1| hypothetical protein [Alloscardovia criceti]

MNEKIFDSPFYEDLMHALEFADIADAISLERFGALDLKVETKSDNTPVSDADRAVERAIRKAIALYYPTDTVYGEELGKQESAGRRWILDPIDGTKNYVRGVPVWASLIALQVDDELVVSVVSAPALGMRWFAVQGAGAYMGKNFEDARRIHVSQVSQLHDASLSLSSLSGWKDRGNRDHLIALTDEVWRVRGFGDFWQYMLVAQGAVDAAAEPELDLYDMAALVPIVTEAGGRFTDLEGNPGPWGGCALGSNGLIHEDILERLR

>HisN_Bifidobacterium_longum |896174917|ref|WP_049187904.1| histidinol-phosphatase [Bifidobacterium longum]

MTEFYDDLIHALEYADVADEISMNRFGALDLHINTKPDNTPVTDADRAVERAIRNKIASHNPSDSIYGEELGKQESTGRRWIIDPIDGTKNFVRGVPVWATLIGLQVGEDLVASVVSAPALGMRWFAAQGAGAFMGTDMNNARQIHVSKVSRLEDASLSLSSLSGWKERGNRDKVIDLTDQVWRLRGYGDFWQYMLVAQGAVDIAAEPELDLYDMAALVPIITEAGGSFTDLNGNPGPWGGCGLATNGLLHAQVLEKLQ

>HisN_Bifidobacterium_crudilactis |917260615|ref|WP_051867327.1| histidinol-phosphatase [Bifidobacterium crudilactis]

MVAKTVYDEQSFTQELKGNPHYDDLLLALEMADAADQLTSHRFGAMDLRVQDKPDHTPVTDADRATEGLIRTMLGESRPDDSIYGEELGKAESTGRRWIIDPIDGTKNFVRGVPVWATLIGLQDGDDIVVGVVSSPMLHNRWFAVKDGGAYMGENPEHAVRLHVSGVADIADASMSLSSLTGWKERGDRDSLITLTDGMWRLRGFGDFWQYMLVAQGAVDVAAEPELDLYDMAALVPVVVEAGGRFTDLNGNPGPWGGNGLASNGLIHQTVLDALNS

>HisN_Bifidobacterium_coryneforme |799123427|ref|WP_045921320.1| histidinol-phosphatase [Bifidobacterium coryneforme]

MSIEAEEWSLQDDLDLAIAMAGQADLVTTDYFKSPHLHVERKADNTPVTQADREAEAVIRRILAEVRPDDTVYAEELGRQESNGRRWIIDPIDGTKNYVRGVPVWATLIGLEVDHQMVVGVVSAPMLGTIWYAARGMGAYMVRQGEAPQAIHVSKVDRMEDASMSISSLTGWKAIGRREQLIDLTDRIWRLRGFGDFWQYMLLAQGAIDLAAEPELDLYDMGALVPIVTEAGGTFTDLSGNPGPWGGNGLASNGLLHKEALSALGR

>HisN_Bifidobacterium_indicum |705388153|ref|WP_033490194.1| histidinol-phosphatase [Bifidobacterium indicum]

MSIEAEEWSLQDDLDLAIAMAGQADLVTTDYFKSPHLHVERKADNTPVTQADREAEAVIRRILAEVRPDDTVYAEELGRQESNGRRWIIDPIDGTKNYVRGVPVWATLIGLEVDHQMVVGVVSAPMLGTIWYAARGMGAYMVRQGEAPQAIHVSKVDHMEDASMSISSLTGWKAIGRREQLIDLTDRIWRLRGFGDFWQYMLLAQGAIDLAAEPELDLYDMGALVPIVTEAGGTFTDLSGNPGPWGGNGLASNGLLHKEALSALNR

>HisN_Mycobacterium_leprae |499210321|ref|WP_010907861.1| histidinol-phosphatase []

MLALTLADRADALTSARFGALNLRVDTKPDLTPVTDADRAVEADVRAVLGRERPKDGILGEEYGGTITFSGQQWIVDPIDGTKNFVRGVPVWASLIALLEDGVPSIGVVSAPALQRRWWAARGQGAFVAVDGVPRRLAVSEVADLNSASLSFSSLSGWAQRGLRDRFLELTDAVWRVRAYGDFLSYCLLAEGAIDVAAEPKVSVWDLAALDIVVREAGGVLTGLDGTPGPHGGSAVATNGRLHQEVLTRIGATVK

>HisN_Mycobacterium_iranicum |638978354|ref|WP_024444301.1| histidinol phosphatase []

MGTSVDDLDLALQLADEADALTMQRFGAVDLRVETKPDMTPATDADLDTEKLLRAGLARHRPDDSVFGEEFGGTKEFTGRQWVVDPIDGTKNFVRGVPVWSTLIALLADGVPVVGVVSAPALGRRWWAAEGQGAFTSFGGATRRISVSGVADLDSASLSFSDLTTGWDDLRARFVELLDSVWRVRGYGDFWSYCLVAEGAVDAAVEPEVKLWDLAPLDILVREAGGRFTDLAGQPGPHGGSALATNGLLHDTVLSALR

>HisN_Mycobacterium_marinum |501361788|ref|WP_012393354.1| histidinol-phosphatase []

MSEQDLHLALALADRADAVTRARFQALDLRVDTKPDLTPVTDADRAVETEIRAALQRERPGDSVLGEEYGGTTTLRGRQWIIDPIDGTKNFVRGVPVWASLIALLEDGVPSIGVVSAPALQRRWWAGRGQGAFTAVQGGPPRRIAVSSVAELNSASLSFSSLSGWAQLGLRGQFVDLTDAVWRVRAYGDFFSYCLLAEGAIDIAAEPEVSVWDLAPLDILVREAGGQFTSLDGTPGPHGGSAVASNGLLHEQVLNRLRSDRV

>HisN_Gordonia_amicalis |917388669|ref|WP_051995381.1| histidinol-phosphatase [Gordonia amicalis]

MSADSGPKNPRTAQSYDDDLELALSLADSADALTTDRFGAVDLRVDDKPDLTPVTDADLACETLLRERLAARRPADTVLGEEFGGDATLTGRQWVIDPIDGTKNFVRGVPVWATLIALLVDGVPTVGVVSAPALRRRWWAAGGLGAHTCFDGEDARQLSVSGVGDLSASSLAFSSLSGWADRGIRDRFVDLTDQVWRVRGYGDFFNYCLVAEGAVDVAAEPEVSLWDLAPLDILVREAGGRFTALDGTPGPAGGSAVASNGLLHDKVLAALRP

>HisN_Gordonia_aichiensis |491312873|ref|WP_005170851.1| histidinol phosphate phosphatase [Gordonia aichiensis]

MFPTPGSDLSADLDFALSLAESADALTMQRFGAIDLRVDSKPDLTPVSDADLACEQMIRERLAAASPDDQVLGEEFGGDAVLTGRQWVIDPIDGTKNFVRGVPIWATLIALLIDGVPVVGVVSAPALRRRWWAAEGRGAHVSFDRGRARRIEVSSVADLASSSLTFSSLSGWADRGIREEFIGLTDRVWRVRGYGDFLNYCLVAEGAVDIAAEPEVSLWDLAPLDVLVREAGGRFTALDGSPGPAGGSAVATNGLLHDEVLAALTT

>HisN_Gordonia_polyisoprenivorans |519014738|ref|WP_020170613.1| histidinol-phosphatase [Gordonia polyisoprenivorans]

MTSHPIPDSDDSDLGIALSLAIAADDLTMARFGALDLQVSQKPDLTPVSDADLACEEMIRAHLSRRRPGDVVLGEEFGGEPTRSGRQWVIDPIDGTKNFVRGVPVWATLIALLVDGVPTVGVVSAPALRRRWWAAQGQGAFAEFDGRTRSLSVSGVADVASASLAFSSLSGWAQAGIRDKFIALTDEVWRVRGYGDFYNYCLVAEGAVDITAEPEVSLWDLAALDILVREAGGRFTSLDGTPGPRGGNAVASNGLLHDAVLTALAAD

>HisN_Gordonia_sputi |491345657|ref|WP_005203594.1| histidinol-phosphatase [Gordonia sputi]

MLPTPGSDATADLDFALSLAESADTLTMGRFGAIDLEVDAKPDLTPVSDADLACERMIRERLAAVFPDDEVLGEEFGGDAILTGRQWVVDPIDGTKNFVRGVPIWATLIALLVDGVPVVGVVSAPALSRRWWAAAGHGAFVAFDHGDARQITVSQVADLASSSLTFSSLSGWADRGVRDKFIDLTDRVWRVRGYGDFLNYCLVAEGAVDIAAEPEVSLWDLAPLDVLVREAGGRFTSLDGSPGPGGGSAVATNGLLHDEVLTALEG

>HisN_Gordonia_malaquae |495651212|ref|WP_008375791.1| histidinol phosphate phosphatase [Gordonia malaquae]

MATDSAGSEHASDLALALELATAADALTSTRFGALDLQVDSKPDLTPVSDADLACERLLRDRLQQARPDDAVLGEEFGGDQVLQGRQWVIDPIDGTKNFVRGVPVWSTLIALLVDGVPVVGVVSAPALNRRWWASAGAGAFTSHDGVERSISVSKVGDLASSSLAFSSLSGWKDLGIRDQFIDLTDSVWRVRGYGDFFNYCLVAEGSVDVTAEPEVSLWDLAPLDILVREAGGRFTALDGSAGPAGGSAVASNGLLHDEVLAALR

>HisN_Nocardia_higoensis |750516000|ref|WP_040797870.1| histidinol-phosphatase [Nocardia higoensis]

MATHSSDLALALRLADEADAITTARFGALDLKVDAKPDLTPVSDADLAVEESIRRLLSHARPDDAVLGEEFGGEADFSGRQWVIDPIDGTKNFVRGVPVWASLIALLSDGVPVVGVVSAPALARRWWAASGSGAWTSFHPGAPKPITVSRVGELASASLAFSSLSGWRDRGLREKFVDLTDEVWRVRGYGDFFNYCLLAEGAVDIATEPEVSLWDLAALDILVREAGGRFTSLAGVDGPHGGDAIATNGLLHDETLARLRQG

>HisN_Nocardia_carnea |702702398|ref|WP_033243177.1| histidinol-phosphatase [Nocardia carnea]

MAAHSSDLELALRLADEADTITRERFGALDLKVDAKPDLTPVSDADLAVEEAIRRMLRHGRPDDSVLGEEFGGSAEFTGRQWVIDPIDGTKNFVRGVPVWATLIALLEDGVPVVGVVSAPALARRWWAAVGQGAWSTFHPGAPRPITVSAVAELDSASLAFSSLSGWRDRGLREKFIDLSDEVWRVRGYGDFLSYCLVAEGAVDIATEPEVSLWDLAALDILVREAGGTFTALDGRPGPHGGDAVATNGRLHQPILDRFAE

>HisN_Nocardia_nova |644652031|ref|WP_025347984.1| histidinol-phosphatase [Nocardia nova]

MSEHAGDLELALRLADEADAITRERFGALDLKVDSKPDLTPVSDADLAVERALRATLIERRPHDAVLGEEFGGDAEFTGRQWVIDPIDGTKNFVRGVPVWASLIALLQDGVPVVGVVSAPALSRRWWAATGSGAWTAFEGGEPSAISVSAVAGLNSASLAISSLSGWRDLGLRDQLIDLTDQVWRTRGYGDFFGYCLLAEGAVDIATEPEVSLWDLAPLDVLIREAGGTFTALDGRPGPHHGSAVATNGLLHESVLSALRA

>HisN_Nocardia_otitidiscaviarum |659885122|ref|WP_029931830.1| histidinol-phosphatase [Nocardia otitidiscaviarum]

MTSYSSDLELALRMADAADGITRDRFGALDLKIDSKPDLTPVSDADLAVEREIRAILAAERPEDAVLGEEFGGEAEFGGRQWVVDPIDGTKNFVRGVPVWATLIALLEDGVPVVGVVSAPALARRWWAAREAGAWSRFESGEPKAISVSAVPQLGSASLAISSLSGWRDIGRRDQLITLTDEVWRVRGYGDFFGYALLAEGAVDIVTEPELSLWDMAALDILIREAGGRFTALDGTPGPHGGSAVATNGLLHDVVLAALAS

>HisN_Nocardia_niigatensis |750578951|ref|WP_040860820.1| histidinol-phosphatase [Nocardia niigatensis]

MTGYSADLELALKLADAADGITRERFGALDLKIDSKPDLTPVSDADLAVERTLREILAVERPDDLVLGEEFGGDVEFSGRQWVIDPIDGTKNFVRGVPVWASLISLLEDGVPVVGAVSAPALARRWWAAAGHGAWSSFEQGEPRAISVSAVAELGAASLAISSLSGWRAIGRRAKLIALTDEVWRVRGYGDFYGYALLAEGAVDIVTEPELSLWDMAALDILIREAGGTFTSLDGKPGPHGGSAVATNSHLQDQVLAALQA

>HisN_Nocardia_seriolae |696558884|ref|WP_033090946.1| histidinol-phosphatase [Nocardia seriolae]

MTGYSDDLELALQLADAADGITRERFGALDLKIDSKPDLTPVSDADLAVERKVREILGTERPGDLVLGEEFGGDVEFSGRQWVIDPIDGTKNFVRGVPVWASLISLLEDGVPVVGVVSAPALSRRWWAAAGAGAWSSFEGGAAKAISVSAVAELGASSLAISSLSGWRAIGRRAKLIALTDEVWRVRGYGDFFGYALLAEGAVDICTEPELSLWDMAALDILIREAGGTFTALDGRPGPHGGSAVATNGHLQDQVLAALQA

>HisN_Rhodococcus_rhodnii |498792047|ref|WP_010837288.1| inositol-phosphate phosphatase [Rhodococcus rhodnii]

MSTDTDADLALALRLADLADAVTLERFGALDLKVNAKPDLTPVSDADLATERTLREILGSERPGDAILGEEFGGAAAVQGRQWVIDPIDGTKNFVRGVPVWATLVALLDDGEPVVGVVSAPALSRRWWAARGAGAWTTFAQRSPRRISVSGVDALDSASLSFSSLTGWAELGIRDRFLALTDTVWRVRGLGDFFSYCLVAEGAVDIAAEPEVSLWDLAALDVLVREAGGTFTSLDGTPGPHGGSAVATNGALHAATLDALAAS

>HisN_Rhodococcus_pyridinivorans |568332908|ref|WP_024101461.1| histidinol-phosphatase [Rhodococcus pyridinivorans]

MTTDYTADLDLALRLADASDAITRDRFGALDLKVDDKPDLSPVSDADLAVERAIREMLGEQRPDDAVLGEEFGGDAVFRGRQWVVDPIDGTKNFVRGVPVWASLISLLEDGVPVVGVVSAPALNRRWWAAADGGAWVSHDGAEPRRISVSEVTDLASASLSFSSLSGWHERGIRDRFVALTDDVWRVRAYGDFFSYCLLAEGALEIALEPEVSLWDLAALDVLVREAGGRFTNLEGADGPHGGSAIATNGTLHDEVLGRLK

>HisN_Rhodococcus_fascians |739327400|ref|WP_037189834.1| histidinol-phosphatase [Rhodococcus fascians]

MTDYAADLSLALRLADEADAITRRRFLAMDLSVDSKPDLTPVSDADLAVETMIRAELGSERPADALLGEEYGGTATFSGRQWVVDPIDGTKNFVRGVPVWATLIALLDDGIPRVGVVSAPALNRRWWAALDLGAWTASDGGTPRRIEVSKVDSVASSSLSFSSLSGWAELGIRDRFLDLTDAVWRVRGYGDFFSYCLVAEGAVDIAAEPEVSLWDLAALDVLVREAGGDFTALDASAGPHGGSAVATNGLLQHQILALLSPSAKNLA

>HisN_Rhodococcus_ruber |697987827|emb|CDZ91809.1| Histidinol-phosphatase [Rhodococcus ruber]

MCGVTSPPLGAGWASTSVWGMSTDFSADLDLALRLADEADAITRTRFGALDLTVTDKPDLTPVSDADLGVERAVRGTLGAERPGDAILGEEFGGDAVFTGRQWVVDPIDGTKNFVRGVPVWASLIALLDDGVPVVGVVSAPALNRRWWAAAGAGSWLAYDGGQPRRISVSQVAELSAASLSFSSLSGWRDRGRRDEFVALTDEVWRVRAYGDFYSYCLLAEGAVDVALEPEVSLWDLAALDVLVREAGGRFTNLDGAAGPHGGSAVATNGALHDGVLTRLQHAG

>HisN_Rhodococcus_erythropolis |918364722|ref|WP_052416997.1| hypothetical protein [Rhodococcus erythropolis]

MLSTRAEIALAITREAGDLARHWFDKSTLRVDTKSDGSPVTQADQEIEQMIRDALSENFPDDGIMGEEFDDVVGSSGNRWIIDPIDGTKSFVHSVPLYANLLAYEEAGEITFGAINLPSAGVLVSAEKGGGCWRNDEPAAVSDRTDLDGAYLMATWLEDWKPDTIRDLNSRGVILRTWGDAYGYAMVACGHADAIVDYTTKAYDLAPMPVIIEEAGGRFSSLDGRRNFDCGNGIASNAHLHDSIRQLVN

>HisN_Tsukamurella_sp._1534 |518032485|ref|WP_019202693.1| histidinol-phosphatase [Tsukamurella sp. 1534]

MSSAPSNPSSDLVLALALADLADSITMARFGALDLRVDAKPDLTPVSDADLATETALRERLAADRPDDAVLGEEFGGDAVFSGRQWVIDPIDGTKNYVRSVPVWSTLIALLEDGVPVAGVVSAPALRRRWYASAGGGAFVVFDGGEPRRIRVSAVSEIADASISFSDLSYFADREARARFLALADDAWRLRGYGDFWSYCLVAEGAVDVAAEPEVSLWDLAAVEILVREAGGVFTGYDGAPGPHGGSAIAANPALHPYVLRRLNP

>HisN_Frankia_sp._Iso899 |737981413|ref|WP_035943459.1| histidinol-phosphatase [Frankia sp. Iso899]

MQSASGPLGDLAVALQLADLADGITTSRFRASDLLVTNKPDLTPVSDADKAVERAIRTHLELQRPGDAFFGEEYGSTGTGPRKWVVDPIDGTKNYVRGVPVWATLIALLLDDEPVLGVVSAPALSQRWWASLGGGAFTSAQGEEPRPIRVSQVTKIADASLAYASLDGWRALGKLNSMIGLTQQVWRSRGYGDFWSYMLLAEGAVDLAAEPELSLWDMAALAPIVVEAGGRFTGLNGVDGVHQGNAAASNGLLHEALLGVLGTNRRRPTSGGAA

>HisN_Frankia_sp._CN3 |737964136|ref|WP_035927320.1| histidinol-phosphatase [Frankia sp. CN3]

MSGSTTELASGGWPTPADDLALALSLADAADKITLARFQAVDLRVESKPDNTPVSDADTAVESMIRERLAAVRPADAVLGEEEGLTGDAQRRWILDPVDGTKNFVRGVPVWATLLGLEVDGEMVVGVASAPAMGRRWWAASGGGAFTRTATGDTRALRVSSVSRLEDAFLSFASLEGWTERGRLDELLHLAGQVWRTRAYGDFWSHMMVAEGAVDVAPEPVVSLWDLAALQVIVEEAGGRFTDLRGRRGAGHGTVLSTNGHLHEAALASFAAGGPAR

>HisN_Frankia_sp._BMG5.1 |827462937|ref|WP_047222702.1| histidinol-phosphatase [Frankia sp. BMG5.1]

MTNAAQTDALEAVGTDLPGSDRQPASSGARPTVDGYATTGHPIGDDLALALALADAADAITLARFQAVDLRVEAKPDATPVTDADTAVEAMIRARLAESRPGDAVLGEEQGLIGADTRRRWVVDPVDGTKNFVRGVPVWGTLLALEVDGEVVVGVASAPAMGRRWWAARGQGAHTRTAVGETRSLAVSAVADLADAYLSFASVESWSAAGRLAPFLGLVDAVWRTRAYGDFWSHMMVAEGAVDLACEPDVSLWDMAALQVIVEEAGGTFTDLTGRRGPGGGSILTTNGRLHTRALDILNQPAQPAVR

>HisN_Blastococcus_sp._URHD0036 |657621728|ref|WP_029432714.1| histidinol-phosphatase [Blastococcus sp. URHD0036]

MTSGRDHTGDMHLAHVLADQADSISMDRFKAQDLQVDTKPDLTPVTDADRAVEEQLRITLARARTRDAVLGEEMGTTGHGPRRWVLDPIDGTKNFVRGVPVWATLIALFDGDEPVVGLVSAPALNRRWWAAKGVGAWTGRRLESATRCRVSGVAELADASLSYSSLSGWEERGMLDGFLDLTRDVWRTRAYGDFWSYVLLAEGAVDVACEPEVSVWDLAALDVIVREAGGTFTDVSGTPGPTGGSALATNGLLHEAALARLKPPTSA

>HisN_Modestobacter_sp._Leaf380 |947625594|ref|WP_056288583.1| histidinol-phosphatase [Modestobacter sp. Leaf380]

MTGTRGFTEDMQLAHVLADQADAISLDRFKAQDLVVDTKPDLTPVTDADRAVEQQLRSSLARTRTRDAVQGEEFGTTGHGARRWVVDPIDGTKNFVRGVPVWATLIALLDGDVPVVGMVSAPALGRRWWAAAGVGAYTGRRLESATRCHVSGVDTLADASLSYSSLSGWEERDRLDGFLDLTRSVWRTRAYGDFWSYTMLAEGAVDIACEPEVSLWDIAALDVIVREAGGTFTDLSGTPGPAGGNAVATNGHLHADVLAALAPR

>HisN_Nakamurella_multipartita |752721476|ref|WP_041368599.1| histidinol-phosphatase [Nakamurella multipartita]

MDRDLALALDLADLADEISLPRFGAADLQVTAKPDLTPVSDADLAVETALRERIARERPDDVVVGEEFGGADRPATGRRWIIDPIDGTKNFVRGVPVWATLIALIDPAVHADRPAVGVVSAPALARRWWAAAGGGAHARFAGGPARRCRVSGVSRLADASLSYSEPGEWQAAGRLRPFQTLVQRCWRTRAYGDFWSYLLVAEGAVDIAAEPDLSLWDVAALIPIVVEAGGRFTAIDGQPSGGSGGSALATNGLLHPEVISLLTG

>HisN_Actinomadura_oligospora |651281835|ref|WP_026413814.1| histidinol-phosphatase [Actinomadura oligospora]

MASYSDDLRLAHVLADAADDITTKRFRALDLSIETKPDLTPVSDADRSVEEQIRSTLSRARPRDAVLGEEYGRTSGSGSGKRCWVVDPIDATKNFVRGVPVWATLIALMEHDEVVVGVVSAPSLNRRWWAMRDGGAWTGRALNRAQRIQVSQVADLSDASLSFSSLSGWEEQGRLDRFLDLTRSVWRTRAFGDFWSHMMVAEGAVDISAEPEVSLWDLAALQVIVEEAGGMFTDLSGVPGPDGGSVVCTNGQLHAEVLKALGGGRLTLRV

>HisN_Actinomadura_rifamycini |651268042|ref|WP_026401236.1| histidinol-phosphatase [Actinomadura rifamycini]

MAGYSDDLRFAHVLADQADDITTKRFRALDLDVETKPDLTPVSDADRGVEEQLRATLGRSRPRDAILGEEYGRTGGGNRCWVIDPIDATKNFVRGVPVWATLIALMENDEVVVGVVSAPALNRRWWAARGGGAFTGRSLTRATPISVSAVGELSDASLSFSSLSGWEEAGRLNHFLDLTREVWRTRAFGDFWSHMMVAEGAVDVSTEPEVSLWDLAALQVIVEEAGGMFTDLSGVPGPDGGSVVCTNGKLHADVLRTLGGGQLSLRI

>HisN_Actinomadura_atramentaria |518463049|ref|WP_019633256.1| histidinol-phosphatase [Actinomadura atramentaria]

MAGYSDDLRLAHVLADGADDITTKRFRALDLAIETKPDLTPVSDADRSVEERIRGTLSRARPRDAVLGEEFGRTGYGRRCWIIDPIDATKNFVRGVPVWATLIALMDGEEVVVGVVSAPALNKRWWAARGGGAWTGRSLTSATRMQVSSVGELKDASLSFSSLSGWEERGGLGNFLELTRSVWRTRAFGDFWSHMMVAEGTVDVSCEPEVSLWDLAALQVIVEEAGGTFTDLSGEPGPDGGSVVCSNGRLHADVLRALGGGTVPGVGLRSV

>HisN_Microbispora_sp._GMKU363 |943651577|ref|WP_055479360.1| histidinol-phosphatase [Microbispora sp. GMKU363]

MGYSDDLHLAHLMADAADDITMRRFKAVDLKVDTKPDLTPVSDADRAVEEAIRGTLRRARPRDAVVGEEYGSTGDSTRSWIVDPIDGTKNYVRGVPVWATLIALMERGEIVVGVVSAPALHRRWWAAKGGGSWTGRNLTKASRCQVSSVGRVDDASLSFSDLEEWEKKGRLDDFLDLTREVWRTRAYGDFWSHVMVAEGAVDVSAEPELSLWDMAALTIIIEEAGGTCTTLDGTSPLDSASSLQEVSLVCTNGLLHGEVLKRLSRSAP

>HisN_Nocardiopsis_alba |516102914|ref|WP_017533494.1| histidinol-phosphatase [Nocardiopsis alba]

MGSFDDDLRLAHVLADAADDIALKRFRSLDLVVDTKPDLTPVTEADRMVEETLRGVLSRARPRDAVVGEEYGKSGNSNRVWVIDPIDGTKNYVRGVPVWATLIALLEGDRPVVGLVSAPALHRRWWASQGGGTWAGRNLSKATRCQVSQVTELSDASLSFSSLTGWEEQGRLESFLGLTRSVWRTRAYGDFWSHVMVAEGVVDIAAEPELSLWDAAPLPIILEEAGGRASALRGQGFEDGGPLVCTNGALHEQTLTWLNGGPTPLRRP

>HisN_Nocardiopsis_gilva |516213313|ref|WP_017617276.1| histidinol-phosphatase [Nocardiopsis gilva]

MASFDDDLRLAHVLADAADDISVKHFRALDLKVDTKPDLTPVTEADRAVEESLRNVLSRARPRDAVIGEEFGKTGNSHRVWCIDPIDGTKNYVRGVPVWATMIALLEGDRPVVGLVSAPALNRRWWASKDHGTWMGRSLTKATRCLVSQVSELEDASLSFSELSEWEQQNRLDSFLGLQRSVWRARAYGDFWSHVMVAEGAVDISAEPELSLWDAAPLPVILEEAGGRATDLRGQSFTDGGALVCTNGALHDQVLTWLNGGPTPLRAT

>HisN_Nocardiopsis_lucentensis |516178428|ref|WP_017596874.1| histidinol-phosphatase [Nocardiopsis lucentensis]

MASFDDDLRLAHVLADAADDLALKRFRALDLVVDTKPDLTPVTESDRLVEETLRGVLSRARPRDAVVGEEYGKTGNSNRVWVIDPIDGTKNYVRGVPVWATLVALLEGDRPVVGLVSAPALHRRWWASKGGGTWTGRGLAKATRCHVSKVSELADASFSYSSLSGWEERGLLDSFLGLTRSVWRTRAYGDFWSHVMVAEGVVDVAAEPELSLWDAAPLPIILEEAGGRATNLRGEGFEDGGPLVCSNGLLHDSALTWLNGGPTPVR

>HisN_Nocardiopsis_kunsanensis |648424306|ref|WP_026116057.1| histidinol-phosphatase [Nocardiopsis kunsanensis]

MNGSYKADLRLALGAAEAAAAYAVEARSGAEYRVDAKADGSPVSTADLRAEEAVRQLIVTERPDDHFLGEESPRSFSASASRVWVVDPIDATRNFVRGIPIWATLIALLDQGHPVVGVVYAPDLGRCWWAAAGEGAFCGESPSDQGRKIHTRDTEQPQDAYISTTAFDTWNEYGLMDEYQYLASRSYCNRGFGDFFQHCLVAEGVLDAALEPVVAPWDVAALVPVVSEAGGKCTDLHGGELPVAGGRGIVSSTPALHQQIMEVFTR

>HisN_Streptosporangium_amethystogenes |917146813|ref|WP_051753525.1| histidinol-phosphatase [Streptosporangium amethystogenes]

MADAADDLTMRRFKALDLRVENKPDLTPVSDADQATEQAIRATLGRARPRDTIIGEEFGKSGYGERSWIIDPIDGTKNYVRGVPVWATLIALVDQGRVVVGLVSAPALGRRWWAARDGGAWTGKSLTKATRCQVSSVAYLEDASFSYSSLGGWEEAGKLDAFLDLNRSVWRSRAYGDFWSHMMVAEGAVDVSAEPELSPWDIAALTVIIEEAGGIWTDLSGVPDIDGGSLLCSNGPLHTKVLERLGSGPLTFPV

>HisN_Streptomyces_rubellomurinus |783216258|ref|WP_045703724.1| histidinol-phosphatase [Streptomyces rubellomurinus]

MADYHDDLRLAHVLADSADSVTLERFRALDLKVETKPDLTPVSDADKAAEELVRGVLQRARPRDAVLGEEFGLQGSGPRRWVIDPIDGTKNYIRGVPVWATLIALIEDGPDGVERPVVGIVSAPALQRRWWAAEGLGAFAGRSLAKASRIHVSGVSRIEDASLSYSSLSGWEERGRLDPFLDLTRACWRTRAYGDFWSYMMLAEGAVDIAAEPELSLWDMAAPCIVVQEAGGRFTGLDGIDGPGGADAVATNGLLHEETLRRLSV

>HisN_Streptomyces_hygroscopicus |664293515|ref|WP_030822984.1| histidinol-phosphatase [Streptomyces hygroscopicus]

MPDYHDDLRLAHVLADAADSTTMERFKALDLKVETKPDMTPVSEADKAAEELIRGSLQRTRPRDAVLGEEFGSEGSGPRRWVVDPIDGTKNYVRGVPVWATLIALMERGEGGDRPVVGVVSAPALGRRWWAAEGLGAYTGRSLSSATRLAVSRVSRLSDASFAYSSLSGWEERGKLNGFLDLTRACWRTRGYGDFWPYMMVAEGSVDLCAEPELSLWDMAANAVIVQEAGGRFTGLDGVPGPNSGDAAASNGLLHGAMLDYLRS

>HisN_Streptomyces_graminilatus |943887777|ref|WP_055526048.1| histidinol-phosphatase [Streptomyces graminilatus]

MPDYLDDLRLAHVLADAADATTMDRFKALDLKVETKPDMTPVSEADKAAEELIRGQLQRARPRDAILGEEYGIEGTGPRRWVIDPIDGTKNYVRGVPVWATLISLMEAGEGGFQPVVGLVSAPALGRRWWAAKGHGAFTGRSLSSSSRLHVSNVSQMSDASFAYSSLSGWEDRGVLGGFLDLTREVWRTRAYGDFWPYMMVAEGAIDICAEPELSLWDMAANAIIVTEAGGTFTGLDGRPGPHSGDAAASNGHLHDEMLGYLNQRY

>HisN_Streptomyces_natalensis |662112364|ref|WP_030067780.1| histidinol-phosphatase [Streptomyces natalensis]

MPDYHDDLRLGHVLADAADAATMERFKALDLKVETKPDMTPVSEADKSAEELIRGQLQRARPRDAVLGEEFGSEGAGPRRWIIDPIDGTKNYVRGVPVWATLIALMERGEGGDRPVVGIVSAPALNRRWWAAKDLGAFTGRSLTSASRLAVSRVGRIQDASFAYSSLTGWEERGKLPGFLDLSRDCWRTRAYGDFWPYMMVAEGSVDICAEPELSLWDMAACAVVVEEAGGRFTGLDGRTGPHSGNAAASNGLLHEDLLTYIGD

>HisN_Streptomyces_xanthophaeus |665570727|ref|WP_031152345.1| histidinol-phosphatase [Streptomyces xanthophaeus]

MPAYDDDLRLALELADAADAVTMQRFRALDLVVETKPDMTPVSEADKAAEEAVRAGIEAARPGDAILGEEYGLKGSGPRRWVVDPIDGTKNYVRGVPVWATLISLMAEGPDGVFEPVVGVVSAPALGRRWWATLGGGAYAGGALGATTEIGVSKVAGLGDASFAYSSLGGWEEQGRLAGFLDLTRACWRTRGYGDFWPYMMVAEGSLDLCAEPELNLWDMAAIAVVVQEAGGRFTSLDGVDGVHGGNAAASNGLLHEEMLEHLRPRA

>HisN_Streptomyces_alboniger |1045231|emb|CAA63164.1| Mono-phosphatase [Streptomyces alboniger]

MPQQADLELAMRLADTADRITTRRFQARDCGYARKPDRTPVTDADTTVEAAVRESVRAARPDDDFAGEETGGEVTAGRTWIVDPIDGTKNFLRGVPVWATLIALLEDGRPTVGVVAAPALRSRWWAAAGHGAWLRRGSAGAEPLRLHVSGVARLENAYLSTTNTRTWDAFHSRAAYLRLADACWEDRAFGDFLQHCMVAEGTVDIAAEPVVSPWDIAALQILVEEAGGVCTDLLGGSPQRGTGALSANPELHRLAVEALAAPAAATTGAATIP

>HisN_Streptomyces_ipomoeae |889488406|ref|WP_048819990.1| histidinol-phosphatase [Streptomyces ipomoeae]

MRMNNDDIELMHLMADRADEITMERFRAADLRTLTKSDMTPVSEADHRVEEELRQLLSRRRPDDAVLGEEFGGRTSGSRSWLIDPIDGTAGYVRGVPVWASLLAVLEGDRVTAGLVSAPALGRRWWASRGNGAWAGAGPDTAQRCHVSGTADLTDASISYSEISEWERGGLLSAFLELNRRCGRSRAYGDFWSHMLVAEGTLDLSVELELEVWDLAPLLIILEEAGAAASGVSGLTGVGQDSLVCANPTLHRQALSILTNNS

>HisN_Streptomyces_varsoviensis |918564620|gb|KOG91654.1| histidinol phosphatase [Streptomyces varsoviensis]

GARALRVREKLDRTPVTDADTAVETAVREALRSDRPEDAFAGEETGGSVTAGRTWMVDPIDGTKNFLRGVPVWATLIALLEDGRPTVGVISAPALRSRWWAAAGQGAWLRRGAADSAAVPLRVSAHTRLDHAYLSTTSTRTWDAFHSRAAYLRLAEECWEDRAFGDFLQHCMVAEGTLDIAAEPVVNPWDITAVQILVTEAGGVCTDLLGASPRGGTGALSANPRLHQLALTVLAAPRP

>HisN_Kitasatospora_mediocidica |737830858|ref|WP_035798158.1| histidinol-phosphatase [Kitasatospora mediocidica]

MADYHDDLRLAHVLADSADSITLERFRALDLKIETKPDLTPVSDADKAAEELVRSVLQRARPRDAVLGEEFGLVGTGPRRWVIDPIDGTKNYIRGVPVWATLIALLEVGADGEEVPVVGIVSAPALQRRWWAAKDLGAYAGRSLAKASRIHVSQVSRIEDASLSFSSLTGWEERGRLDPFLDLTRACWRTRGFGDFWSYMMLAEGSVDIAAEPELSLWDMAAPCIVVQEAGGRFTGLDGVDGPGGADAVASNGLLHEETLRRLSV

>HisN_Kitasatospora_azatica |737879064|ref|WP_035845599.1| histidinol-phosphatase [Kitasatospora azatica]

MADYHDDLRLAHVLADSADSVTLERFRALDLKVETKPDLTPVSDADKAAEELVRGVLQRARPRDAVMGEEFGLVGSGPRRWVIDPIDGTKNYIRGVPVWATLIALLEVGADGQEQPVVGMVSAPALNRRWWAAKGLGAYAGRSLAKASRIQVSAVSRIEDASLSYSSLSGWEERGRLDPFLDLTRACWRTRAYGDFWSYMMVAEGAVDIAAEPELSLWDMAAPCIVVQEAGGRFTGLDGIDGPGGADAVASNGILHQEALRHLSV

>HisN_Kitasatospora_sp._MBT66 |759762440|ref|WP_043472520.1| histidinol-phosphatase [Kitasatospora sp. MBT66]

MTPPVPPEDDLELALRLGDLADGLTVRRFHALDLRVREKPDRTPVTDADTAVETAVREELRSARPDDAFAGEETGGSVTSGRTWLVDPIDGTKNFLRGVPVWATLIALLVDDRPTVGVISAPALHSRWWAAADRGAWLRRGPAGTTPVRLGVSGRTRLDQAYLSTTSTRTWDVFHSRAAYLRLAEACWEDRAFGDFLQHCMVAEGTLDIAAEPVVNPWDIAAVQVLVEEAGGRCTDLRGASPRNGTGALSANPRLHRLAVEALSAPPAAPGPTGP

>HisN_Streptacidiphilus_neutrinimicus |755011784|ref|WP_042365112.1| histidinol-phosphatase [Streptacidiphilus neutrinimicus]

MADYSDDLRLAHVLADHADSVTLDRFRALDLAVETKPDLTPVSDADKAVEDLLRSDLARARPRDTVLGEEFGTTTGQSPRRWVIDPIDGTKNFIRGVPVWATLISLMEAGPGGEQPVVGLVSAPALGRRWWAARGTGAFTGRSLTRASRIRVSEVSRLEDASFSYSSLTGWEERGKLDAFLDLTRACWRTRAYGDFWSYMMVAEGAVDIAAEPELSLWDMAANCVIVEEAGGRFTGLDGIPGVRSGNALVSNGLLHGEALHRLG

>HisN_Streptacidiphilus_oryzae |739719373|ref|WP_037573048.1| histidinol-phosphatase [Streptacidiphilus oryzae]

MPDFHDDLRLAHVLADHADSVTTERFKALDLRVETKPDLTPVSDADKAAEDVVRSVLSRARPRDAVMGEEHGVTGHGPRRWVIDPIDGTKNYVRGVPVWATLISLLELGPGGEEPVVGLVSAPALGKRWWSAKGSGAFTGRSLARATRLQVSDVSRLSDASLSYSSLTGWEERGLLPNFVDLSRKVWRTRAFGDFWSYMMVAEGAVDLAAEPELSLWDMAAPCAIVTEAGGRFTDLQGRPGVHGPNAVASNGLLHEALLGSLNA

>HisN_Rubrobacter_aplysinae |837740934|ref|WP_047866468.1| histidinol phosphate phosphatase [Rubrobacter aplysinae]

MSATQGLRHHLDFATTLAYEAGRMTLGYFRNGVRTETKEDESPVTVADREAERMIRARISERYPSHAILGEEYGPEAGAGSYRWIVDPIDGTLAFVRGVPLYAVLIALEIEGVCEVGAAYFPALDEMVHAATGEGCYCNGRRARVSQVQSLSEGIVAFTDAKGFEAYGREPEWDRLRRAARYARGWSDAYGHALVATGRAEVMLDPAMNPWDCAPFAPILREAGGYLGDWSGRETIYGGEALSTSRVLLPEVLDLVRRE

>HisN_Amycolatopsis_thermoflava |739462714|ref|WP_037322661.1| histidinol-phosphatase [Amycolatopsis thermoflava]

MASYTDDLILAGKLADAADSITTARFRARDLKVSSKPDRTPVTDADTAVEDAVREVLAAERPDDAVAGEERGGTAWESGRAWVLDPIDGTKNFLRGGPVWATLIALVEDGQPVVGMVSAPLLGRRWWAAAGEGAWMSDPAGERRISVSAVGALSDAYLSTTDLGSWTEYHSREAYLGLVDACWETRAFGDFWHHCLVAEGAIDLAAEPIVNPWDVAPMQILLTEAGGRFSDLSGAERFDGGSALSSNGHLHDEALGYLRR

>HisN_Amycolatopsis_benzoatilytica |654457645|ref|WP_027928056.1| histidinol-phosphatase [Amycolatopsis benzoatilytica]

MTVPAYSDDLSLATRLADAADALTTVRFRALDLAVSAKPDRTPVTDADTAVEDAIREILSAERPADAVAGEERGGTAGGEGRAWVIDPIDGTKNFLRGVPVWATLIALVEDGTPVVGMISAPLLGRRWWAARGEGAHLRDSAGQRQLSVSKVAALSDAYLSTTDLNSWTEYHSREKYLALTEACWESRAFGDFWSHCLVAEGAMDVAAEPVVNPWDVAAAQVIVTEAGGRFTDLSGAGTYEGGSALSTNGLLHDAALDLLRR

>HisN_Amycolatopsis_azurea |491297475|ref|WP_005155485.1| histidinol-phosphatase [Amycolatopsis azurea]

MTVPGYENDLELATRLADAADAITTARFRALDLAVERKPDRTPVTDADTAVEDAIRAILATDRPDDAVLGEERGGSAATGRAWVLDPIDGTKNFLRGVPVWATLIALVEDGDPVVGMISAPMLGRRWWAAAGDGAWMSDSAGERRISVSKVASLEDAYLSTTDLNSWVEYHSREKYLDLVDACWESRAFGDFWHHCLVAEGALDVTAECIVNPWDVAAAQVLITEAGGRFSDLDGEARYDNGSALSTNGLLHDEALAILKR

>HisN_Amycolatopsis_vancoresmycina |489195919|ref|WP_003105229.1| myo-inositol-1(or 4)-monophosphatase [Amycolatopsis vancoresmycina]

MTVPGYGDDLALATRLADAADAITTARFRALDLAVERKPDRTPVTDADTAVEDAIRALLAAERPEDAVLGEERGGSAATGRAWVLDPIDGTKNFLRGVPVWATLIALVVDGDPVVGLISAPLLGRRWWAATGSGAFSSDSAGTRQLSVSKVSSLSDAYLSTTDLNSWTEYHSREKYLGLVDACWETRAFGDFWHHVLVAEGALDVAAECIVNPWDVAAAQVIVTEAGGRFSDLDGVARYDNGSALSTNGRVHEEALAILKR

>HisN_Saccharomonospora_saliphila |518646524|ref|WP_019816505.1| histidinol-phosphatase [Saccharomonospora saliphila]

MTVSRYSFDLDLTGTLADAADAITTSRFRALDLDVDSKPDRTPVTDADTAVEDAVRELLARHRPDDAIAGEERGGSVAAPGRVWVIDPIDGTKNFLRGTPVWATLLALVENGTPVAGMISAPLLGRRWWAAAGEGAWMRDTEGARPISVSRVAALSDATVSTTDLGSWVVHHDRGAYLDLVDATWESRAFGDFWQHCLVAEGALDIAVEPVVNPWDVAPVRVLVEQAGGRFTDLGGNPGIEGGSALSTNAQLHDAALELLRR

>HisN_Saccharomonospora_paurometabolica |494084390|ref|WP_007026428.1| histidinol-phosphatase [Saccharomonospora paurometabolica]

MTVSRYSPDLDLARTLADAADGITTARFRALDLTVDSKPDRTPVTDADTAVEDAVRALLTRHRPDDAVAGEERGGSATGPGRVWVIDPIDGTKNFLRGLPVWATLIALVEDGTPVVGMISAPLLGRRWWAGADAGAWVADDSGVRGIGVSRVTEPGDATVSTTDLGSWVEHHRRESYLRLVDATWESRAFGDFWHHCLVAEGALDIAVEPIVNTWDVAPVRVLVEQAGGRFTDLAGTPRIDGGSALSTNGLLHDTALTLLSDPDTAPGTH

>HisN_Saccharomonospora_halophila |518640607|ref|WP_019810715.1| histidinol-phosphatase [Saccharomonospora halophila]

MTVSRYSLDLDLARALADAADAITTARFRALDLTVDSKPDRTPVTDADTAVEDAVRTLLTRHRPDDAVAGEERGGSTTGPGRVWVIDPIDGTKNFLRGLPVWATLIALVEDGTPVVGMISAPLLGRRWWAAADAGAWLSDGSGVRGIGVSRVTDPADATVSTTDLGSWVEHHRRESYLRLVDATWESRAFGDFWHHCLVAEGALDIAVEPVVNTWDVAPVRVLVEQAGGRFTDLAGTPRIDGGSALSTNGLLHDTALALLSEPDTEPGTH

>HisN_Pseudonocardia_spinosispora |655589474|ref|WP_028936616.1| histidinol-phosphatase [Pseudonocardia spinosispora]

MTVTDDLTLALALADAADRITLDRFQASDLKIDRKPDRTPVTDADTATEDALRALIGEHRPDDAVLGEERGLDSRAGDRAWILDPIDGTKNYSRGVPVWATLIALTEAGRPTVGVVSAPALGRRWWASAGGGAFTDAGQGARRISVSGVADLSDACLSTTELSTWVELGALQQYLDLAARCWVVRAFGDFWQHMLVAEGAIDLGLDGEANAWDLAAIQLIVTEAGGRFSDLSGADRFDGGSGISSNGLLHDAALDTLRR

>HisN_Pseudonocardia_autotrophica |739182722|ref|WP_037046440.1| histidinol-phosphatase [Pseudonocardia autotrophica]

MTSTGTDSDTRADLALALQLADIADAITLPRFRATDLRVTRKPDRTPVTDADTAAEDALRAALGGQRPGDAVLGEERGGSLDALPPSGRGWVLDPIDGTKNFSRGMPVWATLIALTEHGRPTVGVVSAPALGRRWWGAAGHGAWTSDRPGGESRRISVTGVADLADCYVSTTDLNTFRAHGLLDGWLALTGACWETRAFGDFWQHCLVAEGVIDLAVEPEANAWDLAAVQPILEEAGGRLTDLQGRPGFAGGNGIASNGLLHDAAVTILGAG

>HisN_Pseudonocardia_sp._P1 |656306689|ref|WP_029239299.1| histidinol-phosphatase [Pseudonocardia sp. P1]

MPADPSDLDLALRLADLADAITLPRFRAADLRVTRKPDRTPVTDADTSAEDAIRAALGHERPRDAILGEERGGDVDGARAAGRGWVIDPIDGTKNFSRGVPAWATLIALVVDGVPVAGVVSAPALQRRWWGSAGAGAWARDLPSGTPRRIAVSGVADLADAYVSTTNQDTFRTEPGPVPRDGWTRLTEACWESRGFGDFWQHVLVAEGVLDVAVEPAANPWDLAAPAVVVAEAGGRLTDLTGEPTWSGGHGLTSNGLLHDTVVEVLRG

>HisN_Thermocrispum_agreste |655465930|ref|WP_028848876.1| histidinol-phosphatase [Thermocrispum agreste]

MTRPYADDIHLAKRLADTADAITVPRYSVRDLTVRTKPDRSPVTDADLAVEEAIRGVLATERPQDRIAGEEGGGEIGSGRTWVLDPIDGTKNFLRGVPVWATLIALVDDGVPVVGVISAPMLARRWWADAGGGAWVADASGERRLRVSQVSDLSDAYVSTTNLNTWDEYHSKQAYLDLVAACWENRAVGDFWSHCLVAEGVIDIAAEPIVNPWDVAAVQVLVTEAGGRFTDLTGAERFDGGNALSTNGVLHEAALRILSR

>HisN_Kribbella_flavida |502687260|ref|WP_012922796.1| histidinol-phosphatase [Kribbella flavida]

MPSHTDDLRLAHILADDADSTTMDRYKALDLHVATKPDLTPVSESDRKVEDVMRKTLARARPRDAFVGEEEGTTGWGVRRWVVDPIDGTKNYIRGVPVWATLISLMIEDQVVVGVVSAPALGRRWWASYGDGAWTGRALMSAQPCRVSDVSKIEDASMSYSSLQGWEKLGKRDQWADLMDSCWRTRAYGDFWSYMLVAEGAVDIAAEPELNLYDMAALAIIVDEAGGKFTSLDGTPGPNGPNAVATNGRLHEEVLSRLG

>HisN_Nocardioides_sp._Soil777 |948230964|ref|WP_056888636.1| histidinol-phosphatase [Nocardioides sp. Soil777]

MPISGPGHQDYTDDLRLAHVLADDADSLTQARFKALDLHVMSKPDLTPVTDADEAVEEGIRRTLSRVRSRDAVLGEEHGSSGHSQRQWIVDPIDGTKNFVRGVPVWATLIALAVDGEVVLGVVSAPQLQRRWWASKGHGAWTGRSLLKATQCQVSDVRRLEDASLSYSSLSGWDERDRLSDFVNLTRRCWRTRAYGDFWSYMLLAEGTVDIAAEPELALYDMAALDVIVREAGGRFTSLDGTDGPYGGNALATNGHLHEAALSFLGSLPDDLDDPDYRPTGHGSVHDLRARQTPPTVAD

>HisN_Nocardioides_insulae |738647110|ref|WP_036555968.1| histidinol-phosphatase [Nocardioides insulae]

MPTPDYTDDLRLAHLLADDADSLTTARFKALDLHVMTKPDLTPVSDADQAVEESIRRTLGRARSRDAVLGEEQGASGHSQRRWIIDPIDGTKNFVRGVPVWATLIALEVDNEIVLSVVSAPQLQRRWWAMTGQGAWTGKSLMKATRCQVSDIRRLEDASLSYSSLSGWEDRERLEDVLSLMRRVWRTRAYGDFWSYMLVAEGAVDIATEPELETYDMAALDIIVREAGGTFTSLDGSDGPWGGNALATNGHLHESVLAFLGTGRLDGEDPDLPRSGHGTVSTLRPRPES

>HisN_Nocardioides_luteus |780294086|ref|WP_045549341.1| histidinol-phosphatase [Nocardioides luteus]

MASTPDYNDDLRLAHLLADDADSLSTSRFKALDLHVMTKPDLTPVSDADKAVEESIRRTLSRARTRDAITGEESGSSGSSSRRWIVDPIDGTKNFVRGVPVWATLIALAVDNEVVMSVVSAPQLGRRWWAAKGQGAHTGKSLMKSTPCQVSDVRRLEDASMSYSSLHGWEERERLEDFLALMRSCWRTRAYSDFWSYMLVAEGAVDIAVEPELEVYDMAALDIIVREAGGSFTSLAGDPGPWGGNALATNGHLHDAVLSFLGGLPDGNNDADWPAAQPGSVTAFRRPASE

>HisN_Aeromicrobium_massiliense |517974963|ref|WP_019145171.1| hypothetical protein [Aeromicrobium massiliense]

MAHAYHDDLRLAHVLADTADNLSMDRFGAVDLQVSTKPDMTFVTESDQAVEEAIRRTLRSARTRDSVLGEEQGEQVGASEGRRWIIDPIDGTSNFVRGVPVWATLIALEEDGEIVAGCVSAPALGRRWWAAKGSGAYTGKSLMSSRRIQVSQVGDLDHASFSYSSLHGWEEIGRLEPFLALTRRCWRTRAYGDFWSYMMLAEGVVDVAAEPELKVWDMAALDVIVREAGGTFASLSGEPGPWGDNALATNTRLHDAAMAYLGYFPDAEPHDGDSNVTPFDWARERD

>HisN_Aeromicrobium_sp._Root236 |947736318|ref|WP_056398458.1| histidinol-phosphatase [Aeromicrobium sp. Root236]

MAHSYNDDLRLAHVLADNADNLSMDRFGAIDLQVDTKPDMTYVTESDEAVEAAIRRTLKSARTRDIVLGEENGEVEGTSGPGGRRWIIDPIDGTSNFVRGVPVWATLIALEEDGEIVAGCVSAPALGRRWWASKGTGAYTGKSLMASREIRVSQVSDLDSASVSYASLGGWDAIGKGDAFAALLRRCWRTRAYGDFWSYMLLAEGAVDIAAEPELKVWDMAALDIVVREAGGTFTSLAGQPGPWGDNALATNGRLHDSAMAYLGHFPDQGPPDQSWTDEPEPEPEPDNVRSFDFTREHVEDAP

>HisN_Aeromicrobium_sp._Root472D3 |947947105|ref|WP_056607079.1| histidinol-phosphatase [Aeromicrobium sp. Root472D3]

MAHSYSDDLRLAHVLADNADSLSMERFLAIDLQVDTKPDMTYVTESDQAVETAIRRTLKSARTRDVVLGEEQGEQDGAGAGGGGGGRRWIVDPIDGTSNFVRGVPVWATLIALEEDGEIVAGCVSAPALGRRWWASKGTGAHTGKSLLSSREIKVSQVADLGAASLSYASLGGWDAIGRGQAFAALMRRCWRTRAYGDFWSYMLLAEGAVDIAAEPELNLWDMAALDVIVREAGGTFTSLAGAPGPWGDNALATNSRLHDAAMAYVGHFPDDGPPDLSWTDEPEDDDAEPARDNVRAFEFTREPADEAP

>HisN_Propionibacterium_thoenii |653567826|ref|WP_027587094.1| histidinol-phosphatase [Propionibacterium thoenii]

MTDYTDDLRLAHVMADDADSITMARFQAADLHVSSKPDHTEVSDADLAVEDSVRRTLSKSRSRDAVHGEERPDSGHGPRRWIVDPIDGTANYVRGVPVWATLIGLAVEDHVVASVVSAPALRRRWWAAEGSGAWTGSSLLKARPIHVSGVSRLEDAFLSYSSLGGWVAAGRGHGFGELMRTVWRTRAFGDFWSYMLVAEGVVDLACEPDLNLHDMAALDVIVREAGGRFTSLDGADGPWGPNAMASNGLLHDRALEAVRPS

>HisN_Propionibacterium_acidifaciens |655297069|ref|WP_028706014.1| histidinol-phosphatase [Propionibacterium acidifaciens]

MAAAPDYTDDLRLAHLMADNADSLALNRFKARDLKVRAKADRTPVSEADQAIEASVRHTLATARPRDAVHGEEMPDTGYGPRQWIIDPIDGTANYVRGVPIWATLIGLRVGGEMVVGVVSAPALGRRWWAAEGSGAFTGTSLFTPNPIRVSGTSHLADAFLSYSSIHGWIDGGRGQGFVDLMRDCGRTRAFGDFFSYMLVAEGAVDLACEPDLELYDMAALVPIVTEAGGRFTNLDGAPGPVGRGALATNGILHDEVLERLAPDEGPDPDDGRS

>HisN_Propionibacterium_acnes |757594027|ref|WP_042842564.1| histidinol-phosphatase [Propionibacterium acnes]

MANHTDDLRLAHILADNADSLTMSRFGARDLTISTKPDHSEVTDADLAVEDSVRRTLSRMRSRDAVHGEERGDTGDGPRRWIIDPIDGTANFLRGVAVWATLIALSVEDEIVASVVSAPALKRRWWAARGLGAWTGKSLASATPIHVSNVRSLDDAFLSYSSLHGWVESGRGRGFGELMRSVWRTRAFGDFWSYMMVAEGVVDLACEPELGLHDMAALDIIVTEAGGKFTGLDGRDGPWSGNAMASNGFLHTEALAMVQPEP

>HisN_Propionibacterium_humerusii |488483035|ref|WP_002526705.1| histidinol-phosphatase [Propionibacterium humerusii]

MADHTDDLRLAHILADDADSLTMSRFGARSLTISTKPDNSEVTDADLAVEDSVRRTLSRIRSRDAVHGEERADTGDGPRRWIIDPIDGTANFLRGVPVWATLIALSVEDQIVTSVVSAPALKRRWWAARGLGAWSGKSLASATPIHVSNVRSLNDAFLSYSSLHGWVENRRGRGFGELMRSVWRSRAFGDFWSYMMVAEGVVDLACEPELGLHDMAALDVIVTEAGGKFTGLDGKDGPWSGNALASNGFLHDQALAMIQPQE

>HisN_Actinoplanes_sp._N902-109 |505438194|ref|WP_015625296.1| histidinol phosphate phosphatase [Actinoplanes sp. N902-109]

MSSYADDLALAHLLADTADSISMARFRALDLHVESKPDLTPVSDADTAVEQAIRSALARARPRDGVIGEEFGSSTAPAGQGNRHWVIDPIDGTKNFIRGVPVWATLIALMEGEHPVAGLVSAPALGRRWWGARGHGAYAGKHQRAATKLSVSAIREMGDASFCYSSLSGWEDAGRLEPMLGIMRDAWRSRAFGDFYGYMLLAEGALEVMVEPELSLWDVAALIPIVTEAGGRFTDVAGQRAPGDGSAIATNGHLHEEILERLSGGKNWSILAG

>HisN_Actinoplanes_globisporus |522002455|ref|WP_020513726.1| histidinol-phosphatase [Actinoplanes globisporus]

MAGYADDLALAHLLADTADSISMGRFRALDLRVESKPDLTPVSDADTAVEQAIRATLARARPRDGVLGEEFGHTEAAAGPGSRRWVIDPIDGTKNFVRGVPIWGTLIALLEGEVPVAGLVSAPALGRRWWAARGHGAFAGKHQHAATRIGVSGVGRLSDASFCFASLDGWASVGRLEAIVDLSLSAWRSRAYGDFYGYMLLAEGALDIMVEPELSLWDVAALIPIVTEAGGTITDQTGRPPGDKTAAIATNGLLHETILTALTSPKR

>HisN_Actinoplanes_missouriensis |504260310|ref|WP_014447412.1| histidinol-phosphatase [Actinoplanes missouriensis]

MAGYADDLALAHLLADTADAISTARFRALDLRVDAKPDLTPVSDADTAVENAIRSTLARARPRDGMLGEEFGRTVAAAGPGSRYWVIDPIDGTKNFVRGVPIWATLIALMEGDTPVVGLVSAPALGRRWWGARGLGAYAGKHQHAASRISVSSVRSLADASFCYASLNGWADNGRLDAMLDILLGVWRSRAYGDFYGYMLLAEGALEAMAEPELSLWDMAALIPIITEAGGKVTDLDGRAPADNSSVIATNGLLHETVLTALARRG

>HisN_Arthrobacter_alpinus |928487300|gb|ALE92639.1| histidinol phosphatase [Arthrobacter alpinus]

MTPPQTYNDDLRLAHVLADTVDSLTMSRFKALDLQVETKPDLTPVTDADKAAEESIRSQLSRVRPRDAVLGEEFGSSGHGSRRWIIDPIDGTKNFVRGVPVWATLIALVDEGRPVVGLVSAPALGKRWWAAEGTGAYMGKSLAAATRIRVSNVTELSDASLSYSSLTGWAERGNREEFLGLTDDVWRTRAYGDFWSYCMVAEGAVDIACEPELNLYDMAALVPIITEAGGRFTSLEGVDGPFGGNALATNSILHAEVLRRLNPGWDDLLA

>HisN_Arthrobacter_nitrophenolicus |496738334|ref|WP_009356872.1| histidinol phosphate phosphatase [Arthrobacter nitrophenolicus]

MIQPASSYNDDLRLAHVLADSVDDQTMSRFKALDLHVETKPDLTPVTDADKAAEEAIRGQLSRSRPRDAVLGEEFGSTGHGSRRWIIDPIDGTKNFVRGVPVWATLIALVDEGEPVVGVVSAPALGKRWWAAKGMGAYMGRSLAAATRLRVSNVSSLSDASLSYSSLSGWKERGNLDEFLGLTESVWRTRAYGDFWSYCMVAEGSVDIACEPELNLYDMAALVPIVVEAGGRFTSLEGEDGPFGGNALATNSILHSEVLQRLNPGLDDLL

>HisN_Arthrobacter_phenanthrenivorans |503366897|ref|WP_013601558.1| histidinol-phosphatase [Arthrobacter phenanthrenivorans]

MIQPASSYNDDLRLAHVLADSVDDQTMSRFKALDLHVETKPDLTPVTDADKAAEEAIRGQLSRSRPRDAVLGEEFGSTGHGSRRWIIDPIDGTKNFVRGVPVWATLIALVDEGEPVVGVVSAPALGKRWWAAKGMGAYMGRSLAAATRLRVSNVSSLSDASLSYSSLGGWKERGNLNEFLGLTEDVWRTRAFGDFWSYCMVAEGSVDIACEPELNLYDMAALVPIVVEAGGRFTSLEGEDGPFGGNALATNSILHSEVLRRLNPDLDDLL

>HisN_Arthrobacter_albus |917441843|ref|WP_052048261.1| histidinol-phosphatase [Arthrobacter albus]

MPDAATLADDLALAHELADAVDALTMERFGAADLYVETKPDATPVTEADRQAEQLIRERLSQARPGDAVFGEEFGSTGDSNRVWIIDPIDGTKNFMRGVPVWATLIALVEDGEVVVGLVSAPALGRRWWASVGGGAFTGASLDEARRIGVSGVTKLEDSSFSYSSLTGWKARGWLDGVLAISDTVWRTRAYGDFFSYCLVAEGAVDFAAEPELNLYDMAALVPIVREAGGVFTSLQGEDGPWGGNAIASNGLLHDAALNVLGTRGAERTPTPLPNHQHSAE

>HisN_Arthrobacter_arilaitensis |930825959|gb|ALG28660.1| histidinol phosphatase [Arthrobacter arilaitensis]

MADFTLQQDLDFALALADKVDQLTLARYGSNDLVVESKPDMTPVSDADRGAEQLILAELAKHRPEDSVLGEEFGVHGSGSRRWVIDPIDGTKNFVRRVPVWATLIALLVDDEPVLGVISAPALSRRWHAATGLGAYVSEPAAGAGERATRKISVSSVSELADSSLAYASLNGWKDAGKLDGFLDLLDAAWRTRGYGDFYSYALLAEGAVDAAFEPELELYDMAALVPVVREAGGRFTSVAGEEGCHGGNALATNGKLHAAILPYLNR

>HisN_Curtobacterium_luteum |970446008|ref|WP_058727077.1| histidinol phosphatase [Curtobacterium luteum]

MDLRDDLDFARSLADTADAISLERFRAADLHVSKKADSTHVTDADQAVERALRERLATERPDDAFLGEETTADTGADAVSEGHRQWVVDPIDGTANYLRGVPVWATLIALAVDGRPVLGVVSAPALGKRWWAAEGLGAHSFDGPLRVSGVDDLGDASLSYNSIQQWDEDDRLESLIELSRTVWRTRAYGDMWSYMMVAEGVLDVAGEPDLKPWDIAALVPIVEEAGGRFTSLDGDPGPWHGSALATNGLVHDAVVDLLRR

>HisN_Curtobacterium_flaccumfaciens |755625045|ref|WP_042539627.1| histidinol-phosphatase [Curtobacterium flaccumfaciens]

MGDVDLSADLDFARSLADTADTISLERFRAADLHVTKKADSTHVTDADQAVERALRERLAAERPDDAFLGEETTADTGAEAVSEGHRQWVVDPIDGTANYLRGVPVWATLIALAVDGRPVLGVVSAPALGKRWWAAEGSGAFSLDGPLRVSGVADLADASLSYNSIQQWDDDDRLEPLVDLSRRVWRTRAYGDMWSYMMVAEGVLDVAGEPDLKPWDIAALVPIVEEAGGRFTSLDGDPGPWHGSALATNGLVHDAVVEVIRRES

>HisN_Curtobacterium_sp._Leaf183 |947461593|ref|WP_056125964.1| histidinol-phosphatase [Curtobacterium sp. Leaf183]

MDLRADLDFARSLADTADAISLERFRAADLRVSRKADSTHVTDADQAVEGALRARITAERPDDAFLGEETTADSGAEAASEGHRQWVVDPIDGTANYLRGVPVWATLIALAVDGRPVLGVVSAPALGKRWWAAEGRGAHSLDGELHVSTVAELGEASLSYNSIQQWDDDDRLQPLIDLSRRVWRTRAYGDMWSYMMVAEGVLDVAGEPDLKPWDMAALVPIVEEAGGRFTSLDGDPGPWHGSALATNGLVHDAVVDVIRR

>HisN_Kocuria_kristinae |970450587|ref|WP_058731281.1| histidinol phosphatase [Kocuria kristinae]

MTATSPYNDDLRLAHVIADAVDRYTLARFQAQDFTVETKPDLTPVTEADREAEQLIRSHLARARTRDAVLGEEFGASGSSPRQWVIDPIDGTKNYVRGVPVWATLIALLDQGEPVVGLVSAPALQRRWWAASGSGAYTGRSLAKAQRIRVSEVTDLADASLSFSSLSGWRQLGLRERFLELTDAVWRVRGFGDFWSHCLVAEGSADLSAEPELNLHDMAALVPIVTEAGGRFTALDGTPGPHGGSALVSNGALHEAALDLLGTRAESRD

>HisN_Kocuria_palustris |923063484|ref|WP_053447134.1| histidinol-phosphatase [Kocuria palustris]

MRPHGSYTDDLRLAHVLADTVDSLTMARFRSQELQVETKPDLTPVTDADRDAEQLIRAQLARVRNRDSVLGEEFGTTGSGSRQWVVDPIDGTKNFVRGVPVWATLIAMIDDGVPVVGLVSAPALQRRWWAGAGTGAWAGRSLAKAERLSVSSVDRLEDASLSYSSLSGWKDRGRRDDFLELTDRVWRTRAYGDFWSYCLVAEGTVDLAAEPELNLYDMAALVPIVEEAGGRFTGLDGAPGPFSGNAVASNGLLHDQVLELIGD

>HisN_Actinotalea_fermentans |736116300|ref|WP_034248584.1| histidinol-phosphatase [Actinotalea fermentans]

MAYDDDLRLAHVIADQVDAVTMSRFRALDLHVETKPDHTPVSDADRLAEEVVRAQLGRTRPRDAVVGEELPATGHGPRQWVIDPIDGTKNFVRGVPVWATLIGLIDNGEVVLGLVSAPALGRRWWAAVGSGAWSGRSLSSATRLQVSQVGALHDASLSYSSLSGWEEQGRLDGFLDLTRAVWRTRAYGDFWSYVLVAEGAVDVACEPELALHDMAALVPIVTEAGGTFTSVRGVPGPFGGSALATNGLLHDQVRAFLDPFDED

>HisN_Micrococcus_sp._MS-ASIII-49 |765340853|ref|WP_044659235.1| histidinol-phosphatase [Micrococcus sp. MS-ASIII-49]

MERPENPATRGRDLTEDLRLAHMLADNVDSITMSRFKAQDLEVSTKPDLTPVTDADRAAEESIRSTLSRARARDGIVGEEFGGSLSRSGRQWVVDPIDGTKNFVRGVPVWATLIALLIDGEPVVGVVSAPALHRRWWAAAGQGAFAGTSLTRAQRITVSGVDRVEDASLSFSSIEGWRERGSIRAFLQLTSDVWRVRGFGDFWSYMLVAEGAVDIAAEPELELHDMAALVPIVREAGGRFTSLDGEDGPFGGHALATNGLLHDDVLARLRTGDETGDGA

>HisN_Janibacter_sp._Soil728 |947276465|ref|WP_055997766.1| histidinol-phosphatase [Janibacter sp. Soil728]

MATSQYADDLRLAHVLADQVERITMSRFQADDLVVESKPDLTPVSDADRSCEEAIRAQLSRSRGRDAVLGEEFGNTGNSQRRWIIDPIDGTKNYVRGVPVWATLIGLVDGEDCVMGLVAAPALGRRWWAAKDAGAWTGRSLAQAKPIGVSKVARLADASMSYSSLDGWRESGRGSNFLNLTSDMWRTRAYGDFWSYMLVAEGAVDVAAEPELELYDMAALVAIVEEAGGRFTSLDGKRGPWGGNAVASNGLLHDEVLSRLTP

>HisN_Janibacter_sp._HTCC2649 |84384074|gb|EAP99954.1| putative inositol monophosphatase [Janibacter sp. HTCC2649]

MTDVPTYDDDLRLAHVLADAVERVTVSRFRAEDLHVESKPDLTLVSDADRSAEELVRSQLKRTRPRDAVIGEEMENTGHGPRQWVIDPIDGTHNFVRGVPVWGTLIGLIDDGKPVLGLVAAPALNRRWWAALGSGAWVGRSLTSAKRMSVSKVSRISDASLSYSSASSWEAVGRQQAFLDLARDCWRERAYGDFWSYMLLAEGAVDIAAEPELNLHDMVALVPIVTEAGGRFTSLQGVDGPFGGNAVATNGLLHDEVLARIGL

>HisN_Intrasporangium_calvum |503258694|ref|WP_013493355.1| histidinol-phosphatase [Intrasporangium calvum]

MTSYDDDLRLAHVLADAVERVTMSRFRSADLLVESKPDLTPVTDADRAAEELIRLQLKRTRPRDAVEGEEFDTTGHGPRRWVVDPIDGTKNYVRGVPVWATLIGLVDNGRPVLGLVAAPALQRRWWAAVGSGAWSGRSLSSAKRISVSQVGRLGDASLSHSSLSGWRVRGRRDDFLDLMDDCWRTRAYGDFWSYMLVAEGAVDIAAEPELAVHDMAALVPIVEEAGGRFTGLDGREGCWSGNALATNGLLHDEVLRRIGTPATEA

>HisN_Brevibacterium_album |656050445|ref|WP_029088871.1| histidinol-phosphatase [Brevibacterium album]

MTDDLALALALADAADALSLPRFRAQDFSVEAKPDLTPVTEVDRAVEELIRARLADERPEDALLGEEFGAHGEASGRRWIVDPIDGTKNFVRGVPVWASLIALYEGTAPLVGVVSAPALGLRWWAATGSGAFRSVAGGAPEPLGVSGIADAADASLSYSSLGGWAERGLRERFLSLADAVWRTRAYGDFWSYMLVAEGVVDLACEPELELYDMAALVPVVREAGGRFTSLSGEDGPFGGDALASNGRLHDFALEAFGTAEAGA

>HisN_Brevibacterium_casei |496826172|ref|WP_009375253.1| histidinol phosphate phosphatase [Brevibacterium casei]

MNDLDLALDLADLADSLSLSRFIAQDFTIETKPDLTPVTECDRAVESALVDRLAVTRPEDSVLGEEFGSHGSSERRWIIDPIDGTKNYVRGVPVWATLISLYEGQRPLLGVVSAPALSRRWWAAAGQGAFARGPGGPDRRIEVSRVDDLGDASLSFASLGGWRDLGVRDEFIALTDAVWRTRGYGDFYSYMLVAEGAVDIACEPELALYDMGALAPIVLEAGGAFTNTAGVPGVFGGNAVATNGRLHERVLDALGRVAPEVAE

>HisN_Brevibacterium_mcbrellneri |492548695|ref|WP_005882100.1| histidinol-phosphatase [Brevibacterium mcbrellneri]

MKDLDLAIALADAADHLTLDSFYNRDFTVDTKPDMTPVTTVDRAVEEKLRELLALHAPDDAIVGEEFEPTTGTGTRQWIIDPIDGTKNFVRGVPVYATLISLYDGETPLLGVVSAPAMNRRWWASKGEGAWTTVAHDPEPVQIHVSQVSKLEDASFSYASLGGWKDLGKRDALLELCDTVWRTRAYGDFWSYMLVAEGAVDIAAEPELEIYDMGALVPVVTEAGGTFTSLNGKTGPFDGNALASNGLVHSSALELLS

>HisN_Cellulomonas_carbonis |759922304|ref|WP_043608103.1| histidinol-phosphatase [Cellulomonas carbonis]

MTVRSGYDDDLRLAHVIADQVDGLTTSRFRAQDLTIDTKPDTTPVTDADRSAEEIVRQQLSRTRPRDAVVGEEMPDTGHGPRQWVIDPIDGTKNFVRGVPVWATLIALIDAGRPVVGLVSAPALGRRWWAAENAGAWTGRSLASATRLQVSKVAQLEDASLSYSSLSGWEDRGLLGGFLDLTRAVWRTRAYGDFWSYVLVAEGAVDVAAEPELALHDMAALVPIVTEAGGTFTSVEGTPGPFGGSALATNGLLHDQVRAFLDPFGSG

>HisN_Cellulomonas_fimi |503537742|ref|WP_013771818.1| histidinol-phosphatase [Cellulomonas fimi]

MSLRPGYDDDLRLAHVMADQVDSLTMSRFKAQDLRVETKPDLTPVSDADRAAEELIRGQLSRARSRDAVQGEEMPDTGHGPRRWVVDPIDGTKNFVRGVPVWATLIALIDGDEVVVGLVSAPALGRRWWAAQGSGAWTGRSLASATRMQVSGVDRLEDASLSYSSLTGWEEHGGLDRFLDLTRRVWRTRAYGDFWSHVLVAEGAVDLSAEPELALHDMAALVPIVTEAGGRFTSVRGVPGPFGGSALVSNGLLHEQALELLDPMP

>HisN_Cellulomonas_massiliensis |517967563|ref|WP_019137771.1| histidinol-phosphatase [Cellulomonas massiliensis]

MSQRTGYDDDLRLAHVLADQVDALTMSRFKAQDLRVETKPDLTPVTDADRAAEELIRQGLSRSRPRDSVQGEELPTTGHGPRRWVVDPIDGTKNFVRGVPVWATLIALLDGDEVVVGLVSAPALGRRWWAAQGLGAWTGRSLAAATPMRVSQVTELADASLSYSSLGGWEEIGRLGSFFDLARSVWRTRAYGDFWSHVLVAEGAVDVSCEPELALHDMAALVPIVTEAGGRFTSVTGEPGPWGGSALVTNGHLHDAALALLEPRG

>HisN_Brachybacterium_squillarum |498220818|ref|WP_010534974.1| histidinol-phosphatase [Brachybacterium squillarum]

MSSRHADDLRLAHVLADAVDQLTMSRFKAQDLEVSTKPDLTEVTDADRAAEELVRAQLRRSRSRDQVIGEEFGSTGSAPRQWVIDPIDGTSNFVRGVPVWGTLIGLIEDGRPVVGLVSAPALSRRWWGGLEAGAWSGSRLSSASRLQVSQVESIDQASLSYSSLHGWAERDRLPQMLNLMQRFWRTRAYGDFWSYMLVAEGAVDVACEPELNLHDMVALAPIVTEAGGRFTSLDGQDGPFGGNVLATNGRLHEEVLEALELRG

>HisN_Brachybacterium_faecium |506256198|ref|WP_015775973.1| histidinol-phosphatase [Brachybacterium faecium]

MSSRFADDLRLAHVLADAVDQLTMSRFKAQDLEISTKPDLTEVTDADRAAEQLVRSQLSRSRSRDQVIGEEFGSTGASPRQWVIDPIDGTSNFVRGVPVWGTLIGLIEDGRPVVGLVSAPSLGRRWWGGEGVGAWTGSRINSASRLQVSTVDAVEEASLSYSSLHGWADRDRLPQMLNLMQRFWRTRAYGDFWSYMLVAEGAVDAACEPELALHDMVALVPIVTEAGGRFTSLEGEDGPFGGSAVATNGLLHEEILGALAPREA

>HisN_Brachybacterium_muris |516435007|ref|WP_017824032.1| histidinol-phosphatase [Brachybacterium muris]

MSSRFADDLRLAHVLADAVDQLTMSRFKAQDLEVTTKPDLTEVTDADRAAEQLVRSQLSRSRSRDQVIGEEFGSTGSSNRQWVIDPIDGTSNFVRGVPVWGTLIGLIEDGQPVVGLVSAPSLSRRWWGGAGAGAWTGSRISSASRLQVSQVDSIEDASLSYSSLHGWADAERLPQMLNLMQRFWRTRAYGDFWSYMLVAEGAVDVACEPELNLHDMVALAPIVTEAGGRFTSIDGEDGPFGGSAVATNGILHDEVLEAIAARDDRD

>HisN_Brachybacterium_phenoliresistens |740588872|ref|WP_038374432.1| histidinol-phosphatase [Brachybacterium phenoliresistens]

MSTRFADDLRLAHVLADAVDQLTMSRFKAQDLVVETKPDLTEVTDADRDAEQLVRSQLARSRSRDQVIGEEFGSTGTAARQWVVDPIDGTANFVRGVPVWATLIGLIEDGEVVMGLVSAPALSRRWWAAAGAGAWAGTRLSSASALRVSQVDELEDASLSYSALDGWAETGRLREMLNLMQRFWRTRAYGDFWSYMLLAEGAVDVATEPELKLHDMVALAPIVTEAGGRFTSLDGADGPFGGNALATNGHLHEQVLAALSAPAPGEAARS

>HisN_Phycicoccus_sp._Soil748 |948225782|ref|WP_056883467.1| histidinol-phosphatase [Phycicoccus sp. Soil748]

MPYDDDLRLAHVLADAVERITMARFRAEDLVVESKPDLTLVSDADKAAEELVRSQLKRTRPRDAVEGEEFETTGHGPRRWVIDPIDGTHNFVRGVPAWATLISLVDDGMPVLGLVAAPALGRRWWAANGSGAWSGRSLAAAKRIHVSKVSRVEDASLSYSSLAGWRRVDRHAQFLELTDDCWRTRAFGDFWSYMLVAEGAVDIAAEPELAVHDMAALVPIVTEAGGRFTSLAGQDGPWGGNALATNGILHDEVLERLGTDARA

>HisN_Sanguibacter_sp._Leaf3 |947472530|ref|WP_056136875.1| histidinol-phosphatase [Sanguibacter sp. Leaf3]

MTARSQYDDDLRLALVIADQVDAQTMSRFKALDLHVESKPDHTPVSDADKSAEEIIRGQLGRSRGRDAVLGEEFGATGHGARRWIIDPIDGTKNFVRGVPVWATLIALADGDDIVMGVVSAPALGRRWWAARGTGAWTGRSLSSATRLRVSGVSDIADASMSYSSLSGWEERGRLPHFLDLSRQVWRTRAYGDFWSYMLVAEGAVDIAVEPELELYDMAALVPIVTEAGGRFTSLAGVDGPFGGNAAVTNGHLHDHVLSAVGFQTH

>HisN_Cellulosimicrobium_funkei |827472839|ref|WP_047232144.1| histidinol-phosphatase [Cellulosimicrobium funkei]

MTPPSTRGYDDDLRLAHVIADQVDAHTMSRFKALDLHVESKPDSTPVSDADRTAEEIIRGQLGRARGRDAIVGEEFGETGHGARRWIVDPIDGTKNFVRGVPVWATLIALADGDEVVVGLVSAPALGRRWWAAKGTGAWTGKSLAAASRLQVSGVSAWSDASFAYASLDGWEERGKLEPFLDLLRGAWRTRGYGDFWSYMLVAEGAADAAAEPELELYDMAALVPIVTEAGGRFTSLDGADGPWGGNAVATNGLLHDEVLGRLG

>HisN_Promicromonospora_kroppenstedtii |739099756|ref|WP_036970448.1| histidinol-phosphatase [Promicromonospora kroppenstedtii]

MPVAAVTKDNYADDLRLAHVIADQVDSITMSRFRALDLKVETKPDLTPVSDADRAAEDFIRGQLARARTRDAIVGEEYGSAGSGARRWIVDPIDGTKNFVRGVPVWATLIALADGDEVVVGLVSAPALGRRWWAAKGSGAWTGKSLASASRMSVSGISRVADASFSYASLGGWEERGKLDGFLDLTRACARTRGYGDFWSYMLVADGAVDIAAEPELEVYDMAALVPIVTEAGGTFTSLDGAPGPWGGNAVATNGRLHEEALDFLG

>HisN_Knoellia_subterranea |917505282|ref|WP_052111699.1| histidinol-phosphatase [Knoellia subterranea]

MPTYDDDLRLAHVLADAVERVTVSRFRAEDLHVESKPDLTLVSDADRSAEELVRAQLKRTRPRDAVIGEEMETTGHGPRQWVIDPIDGTHNFVRGVPVWATLIGLIDDGKPVLGLVAAPALNRRWWAAVGSGAWAGRSLTSAKRISVSRVAKVSDASLSYSSARSWEAVGRQESLLDLARDCWRERAFGDFWSYMLLAEGAVDIATEPELNLHDMVALVPIVTEAGGRFTSLKGEDGPFGGNAVATNGLLHDEVLSRIGL

>HisN_Knoellia_aerolata |737973972|ref|WP_035936608.1| histidinol-phosphatase [Knoellia aerolata]

MPTYDDDLRLAHVLADAVERLTVSRFRAEDLVVESKPDLTLVSDADRSAEELVRSQLRRTRPRDAVIGEEMEPTGHGPRQWVIDPIDGTHNFVRGVPVWATLIGLIDDGKPVLGLVAAPALGRRWWAAVGSGAWAGRSLTSATRLSVSKVSQVSDASLSCSSMTSWDAVGRQEAVLDLARDCWRERAYGDFWSYMLLAEGAVDIAAEPELNLHDMVALVPIVTEAGGRFTSLRGEDGPFGGNALATNGLLHDEVLSRIGL

>HisN_Microbacterium_profundi |696573098|ref|WP_033104581.1| histidinol-phosphatase [Microbacterium profundi]

MTDSPAAADFTNDLALALRLADVADEQSLPRFDVSDLKVSTKADRSHVTDADLATERAIRALLAQERPDDGILGEEFGTEGDAHRQWIIDPIDGTANFMRGVPLWGTMISLAVDGVPQVGVVSMPALSRRWWASTGAGAWTATDAAPRRLETSAVSSPDDASVSFQSITQWADAGQLPALLRIADRVWRDRAYGDVYSYMLLAEGRLEMVAEFDVKEYDIAAAVPIVREAGGRFTSFDGVDTIAARSTLASNGLLHEAFLRLAHSPTNT

>HisN_Microbacterium_maritypicum |544773953|ref|WP_021198649.1| hypothetical protein [Microbacterium maritypicum]

MTTSPDSLRHDLDLALRLADAADAQSLPRFDASDLEISTKADNSHVTDADLATERAIRSILETDRPEDGIFGEEFGAQGSTQRQWIIDPIDGTANFLRGVPLWGTMIALAIDGVPQVGVVSMPALGRRWWASAGGGAWTTLEGEPRRLRTSSVSSLDDASVSFQSIAQWSEAGRLDTLLAVAGRVWRDRAYGDVYSYMLLAEGRIDMVAEFDVKEYDIAAAVPIVREAGGRMTDVDGVETISARSTLATNGTLHDDFLTLMQN

>HisN_Microbacterium_ginsengisoli |885216162|ref|WP_048808882.1| histidinol-phosphatase [Microbacterium ginsengisoli]

MTAAPVTPDAADARDAAASGLFAADLALALRLADAADAVTMARFDAADLEIDVKPDATHVTEADLATERAIRAILGSERPDDGIFGEEYGAEGSTHRQWIIDPIDGTANYLKGIPMWATLIALAVDGVPQVGVVSQPALGRRWWGATGSGAWTATDAGPRRLGVSSVDTLAEASISFQSIQQWDEVGRLDQLVALTRTVWRDRGYGDAWPYMLLAEGRLEFVAEFGVKEYDIAPMFPIIAEAGGRVTAIDGSASLADRSVLATNGALHDAFLGAVARAEGTAP

>HisN_Microbacterium mangrovi |746354727|ref|WP_039399397.1| histidinol-phosphatase [Microbacterium mangrovi]

MNSAASESAAEPSVAVDLAADLQLALRLADAADAVSMARFDAPDLDIRTKADSTHVTEADLATERAIRDILLTERPGDGVFGEEFGSTGSTARQWIIDPIDGTANYLKGIPMWATLIALAVDGVPVVGIVSQPSIGRRWWAARGHGAWTNVPGESAPRSIHVSAVDDVALSSVSFQSIQQWDEAGELDTLIRLSRGVWRDRGYGDAWPYMLLAEGRLEFVAEFGVKEYDIAAHVAIVREAGGRFTAFDGTDSIGDRSSLATNGVLHDAYLTLLHGEGAG

>HisN_Microbacterium_chocolatum |924869647|ref|WP_053548380.1| histidinol-phosphatase [Microbacterium chocolatum]

MTSAPPPSSFDAEFSGDLTADLAFALALADAADAVSMTRFDAPDLEVSTKADSSHVTEADLATERALRERIEAGRPGDGIFGEEYGTTGSSDRRWIIDPIDGTANYLKGIPMWATLIALAVDGVPRVGVVSQPALGRRWWGATGLGAWTRSADGRERALRVSSVDDIATSSVSFQSIGQWRDAGREAELDRLTRAVWRDRGYGDAWPFMLLAEGRLELVAEFGVKEYDIAAHVPIIREAGGRVTSFDGEDDLAARSVLATNGILHASFLDLLHSP

>HisN_Nesterenkonia_sp._NP1 |764922236|ref|WP_044493172.1| histidinol-phosphatase [Nesterenkonia sp. NP1]

MSLHSSPYTEDLRLAHMIADSVDSQTMQSFNAQDFEVETKPDLTPVTVADRQAEEYIRAQLGRARGRDAVYGEEFGSSGSGPRRWVIDPIDGTKNFIRGVPVWATLIALMDHGEPVVGLVSAPALGRRWWAAKDGGAYTGKSLSSAKQLRVSKVSRIEDANFSYSSLYGWREAGRSKNFLEFTDTVWRTRAFGDFWSYCLVAEGVVDVAAEPELNLHDMAALVPIVQEAGGRFTSLDGDPGCSGSNGLATNGLLHEAAMGALALDSATPIQD

>HisN_Nesterenkonia_jeotgali |970973036|gb|KUG59017.1| histidinol phosphatase [Nesterenkonia jeotgali]

MSLSTSAYTDDLRLAHMIADSVDAKTMSYFSSMDFEVETKPDLTPVTEADRQAEELIRGQLGRARGRDAILGEEFGSTGSGPRQWIIDPIDGTKNFIRGVPAWATLIALVDDGEPVIGLVSAPALGRRWWAAKDGGAYTGKSLASAKRLQVSKVSRLEDAFFSYSSLAGWRKLGRSQNFLELTETVWRTRAFGDFWSYCMVAEGQVDMAAEPELNLHDMAALVPIIREAGGIFTSLDGEPGCTGSNGLATNGLLHDAALGALAADRPAPF

>HisN_Dermacoccus_nishinomiyaensis |740782032|ref|WP_038567316.1| histidinol-phosphatase [Dermacoccus nishinomiyaensis]

MSDTLRDPSPYHDDLRLAHVLADSVEEITMSRFGAADLQVESKPDLTPVTEADREAERVIRENLKRARPRDAIKGEEFGTSGDADRLWIIDPIDGTKNYVRGVPVWATLIALVDAGTPVVGLVAAPALHRRWWAAQGTGAWTGRSLTSATSISVSKVDTLADASLSYSSFNGWAERGKDMDLFRLSKECWRSRAYGDFFSHMLVAQGSVDIAPEPELEDYDMAALVPIVTEAGGRFTGLDGRPGCWSGNGVSTNGLLHDEVLARVGDDSPAPSLIPGD

>HisN_Dermacoccus_sp._PE3 |829089542|ref|WP_047311030.1| histidinol-phosphatase [Dermacoccus sp. PE3]

MLREASPYHDDLRLAHVLADSVEEITMGRFGAPDLSIDTKPDLTPVTEADREAERVIRENLKRARPRDAIKGEEFGETGKGPRRWIIDPIDGTKNYVRGVPVWATLIALVDGDEPVVGLVAAPALHRRWWAAKDAGAWTGRNLTSATQIHVSKVDSLADASLSYSSFNGWAERGKDMDLFRLSKEVWRSRAYGDFFSHMLVAQGSVDIAPEPELEDYDMAALVPIVTEAGGRFSGLDGRDGCWSGNGVSTNGLLHAEVLARLGDNSPAPSLIPGE

>HisN_Tetrasphaera_jenkinsii |872696874|ref|WP_048548826.1| histidinol-phosphatase [Tetrasphaera jenkinsii]

MPGYDDDLRLAHVLADAVERVTMTRFKAEDLHVDTKPDATWVSDADRTAEQVIRAQLGRTRPRDAVHGEEMPDTGYGPRRWVIDPIDGTNNFVRGVPVWATLIALMDGPEAVMGLVAAPAIGRRWWAAKGTGAWTGRALTSAKPMKVSGVSRLADASISISDVVSWRATDRLDAMLGMMDACWRNRAYGDFWSYMLLAEGAVDIAAEPELALHDMAALVPIVTEAGGRFTSLDGRPGPFGGSGLATNGLLHDEVLHALTPELRAP

>HisN_Tetrasphaera_elongata |750472039|ref|WP_040753912.1| histidinol-phosphatase [Tetrasphaera elongata]

MLSRAFPIVSPVTTYDDDLRLAHVLADAVERVTMARFRSQDLAVETKPDLTLVSDADRAAEELIRSQLSRTRPRDAVVGEEFEPTGHGPRRWIVDPIDGTHNFVRGVPVWATLIALVVAEEPVMGLVAAPALGRRWWAAKGSGAYAGRSLTSATRIRTSGVQRLADASISLSSVTSWDAVGRTDAVLDLARDCWRQRAYGDFWSYMLLAEGSVDIASEPELALYDMAALAPIVTEAGGRFTSLDGTDGVFGGNALATNGHLHDHVLERIGL

>HisN_Jonesia_denitrificans |506252310|ref|WP_015772085.1| histidinol-phosphatase [Jonesia denitrificans]

MNASRYEDDLRLALVLADQVDNITMSRFKALDLHVEAKPDTTPVSDADRTAEEVIRAQLARSRSRDAIVGEEFGTTGHSSRRWIIDPIDGTKNFVRGVPVWATLIALAEGDDVVVGVVSAPALGRRWWAAQGSGAWSGRSLSSATRLHVSGVTSLADASLSYSSLTGWSQLGLQRRFLDLSDSVWRTRAYGDFWSYMLVAEGAVDAAMEPELELYDMAALVPIVTEAGGTFTSVDGTATGPWGPNAVASNGHLHGDILAALRAEE

>HisN_Jiangella_alkaliphila |820817086|ref|WP_046769731.1| histidinol-phosphatase [Jiangella alkaliphila]

MDPSDTPQRVGWPIVAHDDDIRFAHVLADDADSQTMSRYRAPDLRVETKADQSPVTDADKATEEAIRRTLGRARPRDAVVGEEFGAGGYGARRWVIDPIDGTKNYLRGVPVWATLIALMVEDQVVAGVVSAPALGRRWWAARGTGSYTGRSLSSATRCRVSDVSTLERASLSFSSLGGWEERGLLPNLLDLTRACWRTRAYGDFWSYMLLADGAVDIATEPQLALHDMAAPSIVVEEAGGRFTSLAGDPGPLGTDALATNGPLHDDVLARLGAAG

>HisN_Corynebacterium_callunae |505463714|ref|WP_015650606.1| hypothetical protein []

MSNYADDLALALELAELADSLTLERFEATDLEVSSKPDMTPVSDADLATEQALREKLSTARPADAILGEEFGGDVELSVRQWIIDPIDGTKNFVRGVPVWATLIALLDDGRPVAGVISAPALARRWWAAEGAGAWRTFNGTSPRKLQVSSVSKLEDASISFSSLSGWEERKLREQFIELSDDTWRLRGFGDFFSYCLVAEGAVDIAAEPEVSLWDLAPLSILVTEAGGRFTSLAGVDGPHGGDAIATNGLLHQEVLDRFKQS

>HisN_Corynebacterium_efficiens |493822005|ref|WP_006769508.1| histidinol-phosphatase []

MSNYADDLALALELAELADSITLDRFEATDLDVSSKPDMTPVSDADLAVETALREKLATARPSDAILGEEFGGETELTGRQWVIDPIDGTKNFVRGVPVWATLIALLDDGRPVAGVISAPALARRWWASEGSGAWRTFNGSSPRKLCVSSVTKLADASVSFSSLEGWKARDLRKNFIELSDDTWRLRGYGDFFSYCLVAEGAVDIAAEPEVSLWDLAPLSILVTEAGGRFSSLAGEDGPHGGDAVATNGALHDEVLKRLRG

>HisN_Corynebacterium_halotolerans |505213112|ref|WP_015400214.1| hypothetical protein []

MSTYADDLAFALELADLADSVTLERFEAVDLQVNSKPDMTPVSDADLACEELLREKLADARPSDAVIGEEFGGDVVHSGRQWVIDPIDGTKNFVRGVPVWATLIALLVDGEPVVGVVSAPALTRRWYAAKESGAWRSFNGGSPKRLAVSEVAELADSSISFSSLAGWLERDLREKFISLSDAAWRLRAYGDFFSYCLVAEGAVDIAAEPEVSLWDLAALSVLVSEAGGRFTSLAGEDGPHGGDAVASNGILHDAVLDALR

>HisN_Corynebacterium_ulceribovis |516654512|ref|WP_018023501.1| histidinol-phosphatase []

MSNFADDLALALELAETADAITMQRFESADLKVDAKPDMTPVSDADLAVEEALREKLSTARPADAIIGEEFGGETEFAGRQWVIDPIDGTKNFVRNVPVWATLIALLEDGKPVVGVVSAPALSRRWWAAEGSGAWRAFQNQSPRRLEVSAVNKLGDSSIALSSLTGWRDRGLRDNIIALTDDAWRLRGYGDFFSYCLVAEGAVDIAAEPEVSVWDLAPLAVLVEEAGGTFTSLAGEAGPHGGDAVASNGLLHKEVLQRIGVN

>HisN_Corynebacterium_tuscaniense |690774501|gb|KGF21924.1| histidinol phosphatase [DNF00037]

MSNYSDDLALALELADTADGITLDRFESSDLSVDSKPDMTPVTDADLAVEAALRSLLEDARPADAVLGEEYGGNVTFSGRQWVIDPIDGTKNFVRGVPVWATLIALLEDGKPVVGVVSAPALARRWYASKGSGAWRTFGDGAVRKLSVSGVDRVEDASLAISSLSGWHDRGLRENLIALTDTTWRLRGYGDFFSYCLVAEGAVDIAAEPEVSLWDLAPLAILVTEAGGRFTSLAGEDGPHGGDALATNGLLHEGVRQRLTRA

>HisN_Corynebacterium_capitovis |516648720|ref|WP_018017709.1| histidinol-phosphatase []

MSHYADDLALALELAETADAMTLDRFEAADLSVQSKPDMTPVSDADLAVEKALRARLDEARPSDAVLGEEFGGDVQLEGRQWVIDPIDGTKNFVRGVPVWATLIALLVDGTPVAGVVSAPALARRWYASAEAGAWRTFNGGALKRLKVSEVADLSDASVSMSSLEGWTKRGLRDTFLELTDKAWRLRGYGDFLSYCLVAEGAVDIAAEPEVSLWDLAPLSLLVTEAGGTFTSLSGEAGPHGGDAVASNGLLHDVALREITGA

>Corynebacterium_vitaeruminis |582018832|gb|AHI22175.1| histidinolphosphate phosphatase [DSM 20294]

MTYSDDLAFALTLADLADELTLSRFEASDLHVDSKPDMTPVSDADIATEKLLRARIEKDRPTDAILGEEFGGDVALEGRQWIIDPIDGTKNYVRGVPVWATLIALLVDGRPVVGVVSAPALARRWWAAEGEGAFRTFAGGEPKRLHVSGVSTIEDASVSFSSLDGWRDRELLPGFLGLSDDTWRLRGFGDFFSYCLVAEGAVDIAAEPEVSLWDLAPLAILVEEAGGRFTSLAGVDGPHGGDALATNGLLHEAVRARLS

>HisN_Corynebacterium_genitalium |491433730|ref|WP_005291523.1| histidinol-phosphatase []

MTTYSDDLALALELAELADGITLDRFESADLKVDSKPDMTPVSDADIAVEAALRAKLESARPSDAVLGEEFGGDVQFEGRQWVIDPIDGTKNFVRGVPVWATLIALLEDGKPVVGVVSAPALARRWYASQGSGAWRTFADGSVKRLGVSGVEGLEDASISISSLSGWRDRDLRDQLISLTDDVWRLRGYGDFFSYCLVAEGAVDIAAEPEVSLWDLAALSVLVTEAGGRFTSRAGEDGPHGGDAVATNNLLHDAVLRRLS

>HisN_Corynebacterium_ciconiae |516651190|ref|WP_018020179.1| hypothetical protein []

MPTYAEDLALALELADIADGITTDRFHAADLKVESKPDLTPVSDADTACEQALRERLSTDRPQDSILGEEFGGDYEVAGRQWVIDPIDGTKNFVRGVPVWCTLISLLVDGTPVVGVVSAPALSRRWWASSGQGAWTQFQGASPRRIEVSRVSNLADASVSFSSLAGWQERGQLEEFVRLSEDTWRLRGYGDFFSYALLAEGAVDIALEPEVSLWDLAALSVLVTEAGGRFSSVDGEDGPHGGSALATNGLLHEELLGRFAR

>HisN_Corynebacterium_freiburgense |652648072|ref|WP_027011429.1| histidinol phosphatase []

MHKDLVIALQLADLADSITLPRFEASDLKVDSKPDMTPVSDADLACEEALRILINELAPEDEILGEEFGGSTSAGRQWVIDPIDGTKNYVRGVPVWATLIALLIDGRPVLGVISAPALGRRWWAVEGEGAWRSVFGGEPKRLGVSAISKLEDASVSFSSLDGWRDLNLRDNFFSLSDITWRLRGFGDFYSYCLVAEGAVDIAAEPEVSLWDLAPLAILVTEAGGRFTSLEGIDGPHGGNALATNGLIHEETRKILTEGR

>HisN_Corynebacterium_sputi |652663950|ref|WP_027020652.1| histidinol phosphatase []

MTSYADNLALARRLADVADSLTMSRFESSDLTVESKPDLTPVSDADLECERQLREVLADERPDDEVFGEEFGGDAVPGGRQWVIDPIDGTKNFVRGVPVWATLIALLEDGKPVVGVVSAPALGRRWWASAGVGAFRSTNVNGRSSERKLSVSAVTDLADASIAISSLSGWKDRGLREQLISLTDDAWRLRGYGDFFSYCLVAEGAVDVAAEPEVSLWDLAPLAVLVEEAGGRFTSFAGEDGPHGGDAVATNGPLHDVVLNRLR

>HisN_Corynebacterium_matruchotii |491667807|ref|WP_005524522.1| histidinol-phosphatase []

MPYRSDIDFALTLANTADAITVDRFEATDLNVSSKPDMTPVSDADVACEQELRRLITQQYPNDTIVGEEFGGDAEFAGRQWIIDPIDGTKNFVRGVPVWATLIALLVDGIPTVGVISAPALGRRWWAATGHGAFRSVNNVTKKIRVSNVSSLENASISFSSLEGWQQLGIRDRFLQLTDDTWRLRGFGDFFSYCLVAEGAVDIAAEPEVSLWDLAPLAVLVTEAGGRFTSLSGVDGPHGGSAIATNGLLHDQVLRYLSPAE

>HisN_Corynebacterium_ammoniagenes |489944137|ref|WP_003847444.1| histidinol-phosphatase []

MGKYKEDLALALELAGHADVVTMHRFEATDLSVKSKPDMTPVSDADLACEKKLRAELKKARPKDEVLGEEFGGEPVTSGRQWVIDPIDGTKNYVRGVPVWATLISLLEDGEPVVSVVSAPALRRRWYASKGAGAFRVWCGEPKQLKVSQVSKLADASVAFSSLEGWTERDLLNNFIDLSQSTWRLRGYGDFWNYCLVAEGAVDIGAEPEVSLWDLAAPSLLVTEAGGKFTNLEGVDGPAGGSAVATNGLLHSKVLDKLSK

>HisN_Corynebacterium_atypicum |667687210|gb|AIG63822.1| histidinol phosphatase []

MSSYADDLALALELADLADSITAERFEAADLSVESKPDMTPVTDADLACEEALRAKLAEARPADALIGEELGGAAEMRGRQWVIDPIDGTKNFVRGVPVWATLIALIEDGRPVVGVVSAPALGRRWYASLGSGAWRTVAGGSPKRLSVSKVRELADASLSFSSLSGWRERGMRDRFIGLTDAVWRLRGYGDFFSYCLVAEGAVDIAAEPEVSLWDLATLSVLVVEAGGEFTSLAGRPGPFGGDAVATNGALHREVLARLAAGD

>HisN_Corynebacterium_mastitidis |516826726|ref|WP_018118544.1| hypothetical protein []

MTDLELALRLADAADALTLDRFEAADLAVSAKPDMTPVSDADLACEQELRSLLAAHRPEDAVLGEEFGGSVAFHGRQWVIDPIDGTKNYVRGVPVWATLISLLIDGHPSVGVVSAPALARRWYAAEGHGAWRTFAGGSPTRVRASQVSDLRDASLSFSSLEGWTQRGLRDPFLALTERTWRLRGYGDFFSYCLVAEGAVDIAAEPEVSLWDLAALSCLVTEAGGRFTSLSGKEGPHGGNAVATNGILHEDVLAALRGTGG

>HisN_Corynebacterium_imitans |672594051|gb|AIJ33028.1| histidinol phosphatase []

MSNYADDLALALELADAADALTLDRFESADLKVESKPDMTPVSDADLAVEEALRATLSQSRPADAVLGEEFGGEAVFSGRQWVIDPIDGTKNFVRGVPVWATLIALLVDGEPVVGVISAPALARRWYASKGGGAWRSFNGGELKKLEASGVNSLADASLAMSSLAGWKERGLQEKFIGLTEQTWRLRGYGDFLGYCFVAEGAVDIAAEPEVSLWDLAALSVLVQEAGGRFTSLAGDNGPHGGDAVATNGLLHEEVLQALN

>HisN_Corynebacterium_durum |492955180|ref|WP_006063265.1| histidinol-phosphate phosphatase HisN []

MSDFSTDLAFALQLADAADAITFDRFESSTLQVDSKPDMTPVSDADLTCEGALRNLIEQHRPDDSILGEEFGGDAATHGRQWIIDPIDGTKNYVRGVPVWASLIALLEDGVPVVGVVSAPALTRRWWASSGGGAWRSFNGGKPRQLQVSKVSTLADASLSFSSFEGWETRAKQEHFLGITRDIWRLRAFGDFLSYCLVAEGSVDIAAEPEVSLWDLAPLSILVAEAGGRFTSLKGEEGPHGGDAVATNGLLHDEVLRRLTP

>HisN_Corynebacterium_lipophiloflavum |493895260|ref|WP_006841186.1| histidinol-phosphatase []

MSNYADDLALALKLADLADGITIERFEAADLNVESKPDMTPVSDADLAVEEALRAELSAARPADAVLGEEFGGEAVLEGRQWVIDPIDGTKNFVRGVPVWATLIALLVDGQPVVGVISAPALARRWYASAGAGAWRTFGAGALKRLSVSKVSALSDASVSMSSLEGWQERGLRDNFIALTEKTWRLRGYGDFLSYCFVAEGAVDIALEPEVSLWDLAALAVLVSEAGGTFTSLSGEKGPHGGDAVATNGALHEAVLEEITGA

>HisN_Corynebacterium_pseudotuberculosis |503006456|ref|WP_013241432.1| histidinol-phosphatase []

MTSYDSDLDFALSLADAADAITFPRFESADLVVDSKPDMTPVSDADLACEKALRELITARYPDDAILGEEFGGDVEFKGRQWVIDPIDATKNFVRGVPAWATLIALLVDGTPVVGVVTAPALARRWWAASGQGAWRTFNGGTPTKLSVSRIAHLDNASLSFSSLSGWQSLDLDQRFIDLTECTWRLRGFGDFWSYCLVAEGAVDIAAEPEVSLWDLAPLAVLVEEAGGTFTSLTGEPGPHGGSAVASNALLHDAALDILGTKTQKPSA

>HisN_Corynebacterium_striatum |491672108|ref|WP_005528250.1| histidinol-phosphatase []

MGKCKEDLGLALELAGHADVITMNRFEASDLSVKDKPDMTPVSDADLACEKQIREALKRSRPRDEVLGEEFGGTPTFKGRQWVIDPIDGTKNFVRGVPVWATLIALLEDGEPVVSVISAPALRRRWYAAKGGGAYRVFGGEPKRLHVSQVEKLEHASVAMSSLAGWAERSLQDRFIELTEKTWRLRGYGDFWNYCLVAEGAVDIAAEPEVSLWDLAAPTLLVTEAGGKFTCLTGEEGPHNGSAVASNGLLHDSVLGILN

>HisN_Corynebacterium_maris |529144162|ref|WP_020934146.1| histidinolphosphate phosphatase []

MSTYADDLALALELADAADSLTFERFEAADLNVESKPDMTPVSDADLATEKALREKIESARPSDAILGEEFGGEAVHEGRQWVIDPIDGTKNFVRGVPVWATLISLLVDGRPVVGVVSAPALGRRWYAAEDAGAWRTFKSGSPSRMNVSGVAELADASVSFSSLSGWADRGIRDNFIALTDATWRLRGYGDFYSYCLVAEGAVDVGAEPEVSLWDLAALDVIVREAGGVFTSVEGEAGPHGGSAVATNGLLHDAVLKILN

>HisN_Corynebacterium_doosanense |516653957|ref|WP_018022946.1| histidinol-phosphatase []

MSTYVDDLALALELAEAADAITAERFEASDLEVDTKPDMTPVSEADLATEKQIRSLIETARPADDVLGEEFGGDVTFEGRQWVIDPIDGTKNYVRGVPVWATLISLLDDGVPVVGVVSAPALGRRWWAAKDSGAWRTVHGGSPKRLSVSTVGQLDDASVSFSSLGGWSERGLRENFLDLTERTWRLRGYGDFFSYCLVAEGAVDIAAEPEVSLWDLAALSVLVTEAGGRFTSLAGEDGPHGGDALATNSLLHDEVCAVLGAS

>HisN_Corynebacterium_lubricantis |648504481|ref|WP_026196232.1| histidinol phosphatase []

MSNYADDLALALELAELADSITLERFEAADLEVNSKPDMTPVSDADLAVEETLRAKLEDARPNDAVLGEEFGGDPVFEGRQWVIDPIDGTKNFVRGVPVWATLISLLDDGVPVVGVISAPALTRRWYASAEAGAWRTFNGQALKRLSVSGVASLDDASLSFSSLSGWDEHQGEGAREKFLALTDKCWRLRGYGDFFSYCLVAEGAVDIATEPEVSLWDLAALDILVREAGGTFTSVAGEAGPHGGSAVATNGQLHADVLGALS

>HisN_Corynebacterium_diphtheriae_2 |499236984|ref|WP_010934524.1| histidinol-phosphatase []

MSYHDDLAFALQLADAADAITLARFAASDLKVSSKPDMTPVSDADIATERELRDLIAQRFPEDAILGEEFGGTAEFTGRQWIIDPIDGTKNYVRGVPVWATLIALLVDGHPVVGVVSAPAIAHRWWAAQGEGAWRSNPAQESRRIHVSNVAQLTDASVSFSSLDGWKERGLLNNFVALSDETWRLRGFGDFFSYCLVAEGAVDIAAEPEVSLWDLAPLSILVEEAGGVFSSLDGALGPHGGNALATNGLLHDEVRTMLTSSR

>HisN_Corynebacterium_pilosum |517409507|ref|WP_018581879.1| histidinol-phosphatase []

MSTYADDLALALELAELADSITLDRFEASDLEVNSKPDMTPVSDADLAVEEALREKLADARPGDSVLGEEFGGDAVLEGRQWVIDPIDGTKNFVRGVPVWATLIALLDDGVPVVGVVSAPALARRWYASQDSGAWRTFKGEGLKKLCVSGVTSLEDASLSFSSLEGWREHHDKLDQFLTLTDKCWRLRGYGDFFSYCLVAEGAVDVAAEPEVSLWDLAALSVIVGEAGGTFTSLDGKDGPHGGNAVATNGHLHQQVLDALN

>HisN_Corynebacterium_pseudogenitalium |491464551|ref|WP_005322320.1| histidinol-phosphatase []

MGKFKEDLGLALELAGHADVVTMHRFEASDLSVKEKPDMSPVSDADLTCEKHIRESLKRSRPRDEVLGEEYGGEACYKGRQWVIDPIDGTKNFVRGVPVWATLISLLEDGEPVVSVVSAPALRRRWYAAKGAGAFRVFGGEPKRLGVSHVEKLADSSLAMSSLTGWAERGLRDQFLALTDKTWRLRGYGDFWSYCLVAEGAVDIAAEPEVSLWDLAAPSLIVTEAGGTFTDLDGNPGPHGGSGVASNGLLHKHALAALQR

>HisN_Corynebacterium_ulcerans |503676926|ref|WP_013911002.1| histidinol-phosphatase []

MTSYNSDLEFALSLADAADAITFKRFDSSDLVVDSKPDMTPVSDADLACEKALRELIAARYPDDAILGEEFGGDVELKGRQWVIDPIDATKNFVRGVPAWATLIALLVDGTPVVGVVTAPALARRWWAASGQGAWRTFNGGTPTKLSVSRVADLDHASLSFSSFSGWQTLGLDQRFIDLTERTWRLRGFGDFWSYCLVAEGAVDIAAEPEVSLWDLAPLTVLVEEAGGTFTSLTGERGPHGGSAIASNGLLHAPALDVLGTQKQESRD

>HisN_Corynebacterium_tuberculostearicum |491468755|ref|WP_005326513.1| histidinol-phosphatase []

MGKFKEDLGLALELAGHADVVTMHRFEASDLSVKEKPDMSPVSDADLTCEKHIRESLKRSRPRDEVLGEEYGGEACYKGRQWVIDPIDGTKNFVRGVPVWATLISLLEDGEPVVSVVSAPALRRRWYAAKGAGAYRVFGGEPKRLGVSHVEKLADSSLAMSSLTGWAERGLRDRFLALTDKTWRLRGYGDFWSYCLVAEGAVDIAAEPEVSLWDLAAPSLIVTEAGGTFTDLDGNPGPHGGSGVASNGLLHKHALAALQG

>HisN_Corynebacterium_accolens |491419800|ref|WP_005277599.1| histidinol-phosphatase []

MGKFKEDLGLALELAGHADVVTMHRFEATDLSVKEKPDLTPVSDADLNCEKHIRDSLKRSRPRDEVLGEEFGGQACYEGRQWVIDPIDGTKNFVRGVPVWATLISLLEDGEPVVSVVSAPALRRRWYAAKGAGAYRVFGGEPKRLHVSQIEKLGHASLAMSSLTGWSERGLRDKFLNLTDKTWRLRGYGDFWSYCLVAEGAVDIATEPEVSLWDLAAPSLLVTEAGGTFTDLSGNPGPHGGSAIASNGRLHKAALEMLQD

>HisN_Corynebacterium_aurimucosum |497874947|ref|WP_010189103.1| histidinol-phosphatase []

MGKYKEDLGLALEMAGHADAVTMHRFEAADLSVKDKPDMTPVSDADLACEKAIRDALKRSRPRDEVLGEEFGGEACYEGRQWVIDPIDGTKNFVRGVPVWATLIALLEDGEPVVSVISAPALRRRWYAAKGGGAYRVFGGEPKRLRVSEVSNLEDASLAMSSLAGWGLHRDTFISLTDKVWRLRGYGDFWNYCLVAEGAVDIAAEPEVSLWDLAAPALLVTEAGGRFTNVRGEDGPHGGSALVTNGTLHKAALSALS

>HisN_Corynebacterium_amycolatum |491653873|ref|WP_005510593.1| histidinol-phosphatase []

MTTSYSEDLALALSLADAADDLTMSRFEAANLVVESKPDLTPVSDADIACEKLLREILSDKRPSDEVLGEEFGGEAVFEGRQWVIDPIDGTKNFVRNVPVWATLIALLEDGKPVVGVVSAPALGRRWWASKGDGAFKRVQVGGREATERRIEVSKVADIADCSISNSSLTGWAKRNLRDNFIGLTDDAWRLRGYGDFFSYCLVAEGAVDVAAEPEVSLWDLAALAILVEEAGGRFTSLAGVDGPHGGDAVATNGLLHDSVVKRLDG

>HisN_Corynebacterium_urealyticum |501329118|ref|WP_012360753.1| histidinol-phosphatase []

MSTTYSDDLSLAFELANAADEITMARFEAEDLRIESKPDLTPVSDADTAVERRLREILADSRPDDFVLGEEFGGEAHFEGRQWVIDPIDGTKNFVRGVPAWATLISLLEDGVPKVGLVSAPALCRRWWAAPGAGAWRSFSVPNTFDEDSTPSAQPRRLSVSGVGKIADASLSISSLSGWQDRGLREQLVGLSDQTWRLRGFGDFWSYMMVAEGSVDIAAEPEVSLWDLAALVPIVTEAGGTFTNVEGTPGPHGGSAVATNGLLHEDVIAALNK

>HisN_Corynebacterium_sp._ATCC_6931 |685194952|gb|AIN83118.1| histidinol-phosphatase []

MTTSYSEDLALALSLADAADDLTMSRFEAANLVVESKPDLTPVSDADIACEKLLREILSDKRPSDEVLGEEFGGEAVFEGRQWVIDPIDGTKNFVRNVPVWATLIALLEDGKPVVGVVSAPALGRRWWASKGDGAFKRVQVGGREAAESRIEVSKVADVADCSISNSSLTGWAERDLRDNFIGLTDDAWRLRGYGDFFSYCLVAEGAVDVAAEPEVSLWDLAALAILVEEAGGRFTSLAGVNGPHGGDAVATNGLLHDAVVKRLNR

>HisN_Corynebacterium_casei |493877062|ref|WP_006823436.1| histidinol-phosphatase []

MGKYKEDLGLALELAGHADVVTMHRFEASDLSVKEKPDMTPVSDADLACEKKLRAELKKARPQDEVLGEEFGGDAVTKGRQWVIDPIDGTKNYVRGVPVWATLISLLEDGVPVVSVVSAPALRRRWYASKGAGAFRVWCGEPKQLKVSEVSRLYDASLAFSSLSGWGERNMLNNFINLTESTWRLRGYGDFWNYCLVAEGAVDIATEPEVSLWDLAAPSLLVTEAGGTFTDLDGNEGPAGGSAIATNGLLHEKVLSKLNK

>HisN_Corynebacterium_jeikeium |491436540|ref|WP_005294333.1| histidinol-phosphatase []

MTDSVSPYADDLTLALSLADAADAITMARFEANDLSVESKPDLTPVSDADTAVEKELRELIAAHHPEDALLGEEFGGDVTFAGRQWVIDPIDGTKNFVRGVPVWATLISLLVDGKPVVGVVSAPALGRRWWAAEGAGAWRLFNTPASAAATGGTGNTGDTGDTGSTGNADGGMPSVGARRLEVSKVAQVADSSIAISSLSGWADCGKREQLISLTDSAWRLRGYGDFWSYMLVAEGAVDIAAEPEVSLWDLAALAPIITEAGGRFTSLDGQDGPHGGSAV

ATNGLLHEATLSALE

>HisN_Corynebacterium_auriscanis |698028362|gb|KGM19038.1| histidinol phosphatase []

MTESPSSASSSYSADLTLALSLADAADHITMSRFESTDLRVETKPDLTPVSDADTAVEQELRSIIATHYPEDEILGEEFGGSATFQGRQWVIDPIDGTKNFVRGVPVWATLIALLEDGKPVVGVVSAPALTRRWWAADTMGAWRSFSTPAALGADHPIPSAEPRSLRVSGVTSIGDSSVAISSLSGWADCGLREQLISLTDDAWRLRGYGDFWSYMLVAEGAVDIAAEPEVSLWDLAALVPIISEAGGRFTSVTGEEGPHGGSALATNGALHEAVLGYLAAEK

>HisN_Corynebacterium_terpenotabidum |521254047|ref|WP_020440612.1| hypothetical protein []

MTTPSTLYSDDLTLALNIANAADAVTMSRFEAADLAVESKPDLTPVSDADTATERMVREMISARRPDDAILGEEFGGDVEMVGRQWVVDPIDGTKNFVRGVPVWATLISLLVDGVPVVGVVSAPALSRRWWAAEGMGAWRAFGADIARQMQVSKVAAVADASISLSSLTGWKDAGLRDELLALTDTAWRLRGFGDFWSYCMVAEGTVDVAAEPEVNLWDLAALDIIVREAGGTFTSLSGEPGPHAGSAVGSNGLLHTDVLAALGSGTAT

>HisN_Corynebacterium_nuruki |497807811|ref|WP_010121995.1| histidinol-phosphatase []

MTTPTSPYAEDLTLALTLADSADALTMDRFEAADLEVHAKPDLSPVSDADTATEQKIRDLLAVQRPGDAVLGEEFGGDVEFSGRQWVVDPIDGTKNYVRGVPVWATLIALLVDGVPVVGVVSAPALARRWWAADGMGAWRTFGAAGAADARPMHVSGVADIADASISLSSLEGWKVVGRRDELIALTDSAWRLRGFGDFWSYCMVAEGAVDVAAEPEVNLWDLAALDIIVREAGGTFTSLDGTAGPHGGSAVGSNGRLQDAVLAALRPAAPTSASVDQ

>HisN_Corynebacterium_glycinophilum |610394005|gb|AHW64968.1| Histidinol-phosphatase [AJ 3170]

MSAPTSDCPDYSDDLTLALYLADVADKVTMDRFGAGDLEITTKPDLTPVSDADTEVERQLREILSTRRPDDAVLGEEFGGDTGDATRTGRQWVIDPIDGTKNFVRGVPVWATLISLVIDGTPVVGVVSAPGLARRWWAAEQMGAWAVFNTGEARRISVSKVSDIADSSISLSSLEGWKKVGRRDELIALTDAAWRLRGYGDFWSYCMVAEGTVDIAAEPEVNLWDLAALDILVREAGGQFTALDGTPGPHDGSAVASNGLLQERVLSALDAGQNPAT

>HisN_Corynebacterium_glucuronolyticum |491537539|ref|WP_005395159.1| histidinol-phosphatase []

MLKQDLALAISLAEAADAITLDRFQAQDLNVATKPDLTPVSDADLAVEKTLRGIIERERPDDDILGEEYGGTPATTGRQWVIDPIDGTKSFVRGVPIWATLIALCDGHTPVLGVVSAPALSRRWWATDGGGAFLNGSPLHVSKVSTLADASLGFSSLSGWADRGLREKFVDLTDAVWRIRGYGDFWSYCLVAEGAIDIATEPEVNLWDLAALDILVREAGGTFTSLDGEPGPYGGSAVATNGLLHADVLRALR

>HisN_Corynebacterium_variabile |503775199|ref|WP_014009221.1| histidinol-phosphatase []

MSQPPAASPNHTYADDLNLALNIANAADAITMDRFGAGDLEVHAKPDLTPVTDADTATEQKVRDLISTHRPEDAVLGEEFGGDAGKAGRQWVVDPIDGTKNYVRGVPVWATLISLLVDGVPVVGVVSAPALARRWWAAEGMGAWRTFSAGGTDTARRMHVSKVADVADASVSLSSLTGWRDAGLRDELITLTDSAWRLRGYGDFWSYCMVAEGTVDVAAEPEVNLWDLAALDIIVREAGGTFTSLSGVPGPDQGSAVGSNGLLHDDVLAALGGGVGGVGGGAHGETGAK

>HisN_Corynebacterium_argentoratense |536922423|ref|WP_020975732.1| hypothetical protein []

MPTASSSTGASGGAYAAELALALKLADVADAVTFPRFQASDLSVDTKPDMTPVSDADLACERALRTELQRLCPDDEVLGEEFGGTASFSGRQWVIDPIDATKNYVRGVPVWATLIALLVDGKPVVGVVSAPALGRRWWASAGDGAFRQALGKPAEKISVSKVARIEDASVSFSSLDGWRDCGKLDAFLQLSDVTWRLRGFGDFYSYCLVAEGAVDVGLEPEVSLWDLAPLAILVEEAGGTFTNVAGAPGPHGGSAVATNGALHDAVLRTLNT

>HisN_Corynebacterium_falsenii |578014389|gb|AHI02647.1| histidinol-phosphatase [DSM 44353]

MTDSASSPYANDLTLALSLADTADAITMARFEASDLTVESKPDLTPVSDADKAVEEEIRAILQAHRPDDAILGEEFGGEAIFEGRQWVIDPIDGTKNFVRDVPVWATLISLLVDGTPVLGVVSAPALARRWWAARDMGAWRSFTTTPTRGGHPVPSSGARKLAVSSVKTIGDSSVAISSLSGWADAGKRQQLLDLTDRAWRLRGYGDFWSYMLVAEGAVDIAAEPEVSLWDLAALVPIVEEAGGTFTAVDGTPGPHGGSAVATNGLLHEDTISALAD

>HisN_Corynebacterium_propinquum |516831444|ref|WP_018121033.1| hypothetical protein []

MTNPTSLNSTGSAYAEDLALALELADRADGVTLHRFNSADLQVQSKPDMTPVSDADISCEEMLREHLGGARHKDAILGEEMGGTPVFSGRQWVIDPIDGTKNYVRGVPVWATLIALLVDGEPVVGVVSAPALGRRWYAAKDHGAFRRVFDSEPDPLSVSEVRELDDASVSFSSLAGWQERDKLGNFVELSEKTWRLRGFGDFWSYCLVAEGAADAAAEPEVSLWDLAALDIIVREAGGNFTSLDGKQGPHGGSAVATNGHLHESVLGLLG

>HisN_Corynebacterium_bovis |497952517|ref|WP_010266673.1| histidinol-phosphatase []

MTNTIPVSDDLTLALSLASTADTITMNRFEAADLKVETKPDLSPVSDADTATERELRRLLEAHRPDDAILGEEFGGDVEKVGRQWVIDPIDGTKNFVRGVPVWATLIGLLVDGVATVGVVSAPALGRRWWAAAGMGAWRDTSVGSLDSGPSRLEVSGVKDVADASLSISSLLGWEKIGRREQLVALGDRTWRLRGFGDFWSYCMVAEGAVDIAAEPEVSLWDLAALDVLVREAGGRFTSVDGEDGPGHGSAVATNGLLHDAVLGALGQSAR

>HisN_Corynebacterium_massiliense |551243297|ref|WP_022863646.1| histidinol-phosphatase []

MASYKDDLCYALELAGHADALTMHRFEAADLSVNKKPDMSPVTDADIACEKFMRDDIAAYSPGDEILGEEFGGEPAFSGRQWVIDPIDGTKNFVRGVPVWATLIALLEDGEPVVGVVSAPALRRRWYASKDGGAYRIFGGAPKQLEVSQVAKLADASLAISSLSSWALRGKRNDLVELTDKTWRLRGYGDFWNYCLVAEGAVDIAAEPEVSPWDLPAPSLIVTEAGGTFTNLQGKPSPQGGEKGAIATNGILHRDALKFLRVE

>HisN_Corynebacterium_pyruviciproducens |512623519|ref|WP_016458730.1| histidinol-phosphatase []

MNSDLDLALSLADAADAITMDRFQAADLHVDSKPDLTPVSDADLTVEKTLRALLAKHRPDDDILGEEFGGLPAETGRQWVIDPIDGTKSYVRGVPIWATLIALCDGHTPVLGVVSAPALQRRWWATAGGGAFADSLPLHVSGVSSLADASLGFSSLSGWADRGLREKFIALTDATWRLRGYGDFWSYCLVAEGAIDIAAEPEVNLWDLAALDILVREAGGTFTSLDGTPGPYGGSAVATNGLLHEDVLAALS

>HisN_Corynebacterium_kroppenstedtii |502205398|ref|WP_012732068.1| histidinol phosphate phosphatase [Corynebacterium kroppenstedtii]

MTEMVSALAKTFAIAREGVDDQRLARDIVYNAGRLAWRLREGKQARGGLATEYKTSVSDVVTEADRAAEQFVAEALELLRPDDGVVGEEGASRPSKSGRVWVIDPVDGTYNFTRGSDYWCSAIALVEGSDGSPEAVAENPDRLILGAVHRPAMGYTWIGGPSIPTTLDDKPLPRLDATNHSADHECLGTYLHPTFMKQPGIRSAWMAVVEEFASVRMLGSGSVDLASVAHGELGAWVQHSVKSWDWLPGKALVEGVGGTAHRVDAQGVTWSIAGPTAVVDTIDQALRTGGTSSPASSTAAPASSFIADEGESADSAREDPLPWASSAYGEDVDRALRLADAADAITMKRFLSSELQVSAKPDMTPVSDADLDTERALRGLLSTECPDDEVLGEEFGGEPVLPGRQWVIDPIDGTKNYVRGVPVWATLISLMVDGYPKVGVVSAPALGRRWIGITGEAAWALGNGTASSSALRKLSVSTVSNVGDSSISMSSLEGWKDRGLREAFVSLTDDAWRLRGFGDFYSYCLVAEGAVDIAAEPEVSLWDLAALVPVVEGAGGTFTSLAGESGPQGGDALATNGLLHEAARRRLTDQR

>HisN_Dietzia_cinnamea |494905143|ref|WP_007631188.1| hypothetical protein [Dietzia cinnamea]

MTPRPTRPLDPDLTEHLDELLAASGRIARRHFYGDLAALAATDKGAVASNGAAVGGNGHRYDPVTEADRAIEELLRAGISSMSPGDRVVGEENGVTGPADAARTWYLDPIDGTKAFLTGMTGWGTLVGVVEDGRAVAGWMDQPVLGETFSAVHGRATVRRRTNGPEAIDLRVSGCTELSEAIMYTTHPSMFGDDGEVRSRYDDLGRRVRLQRFGGDCYAYCMLAAGRVDLVVEADLKSYDIVALIPIIEAAGGVITGPDGRQPLEGGTVVAAATPELAEQAWAVLRP

>HisN_Dietzia_alimentaria |498226534|ref|WP_010540690.1| hypothetical protein [Dietzia alimentaria]

MTQRPARPLDADLTERLDELLAASGRIARRHFHADLESLEASDKNSGGSGSAAGGGYDPVTEADRAIEELLRAGISRMSPGDRVVGEENGATGPVDARRTWYLDPIDGTKAFLTGMSGWGTLVGVVEDGRAVAGWMDQPVLGETFSAVNGRAVVRRPNADTGNTVVADLRVSGCAELSDAILYTTHPSMFGERGEDRDRYDELGRRVRLQRFGGDCYAYCMLAAGRVDLVVEADLNSYDIVALIPIIEAAGGVITGPGGGQPLEGGTVVAAATPELAEQAWAVLRP

>HisN_Dietzia_sp._UCD-THP |516446830|ref|WP_017835742.1| hypothetical protein [Dietzia sp. UCD-THP]

MTARPDRPLDPDLIEHLDELLAATGRIARRHFHGELRDLVPEDKGGARGYDPVTEADRDIEALLRAGISSMAPGDRVVGEENGESGPSDASRTWYLDPIDGTKAFMTGMAGWGTLVGVVENGRAVAGWMDQPVLGETFSAVHGRSTVRRRSDGPEAFDLHVSGCDELSEAIMYTTHPSMFGDDGGLRRRYDDLASRVRLQRFGGDCYAYCMLAAGRVDLVVESDLNPYDIIALIPIVEAAGGVITGPDGRQPLDGGTVVAAATPALAEQAWEVLGADPR

>HisN_Gemmata_sp._IIL30 |918689972|ref|WP_052559975.1| histidinol phosphate phosphatase [Gemmata sp. IIL30]

MNADWRTRYDLAVNAAHKAGDLARTYYETTFEVEHKSDSSPVTIADKSAEKLIREAVSAAFPADGFLGEEFGNQPGTSGFRWIIDPIDGTKSFIRHVPIWATLIGLEYQGEQIGGVVYIPVFGMTYRALRGDGAYHNERRIRVSDVSTLADASLCYSSMGWFSRAGREKVFHDLYGKTKRQRGHGDFYGFVLVAEGAADIMLEHGVNPWDVAATKPIVEEAGGAFTDWNGVPTIHTPDVLATNGKLHATVVEMLRG

>HisN_Rhodopirellula_baltica |494561084|ref|WP_007335828.1| inositol monophosphatase [Rhodopirellula baltica]

MSDWTPTQWQSEHDGRLTAMVDIALKAGQHTLTHYGKPSLSVDRKSDDSPVTIADREAEQLVRKLVAEQFPDDAIAGEEFADSEGASRYRWVVDPIDGTKSFICGVPLYSTLLALECDETPIGGVIYLPATDQIVVAALGGGCFHSDDLKTWREARVSEQTDLSKAVFVTSEAKSFGDRGEGPRGDTDVFEALQRDTWLTRTWGDGYGYAMVATGRADLMVDPICNAWDVAAMAPIMSEAGGRFTSWKGIDTVRGGDGVGTNGHLHDAVLALLKK

>HisN_Rhodopirellula_islandica |836669541|ref|WP_047817513.1| inositol monophosphatase [Rhodopirellula islandica]

MSDWTPAQWQSEHGGRLTAMVDIALKAGQHTLTHFGKASLSVDRKSDDSPVTIADREAEQLVRQLVAEQFPEDAIAGEEFADSEGVSRYRWVVDPIDGTKSFICGVPLYSTLLALECDETPFGGVIYLPATDQIVVAALGSGCYHSDDLKTWSEAHVSEQTDLSKAVFVTSEAKSFGDRGDGPRGDSDVFDALQRDTWLTRTWGDGYGYAMVATGRADLMVDPICNAWDVAAMAPILSEAGGRFTSWKGIDTVRGGDGVGTNGHLHEAVLALLKK

>HisN_Rhodopirellula_sallentina |495951974|ref|WP_008676553.1| inositol monophosphatase [Rhodopirellula sallentina]

MQSQELFDQWRAPHAGRLTAMIEVGLAAGAKTLEHFQNRSLEVIRKGDDSPVTIADREAELRTRELLAEMFPGDTIAGEEFAEQTGDNEYRWTVDPIDGTKSFICGVPLYSTLLAIEHAGQPLGGMILIPALGQAVVGAIGHGAFYAAGLPSTASKLDSDTSIDWTPAKVSKRDKLSDAIFVTSEVGSFGKRGNANAYQRLEEACFLSRTWGDGYGYLMVATGRADVMVDPICNAWDVAAMLPILTEAGGRFTDWTGTPTVRGGDGVGTNAVLHDQVIDLLR

>HisN_Chlorobium_ferrooxidans |750129652|ref|WP_040433730.1| histidinol-phosphatase [Chlorobium ferrooxidans]

MTSDLQFALEIAEQAGRLTLDYFSGKSLQVFTKRDASPVTEADRKAEELIREAIRVAYPGDGVLGEEFDERPSANNRRWIIDPIDGTKAFIHGVPLYGVLIALEVDAAVRLGVVHFPALKELYYAEKGCGAYMNGSPIAVSSIAEPQDATVVYTEKEYLLDLPSQHPVDRLRLEGGLVRGWGDCYGHMLVASGRAEVAVDKIMSPWDCAALIPVVTEAGGVCFDYRGEATIYGEGLVSANHAIGSVLLQDIANHL

>HisN_Chlorobium_sp._GBChlB |662568664|gb|KER09801.1| histidinol phosphatase [Chlorobium sp. GBChlB]

MSPDLELALEAATAAGKLTLQYFRSRSLKVDAKRDNSPVTEADRNAEKKIVSLIRSKFPKDGFLGEEFGEKPSKNQRKWIIDPIDGTKSFIHGVPLYGVMLGLEIEGEMRVGVVDLPALGETCYAETGGGAFRNVSRLVVSQVSDLSQATLLTSSESYLTDTMGKHAFDSLKKKAKLLRMWGDCYGHTLIAAGQADVMVEPKMMPWDSAALIPIIEEAGGMCFDYAGKRTIYGKGLLTANRALGEKILRSLSDKAV

>HisN_Chlorobium_chlorochromatii |499681806|ref|WP_011362540.1| histidinol-phosphatase [Chlorobium chlorochromatii]

MKNPFDNPHRLFAFTLINQASQIALTYYGNQSLKVDTKRDASPVTIADRKAEAFIRKELELHYPDDGILGEEFGEKLSQNGRRWVIDPIDGTKAFIHHVPLWGMMLALEVNGEPHLGIIAFPALGTIYHAVQGEGAYEKETPISVSSVTSVADATIVFTEKEYLLDSPSNHPVDMLRNSGGLVRGWGDCYGHMLVASGNAEVAVDKIVSPWDCAAVIPIVTEAGGCCFDYKGNKSSSGEYGLVSTNRQLGEQLLQEIAGKG

>HisN_Pelodictyon_luteolum |499677427|ref|WP_011358161.1| histidinol-phosphatase [Pelodictyon luteolum]

MHSDLHLAMHLAREAGDLTLEYFSRKSLKVDTKRDSSPVTEADRRAEELIRRGIAAQHPHDGLFGEEFDEQPSGTGRRWIIDPIDGTRAFIHGVPLYGVMIGLEVDGKMQLGVINFPALRELYHAERGGGAFMNDDPVSVSSIADTSSATIVFTEKEYLLDPPSAHPVDMLRLEAGLVRGWGDCYGHMLVASGRAEVAVDKIMSPWDCAAVIPIVEEAGGRSFDYRGMATISGEGLVSANGPIGSSLLRAISETL

>HisN_Chlorobaculum_parvum |209572696|sp|P56160.2|HISN_CHLP8 RecName: Full=Histidinol-phosphatase; Short=HolPase; AltName: Full=Histidinol-phosphate phosphatase [Chlorobaculum parvum NCIB 8327]

MTPDLQLALELAEKAGKLTLDYFGRRSLQVFSKRDDTPVTEADRNAEELIRQGISAKFPDDGLFGEEFDEHPSGNGRRWIIDPIDGTRSFIHGVPLYGVMIALEVEGAMQLGVINFPALGELYQAERGSGAFMNGSPVQVSAIAENSASTVVFTEKEYLLDPPSNHPVDQLRIDAGLVRGWGDCYGHMLVASGRAEVAVDKIMSPWDCAAVIPIVEEAGGCCFDYRGRQSIIDGEGLVSANNAMGRNLIAAIGNGERDR

>HisN_Candidatus_Entotheonella_sp._TSY2 |575419400|gb|ETX03935.1| hypothetical protein ETSY2_31665 [Candidatus Entotheonella sp. TSY2]

MPALPMPLEAAIRAARAAGEVILPYFRTSLQVETKTDQSPVTVADRAAEQVIVETLQSQFPDYGFLGEEFGEQAGTTDARWIIDPIDGTKNFIRGIPYFATLLALEEAGDITHGVIYAPAEQTLFYAAKGFGAFTNGHQPLEVSSIDELSQAMLVHGGLDILRQEGYWNGFTRLIDATARQRGFGDYFAHTFVFRGQAEVMVEADVKPWDLAPLKIIAEEAGGRLTDFTGQPTIYGGNAIVSNRRVHAAVLNLLNPVSDSL

>HisN_Anaeromyxobacter_sp._PSR-1 |775302969|dbj|GAO02624.1| histidinol-phosphatase [Anaeromyxobacter sp. PSR-1]

MPELDLQRAMDTARAAAEAASAASLAHFRRGVRVERKPDRSPVTAADRESEAAVLAVVRAAFPDHGFLGEETGEHAGAGETRWIVDPLDGTKGFTRGRGFWGPLVACEHRGEIVAGAMALPALGEVYWAARGLGTWLRAGGAAPARLRVSGLAAWEDATLSLGEPHVLFRPPMLERVAALAISAQAARCYGDLAGCALVLKGEAEAWVEAGVNLWDLAPLKILVEEAGGRFTDLDGRPTVASGSCVASNGLVHDHVLRALAAR

>HisN_Anaeromyxobacter_dehalogenans |501751880|ref|WP_012633785.1| inositol phosphatase [Anaeromyxobacter dehalogenans]

MPELDLQRAMETARAAVEAASAASLAHFRRGVRVERKPDRSPVTAADRESEAAVLAVVRAAFPDHGFLGEETGEHAGSAETRWIVDPLDGTKGFTRGRGFWGPLVACEHRGEIVAGAMALPALGEAYWAARGQGTWLRAGDAAPARLRVSGLAAWEDATLSLGEPHVLFRPPMLERVAALAISAQAARCYGDLAGCALVLKGEAEAWVEAGVNLWDLAPLKILAEEAGGRFTDLDGRPTVASGSCVASNGLLHDHVLRALSGR

>HisN_Roseiflexus_sp._RS-1 |500597011|ref|WP_011958397.1| histidinol phosphate phosphatase [Roseiflexus sp. RS-1]

MASETLDALREFAADLAWHAGRLTLRYFQTGLTPDIKDDQTPVTVADREAERLMRRMIEDRYPHHSILGEEEGETRPGASHRWILDPIDGTKSFVQGVPLYGVLVGLERDGESVVGAVSFPALGDFLTAAKGQGCLWNGRRARVSPVSELRQATLLSSDAESMAPHGRESAYRRLAGSVRLVRTWGDAYGYSLVATGRAEIMIDPVMSVWDCAALFPIVTEAGGTFTDWNGIPTIHAGEAIGTNSVLLEQVLQAIRQG

>HisN_Herpetosiphon_geysericola |936199202|ref|WP_054533340.1| histidinol phosphate phosphatase [Herpetosiphon geysericola]

MPSLRELLDVATEAAYLGGRRTLAYFGADVQVETKGDSTPVTRADREAETIIRECIGRYFPTHTIIGEEHGEQQGDADYRWIIDPIDGTKTFIHGVPFYGVLIGLEVKGQASVGAVYLPAFDEMLAAADGLGCSWNGRPAHVSKVDTLAEATLLTTSVTSAMKRSDAYEQLVRKTKLQRTWGDCYGYVLVATGRAEIMLDPAMNPWDCAPLLPILREAGGHFTTWAGEASIWGADAVATNAALQQEVLAILANERRQQ

>HisN_Spirochaeta_sp._JC230 |917471857|ref|WP_052078274.1| hypothetical protein [Spirochaeta sp. JC230]

MVDFDLPPMRKESLLQFAQDIAKQAGDSTKSFFKTDLHISRKSDESPVTEADIHAEKLLRELISEHYPDHGIIGEEFEEKNPTDGCLFQWVVDPIDGTKSFIHGIPLYTTLIALLYDGKPSIGVIYNPILNELTSACVGVGAFYNQRRIAVSEEASLSKAWLQVTDPTDLLRRYPDSGAELISSVGFTRTWADGHGYALLARGEADIMIDPIVNLWDVACLHPIITEAGGRITDLSGNPGLGTSALAANPALHGKVLELFNKNEYYV

>HisN_Spirochaeta_africana |504268499|ref|WP_014455601.1| inositol monophosphatase/fructose-1,6-bisphosphatase family protein [Spirochaeta africana]

MSTSDWNRIPAQLRHDLWLFAEELALAGGQKSLEFFAKDIGVEWKTDQSPVTIADRETELSMRQMIAERYPDHAIHGEEYGAAPHKEFTWVLDPIDGTKSFISGVPLYCTLAALLYQGTPVIGVIVNPPSGELVSGCMNSGTRDASGDPVRVSASPAPQLRLYTSDYEGLRAAEPDWLQPLKDTYGPGRTVARTWGDAYGYMLVATGRGEIMLDPELKLWDVAPLHPILHEAGGILCDIQGTPATLPRSATAMSRELHQQIFG

>HisN_Rhodanobacter_spathiphylli |495084781|ref|WP_007809605.1| inositol-phosphate phosphatase [Rhodanobacter spathiphylli]

MTPDIAATALSAARDAAAAAAEVIRHYWLRGVEVELKSDATPVTVADREAEQAIRKILQAALPQASIYGEEFGLDGERGGLLWLVDPLDGTKSFVRRTPFFSTQIALMDGDELVLGVSSAPVYGETMWASAGEGAWLDGERVRVAATGEMGAASISTGNIRTLTGDSRWDALGALIRDSNRIRGYGDFCHYHLLARGSLDLVIESDVNILDVAALAVIVREAGGVFTDLDGAAPGLDTRSVLAGTPAIHAQALQRLRR

>HisN_Rhodanobacter_thiooxydans |495711188|ref|WP_008435767.1| inositol-phosphate phosphatase [Rhodanobacter thiooxydans]

MSPDIAATALAAAREAAAAAAEVIRHYWRRGVEVELKPDATPVTIADREAEQAIRKVLQAALPQASIYGEEFGLDGERGDLLWLVDPLDGTKSFVRRTPFFSTQIALLDGDELVLGVSSAPVYGETMWASVGHGAWLDGVRVGVADTAQMAQASISIGNVKTLTADARWEALGALIRDSNRIRGYGDFCHYHLLSRGSLDLVIESDVNILDIAALAVIVREAGGVFTDLDGAPPTLDTRSVLAGTPAIHAQALQRLRR

>HisN_Rhodanobacter_fulvus |494140236|ref|WP_007079988.1| inositol-phosphate phosphatase [Rhodanobacter fulvus]

MDSATLAQALAAAKEAAAAAGEVIRHYWQQGVAVELKEDATPVTIADREAEQAIRKILQAALPQASIYGEEYGLDGERGGLMWLVDPLDGTKSFVRRTPFFSTQIALMEGDELILGVSSAPIYGEVMWASAGGGAWMDGARVQVAGTSAMAQASVSTGNVKTLTTDARWDALGALIRDSNRIRGYGDFCHYHLLARGGLDLVIESDVNILDIAALAVIVREAGGVFTELDGAPLTLDTRSVLAGTPAIHAQALQRLRL

>HisN_Thioalkalivibrio_sp._AKL6 |516879961|ref|WP_018144665.1| inositol-phosphate phosphatase [Thioalkalivibrio sp. AKL6]

MEAAQAAADAAEKVIRHYYEAGVDVETKPDDTPVTRADVESEQAIRQVIREHFPDHGFYGEETGQSDMDSDYVWLIDPIDGTKSFVRRYPFFSTQIALMYKGDLILGLSNGIQFGERAWAEKGQGAFLNGDPIAVEKTTDLGRASLSTGNLKSLAQNAEAWKALGSITAEVNRTRGYGDFYHYHLLARGSIDLIVESDVNILDIAALAVIVREAGGVFTNLSGGELDLQTANVLAAATPELHRTALTRLGWKG

>HisN_Thioalkalivibrio_sp._ALE30 |517711323|ref|WP_018881531.1| inositol-phosphate phosphatase [Thioalkalivibrio sp. ALE30]

MEAAQAAADAAEKVIRHYYEAGVDVETKPDDTPVTRADVESEQAIRQVIREHFPDHGFYGEETGQSDMDSDYVWLIDPIDGTKSFVRRYPFFSTQIALMYKGELILGLSNGIQFGERAWAEKGQGAFLNGNPIAVEKTVDLGRASLSTGNLKSLAQSPEGWTALGGIMAEVNRTRGYGDFYHYHLLARGSIDLIIESDVNILDIAALAVIVREAGGVFTNLSGGELDLKTANVLAAATPELHRTALDRLGWKD

>HisN_Thioalkalivibrio_sp._ALJ16 |517702956|ref|WP_018873164.1| inositol-phosphate phosphatase [Thioalkalivibrio sp. ALJ16]

MEAAQAAADAAEKVIRHYYEAGVDVETKPDDTPVTRADVESEQAIRQVIRERFPDHGFYGEETGQSDMDSDYVWLIDPIDGTKSFVRRYPFFSTQIALMYKGELILGLSNGIQFGERAWAEKDQGAFLNGDPISVEKTTDLARASLSTGNLKTLAQHAEGWRALGGIMAEVNRTRGYGDFYHYHLLARGSIDLIIESDVNILDIAALAVIVREAGGTFTNLSGGELDLETTSVLAAATPDLHRTALNRLAWKG

>HisN_Salinisphaera_shabanensis |916732785|ref|WP_051339841.1| inositol-phosphate phosphatase [Salinisphaera shabanensis]

MQPFLDKAVEAARAAEAIIRRYYHGDFEVEHKADASPVTIADIECERAIKAVLSEAFPAHGFQGEELGREQADADYVWLIDPIDGTKSFVRGYPFFSTQIALRYRGELIVGVSNAPLFNGGEMACAAKGLGATLNGEPIQTSNIDTLAKTSLSLGNIASLAASPAWASVGELIGAVHRIRGYGDFYHYHLLASGRIDAILESDLNILDIAALTVILREAGGDITELGGDEIDLPTRSVFATNGRLRDAIAPYVANWDEARDAPKD

>HisN_Pectobacterium_atrosepticum |499406261|ref|WP_011093728.1| inositol monophosphatase [Pectobacterium atrosepticum]

MSQSLPDIAFFHELATLASQETLPRFRSLKASQIETKPKEGFRFDPVTEADREAERVIREHITCHYPEHAIMGEEFGLSGEGPVRWVLDPVDGTRPFLCGLPVWGTLIGLLHHERAVMGMMSQPFTGECFWADGSQAWRSDRQGETRLSTRKGVSLEQAILHTTAPEALSMHPTVRFAELTESTLMTRYGGECYALAMLAAGQIDICVEFALQPYDIVALIPIIEQAGGIITDLNGQRAEAGGTVVATGNPDLHPQVLAILNGSR

>HisN_Pectobacterium_wasabiae |492816185|ref|WP_005974293.1| inositol monophosphatase [Pectobacterium wasabiae]

MSQTLPDIAFFHELAALASQETLPRFRSLNANQIETKPKEGFRFDPVTEADRQAERVMREHITRHYPEHAIMGEEFGLSGEGPVRWVLDPVDGTRPFLCGLPVWGTLIGLLHHERAVMGMMSQPFTGECFWADGLQAWRSDRQGETRLSTRKGVSLKQAILHTTAPEALSMHPTVRFAELTESTLMTRYGGECYALAMLAAGQIDICVEFALQPYDIVALIPIIEQAGGVITDLNGQRAEAGGTAVATGSPELHQQVLAILNGTR

>HisN_Pectobacterium_sp._SCC3193 |504513014|ref|WP_014700116.1| Mono-phosphatase [Pectobacterium sp. SCC3193]

MSQTLPDITFFHELAALASQETLPRFRSLQANQIETKPKEGFRFDPVTEADREAERVIREHIARHYPEHAIMGEEFGLSGEGPVRWVLDPVDGTRPFLCGLPVWGTLIGLLHHERAVMGMMSQPFTGECFWADGSQAWRSDRQGETRLSSRKGVSLEQAILHTTAPEALSMHPTVRFAELTESTLMTRYGGECYALAMLAAGQIDICVEFALQPYDIVALIPIIEQAGGIITGLDGQRAEAGGTVVATGSPALHQQVLTILNGTR

>HisN_Nevskia_ramosa |551357514|ref|WP_022976870.1| inositol-phosphate phosphatase [Nevskia ramosa]

MTSPYLAAALEAAEAAAAVIRMAYRGQFSVDYKADASPVTEVDIAAEATIKAVLKSHFPDHGFYGEELGREAGDNEHLWLIDPIDGTKSFVRGYPMFSTQIALMHRGELVLGVSSAPCWDGALGEVIFAEKGQGAWCDNQRLQVSSADTLAQTTLSTGNLARLARSPAWARLGELIGQVHRIRGYGDFLHYHLLAGGKIDAIVESDVNILDIAALSVIVHEAGGVFTDLEGQPLGLDTTSVCAATPGLHQQLLDTLRWTPPA

>HisN_Dyella_jiangningensis |740830311|ref|WP_038615594.1| inositol-phosphate phosphatase [Dyella jiangningensis]

MAELNLSHALAAAREAAEAAAEVLRHYWRRGVDIELKSDDTPVTVADREAEMAIREVLHKALPEASIYGEEFGRDEGNPELLWMVDPLDGTKSFVRRTPFFSTQIALMHRGELVLGVSSAPIYGETMWASAGGGCWFEGEQVRVAATSALAQASLSIGNVKSLTADARWMELGALIRDTNRIRGYGDFCHYHLLARGSLDLVLESDVNILDIAALAVIVREAGGLFTDLDGQPLTLDSRSVLAGTPDIHEAVLKRLRSAR

>HisN_Dyella_ginsengisoli |516032154|ref|WP_017462737.1| inositol-phosphate phosphatase [Dyella ginsengisoli]

MSHPDLAPALAAAREAAAAAAEVILHYWRRGVDVELKSDDTPVTVADREAELAIRAILTRALPEAAIYGEEFGLDGDRDGLLWLVDPLDGTKSFVRRTPFFSTQIALMYRGELVLGVSSAPVYGETMWAHAGGGAWLDGNRVQVAPTATMAQAAISTGNVKTLTADARWDALGQLIRDSNRIRGYGDFCHYHLLARGGVDLVIESDVNILDVAALAVIVREAGGVFTDLEGAPPTLDTRGVLAGVPAIHAEALLRLRR

>HisN_Frateuria_sp._Soil773 |947303084|ref|WP_056008158.1| inositol-phosphate phosphatase [Frateuria sp. Soil773]

MSEPSLSQALGAAREAASAAGEVIRHYWQRGVAVELKSDATPVTVADREAEIAIRQVLRAALPEVAIHGEEFGIDGERGEWLWLVDPLDGTKSFVRRSPFFSTQIALMHRGELVLGVSSAPVYGETMWAWQGGGAWLNGRPVEKRVPVADMAEASISTGNVKSLTADARWDALGAMIRDSNRIRGYGDFCHYHLLARGSLDLVIESDVNILDIAALAVIVREAGGVFTDLEGAPLTLETRSVLAGTPAIHAQALARLEAVRAR

>HisN_Halothiobacillus_sp._XI15 |968546749|ref|WP_058574554.1| inositol-phosphate phosphatase [Halothiobacillus sp. XI15]

MQSQYLETALAAARAAEAVVRRYWQQNVEIEIKEDATPVTVADVETEKAIKAVILDAFPDHGFYGEETGKTKPEAEFTWLVDPIDGTKSFVREYPFFSTQIALMKGDELILGVSSAPVFGEYAYAEKGGGAFYMDQQARVSQVDSLADATLSLGNLTTLARQPVGWAGLATMVTEVNRTRGYGDFYHYHLLARGAIDLVVESDVNVLDVAALSVIVQEAGGVFTDLEGNGVGLETTTVCAAANPELHQIARKRLGY

>HisN_Solimonas_variicoloris |522139912|ref|WP_020651121.1| hypothetical protein [Solimonas variicoloris]

MASPHLAAALQAARAATEIIRKAYRGNFRVEYKADASPVTEVDVAAERAIKAVLREHFPDYGFYGEETGREMADAESLWLVDPIDGTKAFVRGYPIFSVQIALMRRSRIEFGLSCAPCWNEGLGELVWAERGAGAWRGLLETPLADFERLQVSAIDQLGQATLSTGNLATLARAPQWAELGRLIPQLHRIRGYGDFVHYHYLAGGKLDAVVESDVNILDIAALSVIVEEAGGRFTDLNGRPLSLETRTVLASNGRLHEAFASLAL

>HisN_Solimonas_flava |654540534|ref|WP_028008385.1| inositol-phosphate phosphatase [Solimonas flava]

MASPHLAAALQAARAATEIIRKAYRGNFRVEYKADASPVTEVDVAAERAIKAVLREHFPDYGFYGEETGREMADAESLWLVDPIDGTKAFVRGYPIFSVQIALMRGSSIEFGLSCAPCWNEGLGELVWAERGAGAWRGLLETPLADFERLQVSSVDRLSQATLSTGNLATLARAPQWAELGRLIPQLHRIRGYGDFVHYHYLAGGKLDAVVESDVNILDIAALSVIVEEAGGRFTDLNGRPLSLETRTVLASNGHVHEAFAGLAL

>HisN_Providencia_rettgeri |491049199|ref|WP_004910850.1| histidinol phosphate phosphatase [Providencia rettgeri]

MSHSQLPDSFFLHELANTAGKISLEYYRTNELSVIENKPKANYRFDPVTQADKLAEKAMRELISKHYPTHSIMGEEYGITGTGPIQWVLDPIDGTRPFLCGLPLWANLIGLTEHGKAVMGMMSQPYIGERFWADQHGSWTSSSHGQYRLSTRKHVKLEQAILHTTSPEPIAARSDIHFMALEKQVLMTRYGGECYAMAMLAAGRIDICVEFALEPYDIVPLIPIIERAGGIVTTLDGSRAEAGGAVVATGCPYLHEQILTILNTGS

>HisN_Providencia_stuartii |921164247|ref|WP_053110150.1| histidinol-phosphate aminotransferase [Providencia stuartii]

MLAIALEAAKSVRAMTVEAFQGAGSFDYKPDRSVVTETDLRVESALREHFAQHTPDVPILGEEFGLEGDTVFESGWVIDPIDGTRAFIYGIPLFSTLIAYVEKGEPIVGVISFPAISTVVYAAKGQGCYLKTHNGLSRRLTLGTDAPKEVANAVISASGIHSSTYNQREGTTIYHLDRVVSCARDFIFANDAYQHAMVATGRLHGAIDTIMKPWDSAALIPCIREAGGDVCSLSGERDNVMFSGSLLSASTPELAEQLVKLMNP

>HisN_Morganella_sp._EGD-HP17 |738467116|ref|WP_036417721.1| inositol monophosphatase [Morganella sp. EGD-HP17]

MNTHHPDIAFFHQLANRAAQETLPRFLPGHTLSVDTKIKEGVSFDPVTDADRQAEIALREMISAAYPQHSILGEEFGLTGEGDYQWILDPVDGTRPFMLGLPVWGTLVGLTCRGTPVMGMMSQPCTGERFWADGERSWHSSPHGTYVMKTRKNITLANAILHTNSPEPMPRFPEIRLTDLMEKVLMTRFGGECYAMAMVAAGKIDLCFDYALQPYDIAAFIPIIEQAGGCVTTLDGGPAIQGGAVLASGCPRLHQQVLTILNKNI

>HisN_Beggiatoa_leptomitiformis |933513451|gb|ALG66687.1| histidinol phosphate phosphatase [Beggiatoa leptomitiformis]

MKTLINAQLFDFINQMATVSGTIIKSYFRTHLTVDDKPDTSPVTLADRAVEQALREMIEKAYPNDGIYGEEFGVKNREAAFTWVIDPIDGTKSFITGKPLFGTLIALAYEGKPILGLLDQPILRERWIGGAGLPTTLNGHPVHVRACADVKKAALYATTPAMFEGEDFTSFGRVRESVKMTVYGGDCYAYALLATGFVDLVVESSLKPYDYCALAPIVENAGGVMTDWQGQALSLESDGRVIAAGDEYTHKQALALLQA

>HisN_Brucella_melitensis |17984172|gb|AAL53289.1| extragenic suppressor protein suhb [Brucella melitensis bv. 1 str. 16M]

MHARAPCFGNRRTGLLIDKAFFSEVAAAAAAQTLPRFRQLTEVDNKYSVGFDPVTEADRAAERAIRAVIGRTFPYHGILGEEYGAENTDRSHVWIIDPVDGTRAFISGLPVWGTLVGLTVDGDARAGMMSQPFTGELFYSDGDGAYLQRGDGAPRRLCVRKNAVLEDATLFTTTPALFKGDDRKAFDRLESAVRLSRYGVDCYAFAMLAGGFVDIVVEAGLQTYDIAALIPIIEQAGGVVTRRDGGPAEQGGDIVAAATPALHQAALDLLHI

>HisN_Brucella_suis |490826711|ref|WP_004688801.1| inositol monophosphatase [Brucella suis]

MLIDKAFFSEVAAAAAAQTLPRFRQLTEVDNKYSVGFDPVTEADRAAERAIRAVIGRTFPDHGILGEEYGAENTDRSHVWIIDPVDGTRAVISGLPVWGTLVGLTVDGDARAGMMSQPFTGELFYSDGDGAYLQRGDGAPRRLCVRKNAVLEDATLFTTTPALFKGDDRKAFDRLESAVRLSRYGVDCYAFAMLAGGFVDIVVEAGLQTYDIAALIPIIEQAGGVVTRRDGGPAEQGGDIVAAATPALHQAALDLLHI

>HisN_Brucella_ovis |492865167|ref|WP_006014969.1| inositol monophosphatase [Brucella ovis]

MLIDKAFFSEVAAAAAAQTLPRFRQLTEVDNKYSVGFDPVTEADRAAERAIRAVIGRTFPDHGILGEEYRAENTDRSHVWIIDPVDGTRAFISGLPVWGTLVGLTVDGDARAGMMSQPFTGELFYSDGEGAYLQRGDGAPRRLCVRKNAVLEDATLFTTTPALFKGDDRKAFDRLESAVRLSRYGVDCYAFAMLAGGFVDIVVEAGLQTYDIAALIPIIEQAGGVVTRRDGGPAEQGGDIVAAATPALHQAALDLLHI

>HisN_Pseudomonas_pelagia |551344912|ref|WP_022964324.1| inositol monophosphatase [Pseudomonas pelagia]

MSLSVEEIEQYHVFADQLADASARAIMPHFRAALDVSNKGEQLYDPVTVADRAAERSMRELIQAVYPDHGILGEEEESFTGTSPLTWVLDPIDGTRAFITGLPLWGTLIALNDGQRPCLGVMNQPFTGERFSGSAQGTWLNGRPLRTRACSSLAEATMMATSLEIFTTRQQRGAFDHLAHQAQLVRFGGDCYAYCMLAAGLVDVIMEASLQPYDVQALIPIVEGAGGKMTTWLGGDAQHGGLVLACGDPQLHDELVSKLTALGPFG

>HisN_Pseudomonas_parafulva |969974189|ref|WP_058639247.1| inositol monophosphatase [Pseudomonas parafulva]

MPLSAEQIAEFREFAEQLADAASAAITPYFRASLEVEDKGGRLYDPVTVADKAAEDAMRERIQARYPDHGILGEEAGVAVGSSPLTWVLDPIDGTRAFITGLPLWGTLIALNDGTRPVVGVMNQPFTGERFIGTPEGAWSGTRALKTRACKELAAATLMCTTPDMFDTPARKAAFEHVASQARLMRYGGDCYAYCMLASGFVDVIVEASLQPYDVQALMPIIKGAGGVITAWDGSSAQQGGCVVACGDPALHAQVVELLRGAM

>HisN_Pseudomonas_monteilii |515085617|ref|WP_016715265.1| inositol monophosphatase [Pseudomonas monteilii]

MSLSAEQIGELRTFAEQLADAAAVAIQPYFRASLAVEDKGGRLYDPVTVADKAAEDAMRELIQARYPDHGILGEEAGVAVGSSPLTWVLDPIDGTRAFITGLPLWGTLIALNDGTRPVVGVMNQPFTGERFVGTPEGAWRSGTPLKTRACADLASATLMCTTPDMFDTAERKAAFEGVAGRARLMRYGGDCYAYCMLASGFVDVIVEASLQPYDVQALMPIIEGAGGVITAWDGSSAQHGGCVVACGDPALHAQVVEMLRHAM

>HisN_Pseudomonas_psychrotolerans |970496334|ref|WP_058773372.1| inositol monophosphatase [Pseudomonas psychrotolerans]

MSLTAAEISYYERFAERLTDAAAVAIQPYFRAQLAVDDKGDAASAGRRYDPVTIADKAAERAMRELIQAEHPDHGILGEEEENVAGESPLTWVLDPIDGTRAFITGLPLWGTLVALNDGSRPVVGVMNQPFTGERYLGTPAGAWRNGVPLHTRPCTSLAQARLMCTSRDIFDTPERLAAFDAVAAEVQLARFGGDCYAYCMLASGFVDVIIEASLQPYDVQALIPIIEGAGGRMTAWDGGDAQQGGAILACGDPALHAQLVERLKHLA

>HisN_Marinobacter_sp._BSs20148 |504685588|ref|WP_014872690.1| inositol monophosphatase [Marinobacter sp. BSs20148]

MNMNESLLKVVHETLAESGTIARHYFRQSLAVTAKRDLSPVTLADQEIEAAMRKVIHQHFPEHSIVGEEGEDQLGTGAYTWTLDPIDGTKSFISGLPLFGTLICVSREEQFLIGAIDMPILNERWIGVLGQGSTFNGTPCKASDVTDIGSATLFSTEPNMFNTGQAERLKKLESAVRLRRYGGDCYSYGLLASGHIDLVVEASLHPYDWMSAVPIIQEAGGVITDWQGNALHRNSEGSVIAAGTPELHSAALALLAGANH
